# Reproducible processing of TCGA regulatory networks

**DOI:** 10.1093/gigascience/giaf126

**Published:** 2025-10-20

**Authors:** Viola Fanfani, Katherine H Shutta, Panagiotis Mandros, Jonas Fischer, Enakshi Saha, Soel Micheletti, Chen Chen, Marouen Ben Guebila, Camila M Lopes-Ramos, John Quackenbush

**Affiliations:** Department of Biostatistics, Harvard T.H. Chan School of Public Health, Boston, MA, 02115 USA; Department of Biostatistics, Harvard T.H. Chan School of Public Health, Boston, MA, 02115 USA; Channing Division of Network Medicine, Brigham and Women’s Hospital, Boston, MA, 02115 USA; Department of Biostatistics, Harvard T.H. Chan School of Public Health, Boston, MA, 02115 USA; Department of Biostatistics, Harvard T.H. Chan School of Public Health, Boston, MA, 02115 USA; Department of Biostatistics, Harvard T.H. Chan School of Public Health, Boston, MA, 02115 USA; Department of Biostatistics, Harvard T.H. Chan School of Public Health, Boston, MA, 02115 USA; Department of Biostatistics, Harvard T.H. Chan School of Public Health, Boston, MA, 02115 USA; Department of Biostatistics, Harvard T.H. Chan School of Public Health, Boston, MA, 02115 USA; Department of Biostatistics, Harvard T.H. Chan School of Public Health, Boston, MA, 02115 USA; Channing Division of Network Medicine, Brigham and Women’s Hospital, Boston, MA, 02115 USA; Department of Medicine, Harvard Medical School, Boston, MA, 02115 USA; Department of Biostatistics, Harvard T.H. Chan School of Public Health, Boston, MA, 02115 USA; Channing Division of Network Medicine, Brigham and Women’s Hospital, Boston, MA, 02115 USA

**Keywords:** gene regulatory network, The Cancer Genome Atlas, cancer, Nextflow, *NetworkDataCompanion*, reproducibility

## Abstract

**Background:**

Technological advances in sequencing and computation have allowed deep exploration of the molecular basis of diseases. Biological networks have proven to be a valuable framework for analyzing omics data and modeling regulatory interactions between genes and proteins. Large collaborative projects, such as The Cancer Genome Atlas (TCGA), have provided a rich resource for building and validating new computational methods, resulting in a plethora of open-source software for downloading, preprocessing, and analyzing those data. However, for an end-to-end analysis of regulatory networks, a coherent and reusable workflow is essential to integrate all relevant packages into a robust pipeline.

**Findings:**

We developed tcga-data-nf, a Nextflow workflow that allows users to reproducibly infer regulatory networks from the thousands of samples in TCGA using a single command. The workflow can be divided into 3 main steps: multiomic data, such as RNA sequencing and methylation, are (i) downloaded, (ii) preprocessed, and (iii) analyzed to infer regulatory network models with the Network Zoo. The workflow is powered by the NetworkDataCompanion R package, a standalone collection of functions for managing, mapping, and filtering TCGA data. Here, we demonstrate how the pipeline can be used to investigate the differences between colon cancer subtypes attributed to epigenetic mechanisms. Lastly, we provide a database of pregenerated networks for the 10 most common cancer types that can be readily accessed by the public.

**Conclusions:**

tcga-data-nf is a complete, yet flexible and extensible, framework that enables the reproducible inference and analysis of cancer regulatory networks, bridging a gap in the current universe of software tools for analyzing TCGA data.

## Graphical Abstract

**Graphical Abstract:**
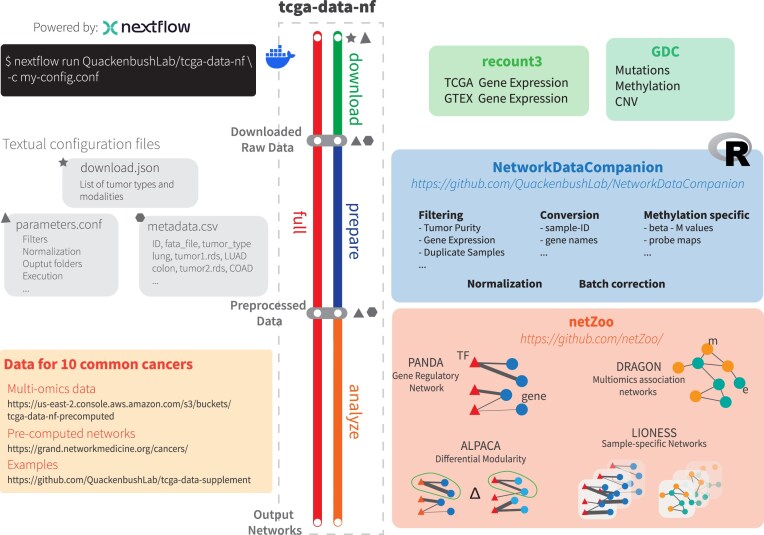
The tcga-data-nf Nextflow workflow orchestrates the key Download, Preprocess, and Analyze steps (represented in the center of the figure) that allow users to reproducibly generate cancer Gene Regulatory Networks (GRNs). With a single command line call and plain text configuration files, the workflow generates intermediate processed files, GRNs, and metadata. Left: Examples of workflow inputs and outputs, alongside key resources that the user can access to analyze ten common cancers and to reproduce all results in this manuscript. Right: Key computational resources and methods that are used in tcga-data-nf. We developed the NetworkDataCompanion R package to easily map and process the downloaded data. Once prepared, these files can be used with the Network Zoo computational methods (PANDA, DRAGON, ALPACA, LIONESS) to infer networks.

## Background

There is a growing recognition of the importance of ensuring that scientific research is reproducible and that analytical steps are transparent [1, 2]. The field of bioinformatics has been particularly receptive to this trend, with many prominent scientists and journals advocating for the use of open-source software, open data, and reproducible methods [3, 4]. Projects such as Bioconductor [5] and Bioconda [6] facilitate the sharing and reuse of bioinformatics software. The Galaxy project [7] has pioneered the development of platforms for training and sharing best practices for complete data analysis workflows. Workflow management tools such as Nextflow [8], Snakemake [9], and WDL [10] facilitate reproducibility of complex data analysis pipelines.

These methodological advances are closely tied to the growing availability of large-scale biological data. The falling cost of sequencing has enabled the generation of population-level omics data, allowing thousands of subjects to be profiled in a single project to investigate complex traits and diseases.

As notable examples, the UKBiobank [11] has collected multiomic and clinical data for more than 500,000 individuals representative of the UK population, and the 1000 Genomes Project [12] includes samples from more than 4,000 individuals collected to characterize human genetic variation. Ongoing data collection efforts (such as the 100,000 Genome Project [13]) demonstrate that this deluge of data is not slowing down. With the consequent massive scope for downstream analyses comes the need for reproducible and rigorously managed software pipelines to work with these data.

The Cancer Genome Atlas (TCGA) [14] was one of the first large-scale collaborative projects designed to study the molecular basis of disease, including samples collected from more than 10,000 patients with cancer representing over 30 tumor types.

TCGA data have been invaluable for the study of regulation in both healthy and tumor tissues [15–19] and for developing and benchmarking analytical methods for omics data [20, 21]. The TCGA dataset has grown in value with data from related projects, such as The Cancer Protein Atlas (TCPA) [22, 23] and the Clinical Proteomic Tumor Atlas Consortium (CPTAC) [24, 25], which create new opportunities for the development of innovative methods and applications.

Given that most pathologies result from the complex interplay among multiple genomic, transcriptomic, epigenomic, and other factors [26, 27], the inference and analysis of network models integrating multiple omics are important analytical approaches that have been enabled by large-scale omic data resources. Biological networks model the high-level organization of biological systems by representing interactions between biological entities, capturing the molecular mechanisms that define biological states and the progression between them. Most notably, network analyses have helped to elucidate the etiology and progression of tumors and provide insight into important clinical features [28–32]. Many network types have been used to explore biological processes, including protein–protein interaction networks, networks of DNA–protein interactions, and coexpression networks. Of particular utility are bipartite gene regulatory networks (GRNs), which consist of transcription factors (TFs) and the genes they regulate in a specific phenotype [33]. Comparison of GRNs across individual phenotypes can lead to the discovery of regulatory “rewiring” to create or eliminate disease-related functions and pathways [33–38]. Multiomic association networks can capture additional interactions that influence regulation by combining multimodal data. For example, combining epigenetic and transcriptomic data identified putative regulatory associations between gene expression patterns and DNA methylation in breast cancer samples [39].

For studies of cancer and the regulatory changes that drive the disease, the TCGA dataset is unique in the breadth and depth of multiomic data available from the same samples, allowing a wide range of complementary network analyses to be performed. However, accessing and analyzing the wealth of data from TCGA or other large-cohort studies, from raw data to networks, can be a nontrivial task. For example, raw sequencing data cannot be publicly released and must be aligned and quantified prior to downstream analyses, integration of multiomic data requires matching of samples across assays, and each data type has its own set of data preprocessing and filtering steps.

Several existing tools have been designed to address these challenges. The Genomic Data Commons (GDC) provides an application programming interface (API) [40, 41] that interfaces with R via the TCGAbiolinks R package [42, 43]. TCGAbiolinks provides both programmable access to the TCGA data and tools for data-wrangling tasks such as identifier mapping and data filtering based on clinical features. For example, with a few lines of code, one could download all RNA sequencing (RNA-seq) and mutation data for a specific tumor (or subtype) from subjects over 70 years of age. The TCGAbiolinks package also provides a number of functions for *ad hoc* preprocessing of the data, in addition to the most commonly used analytical steps, such as differential gene expression analysis [43, 44].

Once the data are downloaded and preprocessed, additional analyses can be performed to extract biologically meaningful insights. The Network Zoo project [45] is a growing open-source suite of tools for the inference and analysis of biological networks [46]. The 16 methods currently in netZoo include PANDA [33], which uses gene expression data together with prior TF–binding information and TF–TF interaction data to infer gene regulatory networks (GRNs) describing TF–gene interactions, and ALPACA [47], which compares the structure of 2 PANDA networks: DRAGON [39], which creates robust multiomic Gaussian graphical models (partial correlation networks), and LIONESS [48], which estimates individual networks for each sample in a population by using a leave-one-out strategy with linear interpolation.

It is then clear that a full analytical pipeline for TCGA or similar data involves a large number of data access, preprocessing, and analysis steps. Although reusable and documented pieces of software exist to handle each specific step, as detailed above, chaining these processes back-to-back from data download to final analysis yields a complex workflow with many interdependent processing decisions. Moreover, tools are required to facilitate the transfer of data and results from one step to the next. As with any programmatic workflow, each step introduces additional possibilities for issues with accuracy and reproducibility. Given the number of steps involved in network analysis of TCGA data, in particular, we propose that a single, robust, and transparent workflow is an essential tool for conducting accurate, reproducible analyses that yield meaningful results.

To this end, we developed *tcga-data-nf*, a Nextflow workflow that generates network models of TCGA data with a single command, managing all steps from data download through preprocessing to network generation. *tcga-data-nf* is structured around 3 key workflow modules: Download, Prepare, and Analyze. First, in the Download module, *tcga-data-nf* facilitates the download of TCGA clinical and phenotypic data, as well as several omics modalities, including RNA-seq, mutation, methylation, and copy number variation (CNV) data. The Prepare module involves preprocessing steps specific to each data type. Finally, the Analyze module generates individual sample GRNs and expression–methylation/CNV association networks by combining LIONESS with PANDA and DRAGON, respectively. In a detailed example, we show that *tcga-data-nf* allows us to not only swiftly generate networks for the 4 consensus subtypes of colon cancer [49] but also demonstrate the use of *tcga-data-nf* to expand our understanding of the regulatory processes driving subtype-specific prognosis. In particular, we find molecular evidence for epigenetic involvement in the more aggressive CMS4 subtype.

As part of developing *tcga-data-nf*, we created the *NetworkDataCompanion* (*NDC*). *NDC* streamlines routine steps in TCGA data processing, including filtering and mapping gene and sample identifiers between modalities (which is often a challenge with such heterogeneous data) and modality-specific data transformation, such as normalization and cleaning. While *NDC* was designed to provide necessary back-end functions for *tcga-data-nf*, it also serves as a standalone tool for separate use.

To provide users with a seamless out-of-the-box experience, *tcga-data-nf* comes equipped with all essential supplementary components, including a Docker container, conda environments, comprehensive documentation, and introductory tutorials to help users get started. As an additional illustrative example and resource for the public, we have applied *tcga-data-nf* to generate GRNs for the 10 most frequent cancer types (breast invasive carcinoma [BRCA], lung adenocarcinoma [LUAD], lung squamous cell carcinoma [LUSC], kidney renal clear cell carcinoma [KIRC], liver hepatocellular carcinoma [LIHC], pancreatic adenocarcinoma [PAAD], skin cutaneous melanoma [SKCM], stomach adenocarcinoma [STAD], colon adenocarcinoma [COAD], and prostate adenocarcinoma [PRAD]) and published these networks on the Gene Regulatory Network Database (GRAND) [50] (version 1.7) for easy access and exploration.

## Findings

The open-source *tcga-data-nf* is a Nextflow [8] pipeline that allows users to fully execute network analysis on TCGA data in a single, end-to-end pipeline spanning data download, preparation, and network inference. By chaining and combining atomic tasks, called processes, this pipeline enables users to infer GRNs and other networks with a single command. Specific tumor sources (such as BRCA or COAD) and workflow parameters are specified by the user in configuration files called by the workflow.

The whole workflow consists of 3 main steps: **Download, Prepare**, and **Analyze**, which involve downloading the raw data, preparing the data for the analysis, and inferring the networks. For **Download**, data downloaded include RNA-seq, mutation, methylation, and copy number variation data, which are the modalities used to generate regulatory networks and to characterize genetic and epigenetic aberrations that could explain dysregulation in cancer. Clinical and phenotypic data are also downloaded to support downstream investigations. For **Prepare**, gene expression and methylation data are cleaned and preprocessed. Sample duplicates, outliers, and lowly expressed genes are removed. CpG-level methylation is mapped to overall gene promoter methylation values, and sample identifiers are matched to facilitate multiomic integration. For **Analyze**, the *tcga-data-nf* pipeline provides an interface for estimating GRNs with PANDA [33] and multiomic partial correlation networks with DRAGON [39]. For both methods, *tcga-data-nf* also facilitates the generation of sample-specific networks with LIONESS [48], and ALPACA [47] is used to identify differential modules between multiple PANDA networks.

Although designed as a full pipeline that runs all the steps above, the modular design of *tcga-data-nf* allows users to run the Download, Prepare, and Analyze steps independently. The decoupling of these 3 different steps is useful from a practical standpoint; the Download step does not require efficient computational resources, but it is time-consuming and performed once to generate a long-term data repository. The Prepare step involves several choices of data preparation parameters, and a user may wish to test several configurations. The Analyze step is likely to be run multiple times in the course of an investigation, to observe how various parameters affect the results or fit new types of networks.

The Prepare step of *tcga-data-nf* is powered by the associated *NDC* R package, which provides a wide range of functions for working with different data modalities in TCGA. While there are many existing packages to work with TCGA data, it is often necessary to resort to multiple tools to carry out simple functions. The *NDC* package addresses this issue by integrating a set of tools into a single package, streamlining the routine steps of TCGA data processing. TCGA-specific functions enable users to filter and map gene and sample identifiers across modalities, addressing one of the often vexing challenges with heterogeneous data. Omic-specific functions allow users to normalize, transform, and filter data according to the specific needs of downstream tasks. Although *NDC* is explicitly designed to support *tcga-data-nf*, the package is standalone and can be reused in other applications.

State-of-the-art environment and containerization tools are also integrated with *tcga-data-nf*, which can interface with Docker [51], Singularity [52], and conda [53]. We provide containers and configuration details for both Docker and Singularity, as well as a customizable configuration structure that allows users to define conda environments.

In developing *tcga-data-nf* and *NetworkDataCompanion*, we generated PANDA and PANDA–LIONESS networks for the 10 most common tumors in TCGA. We have published these networks in GRAND, our group’s cloud-based network database, so they can be used by the broader cancer research community without the need to run the more expensive steps of the workflow. Finally, we present a complete analysis of the 4 consensus molecular subtypes of colon cancer (TCGA–COAD) that integrates the PANDA and DRAGON COAD networks to uncover key epigenetic and regulatory differences between them. Below, details of the workflow and COAD application are presented, including descriptions of the *tcga-data-nf* pipeline steps and *NetworkDataCompanion* functionalities.

### Download

For network generation and analysis, we focus on 5 TCGA data modalities: gene expression, methylation, CNV, and mutation data, alongside patient clinical data. All TCGA data are downloaded from the GDC project [54]. To download methylation, CNV, mutation, and clinical data, *tcga-data-nf* uses the “TCGAbiolinks” [43] and “GenomicDataCommons” [40] R packages. For the gene expression data, *tcga-data-nf* uses the recount3 R package [55], taking advantage of recount3’s normalization tools and standardized processing across studies. We also leverage recount3 to download GTEx gene expression data, enabling future analyses that compare cancer with normal tissue [56].

The Download step is driven by a JSON configuration file (Listing 1) that is modality-centric, which means that for each data type (gene expression, mutations, CNVs, etc.), one can specify multiple cancer types to be downloaded. It also provides users with the ability to pass a list of samples of interest, which is helpful in selecting data from a specific subpopulation or discarding problematic samples.

The Download step generates simple, comma-separated tables of metadata that store key configuration parameters used to download each dataset and the path of the resulting files. These metadata tables directly interface with the following Prepare step. A schematic of the Download step is shown in Supplementary Fig. S1. For the Download step, we have written a dedicated testing profile (“testDownload”) described in the “Testing” section that allows the workflow to be piloted and validated.

### Prepare

The raw downloaded data need to be preprocessed before being used in downstream analyses. Preprocessing requires a range of parameter choices for steps, such as normalization and filtering, leading to a large number of possible parameter configurations. Since these choices can affect the results and conclusions from any analysis [57, 58], *tcga-data-nf* provides a config file for the user to define such parameters for the preprocessing steps implemented in the Prepare step.

As implemented in *tcga-data-nf*, the Prepare step primarily deals with preprocessing gene expression and methylation data, which are the data types we used to generate GRN and multiomic association networks. However, users may also have specific preprocessing steps that are unique to their analysis. Consequently, *tcga-data-nf* is naturally extensible, such that users can easily implement their own functions to integrate with the existing Prepare step. Below, we describe the key steps used to preprocess gene expression and methylation data in the current pipeline.

**Expression** From recount3, we obtain gene-level raw count data for RNA-seq data from both TCGA and GTEx. To clean these data, we implement the following steps:

- Normalization: Raw count data are normalized, with options to generate either transcripts per million [TPM] [59, 60] or counts per million with Trimmed Mean of M-values (CPM with TMM normalized library size [61, 62]).
- Duplicates: Where duplicate samples are present (2 or more samples from the same research subject), the sample with the greatest sequencing depth is retained, and others are discarded.
- Batch correction: If specified, batch effects are removed using ComBat [63, 64]. We also visualize the effect of batch removal using PCA.
- Low expression: We remove genes that have low expression, defined as those genes with less than *n* counts in at least $p\%$ of samples for user-defined values of *n* and *p*.
- Sample purity: For TCGA tumor samples, we remove those that have low purity, using previously computed purity values [65] and allowing the user to specify their method of choice.
- Tissue type: TCGA contains both tumor and adjacent normal tissue samples, and one can save the data for these tissue types separately to facilitate subsequent analyses comparing tumor to adjacent normal tissue.

All these choices are set by appropriate parameters in the configuration file, and users can specify multiple values for each parameter, allowing *tcga-data-nf* to output results for all parameter combinations.

**Methylation** The TCGA methylation array data undergo 2 key preprocessing steps: mapping of individual CpG probes to genes to generate gene-level promoter methylation values and general data cleaning/transformation. By default, CpGs are mapped to genes using the publicly available annotation for the Illumina 450k array, which is mapped to hg38 using GENCODE v36 [66, 67]; however, users can supply their own annotation file if desired. Once CpGs are mapped to genes, the average promoter methylation for each gene is calculated. The promoter region is defined as the area 200 base pairs upstream of the transcription start site (users can redefine this boundary), and the average of methylation $beta$-values for any probes falling within this region is calculated.

It is not always necessary to perform this mapping to promoter methylation for all the genes represented on the EPIC array; in some cases, only a particular subset may be relevant for a specific analysis. For example, in the application of DRAGON below, we map the probes only to genes encoding TFs. The default behavior is to map to all genes on the array.

After obtaining gene-level methylation for each sample, the following preprocessing options are available:

- Duplicates: If a sample has multiple methylation array profiles, the user can handle these in several ways, including selecting the duplicate with the least missingness, choosing a duplicate at random, or excluding samples with duplicates altogether.
- Missing data: Any gene that has missing promoter methylation values for more than $m\%$ of the samples is removed from the analysis, with $m = 20\%$ by default. If a gene has missing values for $\le m\%$ of the samples, these missing values are estimated by mean imputation. If a user does not wish to impute any data, they may set $m=0$ to exclude all missing data.
- Conversion from $beta$ to *M*-values: The mean promoter methylation $beta$ value $\beta \in [0,1]$ is converted into an *M*-value $M \in (-\infty , \infty )$ using the following formula:
  (1) \begin{eqnarray*}
M = \log _2\left\lbrace \frac{\beta }{1-\beta }\right\rbrace
\end{eqnarray*}
  See [68] for a detailed discussion of the relative merits of using $\beta$-values versus *M*-values in methylation analyses.
- Transformation to approximate normality: We provide the option to apply a nonparanormal transformation [69] to the *M*-values to achieve approximate normality in the distribution for input into DRAGON, which requires approximately normal data. The nonparanormal transformation is powered by the huge.npn function of the R package huge [70].

A simple schematic of the Prepare step is shown in Supplementary Fig. S2. At the end of these steps, we obtain a comma-separated file with probes in rows and samples in columns. As in the Download step, a metadata table storing the parameters used for the Prepare step is also produced. The Prepare step relies heavily on the companion R package *NDC*, which is described in depth in the *NetworkDataCompanion* section.

### Analyze

Following the downloading and preparation of TCGA data, the Analyze step implements the necessary code to generate network models. The Analyze step generates 2 types of networks: GRNs, generated with PANDA, and multiomic association networks, generated with DRAGON. PANDA [33] is a GRN inference method that uses bulk expression data, together with a prior TF–gene binding network (based on TF motif mapping) and a TF–TF protein interaction network, and generates population-level bipartite TF–gene regulatory networks. DRAGON [39] is a network inference method based on Gaussian graphical models that infers partial correlations on multiomic data. We also used LIONESS [48], which estimates sample-specific networks by interpolation between a network for the entire population and that for the population, less the sample for which we are estimating a network. In our pipeline, we used LIONESS to estimate both sample-specific PANDA and sample-specific DRAGON networks for each sample. Finally, to compare the connectivity of PANDA GRNs, we use a netZoo method called ALPACA [47], which identifies the gene modules that best distinguish 2 networks by maximizing their differential modularity. We show how the workflow can be easily extended to run methods that do not belong to the netZoo suite. For example, we implemented WGCNA [71] and GENIE3 [72], both methods developed by others and not included in netZoo. A simple scheme of the Prepare step is shown in Supplementary Fig. S2.

The *analyze* workflow relies on implementations of PANDA, DRAGON, and LIONESS in the netZooPy package and ALPACA from the netZooR package [46]. The processes for network inference use the command-line interfaces and Python objects for PANDA, DRAGON, and LIONESS; these require users to specify the various input omic files and the parameters for each method. ALPACA uses the output from PANDA networks to identify condition-specific modules and only requires the unique identifiers of the inferred GRNs. Conveniently, the Prepare step in *tcga-data-nf* generates a metadata table that the users can directly use to specify which files are then used by the Analyze step. Given the storage requirements of the data the pipeline can generate, we have updated netZooPy to save networks in a hierarchical data format (HDF), which reduces the size and reading/writing time for storing the networks.

Lastly, we note that although the pipeline described here primarily uses gene expression and methylation data to generate network models, the workflow can be easily edited to include other data types and methods. As an example, we included an analysis that integrates CNV data with gene expression to estimate DRAGON networks. To demonstrate extensibility, we also include 2 externally developed network inference methods, WGCNA and GENIE3. To facilitate engagement among the broader biomedical research community, *tcga-data-nf* documentation includes clear explanations of how to extend the pipeline.

### Full

The full pipeline combines the Download, Prepare, and Analyze steps described above. It is designed to be run with a single command and represents the most comprehensive network analysis pipeline described in this article. Specifically, the full pipeline integrates PANDA, ALPACA, DRAGON, and LIONESS. It generates PANDA GRNs, which are compared with ALPACA. It also generates multiomic DRAGON methylation–expression and CNV–expression networks using DRAGON, as well as incorporates LIONESS to generate both PANDA–LIONESS and DRAGON–LIONESS sample-specific networks.

While we recommend separating the 3 steps by, for example, downloading the data once and then running the Prepare and Analyze steps as needs change, there are instances when researchers may wish to use the full pipeline to run all 3 steps for a single project at once. We illustrate this approach in the sections “Multiomic partial correlation networks identify differences between colon subtypes,” and “Regulatory differences between colon cancer subtype GRNs,” where we demonstrate how the full pipeline can be used to generate DRAGON and PANDA networks for the TCGA colon cancer consensus molecular subtypes, as well as gain insight into the differences between their regulatory programs.

For the full pipeline, the configuration files and parameters mirror those of the 3 modular pipelines above. First, a “JSON” configuration file similar to that used in the Download step (Listing 2) needs to be populated with all the data modalities of interest. Then, the processing and analysis parameters need to be specified in the Nextflow configuration file as they were in the Prepare and Analyze steps.

### NetworkDataCompanion

The *NetworkDataCompanion* (*NDC*) R package supports preprocessing of TCGA bulk RNA-seq and DNA methylation data. The purpose of having a version-controlled R package for these functions is to attain the high standard of reproducibility in the overall *tcga-data-nf* pipeline. While *NDC* is the engine behind the *tcga-data-nf* workflow processing steps, the software is standalone and can be installed and used outside the workflow. *NDC* currently provides 3 broad classes of functions: functions for mapping identifiers, functions for filtering data, and functions for preparing expression and methylation data (such as normalization and scaling). While many of the functions described below are intuitively simple, it is worth noting that the complexity of the TCGA project and its wealth of data require commensurately complex data wrangling to handle tasks such as filtering by sample quality and matching sample identifiers between omics types. The goal of *NDC* is to provide a version-controlled and unit-tested environment for developing and maintaining tools for these tasks, with the end goal of enhancing accuracy and reproducibility.

#### Mapping functions

Given the variety of data types used in the pipeline and the number of other resources with which they interface, there are many instances where we need to map between “synonymous” identifiers. We have implemented several wrapper functions that use existing tools such as GDC and TCGAutils to retrieve and convert various sample identifiers (TCGA barcodes, UUIDs). We have also implemented functions for translating gene names between Ensembl IDs [73], HUGO Gene Nomenclature [74], and Entrez IDs [75, 76] by leveraging GENCODE v26 [77], which was used for TCGA and the AnnotationDbi R package [78].

#### Sample filtering functions

By default, *tcga-data-nf* downloads and processes all samples available for a particular TCGA dataset. *NDC* provides 3 different functions that allow a user to filter these samples. First, a user may eliminate duplicate samples using one of several methods: select the sample with the strongest signal based on RNA sequencing depth (for expression duplicates), select the sample with the least missing data (for methylation duplicates), select the sample with the highest tumor purity (for any sample type for which tumor purity is available), select a single sample from a set of duplicates at random, or exclude all samples with duplicates. Second, users can filter samples based on the TCGA sample type (e.g., primary tumor tissue, metastatic tissue, or adjacent normal tissue). Finally, a user may filter samples based on tumor purity, excluding samples where the number of nontumor cells is too large according to the sample’s published annotation [65].

#### Data preparation functions

With *NDC*, users can apply common data transformations to both gene expression and methylation data. For RNA-seq data from recount3, one can normalize read counts to TPM [59] or CPM [62] and their corresponding log transformations, where a pseudocount is added to avoid undefined logs. For methylation data, functions are provided to convert methylation $beta$-values to *M*-values (logit base-2 transformed $beta$-values) and vice versa [79] and to aggregate methylation values to get gene-level information, such as average methylation within a promoter region or gene body.

Collectively, the functions in *NetworkDataCompanion* represent a comprehensive set of tools for basic processing, filtering, and mapping that are needed to clean and prepare TCGA data. We note that although there are R packages that cover each of the individual tasks described above, *NetworkDataCompanion* aggregates them into a unified solution in 1 version-controlled and unit-tested package to facilitate their use together and to allow them to be seamlessly integrated into the *tcga-data-nf* pipeline. To engage the broader research community, *NetworkDataCompanion* is hosted on GitHub and contributions are encouraged.

### Multiomic partial correlation networks identify differences between colon subtypes

Colorectal cancer affects nearly 2 million individuals worldwide each year and is forecast to increase in incidence by $84\%$ in the next 20 years [80]. Molecular profiling studies have identified 4 major expression-based consensus subtypes (CMS1: MSI immune; CMS2: canonical; CMS3: metabolic; CMS4: mesenchymal) [49] with distinct phenotypic and clinical features. CMS1 and CMS3, each of which has a distinctive genomic and epigenomic profile, together represent approximately $25\%$ of cases. CMS2 and CMS4 are the most common subtypes, and although they have similar patterns of somatic mutation, structural variation, and methylation, they differ significantly in outcomes; CMS2 tumors have relatively good survival rates, while CMS4 cases are characterized by more aggressive tumors and poorer prognosis [81]. Additional studies have described intratumor heterogeneity and clinically actionable features that distinguish CMS4 and have found evidence that this more aggressive mesenchymal subtype often arises from CMS2-like tumors [82]. We then reasoned that multiomic association networks and GRNs could provide further insight into the mechanisms that drive the difference between these subtypes.

Using the full pipeline, we specified the TCGA samples that belong to each subtype [49], downloaded and preprocessed the data, and generated multiomic and GRN networks for each subtype. We began by constructing 4 subtype-specific DRAGON partial correlation networks that integrate TF gene expression with promoter methylation. Given $F_M$ transcription factors for which we have methylation data and $F_E$ for which we have expression data, each network has a total of $F_M + F_E$ nodes. The corresponding adjacency matrix is symmetric (as DRAGON networks are undirected) and can be divided into 3 blocks: (i) expression–expression partial correlations $(E_i, E_j)$, (ii) methylation–expression partial correlations $(M_i, E_j)$, and (iii) methylation–methylation partial correlations $(M_i, M_j)$.

**Figure 1:**
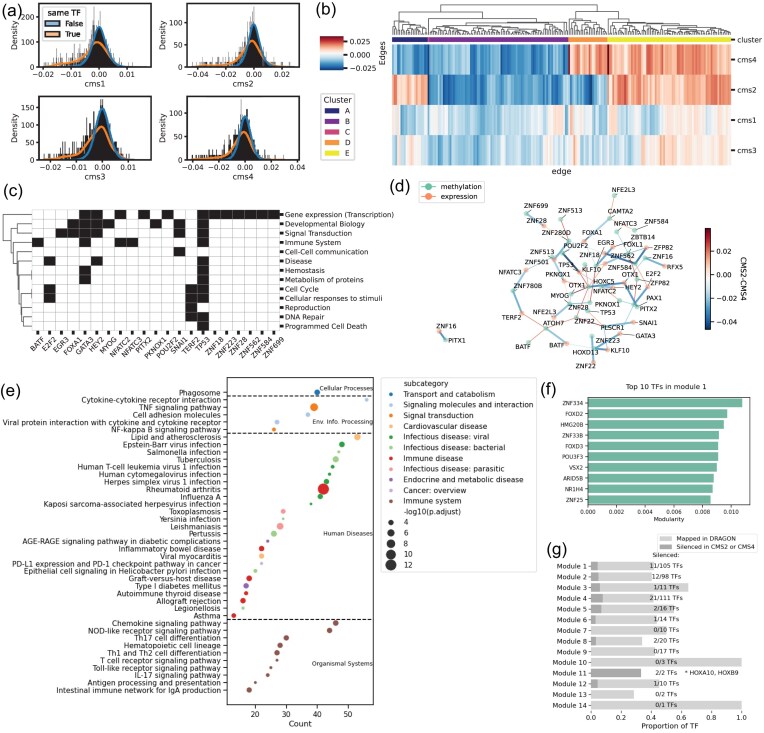
Multiomic and regulatory differences between colon cancer subtypes. (A) Distribution of partial correlation DRAGON values between methylation and expression of TFs in all subtypes. In orange, we show the values for the edges of the same TF, which is the correlation between the methylation of the promoter and the expression of that same TF. As expected, methylation and expression tend to be negatively correlated. The histogram represents the distribution density, normalized per subtype and per group. (B) For intermodality DRAGON edge weights (methylation-to-expression), we remove the edges between the same transcription factor, select the first 200 strongest edges (highest average absolute value of correlation), and cluster them by correlation values for each subtype (average linkage, Euclidean distance). It is interesting that, for clusters A and D, the edge values for CMS2 and CMS4 are swapped in direction. (C) Detailed annotation of all TFs in cluster D (columns) to the Reactome parent term. “Immune system” and “Cellular responses to stimuli” are more consistently involved in cluster D, in comparison to cluster A. Here, we can identify which TFs are annotated to each term. For example, a good number of TFs are involved in the immune system (BATF, GATA3, NFATC2, NFATC3, TP53). As expected, many of the TFs are annotated to generic transcription pathways, and TP53 is annotated to almost all terms. (D) DRAGON association graph for the TFs in cluster D, showing the difference between the partial correlations in CMS2 and CMS4. We have selected the edges that belong to cluster D (thicker edges), and we have added 20 other top edges, by absolute value, that connect the same TFs (thinner edges), such that we have a connected graph. We show the TFs as nodes with different colors for methylation (green) and expression (orange), and we show edge values color-coded by the differential partial correlation values. (E) Pathway analysis (overrepresentation) of module 1 of ALPACA comparing CMS2 and CMS4. We show all significant pathways (adjusted *P* < 0.01). The x-axis represents the overlap between module 1 and each pathway, and the dot size depends on the adjusted *P* value. Dots are organized by category (annotated on the plot) and colored by subcategory. Many of the terms are relative to “Immune system” (brown dots) and signaling pathways (blue and orange dots). Among the “Human Diseases” terms, many of which involve the immune system, we find PD-L1 expression and the PD-1 checkpoint pathway in cancer. (F) Top 10 TFs in module 1 of ALPACA sorted by modularity. (G) Overlap between the ALPACA and DRAGON results. In light gray, we show the share of TFs in each ALPACA module that is mapped in DRAGON (around $50\%$ among most modules). In dark gray is the proportion of the TFs that are considered “silencing” in either subtype CMS2 or CMS4. Module 11 has evidence of silencing in CMS2 for both TFs, HOXA10 and HOXB9, known for their developmental role.

Because promoter methylation is generally inhibitory, we expected it to exhibit an inverse partial correlation with gene expression [83, 84]. Indeed, by examining the distribution of methylation to expression edge weights ($M_i, E_j$), we saw that weights for edges connecting a gene to its promoter methylation ($M_i, E_i$) have a distribution more strongly skewed to negative values than the distribution of the promoter methylation to the other genes ($M_i, E_j$) (Fig. 1A). We also noticed that there is often consistency in these ($M_i, E_i$) edges across different subtypes (Supplementary Fig. S4).

To evaluate the relevance of these edges, we investigated the relationship between cancer phenotype and TFs for which we inferred direct epigenetic regulation based on the DRAGON networks. We first considered the actual edge weights ($M_i, E_i$) of the cancer drivers (according to the COSMIC cancer gene census). We used a Kolmogorov–Smirnov test to check if the cancer-associated TFs have lower edge weights than is the case for TFs that are not annotated as being cancer-associated. We found that colon drivers have lower edge weights in CMS2 (*P* = 0.039), and all drivers have lower edge weights in CMS4 (*P* = 0.088). Then, we selected the TFs for which the ($M_i, E_i$) edge weights are in the first decile of the distribution of at least 1 subtype, and we found 108 TFs with evidence of methylation-driven “silencing” (Supplementary Fig. S5). Some of these TFs (STAT5A, CREB3L1, ZNF24, HMGA1, IRF8, PAX8, CDX2, CREB3L2, TFEB, MGA, NFIB, KLF6, LEF1, HOXD13, HOXA13, HOXB13, GATA2) are known to be cancer drivers [85–88, 85–88]. For example, STAT5A, the only TF that has low ($M_i, E_i$) edges in all subtypes, is a known oncogene involved in the JAK signaling cascade [89, 90], while CREB3L1, LEF1, and PAX8 are all known to be involved in invasion and metastasis [91–94].

We also assessed the overrepresentation of the TFs under putative epigenetic regulation in the Reactome pathway database using a hypergeometric test. While the pathway analysis on TFs is not sufficiently powered because most pathways contain fewer than 10 TFs, we found nominal enrichment not only for broadly defined transcriptional pathways, such as “Generic Transcription Pathway” (odds ratio [OR] = 1.71, *P* = 0.0093) and “RNA Polymerase II Transcription” (OR = 1.64, *P* = 0.014), but also for more specific pathways such as “Formation of paraxial mesoderm” (OR = 31.96, *P* = 0.006), “Gastrulation” (OR = 2.72, *P* = 0.032), “Incretin synthesis, secretion, and inactivation” (OR = 22.63, *P* = 0.033), and “Beta-catenin independent WNT signaling” (OR = 6.37, *P* = 0.045) (Supplementary Fig. S5). These pathways are all related to the epithelial–mesenchymal transition (EMT), a well-established hallmark of cancer that drives invasion and metastasis [95, 96]. Lastly, to further validate these networks, we compared DRAGON edges with the coexpression edges in the StringDB database and found evidence of a correlation with DRAGON expression–expression edges (Supplementary Fig. S6).

We then explored the interomic partial correlation edges $(M_i, E_j)$ that connect methylation associated with 1 TF to the expression of another TF and focused on the edges that differ between the subtypes, under the hypothesis that such edges can identify complex regulatory relationships that distinguish disease states. We selected the edges with the highest absolute value (either the strongest positive or negative partial correlation) and performed hierarchical clustering (using average linkage and Euclidean distance) on these edges. We identified 5 clusters (Fig. 1B), with clusters A and D showing distinct patterns that represent opposite associations between CMS2 and CMS4. Even when compared to the rest of the edges connecting the same TFs (Supplementary Fig. S7), edges in clusters A and D switch direction between these 2 subtypes, suggesting a specific change in the regulatory process.

Using functional enrichment analysis, we found that TFs in clusters A and D are involved in the metabolism of proteins and DNA repair. TFs in cluster D, in contrast to cluster A, include genes preferentially involved in the Immune System (Supplementary Fig. S8). These include BATF, GATA3, NFATC3, NFATC2, and TP53, which we found to be involved in the “Immune System” pathways (including “Signaling by the B Cell Receptor (BCR)” (nominal *P* = 0.023, OR = 13.8) and “Innate Immune System” (nominal *P* = 0.03, OR = 4.71), while E2F, TERF2, and TP53 participate in “Cellular responses to stimuli” pathways, including “DNA Damage/Telomere Stress Induced Senescence” (nominal *P* = 0.014, OR = 19.3) and “Cellular Senescence” (nominal *P* = 0.024, OR = 6.54) (Fig. 1C). We note that a general limitation of this functional enrichment analysis is that most TFs are only annotated to a small subset of pathways related to transcription and not comprehensively mapped to those on which they have a regulatory effect. This structural limitation of the pathway database results in smaller pathway overlap and reduced power to detect enrichment.

This overrepresentation of immune-related TFs is consistent with reports that CMS4-like tumors exhibit a greater degree of immune infiltration than do other subtypes [82]. Further, epigenetic changes in the FOX/HOX and SNAIL TF families are known to be involved in colorectal cancer etiology [97], and TP53, FOXA1, GATA3, and GATA6 are all TFs that, when active in an aberrant form, are recognized as being hallmarks of cancer [87].

While we have described the individual role of these TFs, it is important to remember that these differences between CMS2 and CMS4 can also be contextualized as the subgraph that emerges from cluster D (Fig. 1D). For instance, there is a clearly strong difference between the association of methylation of POU2F2 and expression of TP53 (edge values CMS2: $-0.024$, CMS4: 0.021). Positive correlation between TP53 expression and POU2F2 methylation is consistent with evidence that silencing of TP53 and availability of POU2F2 are leading to oncogene-induced senescence escape and tumor progression in colorectal cancer [98]. If we instead focus on interactions between more than 2 nodes, we can see how PAX1, OTX1, HOXC5, and PKNOX1 are differentially associated between the 2 subtypes. These are all TFs involved in the “Wnt/$\beta$-catenin signaling” pathway, which is well known for its role in cancer development and progression [99] and was also enriched for the epigenetically controlled TFs in our analysis of DRAGON edges. It is finally worth mentioning that the cancer driver TFs are only overrepresented in cluster E, with 19 TFs (CAMTA1, HLF, IRF4, STAT5A, HOXB13, ETV4, GATA2, STAT5B, HOXC13, FOXA1, ATF1, MGA, PRRX1, IKZF3, ERF, HOXA13, DOT1L, MNX1, TP53) annotated in the OncoKB dataset (Fisher’s exact test OR = 1.84, *P* = 0.005) and 15 annotated in the Cancer Gene Census dataset (Fisher’s exact test OR = 1.84, *P* = 0.013). In this module, we do not see differentially associated edges between CMS2 and CMS4, which suggests a shared contribution of methylation to cancer etiology.

Since somatic DNA variation is well studied in the context of cancer and has previously been established as characteristic of the CMS1 subtype, we have focused the above investigation on the associations between promoter methylation and expression, data types that have not yet been ethoroughly explored. However, as an illustrative example of the flexibility of *tcga-data-nf* to include additional omics, we have also generated CNV–expression DRAGON networks that capture associations between copy number changes at the gene level and expression. Analogously to what we described above for the methylation–expression DRAGON networks, we now have 3 types of edges: CNV–CNV ($C_i, C_j$), CNV–expression ($C_i, E_j$), and expression–expression ($E_i, E_j$). By exploring these edge distributions, we demonstrate that CNV–expression DRAGON networks can also detect key effects of DNA aberrations, as follows.

As we did for the methylation–expression DRAGON networks, we first confirmed the validity of these CNV–expression networks. CNV usually involves deletions or duplications of DNA segments. For this reason, genes that are near each other on the genome are expected to have similar CNV values. This effect was also visible in the adjacency matrices, where there is an evident block structure in the CNV–CNV edges (Supplementary Fig. S9A). Moreover, we have evidence that, even in this case, DRAGON networks can capture direct effects of CNV onto expression; the CNV–expression edges on the same TFs ($C_i, E_i$) tend to be skewed toward positive values (Supplementary Fig. S9B), which is expected behavior for somatic variation. Within the top 20 TFs that showed high correlation between their own CNV and expression, we observed known colon cancer drivers [87] ZNF703 and SMAD4, as well as IRF2 and GATA6, whose DNA aberrations are likely oncogenic [83] (Supplementary Fig. S9B).

Finally, we explored the greatest differences between CMS2 and CMS4 subtypes in terms of partial correlations between CNV and expression. For each CNV node $C_i$, we compared the edge set $\lbrace (C_i, E_j)\rbrace$ between the 2 subtypes. We conducted a paired Wilcoxon signed-rank test for each TF *i*, testing the null hypothesis that there is no difference in CNV–expression edge weights between CMS2 and CMS4 (Supplementary Fig. S9C). Among the top TFs with varying edges between CMS2 and CMS4 based on the Wilcoxon test results were ZNF554, for which we have partial evidence of involvement in cancer progression through the WNT/$\beta$-catenin signaling pathway [100]; KLF16, which plays a role in the stress-related programming of colorectal cancer [101]; and DOT1L, a methyltransferase that regulates core stem cell genes and affects tumorigenesis and drug resistance [102]. These TFs are significantly associated with colon cancer drivers. When comparing the Wilcoxon test statistics of the cancer TFs to the rest of the TFs, we get a significant Kolmogorov–Smirnov test (stat $=0.21$, *P* = 0.013). Also, there are 27 significant (false discovery rate [FDR] $< 0.01$) TFs that are annotated by either the Cancer Gene Census or the DisGeNET dataset as colon cancer drivers (Fisher’s exact test OR = 1.6, *P* = 0.057, Supplementary Fig. S9D). Among the cancer drivers that show the biggest association changes between CMS2 and CMS4, we found HIF1A and CUX1, both of which are implicated in tumorigenesis and progression [88], and GATA4, which controls senescence [103, 104].

### Regulatory differences between colon cancer subtype GRNs

The DRAGON networks that we inferred capture information about patterns of DNA methylation and how these patterns are associated with transcription factor expression. The working hypothesis behind this analysis was that some transcription factors exhibit altered patterns of methylation that affect gene expression and ultimately exert downstream effects that help to define different cancer subtypes. Indeed, in the DRAGON networks constructed using transcription factors, we identified several edges that differ between the CMS2 and CMS4 subtypes, providing a plausible explanation for the differences they present in the clinical context. However, the partial correlations represented by edges in DRAGON networks are limited in that they are measures of association and do not capture the actual regulatory effects that TFs have on their target genes.

PANDA is a GRN inference method that uses prior knowledge on motif binding and TF–TF physical interactions together with assayed gene expression to generate a bipartite graph associating TFs with the genes they likely regulate. Each TF–gene edge is weighted by a measure of the evidence of a regulatory relationship. We generated PANDA GRN networks using the TCGA–COAD data, enabling us to perform an analysis aimed at identifying the regulatory context for the TFs identified in clusters A and D, for which we have evidence of changes in methylation and expression patterns between CMS2 and CMS4. After identifying the TFs using the DRAGON analysis, we proceeded to investigate the functional role of their targets.

We compared the 2 PANDA networks and selected the edges with the greatest differences in edge weights between CMS2 and CMS4 to identify patterns of differential regulation attributable to the selected TFs from the DRAGON analysis. A gene set enrichment analysis on these differentially targeted genes reveals that they are involved in the transcriptional misregulation of cancer and various immune-related Reactome pathways that include “Creation of C4 and C2 activators,” “Initial triggering of complement,” and “Signaling by the B Cell Receptor (BCR)” (Supplementary Tables S1 and S2). Moreover, TFs in cluster D exhibit consistent differential targeting of genes in the “TGF-$\beta$ signaling,” “Cytokine–cytokine receptor interaction,” and “Antigen processing and presentation” pathways (Supplementary Fig. S10 and Supplementary Tables S3 and S4). These pathways are consistent with prior evidence that TGF-$\beta$ is not only associated with poorer prognosis in colon cancer but also involved in the transition from CMS2 to CMS4 [81].

Finally, we extended our search beyond the TFs identified by DRAGON and performed a genome-wide comparison between the 2 PANDA networks with ALPACA. ALPACA identifies submodules that optimize the differential modularity of 2 networks, finding 14 differential modules between CMS2 and CMS4. First, we checked the overall differences in network connectivity by looking at the differential in-module and out-module degrees of genes and TFs (details in the Methods section). Modules 1, 2, and 4 are those exhibiting the most significant difference in connectivity between CMS2 and CMS4 (Supplementary Tables S5 and S6).

ALPACA uniquely assigns TFs and genes in the network to modules and also provides a ranking of nodes based on their contribution to modularity. We can thus use ALPACA results to investigate the functional role of each module. For example, we find that module 1 is enriched for many immune-related pathways, including “Immune System,” “Immune Disease,” and “Infectious Disease,” as well as cytokine-related signaling pathways, “TNF signaling pathway,” and “NF-$\kappa$B” (Fig. 1E). Of the TFs that have the highest contribution to modularity for module 1, most have been validated as key regulators of colorectal cancer (Fig. 1F). Indeed, 6 out of 10 leading TFs have direct evidence and some experimental validation of their involvement in colon cancer risk and progression: ZNF334 [105], FOXD2 [106], FOXD3 [107], POU3F3 [108], NR1H4 [109], and VSX2 [110].

We performed a similar analysis on all the other modules. Module 2 appears to be more cancer-specific as it is consistently enriched for cell cycle pathways (“Cell cycle,” “Cellular senescence”), known cancer-related signaling pathways (“mTOR” and “JAK-STAT” signaling pathways), metabolic pathways, and cancer-specific disease terms (Supplementary Fig. S11). Module 4 is strongly enriched for signaling pathways, including “Wnt signaling,” and development and regeneration pathways (Supplementary Fig. S11). While we know that zinc finger proteins (ZNFs) are still not well characterized, hindering the functional annotation of module 4 where 9 out of 10 top TF are ZNFs, we found that ZNF341, which is the fourth highest-ranking hit for modularity contribution (Supplementary Fig. S12), is a regulator of STAT3 and plays a key role in hyper-IgE syndrome (HIES), a condition that often manifests as severe and chronic bacterial infections [111]. In parallel, we found reports of associations between IgE-mediated immune reactions and colorectal cancer risk [112]. Although we do not have definitive evidence or validation of the role of ZNF341 in the colon cancer subtypes, we believe that this suggestive result demonstrates how multiomic network analysis can find complex regulatory patterns. Taken together, the analysis of modules 1, 2, and 4 shows that CMS2 and CMS4 at the regulatory level exhibit differences in the immunological response, cellular processes such as growth and senescence, and signaling and developmental pathways.

Finally, we examined the relationship between the results of the multiomic DRAGON analysis (methylation–expression) and ALPACA. It is worth noting that DRAGON, as used here, estimates partial correlations between the methylation and expression of TFs, while ALPACA infers differential modularity between GRNs. Methylation is only one of many possible modifications that can change gene expression, translation, and regulation (mutations, CNVs, chromatin accessibility, etc.). Therefore, we do not necessarily expect DRAGON and ALPACA (used with PANDA-based GRNs) to be concordant. However, using the DRAGON results, we explored whether promoter methylation can explain the differential modularity of these colorectal cancer GRNs.

To interpret these results, we first note that DRAGON uses a different, more stringent filtering method than PANDA. Consequently, only about $50\%$ of TFs in the ALPACA modules can be found in the DRAGON networks (Fig. 1G). Nevertheless, we investigated the representation of these overlapping TFs in the ALPACA modules that might indicate that epigenetic changes drive the regulatory differences in a specific module. Specifically, we looked for TFs in ALPACA modules for which we have evidence of methylation-driven “silencing” in either CMS2 or CMS4, which are those with a strong negative association between expression and methylation of the same TF in DRAGON (Fig. 1G, Supplementary Fig. S13). While most “silenced” TFs are distributed across the ALPACA modules, in module 11, we found both the TFs, HOXA10 and HOXB9, “silenced” in CMS2 and not CMS4. This result suggests that these TFs may play an important role in the overall differential regulation of gene expression in the 2 subtypes. It is also consistent with reports that HOX TFs are differentially accessible in colorectal cancer and that they are involved in cancer development and progression [97].

## Testing

Apart from the main workflows for TCGA data analysis, showcased in the previous section, our framework also offers a testing option that assesses the general availability and formatting of data and software and runs a trial analysis on a sample dataset.

Nextflow profiles are sets of configuration parameters accessible with the “–profile” scope. For testing purposes, we provide testing configuration profiles for all workflows, with the following names: “test” for the full pipeline and “testDownload,” “testPrepare,” and “testAnalyze” for the specific workflows.

### test

This profile runs the full *tcga-data-nf* pipeline on a subset of TCGA PAAD data. All parameters are specified in the “conf/test.config.”

### testDownload

To test the download step, we need to confirm that the TCGA data are available and retrievable, and therefore, we cannot use minimal dummy datasets. To avoid downloading files that are too large, during testing, we only retrieve data from TCGA PAAD, one of the smallest datasets. All data are downloaded into the “results/download_test/” folder.

### testPrepare

To test the prepare step, dummy expression and methylation datasets are provided that contain randomized sample labels and values. Both datasets have only 10 samples each; consequently, this step computes quickly. Each of the recount pipeline parameters is tested for only 1 value, such that only 1 input configuration is tested and only 1 output is produced.

### testAnalyze

To test the analyze step, we provide dummy preprocessed data for expression and methylation data in the testdata/analyze_expression.csv and testdata/analyze_methylation.csv files. Alongside the input data, we also provide dummy TF–target binding and dummy protein–protein interaction (PPI) data, which are necessary to infer PANDA networks. The test computes all network methods (PANDA, DRAGON, LIONESS, and ALPACA) in order to confirm that the whole implementation is functioning properly and the netZooPy and netZooR packages are correctly installed and running.

## Computational Performance

Nextflow allows users to track computational requirements and performance of all processes. While wall and central processing unit (CPU) time heavily depend on the data type and size, estimating CPU and memory usage on sample datasets can help guide users in their choices. First, we report the performance of the pipeline on test data where the analyses were run on an AWS EC2 c5.4xlarge instance with 32 Gb of memory and 16 vCPUs. We ran all tests with the v0.0.18 version of *tcga-data-nf* with standard configuration files that allow Nextflow to run all jobs serially, with no job parallelization, where each process is allowed to use all computing resources available. For reference, this would be consistent with testing the workflow on a local machine. The testDownload workflow took 4 minutes, 2 seconds (1.1 CPU hours) and has a peak memory usage of 4.1 Gb for the download CNV process. The testPrepare run took 1 minute, 17 seconds (0.3 CPU hours) and has a peak of 3.5 Gb for GetGeneLevelPromoterMethylation. The testAnalyze run only took 59 seconds (0.2 CPU hours) and has a peak of 3.1 Gb of memory for the runTCGAAlpaca process. We recommend using these tests first to ensure smooth operation of the workflow on dummy data. The test full workflow, instead, requires 1 hour, 48 minutes of wall time (28 CPU hours) and a peak memory of 22 Gb for runTCGAPanda. This is a realistic run of the workflow on the actual TCGA–LUAD data and includes both ALPACA and LIONESS runs, which are very time-consuming. The *tcga-data-nf* documentation includes the report for this test workflow [113], with a detailed description of the CPU and memory performance for each of the processes.

Second, running LIONESS with PANDA is undoubtedly the most expensive process, as it reconstructs 1 leave-one-out PANDA network for each sample in the population and requires double the memory [114]. However, netZooPy supports GPU-based computation of PANDA–LIONESS networks [115]. On an Nvidia H100 GPU with 32 GB of memory allocated, running PANDA–LIONESS for TCGA–PAAD (183 samples) took 62 minutes, meaning that computing each PANDA–LIONESS network takes approximately 19 seconds.

## Discussion

There is a growing recognition that inferring and analyzing gene regulatory network models can provide unique and verifiable insights into the drivers of disease, particularly in rich public databases such as TCGA [34]. Indeed, the GRAND database already provides access to more than 20,000 genome-wide gene regulatory network models, built on publicly available datasets (including TCGA) and used to generate insights in numerous published studies over the years [50]. However, as methods and data evolve, we anticipate that others will be interested in generating and investigating novel networks using TCGA data. Because setting up the requisite environments and data structures to estimate such network models can be challenging, even for those with experience in bioinformatics and computational biology, in this article, we bridge the gap between the wealth of data in TCGA and the skills and computational resources needed to analyze it by providing a user-friendly, standalone workflow.

We developed an end-to-end reproducible workflow capable of downloading, preprocessing, and generating regulatory networks from TCGA cancer data with a single command. This workflow is publicly available and uses fully open-source software, adhering to what has been deemed the “gold standard” for reproducibility [3]. The workflow allows the preprocessing of multiomic data and inference of regulatory networks without requiring users to write code *de novo*; instead, the user needs only to specify a relatively small number of parameters. In addition to the workflow itself, we provide several supporting resources, including Docker containers and conda environments, extensive configuration files, documentation describing how to reuse the workflow, and pregenerated networks for the 10 most common cancer types in TCGA.

As a demonstration of the value and flexibility of the *tcga-data-nf* pipeline, we applied the workflow to TCGA–COAD data. We used *tcga-data-nf* to generate multiomic association networks (DRAGON) and GRNs (PANDA) for the 4 consensus molecular subtypes. This is an excellent use case for the work presented here; the ability of the *tcga-data-nf* pipeline to reproducibly and repeatedly run the same workflow with different inputs made it straightforward to analyze different COAD subtypes with multiple network estimation methods. This analysis uncovered evidence of previously undescribed methylation–gene expression interactions that suggest epigenetic regulation of TGF-$\beta$ signaling and may help to explain factors influencing the transition between subtypes CMS2 and CMS4. To fully showcase the use of *tcga-data-nf*, we also computed DRAGON networks for CNV and expression. We used ALPACA to analyze the differences between CMS2 and CMS4 PANDA networks, providing further evidence for relevant epigenetic silencing of critical TFs in the CMS2–CMS4 transition. The code for this example is available on GitHub [116], and the data have been archived on the Harvard Dataverse [117], ensuring complete reproducibility of the analysis.

The biggest challenge we faced in designing *tcga-data-nf* was balancing the trade-off between flexibility and completeness. We reasoned that individual pieces of software, such as GDC, TCGABiolinks, edgeR, and netZooPy, already provide a broad set of functions covering all the steps required for network generation and analysis. As such, the workflow was designed to chain these tools seamlessly together, allowing users to carry out complex analyses simply by specifying a small number of parameters. While flexible by design, the release version of *tcga-data-nf* does not provide users with unlimited options. For example, we provide only 2 commonly used RNA-seq normalization methods; the full workflow can only be applied to single tumor types and cannot generate pan-cancer analyses without *post hoc* coding, and the workflow does not cover all possible data types available from TCGA, such as microRNA expression. However, the flexibility of *tcga-data-nf*’s design should allow users to overcome these limitations; we provide individual Download, Prepare, and Analyze steps that can be used separately from the rest of the pipeline and run with custom input data. We have also provided examples of how the pipeline can be modified and expanded for different analyses—for example, downloading additional data modalities or running other GRN inference or analysis methods. Finally, we recognize that a limitation of *tcga-data-nf* is that it was designed to generate GRNs from bulk sequencing data from TCGA, and most of the intermediate steps available are specific to the analysis of such data. While one could, in principle, use *tcga-data-nf* to download single-cell RNA-seq data from GDC from the CPTAC3 project, or extend the Analyzesteps to include SCORPION [118] to infer GRNs from single-cell transcriptomics data, creating a dedicated workflow would handle the single-cell data more efficiently.

Overall, *tcga-data-nf* addresses a clear need for reproducible, easy-to-use, and efficient network analysis workflows for large-scale cancer data. In this article, we demonstrate how this pipeline can be utilized to generate new insights into multiomic data from TCGA, and we provide comprehensive documentation and supplemental resources to aid researchers in navigating precomputed networks or generating new ones. We have also illustrated the modularity of the pipeline and its extensibility to other tools within and beyond the Network Zoo. Not only do we expect *tcga-data-nf* to grow as we develop novel network analysis methods, but we also envision *tcga-data-nf* contributing broadly to future biomedical research, serving as the foundation for other GRN analysis workflows.

## Methods

### Pathway analysis

In the section “Multiomic partial correlation networks identify differences between colon subtypes,” we carried out all pathway analysis using the GSEApy package [119]. We tested for overrepresentation (ORA) of TFs and genes in both the Reactome and KEGG sets of pathways with a hypergeometric test. For both cases, we selected the appropriate background, that is, all TFs in the DRAGON networks or the gene targets in the PANDA networks. The KEGG dataset was downloaded from the GSEApy package as the “KEGG2021” dataset. For all tests, we applied the Benjamini–Hochberg procedure to control the FDR during multiple testing [120]. We downloaded the pathway files from the Reactome database on 18 June 2024. We downloaded the tables that map each gene identifier to a pathway—and that also map each pathway to its parent terms. For instance, “Intracellular signaling by second messengers,” “Signaling by GPCR,” “Signaling by Hedgehog,” and so on are all part of the “Signaling Transduction” term. To reduce the number of tested pathways, which also reduces overlaps between pathways, we have generated a “slim” set of pathways. For each leaf in the Reactome dataset, we have retained only the “parent” node. In this way, we avoid keeping all the nodes that are too small and keep only the depth-1 term.

In the section “Regulatory differences between colon cancer subtype GRNs,” we used R’s clusterProfiler package [121] on ALPACA results to run the KEGG pathway analysis. Test *P* values were corrected with the Benjamini–Hochberg procedure, and we considered as significant those with FDR $<0.05$.

All code and data used for the pathway analysis are in the “tcga-data-supplement” repository [116].

### Reference data

PANDA uses “prior knowledge” on putative TF–motif binding and a TF–TF “cooperativity prior” based on TF PPI data, together with correlations between genes computed from the expression profiles of all samples. To create a regulatory motif network, we downloaded TF motifs for *Homo sapiens* with direct or inferred evidence from the Catalog of Inferred Sequence Binding Preferences (CIS-BP) Build 2.0, accessible at http://cisbp.ccbr.utoronto.ca. These TF position weight matrices (PWMs) were mapped to the human genome (hg38) using FIMO [122]. We retained only highly significant matches ($p \le$  $10^{-5}$) occurring within the promoter regions of Ensembl genes (specifically, GENCODE v39 annotations retrieved from http://genome.ucsc.edu/cgi-bin/hgTables). These promoter regions were defined as the interval of [−750; +250] base pairs centered on the transcription start site (TSS). This process yielded an initial set of potential regulatory interactions involving 997 TFs that collectively targeted 61,485 genes.

For the TF–TF cooperativity prior, we obtained PPI data from the StringDB database (version 11.5) using the STRINGdb Bioconductor package [123]. Subsequently, we filtered the PPI data to retain only interactions between transcription factors in the TF–motif network (using a score threshold index of 0). To maintain consistency in PPI scores, we normalized them by dividing each score by 1,000, thereby restricting the values to a range of 0 to 1 for both the PPI dataset and the TF–motif network. Additionally, we set self-interactions between TFs to a value of 1. Since PPI networks are inherently undirected, we transformed the data into a symmetric PPI matrix.

### Cancer datasets

We annotated the TFs to cancer-specific gene collections from a variety of resources. We downloaded the OncoKB [85, 124] gene list on 13 December 2022. We downloaded the Cancer Gene Census v101 [88, 125] on 15 April 2025; for the colon-only genes, we selected those that have the terms “colon” or “colorectal” as Tumor Types (Somatic). Finally, we downloaded the DisGeNet v25.1.1 [86, 126] curated gene–disease associations for the term C0009404 (Colorectal Neoplasms) on 15 April 2025.

### Differential degree testing

Given 2 PANDA networks for subtypes CMS2 and CMS4, with weighted edges $e_{u,v}^{(CMS2)}$ and $e_{u,v}^{(CMS4)}$ connecting TF *u* to gene *v*, ALPACA partitions the set of *P* TFs and *R* genes into *C* modules that maximize the differential modularity between CMS2 and CMS4. For each gene *r* in module *c*, we defined the *in-module differential degree* as


\begin{eqnarray*}
\mathrm{diff}_{r}(c) = \sum _{p \in \mathcal {P}}(e^{CMS2}_{pr} - e^{CMS4}_{pr})
\end{eqnarray*}

This is the sum of all differences between CMS2 and CMS4 edges connecting the gene to all the TFs *P* that belong to the same module.

For comparison, for each gene *r* in module *c*, we also defined the *out-module differential degree*, accounting for all edges from gene *r* directed to nodes that do not belong to module *c*:


\begin{eqnarray*}
\mathrm{diff}_{r}(\lnot c) = \sum _{i \notin \mathcal {P}}(e^{CMS2}_{ir} - e^{CMS4}_{ir})
\end{eqnarray*}

For each module, we ran a Wilcoxon signed-rank test between the in-module and out-module gene differential degrees (Supplementary Table S5). We then ran the same analysis with each TF (Supplementary Table S6), where we obtained the degrees by summing up the edge values connecting each TF *p* to the *R* genes.

## Availability of Source Code and Requirements

### tcga-data-nf

- Project name: *tcga-data-nf*
- Project homepage: e.g., https://github.com/QuackenbushLab/tcga-data-nf
- Operating system(s): e.g., Platform independent
- Docker: https://hub.docker.com/r/violafanfani/tcga-data-nf
- Programming language: Nextflow, R, Python, bash
- Other requirements: Java, Nextflow
- License: GNU General Public License v3.0
- WorkflowHub SEEK ID: https://workflowhub.eu/workflows/1306?version=1

### NetworkDataCompanion

- Project name: *NetworkDataCompanion, NDC*
- Project homepage: https://github.com/QuackenbushLab/NetworkDataCompanion
- Operating system(s): MacOS, Linux
- Programming language: R
- License: GNU General Public License v3.0
- SciCrunch registry: RRID:SCR_026532
- bio.tools registry: https://bio.tools/NetworkDataCompanion

### Notebooks and configuration files

We provide a GitHub repository that contains (i) all configuration files mentioned in this article and (ii) notebooks and supplementary data for the analysis of colon cancer subtypes.

- Project name: tcga-data-supplement
- Project home page: e.g., https://github.com/QuackenbushLab/tcga-data-supplement
- Operating system(s): Linux, MacOS, Windows
- Programming language: Python
- License: MIT

## Additional Files

**Supplementary Fig. S1**. Download. Directed acyclic graph of the processes specified in the Download pipeline. For each modality that is specified in the configuration file, *tcga-data-nf* downloads the data and generates metadata tables with the names, paths, and parameters of the files. Whenever *tcga-data-nf* is run, we also generate and save the configuration parameters, which can then be examined and reused (saveConfig process).

**Supplementary Fig. S2**. Prepare. Directed acyclic graph of the processes specified in the Prepare pipeline. The expression and methylation data specified in the configuration metadata are processed using the combination of all input parameters (tissues, purity, minTPM, etc.). Whenever *tcga-data-nf* is run, we also generate and save the configuration parameters, which can then be examined and reused (saveConfig process).

**Supplementary Fig. S3**. Analyze. Directed acyclic graph of the processes specified in the Analyze pipeline. Using the input metadata, the *tcga-data-nf* workflow generates PANDA, DRAGON, LIONESS, GENIE3, and WGCNA networks and matches them with log files and intermediate tables, useful for further investigation of the results. PANDA networks are compared with ALPACA. Whenever *tcga-data-nf* is run, we also generate and save the configuration parameters, which can then be examined and reused (saveConfig process).

**Supplementary Fig. S4**. Correlation between DRAGON methylation–expression edges on the same TFs. For all $(M_i, E_i)$ edges, we plot their distribution in each subtype (histograms on the diagonal) and the correlation of the edge weights between each pair of subtypes. While all Pearson correlation values are above 0.50, it is worth noting that CMS2 and CMS4 are the most similar to each other with $\rho = 0.77$. This indicates that many of the TFs with negative partial correlations between methylation and expression are conserved across the CMS2 and CMS4 subtypes, whereas they are more distinct for CMS1.

**Supplementary Fig. S5**. TFs with evidence of epigenetic effect on expression. From all $(M_i, E_i)$ edges, for each subtype, we select those whose values are in the first decile of the distribution, that is, the smallest $10\%$ of edges. Here we plot the edge value on the left and the annotation to one of each TF to a cancer-related database. In particular, OncoKB (downloaded 13 December 2022), Cancer Gene Census (v101, downloaded 25 April 2025), and DisGeNet (GDA CURATED C0009404, downloaded 25 April 2025).

**Supplementary Fig. S6**. Relationship between DRAGON edges and StringDB coexpression evidence. For each colon cancer subtype (rows), we plot the values of the DRAGON edges on the x-axis, grouped by edge type (columns), and the confidence score in the StringDB database on the y-axis. For all subtypes, there is evidence of a correlation between the DRAGON and StringDB coexpression, confirming the validity of our inferred associations; methylation–methylation and methylation–expression edges serve as negative controls.

**Supplementary Fig. S7**. Edge weights for nodes in clusters A and D of the DRAGON networks. We select first the subgraphs with all the nodes in each cluster. It is worth noting that these graphs contain both the edges represented in Fig. 1B and those that connect the nodes in the cluster but were not the strongest edges. We compare the values of the edges of interest (orange), which are shown in Fig. 1B, and the rest of the edges (blue) that connect the same TFs, as controls. We observe that for both groups, the average edge value is around 0, meaning that there is no general difference between the edge values of the 2 subtypes. However, the edges selected in the clusters switch between higher and lower values for CMS2 and CMS4.

**Supplementary Fig. S8**. Reactome pathways for clusters A and D. Using the clusters found from the DRAGON edges, we run a pathway overrepresentation analysis of the TFs in both clusters A and D. With REACTOME, we can identify the general pathway to which each term belongs. For each “parent” pathway (y-axis), we plot the $-log_{10}(pvalue)$ (x-axis) of all the pathways tested that belong to that parent term, and we color them by the corresponding odds ratio. The red dashed line corresponds to a *P* value of 0.05, which is the line for nominal significance. Since pathway analysis on TFs is challenging (there are only approximately 1,000 TFs, and many of them are annotated only to the general transcriptional pathway terms), we report here all results, even those that are not significant, such that one can observe the general trend. TFs in cluster D seem to be more consistently annotated to Immune System pathways, while the TFs in cluster A have some stronger terms related to Developmental Biology.

**Supplementary Fig. S9**. DRAGON CNV–expression networks. (A) DRAGON CNV–CNV edges for genes in chromosomes 1 and 4. For both CMS2 and CMS4, there is evidence of a block structure that depends on the genome location. Adjacent genes are more likely to be correlated. (B) Distribution of partial correlation values between CNV and expression of TFs in both subtypes. In orange, we show the values for the edges of the same TF ($(C_i,E_i)$), while in blue, we show all the others ($(C_i,E_j)$). As expected, CNV and expression tend to be positively correlated. The histogram represents the distribution density, and it is normalized per subtype and per group. We have also annotated the 20 TFs that have the greatest mean value in both subtypes that are also known for their role in cancer. (C) TFs with CNV–expression edges that are significantly different between CMS2 and CMS4. We show the 20 with the lowest adjusted *P* value (Benjamini–Hochberg FDR) from the paired Wilcoxon signed-rank test. (D) Colon cancer–related TFs with CNV–expression edges that are significantly different between CMS2 and CMS4. We retrieved the colon cancer drivers from both the DisGeNET and the Cancer Gene Census datasets.

**Supplementary Fig. S10**. PANDA edges involved in the main pathways targeted by cluster D. We selected the regulatory edges of the TFs in cluster D (defined by the analysis on DRAGON networks), and we investigated which edges in the PANDA networks underwent the biggest changes between CMS2 and CMS4. The target genes of the edges were found to be preferentially involved in the TGFβ signaling pathway, transcriptional misregulation in cancer, cytokine–cytokine receptor interaction, and antigen processing and presentation. Here, we represent the PANDA edges (edge weight represented by different colors) connecting the main targets for each pathway, for both CMS2 and CMS4 (rows).

**Supplementary Fig. S11**. Pathway analysis of ALPACA’s modules. For each module, we ran the pathway analysis between the nodes in each module and the KEGG pathways, using R’s clusterProfiler package. We grouped the results based on the KEGG subcategory and counted the significant terms (FDR-adjusted *P* < 0.05). We removed clusters with no significant pathway overrepresentations.

**Supplementary Fig. S12**. ALPACA’s top TFs. For each module, we selected the TFs with the highest modularity up to a total of 10 TFs (for modules larger than size 10), that is, those that contribute the most to the differential modularity between CMS2 and CMS4.

**Supplementary Fig. S13**. Overlaps between DRAGON and ALPACA results. For each “silenced” TF that we get from the DRAGON analysis, we plot the partial correlation values between promoter methylation and expression (x-axis) in CMS2 and CMS4 (red and blue bars). The name of the TF is colored based on whether it is significantly silenced in CMS2 (red), CMS4 (blue), or both (purple). The TFs are organized by the ALPACA module they belong to, and we have skipped those that do not have any DRAGON TFs. Of particular interest is module 11, where both HOX TFs have evidence of promoter methylation silencing in subtype CMS2 and not in CMS4.

**Supplementary Table S1**. REACTOME pathway enrichment for the targets of TFs in cluster D. For the targets of the TFs in cluster D, we ran a pathway overrepresentation analysis with the REACTOME pathway database. Here we show the pathways with *P* < 0.05.

**Supplementary Table S2**. REACTOME pathway enrichment for the targets of TFs in cluster A. For the targets of the TFs in cluster A, we performed a pathway overrepresentation analysis using the REACTOME pathway database. Here we show the pathways with *P* < 0.05.

**Supplementary Table S3**. KEGG pathway enrichment for the targets of TFs in cluster D. For the targets of the TFs in cluster D, we ran a pathway overrepresentation analysis with the KEGG pathway database. Here we show the pathways with *P* < 0.05.

**Supplementary Table S4**. KEGG pathway enrichment for the targets of TFs in cluster A. For the targets of the TFs in cluster A, we performed a pathway overrepresentation analysis using the KEGG pathway database. Here we show the pathways with *P* < 0.05.

**Supplementary Table S5**. Comparison of edge differences (CMS2–CMS4) for each gene’s IN-module versus OUT-module degree. For each module, we apply a pairwise Wilcoxon signed-rank test, for which we report the statistic and *P* value, and we compute the Bonferroni FWER. We also report the average IN-module and OUT-module degrees.

**Supplementary Table S6**. Comparison of edge differences (CMS2–CMS4) for each TF’s IN-module versus OUT-module degree. For each module, we apply a pairwise Wilcoxon signed-rank test of which we report the statistic and *P* value, and we compute the Bonferroni FWER. We also report the average IN-module and OUT-module degrees.

## Abbreviations

API: application programming interface; BRCA: breast invasive carcinoma; CIS-BP: Catalog of Inferred Sequence Binding Preferences; CNV: copy number variation; COAD: colon adenocarcinoma; CPM: counts per million; CPTAC: Clinical Proteomic Tumor Atlas Consortium; CPU: central processing unit; EMT: epithelial–mesenchymal transition; FDR: false discovery rate; GDC: Genomic Data Commons; GRAND: Gene Regulatory Network Database; GRN: gene regulatory network; HDF: hierarchical data format; HIES: hyper-IgE syndrome; KEGG: Kyoto Encyclopedia of Genes and Genomes; *NDC: NetworkDataCompanion*; OR: odds ratio; PAAD: pancreatic adenocarcinoma; PPI: protein–protein interaction; PWM: position weight matrix; RNA-seq: RNA sequencing; TCGA: The Cancer Genome Atlas; TCPA: The Cancer Protein Atlas; TF: transcription factor; TMM: Trimmed Mean of M-values; TPM: transcripts per million; TSS: transcription start site; ZNF: zinc finger protein.

## Supplementary Material

giaf126_Supplemental_File

giaf126_Authors_Response_To_Reviewer_Comments_Original_Submission

giaf126_Authors_Response_To_Reviewer_Comments_Revision_1

giaf126_GIGA-D-24-00535_Original_Submission

giaf126_GIGA-D-24-00535_Revision_1

giaf126_GIGA-D-24-00535_Revision_2

giaf126_Reviewer_1_Report_Original_Submission Xi Chen -- 1/26/2025

giaf126_Reviewer_1_Report_Revision_1 Xi Chen -- 5/16/2025

giaf126_Reviewer_2_Report_Original_Submission Jérôme Salignon -- 2/12/2025

giaf126_Reviewer_2_Report_Revision_1 Jérôme Salignon -- 5/13/2025

giaf126_Reviewer_2_Report_Revision_2 Jérôme Salignon -- 9/12/2025

## Data Availability

We precomputed networks for 10 common solid tumors: breast invasive carcinoma (BRCA), lung adenocarcinoma and lung squamous cell carcinoma (LUAD, LUSC), kidney renal clear cell carcinoma (KIRC), liver hepatocellular carcinoma (LIHC), pancreatic adenocarcinoma (PAAD), skin cutaneous melanoma (SKCM), stomach adenocarcinoma (STAD), colon adenocarcinoma (COAD), and prostate adenocarcinoma (PRAD). Raw, multimodal data, and processed data are available through AWS {awsdata}, and a guide to the configuration files and data structure can be found in the supplemental repository [116]. PANDA and PANDA-LIONESS networks are available on GRAND (v1.7) [50, 127]. Replication data for the “Multi-omic partial correlation networks identify differences between colon subtypes” subsection are stored on the Harvard Dataverse [117].
