# Supplementary material for: Reproducible processing of TCGA regulatory networks
Source: Gigascience. 2025 Oct 20;14:giaf126. doi: 10.1093/gigascience/giaf126 (PMC12720619; doi:10.1093/gigascience/giaf126)
Supplement: giaf126_Supplemental_File [file giaf126_supplemental_file.pdf]

GigaScience, 2023, 1–S22

doi: xx.xxxx/xxxx
Manuscript in Preparation
Supplementary material

SUPPLEMENTARY MATERIAL

Reproducible processing of TCGA regulatory networks

Viola Fanfani[1], Katherine H. Shutta[1,2], Panagiotis Mandros[1], Jonas Fischer[1], Enakshi Saha[1], Soel Micheletti[1], Chen Chen[1], Marouen Ben Guebila[1], Camila M. Lopes-Ramos[1,2,3] and John Quackenbush[1,2]

[1]Department of Biostatistics, Harvard T.H. Chan School of Public Health, Boston, MA, USA and [2]Channing Division of Network Medicine, Brigham and Women's Hospital, Boston, MA, USA and [3]Department of Medicine, Harvard Medical School, Boston, MA, USA

[*]Corresponding Author, johnq@hsph.harvard.edu

Compiled on: September 28, 2025.
Draft manuscript prepared by the author.

Supplementary Material

Quickstart

Here is a brief guide on how to initiate the workflow and where to locate all the necessary resources. However, we always recommend reading and following the updated documentation that is at https://github.com/QuackenbushLab/tcga-data-nf.git

Relevant resources and links

- **Supplementary code**: We maintain all companion data and configuration files for the paper in the `tcga-data-supplement` repository https://github.com/QuackenbushLab/tcga-data-supplement. This repository contains all the code for the colon cancer analysis, as well as links and instructions for the precomputed GRNs of common cancer types.
- **Supplementary data**: all data used and generated for this paper is hosted on the Harvard Dataverse https://doi.org/10.7910/DVN/MCSSYJ
- **Docker**:The docker container is hosted on docker.io at https://hub.docker.com/r/violafanfani/tcga-data-nf
- **Nextflow**: Nextflow.io hosts extensive documentation on the language and the best practices to build a workflow https://nextflow.io/docs/latest/index.html

Getting started

Here are the main steps required to run the workflow. Please refer to the official and updated documentation for a more comprehensive explanation.

1. Install Nextflow on your machine; you can follow the instructions on their website[1].
2. Pull the workflow: `nextflow pull QuackenbushLab/tcga-data-nf`
3. Install and pull the docker/singularity container or conda to run the whole pipeline. Details can be found in the documentation[2]
4. Run some test workflows:
   - test the download: `nextflow run QuackenbushLab/tcga-data-nf -profile <docker/conda>,testDownload`
   - test the prepare: `nextflow run QuackenbushLab/tcga-data-nf -profile <docker/conda>,testPrepare`
   - test the analyze: `nextflow run QuackenbushLab/tcga-data-nf -profile <docker/conda>,testAnalyze`
   - test the full workflow: `nextflow run QuackenbushLab/tcga-data-nf -profile <docker/conda>,test`

Configuration

The whole workflow can be personalized by changing the configuration parameters inside a `my-config.conf` file, which is then added to the workflow run as follows:

```
‘‘‘nextflow run QuackenbushLab/tcga-data-nf -c my-config.conf‘‘‘
```

First, three main parameters need to be passed by the user:

- `resultsDir = "results"`: general folder under which you want to find the results. This can be directly referenced as an AWS S3 bucket.
- `batchName = "my-batch"`: name of the run, this is gonna create a subfolder where the results are stored.
- `pipeline = "download"`: name of the pipeline, one of download, prepare, analyze, full.

This way, all data generated by the pipeline will be found inside the `resultsDir/batchName/` folder. If nothing is passed, all results will be in the `results/my-batch` folder. One of the advantages of Nextflow is the consistent organization of the results folder. For an updated and complete description of the result folders, please refer to the documentation[3].

For a complete list of the configuration parameters, refer to the docs[4].

1 https://www.nextflow.io/docs/latest/install.html
2 https://github.com/QuackenbushLab/tcga-data-nf/blob/main/docs.md#conda
3 https://github.com/QuackenbushLab/tcga-data-nf/blob/main/docs.md#result-folders
4 https://github.com/QuackenbushLab/tcga-data-nf/blob/main/docs.md#configurations

Download

Listing 1. download-test.json, Example of a JSON configuration file for the download step. With this file, the pipeline downloads all modalities for TCGA LUAD and GTEx lung.

```
{
    "expression_recount3": {
          "tcga_luad": {
                  "project": "LUAD",
                  "project_home" : "data_sources/tcga",
                  "organism" : "human",
                  "annotation" : "gencode_v26",
                  "type" : "gene",
                  "samples" : "NA"
                },
          "gtex_lung": {
                      "project": "LUNG",
                      "project_home" : "data_sources/gtex",
                      "organism" : "human",
                      "annotation" : "gencode_v26",
                      "type" : "gene",
                      "samples" : "NA"
                    }
          },
          "mutation_tcgabiolinks":{
            "tcga_luad":{
                  "project" : "TCGA-LUAD",
                  "data_category" : "Simple Nucleotide Variation",
                  "data_type" : "Masked Somatic Mutation",
                  "download_dir" : "gdc_tcga_mutation",
                  "samples" : "NA"
                }},
          "clinical_tcgabiolinks": {
            "tcga_luad":{
                  "project" : "TCGA-LUAD",
                  "data_category" : "Clinical",
                  "data_type" : "Clinical Supplement",
                  "data_format" : "BCR Biotab"
                  }},
          "methylation_gdc":{
            "tcga_luad":{
                  "project" : "TCGA-LUAD",
                  "gdc_type" : "methylation_beta_value",
                  "gdc_platform" : "illumina human methylation 450",
                  "download_dir" : "gdc_tcga_methylation",
                  "samples" : "NA"
                }
          }
    }
```

Listing 2. full-test.json, Example of a JSON configuration file for the full pipeline. With this file, we specify which modalities and samples will be downloaded, pre-processed, and analyzed. In this case, we are interested in LUAD samples.

```
{ "tcga_luad": {
        "expression_recount3": {
              "project" : "LUAD",
              "project_home" : "data_sources/tcga",
              "organism" : "human",
              "annotation" : "gencode_v26",
              "type" : "gene",
              "samples" : "testdata/tcga_luad_samples.txt"
            },
      "mutation_tcgabiolinks": {
              "project" : "TCGA-LUAD",
              "data_category" : "Simple Nucleotide Variation",
              "data_type" : "Masked Somatic Mutation",
              "download_dir" : "gdc_tcga_mutation",
              "samples" : "testdata/tcga_luad_mutation_samples.txt"
            },
      "clinical_tcgabiolinks": {
              "project" : "TCGA-LUAD",
              "data_category" : "Clinical",
              "data_type" : "Clinical Supplement",
              "data_format" : "BCR Biotab"
              },
      "methylation_gdc": {
              "project" : "TCGA-LUAD",
              "gdc_type" : "methylation_beta_value",
              "gdc_platform" : "illumina human methylation 450",
              "download_dir" : "gdc_tcga_methylation",
              "samples" : "testdata/tcga_luad_samples.txt"
            }
      }
    }
```

Supplementary Figures

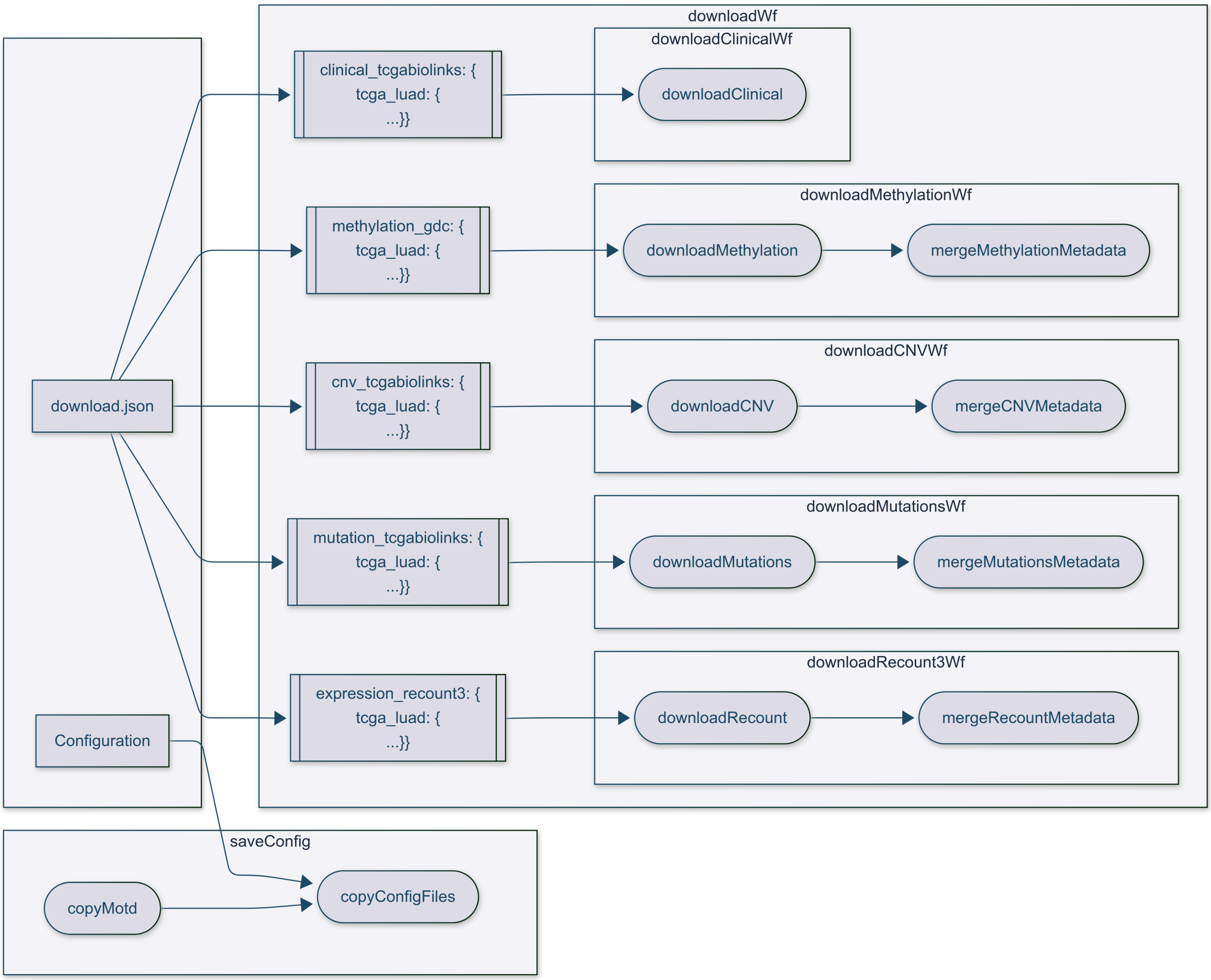

Figure S1. Download. Directed Acyclic Graph of the processes specified in the **Download** pipeline. For each modality that is specified in the configuration file, *tcga-data-nf* downloads the data and generates metadata tables with the names, paths, and parameters of the files. Whenever *tcga-data-nf* is run, we also generate and save the configuration parameters, which can then be examined and reused (saveConfig process).

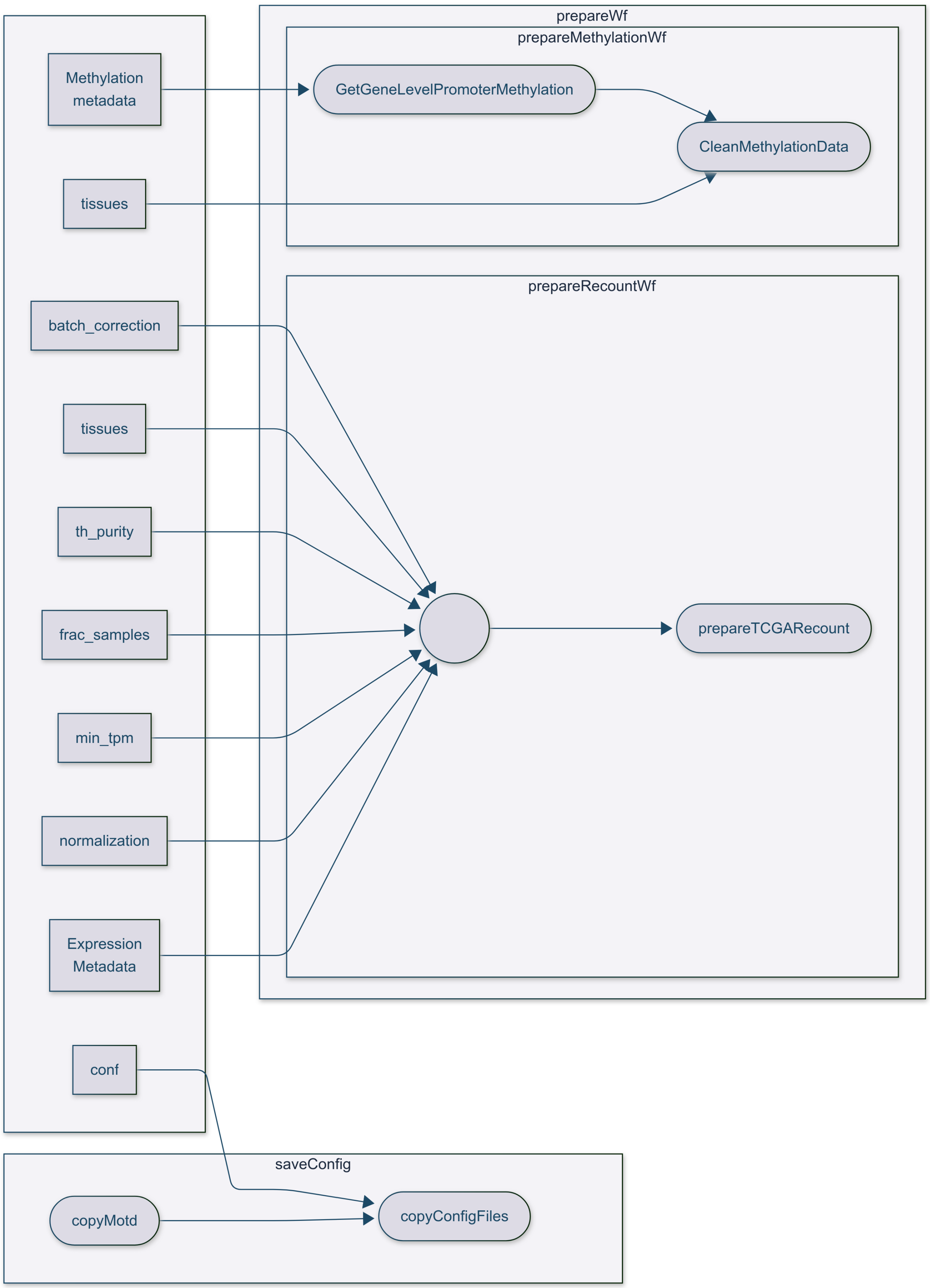

Figure S2. Prepare. Directed Acyclic Graph of the processes specified in the **Prepare** pipeline. The expression and methylation data specified in the configuration metadata are processed using the combination of all input parameters (tissues, purity, minTPM...). Whenever *tcga-data-nf* is run, we also generate and save the configuration parameters, which can then be examined and reused (saveConfig process).

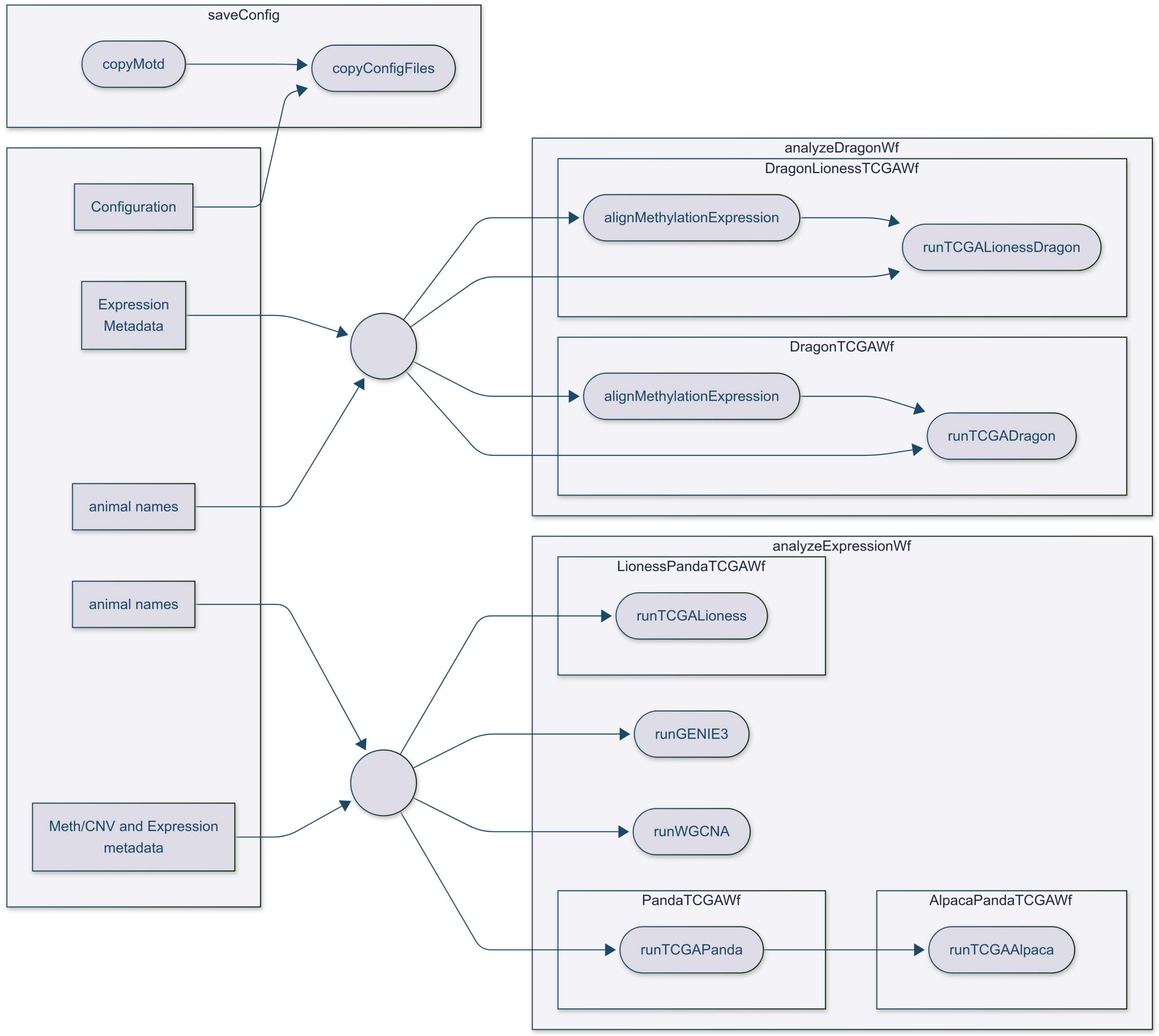

Figure S3. Analyze. Directed Acyclic Graph of the processes specified in the Analyze pipeline. Using the input metadata, the *tcga-data-nf* workflow generates PANDA, DRAGON, LIONESS, GENIE3, and WGCNA networks, and matches them with log files and intermediate tables, useful for further investigation of the results. PANDA networks are compared with ALPACA. Whenever *tcga-data-nf* is run, we also generate and save the configuration parameters, which can then be examined and reused (saveConfig process).

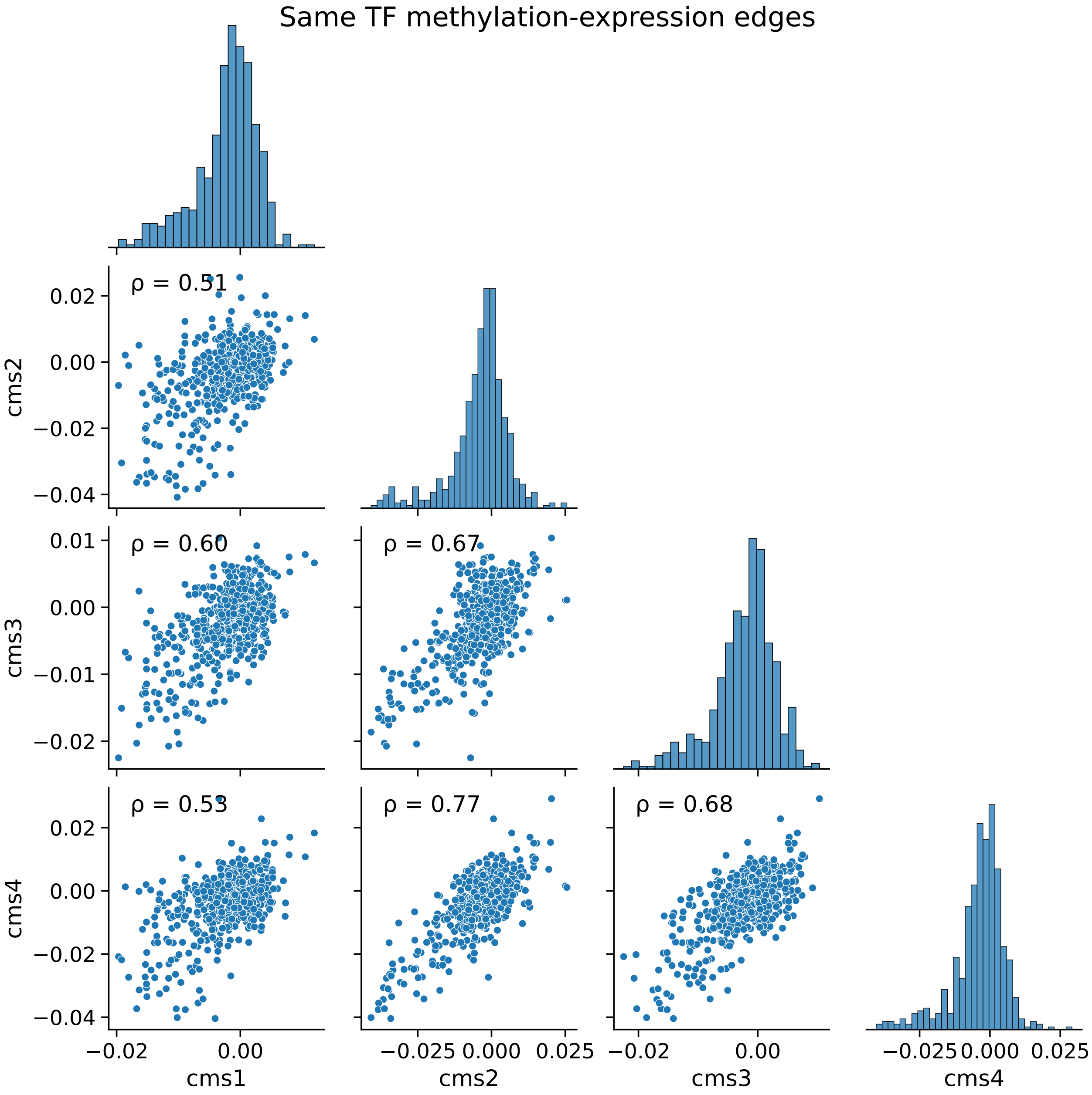

Figure S4. Correlation between DRAGON methylation-expression edges on the same TFs. For all (M_i, E_i) edges, we plot their distribution in each subtype (histograms on the diagonal) and the correlation of the edge weights between each pair of subtypes. While all Pearson correlation values are above 0.50 it is worth noting that CMS2 and CMS4 are the most similar to each other with $\rho = 0.77$. This indicates that many of the TFs with negative partial correlations between methylation and expression are conserved across the CMS2 and CMS4 subtypes, whereas they are more distinct for CMS1.

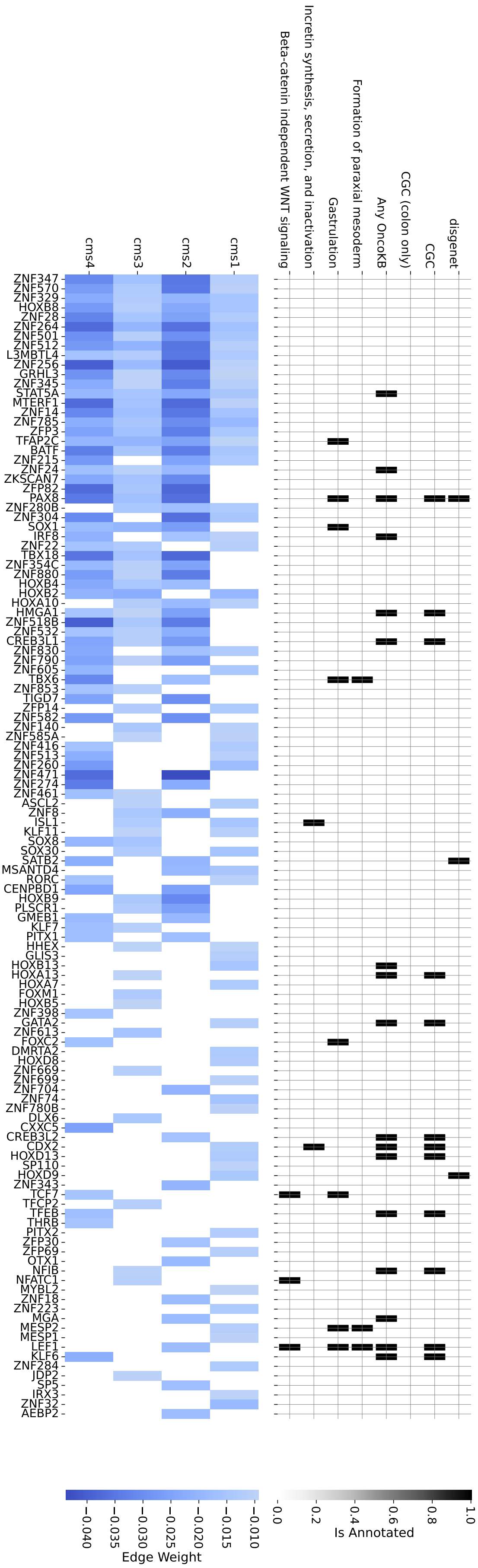

Figure S5. TFs with evidence of epigenetic effect on expression. From all (M_i, E_i) edges, for each subtype, we select those whose values are in the first decile of the distribution, that is, the smallest 10% of edges. Here we plot the edge value on the left and the annotation to one of each TF to a cancer-related database. In particular, OncoKB (downloaded 12/13/2022), Cancer Gene Census (v101, downloaded 04/25/2025), and DisGeNet (GDA CURATED C0009404, downloaded on 04/25/2025).

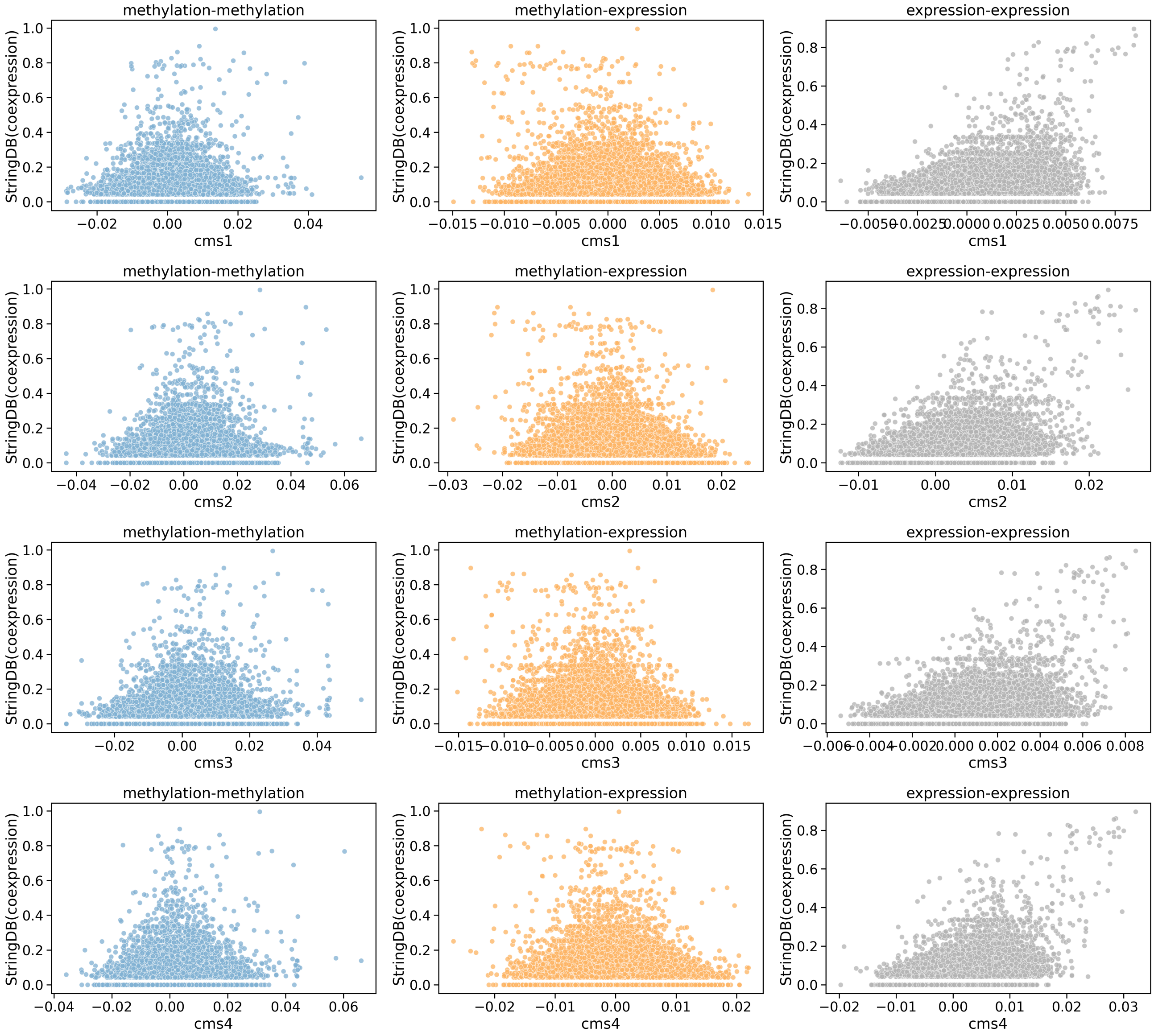

Figure S6. Relationship between DRAGON edges and StringDB co-expression evidence. For each colon cancer subtype (rows), we plot the values of the DRAGON edges on the x-axis, grouped by edge type (columns), and the confidence score in the StringDB database on the y-axis. For all subtypes, there is evidence of a correlation between the DRAGON and StringDB co-expression, confirming the validity of our inferred associations; methylation-methylation and methylation-expression edges serve as negative controls.

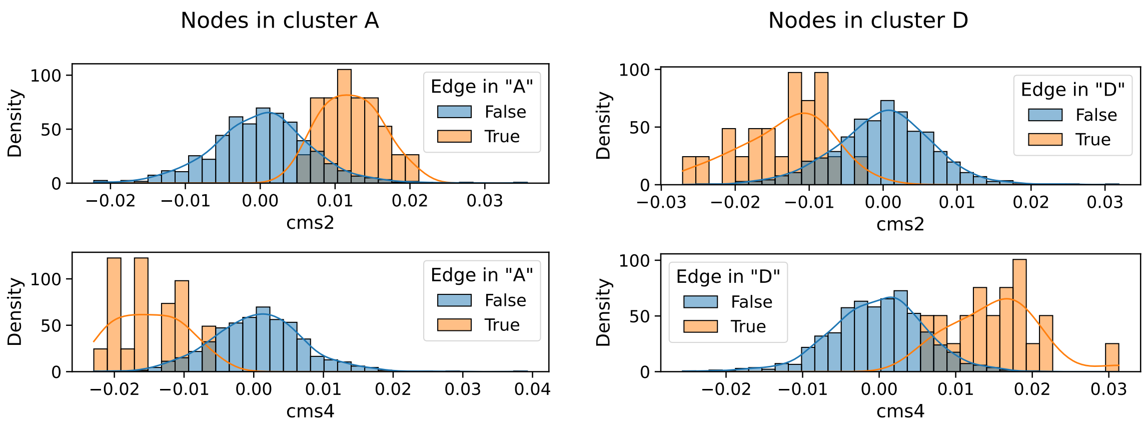

Figure S7. Edge weights for nodes in Cluster A and D of the DRAGON networks. We select first the subgraphs with all the nodes in each cluster. It is worth noting that these graphs contain both the edges represented in Figure 2B, and those that connect the nodes in the cluster but were not the strongest edges. We compare the values of the edges of interest (orange), which are shown in Figure 2B, and the rest of the edges (blue) that connect the same TFs, as controls. We observe that for both groups the average edge value is around 0, meaning that there is no general difference between the edge values of the two subtypes. However, the edges selected in the clusters switch between higher and lower values for CMS2 and CMS4.

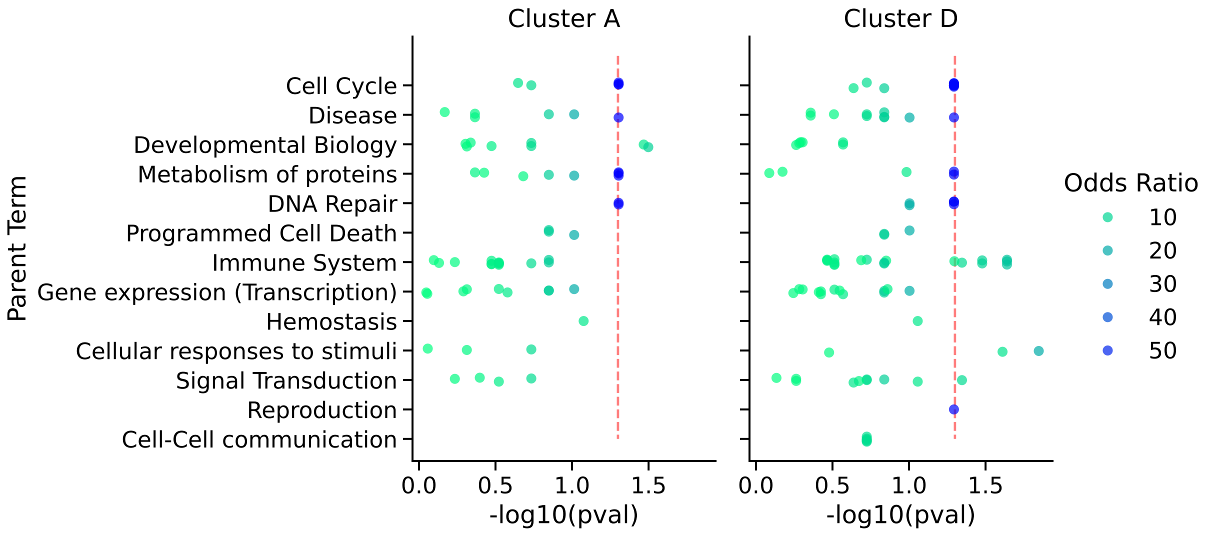

Figure S8. Reactome pathways for clusters A and D. Using the clusters found from the DRAGON edges, we run a pathway over-representation analysis of the TFs in both cluster A and D. With REACTOME, we can identify the general pathway to which each term belongs. For each "parent" pathway (y-axis), we plot the $-log_{10}(pvalue)$ (x-axis) of all the pathways tested that belong to that parent term, and we color them by the corresponding Odds-Ratio. The red dashed line corresponds to a p-value of 0.05, which is the line for nominal significance. Since pathway analysis on TFs is challenging (there are only $\sim$ 1000 TFs and many of them are annotated only to the general transcriptional pathway terms), we report here all results, even those that are not significant, such that one can observe the general trend. TFs in cluster D seem to be more consistently annotated to Immune System pathways, while the TFs in cluster A have some stronger terms related to Developmental Biology.

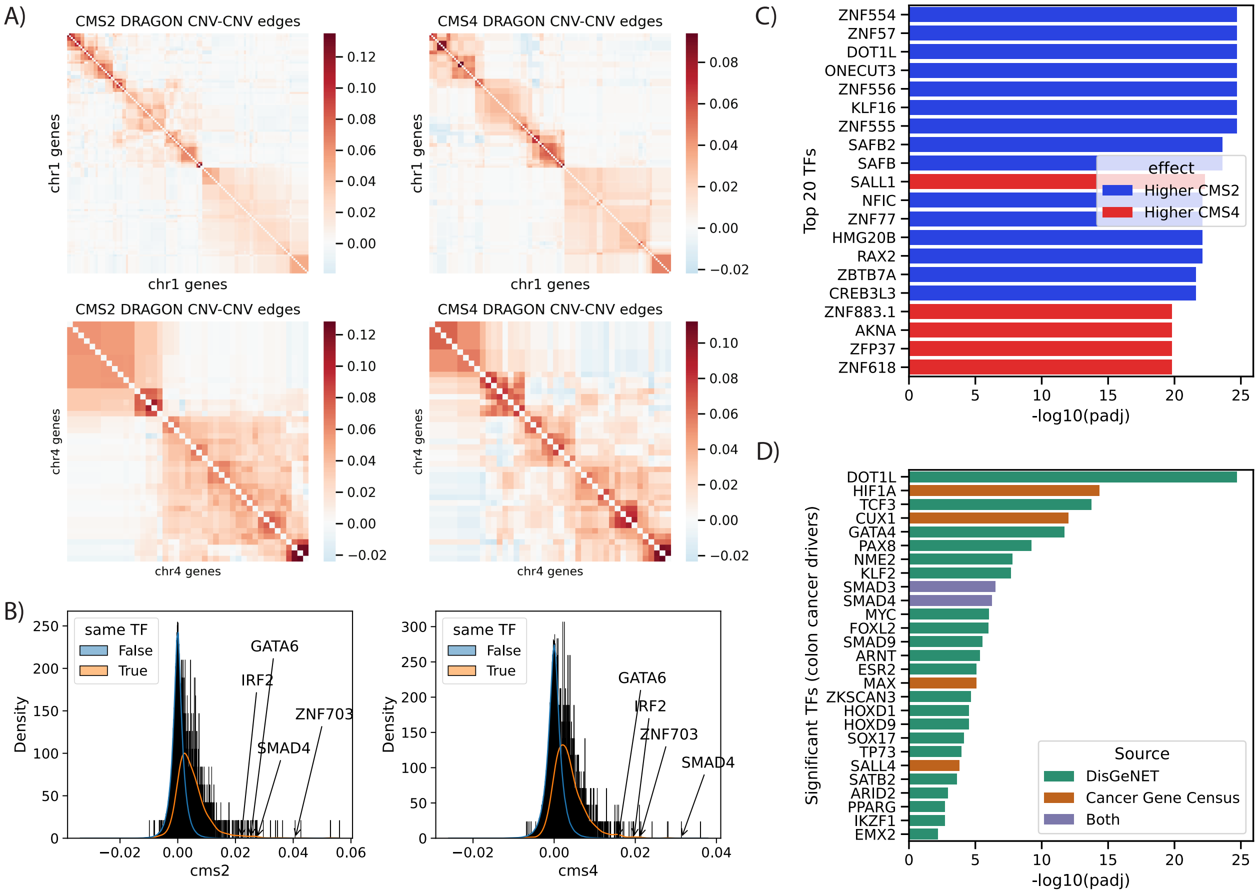

Figure S9. DRAGON CNV-expression networks. A) DRAGON CNV-CNV edges for genes in chromosomes 1 and 4. For both CMS2 and CMS4, there is evidence of a block structure that depends on the genome location. Adjacent genes are more likely to be correlated. B) Distribution of partial correlation values between CNV and expression of TFs in both subtypes. In orange we show the values for the edges of the same TF ((C_i, E_i)), while in blue we show all the others ((C_i, E_j)) . As expected, CNV and expression tend to be positively correlated. The histogram represents the distribution density, and it is normalized per subtype and per group. We have also annotated the 20 TFs that have the greatest mean value in both subtypes that are also known for their role in cancer. C) TFs with CNV-expression edges that are significantly different between CMS2 and CMS4. We show the 20 with the lowest adjusted p-value (Benjiamini-Hochberg FDR) from the paired Wilcoxon signed-rank test. D) Colon cancer-related TFs with CNV-expression edges that are significantly different between CMS2 and CMS4. We retrieved the colon cancer drivers from both the DisGeNET and the Cancer Gene Census datasets.

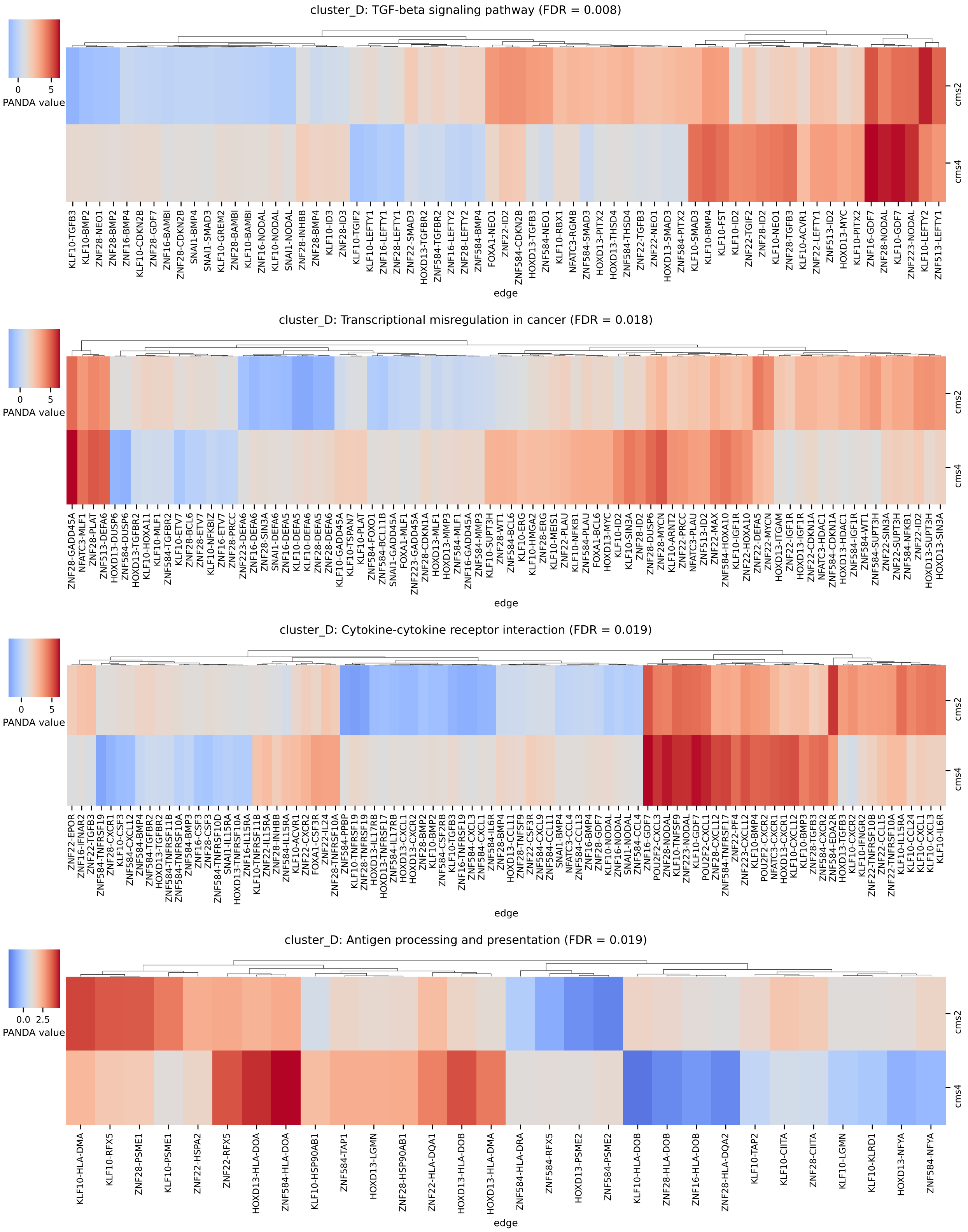

Figure S10. PANDA edges involved in the main pathways targeted by cluster D. We selected the regulatory edges of the TFs in cluster D (defined by the analysis on DRAGON networks), and we investigated which edges in the PANDA networks underwent the biggest changes between CMS2 and CMS4. The target genes of the edges were found to be preferentially involved in the TGF-beta signaling pathway, Transcriptional misregulation in cancer, Cytokine-cytokine receptor interaction, and Antigen processing and presentation. Here, we represent the PANDA edges (edge weight represented by different colors) connecting the main targets for each pathway, for both CMS2 and CMS4 (rows).

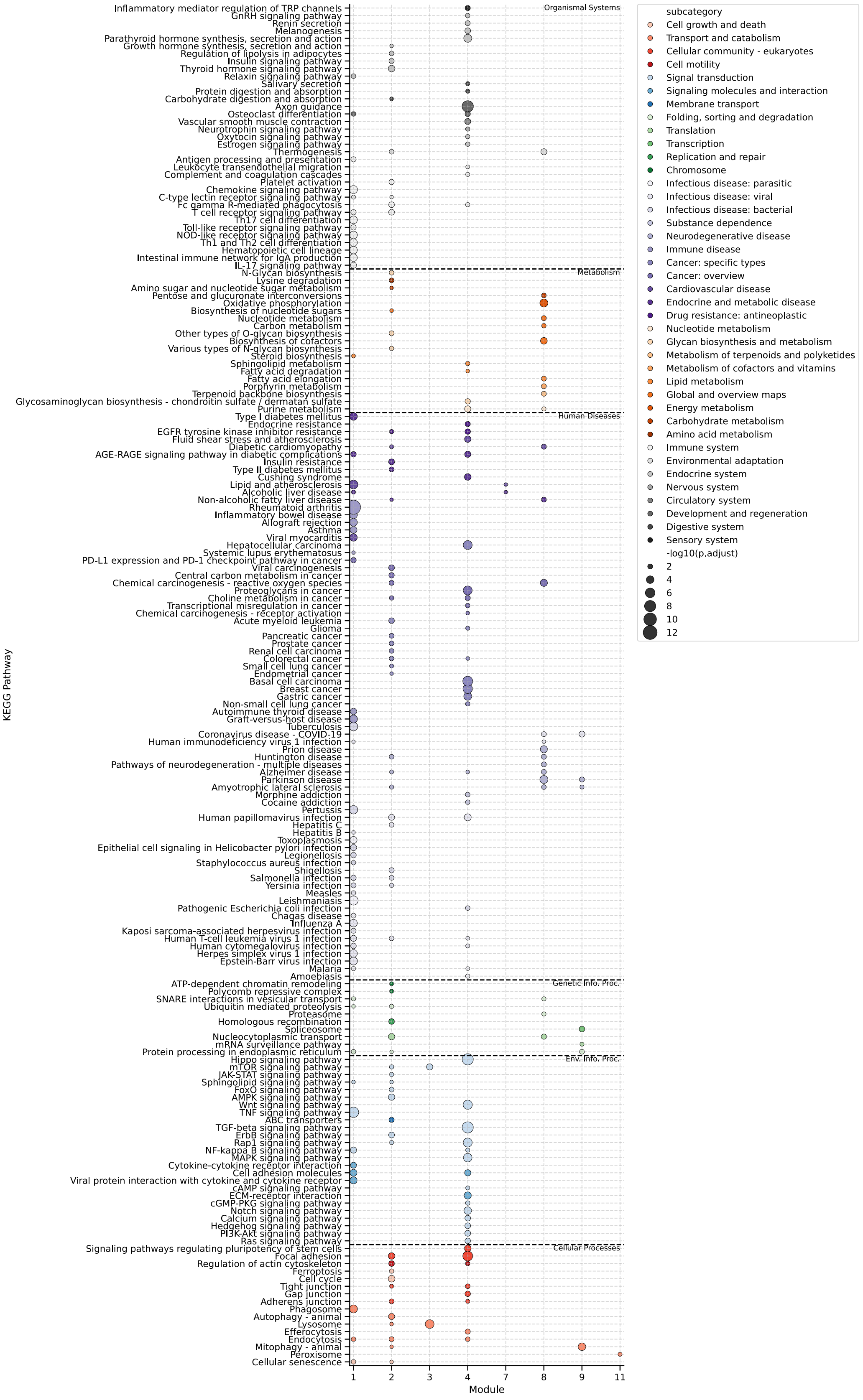

Figure S11. Pathway analysis of ALPACA's modules. For each module, we ran the pathway analysis between the nodes in each module and the KEGG pathways, using R's clusterProfiler package. We grouped the results based on the KEGG subcategory and counted the significant terms (FDR-adjusted *p*-value < 0.05). We removed clusters with no significant pathway over-representations. We remove clusters with no significant pathway over-representations.

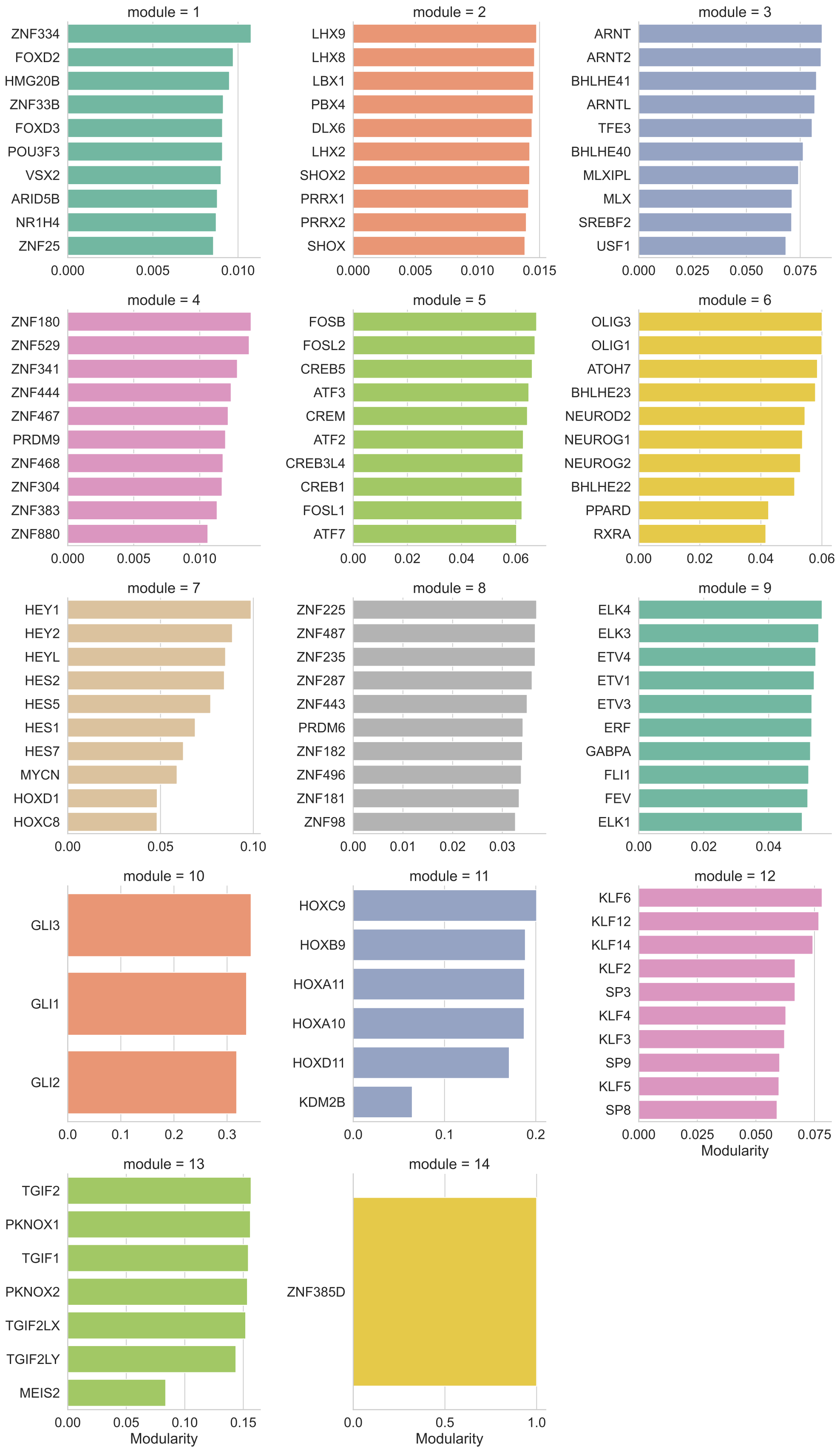

Figure S12. ALPACA's top TFs. For each module, we selected the TFs with the highest modularity up to a total of 10 TFs (for modules larger than size 10), that is, those that contribute the most to the differential modularity between CMS2 and CMS4

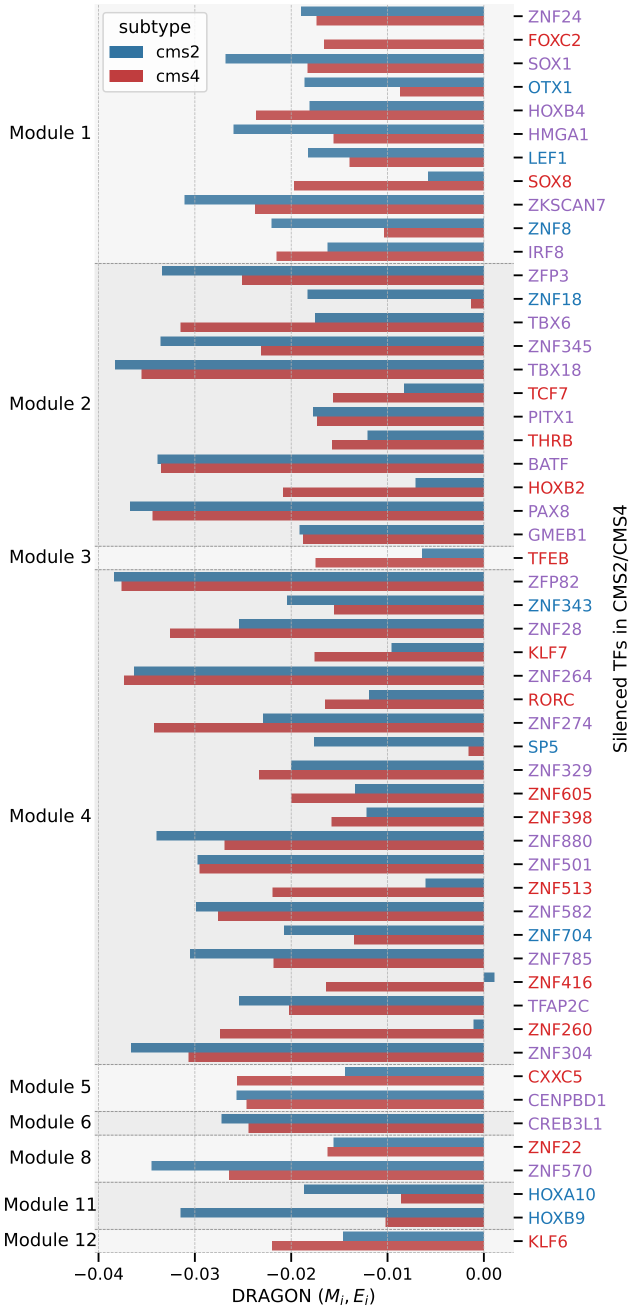

Figure S13. Overlaps between DRAGON and ALPACA results. For each "silenced" TF that we get from the DRAGON analysis, we plot the partial correlation values between promoter methylation and expression (x-axis) in CMS2 and CMS4 (red and blue bars). The name of the TF is colored based on whether it is significantly silenced in CMS2 (red), CMS4 (blue), or both (purple). The TFs are organized by the ALPACA module they belong to and we have skipped those that do not have any DRAGON TFs. Of particular interest is Module 11, where both HOX TF have evidence of promoter methylation silencing in subtype CMS2 and not in CMS4.

Supplementary Tables

REACTOME enrichment for targets of TFs in cluster D

Term	Overlap	P-value	Adjusted P-value	Odds Ratio
Creation of C4 and C2 activators	31/81	1.1188890835418E-09	6.90354564545292E-07	4.6464702446038900
Cell surface interactions at the vascular wall	51/182	3.0709751817686E-09	9.47395843575613E-07	2.928937872861060
Binding and Uptake of Ligands by Scavenger Receptors	34/102	1.16220368969732E-08	2.39026558847748E-06	3.7526856922617600
Initial triggering of complement	31/89	1.56987176647831E-08	2.42152719979279E-06	4.00919352961713
Leishmania phagocytosis	39/129	2.23130556805187E-08	2.75343107097601E-06	3.254975191441080
Complement cascade	35/110	2.75594984807199E-08	2.83403509376736E-06	3.5035349241328300
Fcgamma receptor (FCGR) dependent phagocytosis	41/156	6.8681651106458E-07	6.05379696181208E-05	2.6779980022871300
Signaling by the B Cell Receptor (BCR)	42/167	1.77910217466724E-06	0.00013721325522121100	2.5236253682339000
Anti-inflammatory response favouring Leishmania parasite infection	35/137	8.63735349188979E-06	0.0005921385671662220	2.576603530486490
rRNA processing in the mitochondrion	14/35	2.31537263450701E-05	0.0014285849154908300	4.995559391886160
Fc epsilon receptor (FCERI) signaling	40/194	0.0003690257884500380	0.020698991952152200	1.9484735437419800
Class A/1 (Rhodopsin-like receptors)	35/167	0.0006085153001697720	0.03128782835039580	1.9897413064168900
GPCR ligand binding	42/224	0.001961469140422810	0.09309434304929800	1.7296595421891000
Peptide ligand-binding receptors	23/105	0.002715058138266520	0.11965649080788900	2.110627272074610
Amine ligand-binding receptors	5/10	0.0036276202738507000	0.14921611393105900	7.38555246847617
FOXO-mediated transcription	13/53	0.008199622126970790	0.31619792827131100	2.4652941596969000
Insulin-like Growth Factor-2 mRNA Binding Proteins (IGF2BPs/IMPs/VICKZs) bind RNA	4/8	0.009540610302219240	0.34626803273348600	7.382824182866910
Antimicrobial peptides	8/28	0.014087406060553000	0.4583737223150080	3.0635722599468700
FGFR4 ligand binding and activation	3/5	0.014115236181499400	0.4583737223150080	10.332735426009000
SARS-CoV Infections	68/442	0.01686712763842170	0.5203508876453100	1.358632067296990
Amyloid fiber formation	10/42	0.023488719520119900	0.6310051520689820	2.3874754930227200
Dissolution of Fibrin Clot	4/10	0.023522072119265100	0.6310051520689820	5.11059438318571
Kidney development	9/36	0.022815479951734500	0.6310051520689820	2.552488273875560
Regulation of necroptotic cell death	8/31	0.025896689199173200	0.6657607181620780	2.672013513575040
Biological oxidations	26/153	0.0400358591366688	0.8451238953644460	1.5380033574784200
Innate Immune System	131/945	0.03681566433944630	0.8451238953644460	1.2012559517099100
Myogenesis	6/22	0.039771494279398200	0.8451238953644460	2.9088511801789500
Loss of Function of TGFBR1 in Cancer	3/7	0.041091923599567900	0.8451238953644460	5.739744228533470
Adaptive Immune System	106/750	0.035921911295563	0.8451238953644460	1.2283152941168300
Defective RIPK1-mediated regulated necrosis	3/7	0.041091923599567900	0.8451238953644460	5.739744228533470

1

Table S1. REACTOME pathway enrichment for the targets of TFs in cluster D. For the targets of the TFs in cluster D, we run a pathway over-representation analysis with the REACTOME pathway database. Here we show the pathways with p-value < 0.05.

REACTOME enrichment for targets of TFs in cluster A

Term	Overlap	P-value	Adjusted P-value	Odds Ratio
Leishmania phagocytosis	26/129	4.21569919738418E-07	0.0002360791550535140	3.5828676075905400
Cell surface interactions at the vascular wall	30/182	4.83181100421583E-06	0.000825468123954678	2.799768607790200
Fcgamma receptor (FCGR) dependent phagocytosis	27/156	5.5958460183126E-06	0.000825468123954678	2.969623006175530
Creation of C4 and C2 activators	18/81	5.89620088539056E-06	0.000825468123954678	4.060816767411140
Complement cascade	21/110	1.28921880837977E-05	0.0012716123180039300	3.3512599252410800
Binding and Uptake of Ligands by Scavenger Receptors	20/102	1.36244176928992E-05	0.0012716123180039300	3.465189205487710
Initial triggering of complement	18/89	2.3373435996165E-05	0.0018698748796932000	3.604877735943840
Anti-inflammatory response favouring Leishmania parasite infection	22/137	0.00012709123933941700	0.008896386753759200	2.7158487353964500
Signaling by the B Cell Receptor (BCR)	25/167	0.00014565613639090400	0.009063048486545160	2.4968073275774900
Fc epsilon receptor (FCERI) signaling	25/194	0.0014133478427089400	0.07914747919170080	2.0959648329531300
Kidney development	8/36	0.00234245308328935	0.1192521569674580	4.133478367895470
FOXO-mediated transcription	10/53	0.002585571926244530	0.1206600232247450	3.3476825962256200
Aspirin ADME	5/19	0.007310164794079320	0.31489940651418600	5.249063890302300
Loss of function of MECP2 in Rett syndrome	4/13	0.009053510120533320	0.3621404048213330	6.551885384538850
Myogenesis	5/22	0.013989648003434500	0.45044475301132200	4.348510891303170
Innate Immune System	81/945	0.01567025708207290	0.45044475301132200	1.3198759025841800
Insulin-like Growth Factor-2 mRNA Binding Proteins (IGF2BPs/IMPs/VICKZs) bind RNA	3/8	0.013319620109513900	0.45044475301132200	8.79730054607274
Transcriptional regulation of granulopoiesis	6/31	0.016087312607547200	0.45044475301132200	3.5279937041079200
Transcriptional regulation by the AP-2 (TFAP2) family of transcription factors	6/30	0.013739054736949700	0.45044475301132200	3.6721943605158800
Glucuronidation	4/15	0.015512274229252800	0.45044475301132200	5.411835292183780
Dissolution of Fibrin Clot	3/10	0.02578302194706470	0.6562951041071020	6.450648532930360
Regulation of CDH11 function	3/10	0.02578302194706470	0.6562951041071020	6.450648532930360
Transcriptional regulation by RUNX3	12/96	0.02808705812843130	0.6838588066052840	2.050130429596150
Smooth Muscle Contraction	6/36	0.032000930629106800	0.7466883813458260	2.9488271332890500
Signaling by NOTCH1 HD+PEST Domain Mutants in Cancer	8/57	0.03678967677180870	0.7923930381620330	2.377146512527630
Signaling by NOTCH1 PEST Domain Mutants in Cancer	8/57	0.03678967677180870	0.7923930381620330	2.377146512527630
Postsynaptic nicotinic acetylcholine receptors	2/5	0.03976267467822500	0.8247073266594800	9.868144313217930
Surfactant metabolism	4/20	0.042127490094425900	0.8425498018885190	3.770854098642380
Signaling by NOTCH1	9/70	0.045349117204883900	0.8550701771095160	2.138624828373520
Signaling by NOTCH4	10/81	0.04580733091658120	0.8550701771095160	2.0335786507453800
GPCR ligand binding	22/224	0.04985446694235830	0.8554507505937300	1.5416294024956300
Glycosphingolipid metabolism	7/50	0.04965414778353900	0.8554507505937300	2.38576524545609

1

Table S2. REACTOME pathway enrichment for the targets of TFs in cluster A. For the targets of the TFs in cluster A, we performed a pathway over-representation analysis using the REACTOME pathway database. Here we show the pathways with p-value < 0.05.

KEGG 2021 enrichment for targets of TFs in cluster D

Term	P-value	Adjusted P-value	Odds Ratio
TGF-beta signaling pathway	2.48574311940069E-05	0.0077058036701421300	3.019988310929280
Transcriptional misregulation in cancer	0.00011838600511097900	0.018349830792201800	2.2167008430166300
Cytokine-cytokine receptor interaction	0.00018606258744939800	0.018861201326119900	2.01001783033033
Antigen processing and presentation	0.00024337033969187000	0.018861201326119900	3.1492372152986700
Amoebiasis	0.003167301213570950	0.16063641285107300	2.2336917562724000
Asthma	0.003300331201939580	0.16063641285107300	4.703154782744
Viral protein interaction with cytokine and cytokine receptor	0.003627273838572620	0.16063641285107300	2.25914860748794
Gastric cancer	0.004165072298866050	0.16139655158106000	1.930047973264340
Pathways in cancer	0.005309881763878660	0.18289592742248700	1.4278343896029800
Hematopoietic cell lineage	0.00642522348971196	0.19918192818107100	2.169620497014060
Epstein-Barr virus infection	0.00857343648688083	0.22511457232477200	1.6532418398493700
Arachidonic acid metabolism	0.008714112477087950	0.22511457232477200	2.6239508869930700
Neuroactive ligand-receptor interaction	0.011684597675844700	0.2734075242836080	1.7469509466507200
Calcium signaling pathway	0.013035464353594000	0.2734075242836080	1.6528891842498800
Signaling pathways regulating pluripotency of stem cells	0.013803833745416300	0.2734075242836080	1.7899688597363000
Retinol metabolism	0.014111356092057200	0.2734075242836080	2.54912389563573
Staphylococcus aureus infection	0.01520219063915510	0.27721641753753300	2.185045435045440
IL-17 signaling pathway	0.01630080366536720	0.2807360631257680	1.972852233676980
Inflammatory bowel disease	0.020345197764012200	0.33194796351809400	2.2588584083440500
Intestinal immune network for IgA production	0.02281522942217870	0.3536360560437700	2.4633832976445400
Primary immunodeficiency	0.02589642462614270	0.36567840481762300	2.5699821322215600
Linoleic acid metabolism	0.025951370664476400	0.36567840481762300	3.6923899102180400
Complement and coagulation cascades	0.029464306184411400	0.3971276050942400	1.9615125329411000
Type I diabetes mellitus	0.031024169351406000	0.40072885412232700	2.4627568493150700
Autoimmune thyroid disease	0.03533995207094060	0.4382154056796640	2.585387248609330
Graft-versus-host disease	0.042424200647072800	0.5032938481671670	2.4621309370988400
Prostate cancer	0.043835270646817800	0.5032938481671670	1.6761839278040400
Hepatocellular carcinoma	0.04938104058133400	0.5467186635790540	1.4917963805311000

1

Table S3. KEGG pathway enrichment for the targets of TFs in cluster D. For the targets of the TFs in cluster D, we run a pathway over-representation analysis with the KEGG pathway database. Here we show the pathways with p-value < 0.05.

KEGG 2021 enrichment for targets of TFs in cluster A

Term	P-value	Adjusted P-value	Odds Ratio
Pathways in cancer	0.000747938	0.14230677574040300	1.7114527709087400
Transcriptional misregulation in cancer	0.000978053	0.14230677574040300	2.302796633179470
Gastric cancer	0.003165216	0.250779096	2.272352647352650
Intestinal immune network for IgA production	0.009353948	0.250779096	3.3438080050194800
Hippo signaling pathway	0.005438134	0.250779096	2.0849770642201800
Hepatocellular carcinoma	0.005589136	0.250779096	2.031952209993530
Basal cell carcinoma	0.007511519	0.250779096	2.9016263177411700
Cytokine-cytokine receptor interaction	0.004160565	0.250779096	1.9401753529151800
PPAR signaling pathway	0.015161895289182000	0.250779096	2.545488354795220
Endometrial cancer	0.013600153655384300	0.250779096	2.5986617312072900
Bacterial invasion of epithelial cells	0.012675416382725400	0.250779096	2.475711029092490
Bile secretion	0.012160736019803900	0.250779096	2.6540978044879600
Hematopoietic cell lineage	0.011645194336257000	0.250779096	2.383638307984790
Colorectal cancer	0.011599440441574000	0.250779096	2.280352786639140
Epstein-Barr virus infection	0.010780171259324300	0.250779096	1.8245258620689700
TGF-beta signaling pathway	0.00965665	0.250779096	2.3448453276738100
Fatty acid biosynthesis	0.015512109	0.250779096	5.030944849401730
Linoleic acid metabolism	0.015512109	0.250779096	5.030944849401730
Metabolism of xenobiotics by cytochrome P450	0.018044423	0.2723088279033320	2.6387744779247100
Adherens junction	0.018715383361053700	0.2723088279033320	2.310157042
Porphyrin and chlorophyll metabolism	0.021584665678258800	0.28550626	3.0764309764309800
Steroid hormone biosynthesis	0.021584665678258800	0.28550626	3.0764309764309800
Mineral absorption	0.026930818315737400	0.3407333969512860	2.61967502
Thyroid cancer	0.028226189	0.34224254437779000	2.863949843260190
Asthma	0.029600377927220100	0.3445483990728420	3.952236870542470
Viral protein interaction with cytokine and cytokine receptor	0.033977179	0.3802830416208250	2.068003448
Drug metabolism	0.036708966396366000	0.395641082	2.037479886
Ether lipid metabolism	0.040496699562009600	0.4208764133051710	2.5950284090909100
Retinol metabolism	0.045230707223669600	0.45386675179613300	2.5162534435261700
Ascorbate and aldarate metabolism	0.049349391	0.47868908895841100	3.254249354810000

1

Table S4. KEGG pathway enrichment for the targets of TFs in cluster A. For the targets of the TFs in cluster A, we performed a pathway over-representation analysis using the KEGG pathway database. Here we show the pathways with p-value < 0.05.

module	stat	p	Bonferroni FWER	mean IN-module degree	mean OUT-module degree
1	385813.00	0.00E+00	0.00E+00	-78.194178	43.655882
2	2143764.00	1.87E-32	5.81E-31	2.421301	10.373974
3	36981.00	5.90E-03	1.83E-01	-0.132168	13.994770
4	1433866.00	0.00E+00	0.00E+00	-63.115197	89.458204
5	98890.00	2.12E-04	6.58E-03	0.165063	12.623169
6	104579.00	2.28E-19	7.05E-18	0.347839	28.194577
7	32643.00	3.15E-05	9.78E-04	-0.414056	21.782637
8	2663962.00	8.13E-07	2.52E-05	-4.844230	2.151773
9	218707.00	7.61E-07	2.36E-05	-1.094298	14.393717
10	955.00	8.54E-03	2.65E-01	0.074718	39.860753
11	869.00	1.59E-09	4.93E-08	0.659262	53.029421
12	98998.00	3.37E-01	1.00E+00	-0.456192	9.702970
13	9620.00	6.20E-02	1.00E+00	-0.154742	14.593134
14	62.00	1.96E-01	1.00E+00	0.024142	-20.562472

Table S5. Comparison of edge differences (CMS2-CMS4) for each gene's IN-module vs OUT-module degree. For each module, we apply a pairwise Wilcoxon signed-rank test, for which we report the statistic and *p*-value, and we compute the Bonferroni FWER. We also report the average IN-module and OUT-module degrees.

module	stat	p	Bonferroni FWER	mean IN-module degree	mean OUT-module degree
1	40.00	1.02E-42	1.43E-41	-1121.821654	1331.685448
2	7088.00	1.77E-12	2.47E-11	33.390045	133.276001
3	11.00	8.39E-04	1.17E-02	-3.249790	-110.794156
4	788.00	9.05E-42	1.27E-40	-1139.382961	1268.302363
5	207.00	8.31E-01	1.00E+00	3.910279	-2.354879
6	2.00	1.40E-09	1.96E-08	8.815545	115.128064
7	83.00	4.30E-01	1.00E+00	-8.550258	13.984968
8	254.00	1.91E-06	2.67E-05	-282.032701	170.765688
9	136.00	1.15E-04	1.62E-03	-28.205519	-121.859152
10	3.00	1.00E+00	1.00E+00	1.892866	-2.441624
11	0.00	3.12E-02	4.38E-01	11.427210	139.448435
12	28.00	6.94E-04	9.71E-03	-13.333253	-158.706423
13	0.00	1.56E-02	2.19E-01	-4.686480	206.724260
14	0.00	1.00E+00	1.00E+00	0.458702	-102.928285

Table S6. Comparison of edge differences (CMS2-CMS4) for each TF's IN-module vs OUT-module degree. For each module, we apply a pairwise Wilcoxon signed-rank test of which we report the statistic and *p*-value, and we compute the Bonferroni FWER. We also report the average IN-module and OUT-module degrees.
