# Supplementary material for: Reproducible processing of TCGA regulatory networks
Source: Gigascience. 2025 Oct 20;14:giaf126. doi: 10.1093/gigascience/giaf126 (PMC12720619; doi:10.1093/gigascience/giaf126)
Supplement: giaf126_GIGA-D-24-00535_Revision_1 [file giaf126_giga-d-24-00535_revision_1.pdf]

GigaScience

Reproducible processing of TCGA regulatory networks

--Manuscript Draft--

**Manuscript Number:**	GIGA-D-24-00535R1
**Full Title:**	Reproducible processing of TCGA regulatory networks
**Article Type:**	Technical Note

Funding Information:

NATIONAL CANCER INSTITUTE, NIH (R35CA220523)	Prof John Quackenbush
NATIONAL CANCER INSTITUTE, NIH (U24CA231846)	Prof John Quackenbush
NATIONAL CANCER INSTITUTE, NIH (P50CA127003)	Prof John Quackenbush
National Human Genome Research Institute (R01HG011393)	Prof John Quackenbush
NHLBI, NIH (T32HL007427)	Dr Katherine H. Shutta
NHLBI, NIH (P01HL114501)	Dr Katherine H. Shutta
NHLBI, NIH (K01HL166376)	Dr Camila M. Lopes-Ramos
American Lung Association (LCD-821824)	Dr Camila M. Lopes-Ramos

Abstract:

Background. Technological advances in sequencing and computation have allowed deep exploration of the molecular basis of
diseases. Biological networks have proven to be a useful framework for interrogating omics data and modeling regulatory gene and protein interactions. Large collaborative projects, such as The Cancer Genome Atlas (TCGA), have provided a rich resource for building and validating new computational methods resulting in a plethora of open-source software for downloading, pre-processing, and analyzing those data. However, for an end-to-end analysis of regulatory networks, a coherent and reusable workflow is essential to integrate all relevant packages into a robust pipeline.
Findings. We developed tcga-data-nf, a Nextflow workflow that allows users to reproducibly infer regulatory networks from the
thousands of samples in TCGA using a single command. The workflow can be divided into three main steps: multi-omics data,
such as RNA-seq and methylation, are (i) downloaded, (ii) pre-processed, and (iii) analyzed to infer regulatory networkmodels
with the Network Zoo. The workflow is powered by the NetworkDataCompanion R package, a standalone collection of functions for managing, mapping, and filtering TCGA data. Here, we demonstrate how the pipeline can be used to study the differences between colon cancer subtypes that could be explained by epigenetic mechanisms. Lastly, we provide a database of pre-generated networks for the 10 most common cancer types that can be readily accessed by the public.
Conclusions. tcga-data-nf is a complete yet flexible and extensible framework that enables the reproducible inference and
analysis of cancer regulatory networks, bridging a gap in the current universe of software tools for analyzing TCGA data.

**Corresponding Author:**	John Quackenbush<br>Harvard University HSPH: Harvard University T H Chan School of Public Health<br>Boston, MA UNITED STATES
**Corresponding Author Secondary Information:**	
**Corresponding Author's Institution:**	Harvard University HSPH: Harvard University T H Chan School of Public Health
**Corresponding Author's Secondary Institution:**	

Powered by Editorial Manager® and ProduXion Manager® from Aries Systems Corporation

**First Author:**	Viola Fanfani
**First Author Secondary Information:**	
**Order of Authors:**	Viola Fanfani
	Katherine H. Shutta
	Panagiotis Mandros
	Jonas Fischer
	Enakshi Saha
	Soel Micheletti
	Chen Chen
	Marouen Ben Guebila
	Camila M. Lopes-Ramos
	John Quackenbush
**Order of Authors Secondary Information:**	
**Response to Reviewers:**	Dear Dr. Lan,<br>Attached please find a revised version of our paper, “Reproducible processing of TCGA regulatory networks,” that we are resubmitting to GigaScience as a Technical Note.<br>In this manuscript we describe, tcga-data-nf, a robust, highly configurable Nextflow workflow that allows users to reproducibly infer gene regulatory networks for the thousands of samples in The Cancer Genome Atlas (TCGA) and that can easily be adapted to working with other large datasets.<br>Multiomic and gene regulatory network (GRN) modeling has been shown to provide insight into the drivers of health and disease phenotypes. Our research group has long been a leader in developing methods for inferring and analyzing GRNs; our collected tools, “The Network Zoo” (NetZoo), including DRAGON, PANDA, LIONESS, and ALPACA, have been cited more than 700 times. Given our interest in understanding common features among various cancer types, we undertook a project to infer GRNs in each sample and each cancer type represented in TCGA. Because consistency in the inference of so many networks was essential if we were to compare them, we recognized we needed to establish a uniform, validated workflow in which software tools were chained in a logical and justifiable order and that could be easily used by individuals with limited experience in GRN inference and analysis. In this manuscript we detail the structure and implementation details of the pipeline and then demonstrate how it can be used to identify differences between colon cancer subtypes.<br>Following the reviewers’ requests we have now implemented the following substantial changes to the pipeline and the manuscript:<br>-<br>We added DRAGON for joint analysis of CNV-expression data and ALPACA for comparison of PANDA networks. We also included these tools in our analysis of the regulatory changes that distinguish colon cancer subtypes.<br>-<br>We documented how to add network inference methods (GENIE3 and WGCNA) that were developed by other groups, increasing the overall impact of our work.<br>-<br>We improved and extended the “cross-validation” evidence of quality and usefulness of the networks.<br>-<br>We now provide details on the computational performance of the pipeline.<br>-<br>We addressed all minor issues raised by the reviewers, both in the code and the manuscript.<br>We believe that we have addressed all reviewer’s concerns and correctly edited the manuscript to meet the journal’s standards. A more detailed accounting of the changes we have made can be found in the response letter attached to this submission. As you requested, we have now registered the tools on WorkflowHub, bio.tools, and scicrunch.<br>As always, if you have any questions or if we can provide any additional information to

Powered by Editorial Manager® and ProduXion Manager® from Aries Systems Corporation

assist you in your decision, please let us know.
On behalf of my co-authors and colleagues, I offer you my best,
John Quackenbush, Ph.D.
Henry Pickering Walcott Professor of Computational Biology and Bioinformatics

Response to Reviewers
We would like to thank the editor and the reviewers for their comments and guidance. We have now extended the pipeline, improved the NetworkDataCompanion package, and revised the manuscript accordingly. Hopefully, you will find the presentation in the manuscript to be clearer and the pipeline more complete. We have also revised the colon cancer subtype analysis, adding more results obtained by using multiple omics and our group's state-of-the-art network analysis tools (DRAGON, ALPACA) to identify key regulatory differences between the CMS2 and CMS4 colon cancer subtypes. Following the editor's guidance, we have now registered the workflow on WorkflowHub (SEEK ID: https://workflowhub.eu/workflows/1306?version=1), and NDC on bio.tools (https://bio.tools/NetworkDataCompanion) and scicrunch (RRID:SCR_026532)
Moreover, point-by-point responses (in blue) to all points raised by the reviewers can be found below, together with the original questions/comments.
Reviewer #1:
Fanfani et al. present tcga-data-nf, a Nextflow pipeline that streamlines the download, preprocessing, and network inference of TCGA bulk data (gene expression and DNA methylation). Alongside this pipeline, they introduce NetworkDataCompanion (NDC), an R package designed to unify tasks such as sample filtering, identifier mapping, and normalization. By leveraging modern workflow tools—Nextflow, Docker, and conda—they aim to provide a platform that is both reproducible and transparent. The authors illustrate the pipeline's utility with a colon cancer subtype example, showing how multi-omics networks (inferred via PANDA, DRAGON, and LIONESS) may help pinpoint epigenetic factors underlying more aggressive tumor phenotypes. Overall, this work addresses a clear need for standardized approaches in large-scale cancer bioinformatics.
Major
While tcga-data-nf promises a valuable resource, the following issues should be addressed more thoroughly before publication:
1. While PANDA, DRAGON, and LIONESS form a cohesive system, they were all developed by the same research group. To strengthen confidence, please include head-to-head comparisons with other GRN inference methods (e.g., ARACNe, GENIE3, Inferelator)**. A small benchmark dataset with known ground-truth (or partial experimental validation) would be especially valuable.
We thank the reviewer for the suggestion. While we had organized the workflow to be modular and extensible beyond the netZoo tools, we agree that we had not shown how that could have been done. The goal of this workflow is to enable researchers to generate regulatory networks from cancer datasets and so, as you suggested, we have now added GENIE3 and WGCNA to the pipeline, together with comprehensive documentation on how to add tools to the workflow (add link.XXX). However, we believe that benchmarking the quality of the resulting networks is beyond the scope of our work.
First, the methods are all published and have been benchmarked against various others and the results have been reported in the literature (https://journals.plos.org/plosone/article?id=10.1371/journal.pone.0064832 , https://doi.org/10.1371/journal.pone.0064832, https://doi.org/10.1016/j.isci.2019.03.021, https://academic.oup.com/nar/article/51/3/e15/6931867, https://www.nature.com/articles/s41540-018-0052-5). Second, any new method for GRN inference would require its own peer-reviewed manuscript to prove the correctness and relevance of the output networks. Third, many of these methods are based on different assumptions about networks and produce outputs that differ in structure and require nuanced interpretations. We believe that enabling broader application of existing methods is valuable beyond choosing the "best performing" tool, which itself can generally only be determined in a context-specific manner.
Although the manuscript identifies intriguing TFs and pathways, it lacks confirmation through orthogonal data or experiments. If available, consider including ChIP-seq or CRISPR-based evidence to reinforce at least a subset of inferred regulatory interactions. Even an in silico overlap with known TF-binding sites or curated gene sets

Powered by Editorial Manager® and ProduXion Manager® from Aries Systems Corporation

would help validate the predictions.
We thank the reviewer for the comment and we have now added more controls on the quality of the inferred networks. For DRAGON edges we show the correlation between our predictions and StringDB co-expression edges, we show some exploratory statistics on the networks to confirm the validity of the predictions (promoter methylation and expression are negatively correlated, while CNV and expression on the same gene are positively correlated) and we have found both methylation-expression and CNV-expression evidence of TFs involvement in cancer progression. Moreover, we have added the ALPACA tool for comparing different PANDA GRNs, which allows identification of differential modules between CMS2 and CMS4. Membership values in those modules allowed us to prioritize TFs assigned to them by PANDA; many of the TFs that we identified using this approach have experimental evidence of a role in colorectal cancer risk and progression. Unfortunately, as these are subtype specific networks we were unable to retrieve orthogonal ChIP-seq or CRISPR-based matched to the “tissue” or subtype, but, we would like to reiterate that these methods have been extensively used, and published work from other labs have used our tools to identify a new TF in RA and have experimentally validated the results (https://www.life-science-alliance.org/content/8/1/e202402808)
1. PANDA and DRAGON emphasize correlation/partial correlation, so they may overlook nonlinear or combinatorial regulation. If feasible, please provide any preliminary steps taken to capture nonlinearities or discuss approaches that could be integrated into the pipeline.
We thank the reviewer for this point. First, we would point out that PANDA includes the effect of transcription factor complexes (PPIs) in its model, which could partially capture combinatorial regulation. Also, we have seen in practice that the appropriate choice of the priors can improve prioritization of TF-gene regulation, and they can be updated to include more complex
regulation mechanisms. To some extent, DRAGON has the potential to capture non-linear or combinatorial regulation in the sense that Gaussian graphical models (partial correlation networks) have been shown to capture known metabolic reactions involving non-linear network motifs such as feedback loops (Krumsiek et al. 2011 https://link.springer.com/article/10.1186/1752-0509-5-21). This is an advantage over correlation-based networks, as Krumsiek et al. demonstrate, and is likely due to the fact that partial correlations between two nodes involve conditioning on the states of the other nodes in the network, while correlations are simply marginal calculations between the two nodes and thus capture both direct and indirect dependence (Shutta et al. 2022 https://doi.org/10.1002/sim.9546)
Nonetheless, we recognize that nonlinearities are harder to manage with our existing tools. For that reason, we would recommend the user to extend the workflow to non-linear methods following the steps at https://github.com/QuackenbushLab/tcga-data-nf/blob/main/extend_workflow.md
1. LIONESS reconstructs a network for each sample in a leave-one-out manner, which can be demanding for large cohorts. **The paper does not mention runtime or memory requirements. Adding a Methods subsection with approximate CPU/memory benchmark**s (e.g., "On an HPC cluster with X cores, building LIONESS networks for 500 samples took Y hours") is recommended to guide prospective users.
We thank the reviewer for the helpful suggestion. We have now added the section “Computational performance” with details on the minimal requirements to run the workflow tests and LIONESS. We have also added the details to the workflow itself, such that users can be guided in the choice of the machine they ultimately choose to use for their analysis.
Currently, the pipeline only covers promoter methylation and standard gene expression, yet TCGA and related projects include other data types (e.g., miRNA, proteomics, histone modifications)**. If possible, offer a brief example or instructions on adding new omics layers,** even conceptually.
We thank the reviewer for the comment. First, we need to acknowledge that the pipeline already includes the download and pre-processing of mutation and CNV data, even though these were not used by the GRN inference methods. However, we adopted this suggestion and enabled the users to run DRAGON networks with CNV data. We have slightly modified the workflow – the metadata and output filename format – to infer DRAGON networks with any omics. For this paper, we have computed CNV-expression networks for the colon cancer subtypes and added the results to the “Multiomics associations identify changes between colon subtypes” section.

Recent methods often target single-cell RNA-seq, but tcga-data-nf is geared toward bulk datasets. **Please clarify limitations and potential extensions for single-cell or multi-region tumor data.** This would help readers understand whether (and how) the pipeline could be adapted to newer high-resolution profiles.
We thank the reviewer for the suggestion. First, we have included more examples on how the workflow can be used and extended to download other GDC-based data (even single cell data)
and how it can be instead used with different datasets. Nonetheless, we have now modified the "Discussion" section to recognize that this specific workflow is designed for enabling seamless processing of bulk tumor data from TCGA, and that scRNAseq or other large-scale projects would benefit from dedicated pipelines.
Minor point:
1. Provide clear guidance on cutoffs for low-expressed genes, outlier samples, and methylation missing-value imputation.
2. Consider expanding the supplement with a "quick-start" guide, offering step-by-step usage examples.
3. Ensure stable version tagging in your GitHub repository so that readers can reproduce the exact pipeline described in the manuscript.
We thank the reviewer for the suggestions. We have expanded the documentation to include clearer guidance on parameters and we have added the quickstart guide to the supplement so that readers have an overview of the online resources. We have now switched to a more stable tagging of the versions.
Reviewer 2:
This manuscript presents tcga-data-nf, a Nextflow-based pipeline for downloading, preprocessing, and analyzing TCGA multi-omic data, with a focus on gene regulatory network (GRN) inference. The workflow integrates established bioinformatics tools (PANDA, DRAGON, and LIONESS) and adheres to best practices for reproducibility through containerization (Docker, Conda, and Nextflow profiles). The authors demonstrate the utility of their pipeline by applying it to colorectal cancer subtypes, identifying potential regulatory interactions in TGF-β signaling.
The manuscript is well-written and well-structured and provides sufficient methodological details, as well as Jupyter notebooks, for reproducibility. However, there are some areas that require clarification and improvement for acceptance in GigaScience, particularly regarding the scope of the tool, the quality of the inferred regulatory networks, the case study figure, benchmarking, statistical validation, and parameters.
Major comments:
While the pipeline is well designed and executed, the overall impact of the tool feels somewhat limited, especially for a journal like GigaScience, due to its pretty specific application to building GRNs in TCGAs, the relatively small number of parameters, the support of only 2 omics type, and the lack of novel algorithms.
We agree with the reviewer that the scope of GRNS in TCGA, is of course, somewhat limited. Below, we detail how we have extended the pipeline to include more omics types and methods, such that even within its boundaries, tcga-data-nf is broader and more flexible. We would also like to draw the reviewers' attention to the flexibility of NDC, which extends beyond the functions that are directly used in the pipeline. Moreover, based on the wealth of information encapsulated in TCGA data that has yet to be analyzed with network models, we believe this pipeline will serve to provide meaningful biological insights that are relevant for the broader cancer research community.
To increase the impact of this tool I would recommend **adding functionalities**, such as:
- **Supporting additional tools.** A great strength of the pipeline is the integration with the Network Zoo (NetZoo) ecosystem. However, only three tools are included from NetZoo. Including additional tools would likely increase the scope of users interested in using the pipeline. In particular, an important weakness of the current pipeline is that it is not possible to conduct differential analysis between different networks, which prevents users from identifying the most significant differences between two networks of interest (e.g., CMS2 vs CMS4). **The NetZoo contains different tools to conduct such analyses, such as Alpaca 1 or Crane 2,** thus this may be implemented to make the pipeline more useful to a broader user base.
We thank the reviewer for this suggestion and we agree that expanding the pipeline to include other methods would be useful. We have now added ALPACA for differential network analysis, and have incluced a demonstration of its application to the CMS2 vs

Powered by Editorial Manager® and ProduXion Manager® from Aries Systems Corporation

	CMS4 question. Together, we have modified the Results section (subsections: "Analyze", "Full", " Multi-omic partial correlation networks identify differences between colon subtypes") to reflect these changes in the pipeline. We also added CNV-expression DRAGON networks to the pipeline, which are useful to estimate complex effects of DNA aberrations.<br>**o Adding parameters.** A strength of the pipeline is the ability to customize it using various parameters. However, as such the pipeline does not offer many parameters. It would be beneficial to make the pipeline a bit more customizable. For example, novel parameters could be: adding options for excluding selected samples, using different batch correction methods, different methods to map CpGs to genes, additional normalization methods, and additional quality controls (e.g., PCA for methylation samples, md5sum checks). These are just examples and do not need to be all implemented but adding some extra parameters would help make the pipeline more appealing and customizable to various users.<br>We also thank the reviewer for this suggestion. As explained in the discussion of the paper, the workflow attempts to balance flexibility (we allow the user to specify many parameters) and ease-of-use, so that even moderately experienced users can run GRN inference with relatively little effort and a shallow learning curve but still achieve meaningful results. However, as<br>requested, we have added some features that we thought could benefit the workflow; drawing PCA plots for methylation, filtering duplicate methylation samples based on minimum missing data and parameters (e.g., choice of TCGA tumor purity method, choice of threshold for missingness to exclude vs. impute methylation data). Also, we plan to add more as needed based on user requests or changes in the methods.<br>- **The quality of the inferred regulatory networks is hard to judge.** There are no direct comparisons with any other tools. For instance, it is mentioned in the text that GRAND networks were derived using a fixed set of parameters, but it could be helpful to show a direct comparison between GRNs built from your tools with those from GRAND. This could reveal how the ability to customize GRNs using the pipeline's parameters helps in getting better biological insights.<br>o Alternatively, or in addition, one **could compare how networks built by your method fare in comparison to networks** built from other methods, like RegEnrich 3 or NetSeekR 4, in terms of biological insights, accuracy, scalability, speed, functionalities and/or memory usage.<br>We thank the reviewer for this comment. First, upon re-reading our conclusions we realized that we had not explained our conclusions regarding GRAND. We have now modified the paragraph to explain how GRAND is a dynamic database where published work is stored and accessible, while emphasizing that each user may want to carry out their own individual analyses like we did in this paper. Additionally, since the goal of this workflow is to enable researchers to easily generate regulatory networks from cancer datasets, we have added GENIE3 and WGCNA to the pipeline and have included comprehensive documentation on how users can add their choice of tools to the workflow.<br>With regard to judging the results, we do not believe this is necessary, as all of the methods included in the workflow have have been published and widely benchmarked in the literature ((<br>https://journals.plos.org/plosone/article?id=10.1371/journal.pone.0064832 ,<br>https://doi.org/10.1371/journal.pone.0064832,<br>https://doi.org/10.1016/j.isci.2019.03.021,<br>https://academic.oup.com/nar/article/51/3/e15/6931867,<br>https://www.nature.com/articles/s41540-018-0052-5).); our intention is to ease the use of these methods in a combined workflow. We do agree that any novel method for GRN inference or benchmarking would require its own peer-reviewed manuscript to prove the fidelity and quality of the output networks, but that is not our goal in implementing this workflow.<br>o Another angle to judge the regulatory networks would be to check in a case study if the predicted gene interactions between disease and control networks are enriched in disease and gene-gene interactions databases, such as DisGeNet 5.<br>We thank the reviewer for this idea. We have now added evidence of the relevance of the DRAGON edges we found. We now report (1) negative correlation between methylation and expression on the same TF, (2) positive correlation between CNV and expression, (3), that co-expression network (expression-expression edges) are supported by evidence from the STRINGDB database, (4), edges in cluster A of the DRAGON methylation-expression networks are enriched for TFs that are involved in

Colorectal Neoplasms according to the DisGeNet
gene-disease association lists. Please note that we have used the original release of DisGeNet in the analysis reported here:
https://academic.oup.com/database/article/doi/10.1093/database/baaf007/7994270
* Figure 2 needs re-work:
o Panel A and C: text is too small. "tf" should be written TF. "oi" should have another name. These panels might be moved to the supplements.
o Panel D is confusing. Without significance it is hard to understand what the point of this panel is. I can see that certain TFs are cited in the main text but without information about significance, these may seem like cherry-picking. The legends states: `Annotation of all TFs in cluster D (columns) to the Reactome parent term. "Immune system" and "Cellular respondes to stimuli" are more consistenly involved in cluster D, in comparison to cluster A.`. However, this is a key result which should be shown in a main figure, not in Figure S6. I would also recommend using a -log scale when displaying the p-values to highlight the most significant entries.
o Panel E is quite confusing; first, the color coding is unclear. For instance, what represents blue, purple and red colors? Second, what represents the edges' widths? I would recommend using different shapes for the methylation and expression nodes to reduce the number of colors, and adding a color legend. I would also consider merging the two graphs and representing in color the difference in the edge values so the reader can directly see the key differences.
We thank the reviewer for the detailed comments and have updated the figure accordingly. After careful consideration, we decided to keep the figure in panel C and update the text to better explain what it represents. Indeed, we only have a small number of TFs and we are interested in showing the general functional annotation terms they represent. These TFs were not preselected or chosen in a biased fashion, but instead encompass all of those in cluster D that were annotated to at least one pathway. Indeed, functional enrichment analysis on TFs is usually more “difficult” as the power is lower (give the total number of TFs) and they are all annotated to the regulatory terms, skewing the adjusted p-value. Nonetheless, it is interesting to see that these TFs present meaningful biological insights.
* Benchmarking analysis could be included to show the runtime and memory requirement for each pipeline step. It would also be beneficial to analyze a larger dataset than colon cancer to assess the scalability.
We thank the reviewer for the helpful suggestion. We have now added the section “Computational performance” with details on the minimal requirements to run the workflow tests and LIONESS. We have also added the details to the workflow itself, such that users can be guided in the choice of the machine.
* Statistical analysis: If computationally feasible, permutation testing could be implemented to quantify the robustness of inferred regulatory interactions. Also, in the method section, it should be clarified that FDR correction was applied for pathway enrichment analysis.
We thank the reviewer for these points. We have now revised and expanded the methods section. Regarding robustness, we agree that this is a significant question and that additional methods to quantify robustness would be useful. However, we also note that before showing statistical significance, one should investigate how to properly randomize data for each of these methods, including to provide good empirical estimates of the confidence intervals, or analytical work should be done to infer closed form solutions for the confidence interval; all of this would require its own detailed analysis and is beyond the scope of our current work.
Minor comments:
* I am not sure why duplicate samples are discarded in the pipeline. Why not add counts for RNA-Seq and averaging beta values? I would expect that to yield more robust results.
We thank the reviewer for the comment. From visually inspecting some of the data we had noticed that some of the duplicated samples, with lower depth, seemed very sparse. NDC does not support combining duplicated samples as there may be important technical differences that should not be masked (for example, contamination of a vial or differences in bulk tissue cell type composition). However, we have extended the options for selecting the duplicate based on missingness in methylation data (i.e., select the sample with the least overall missingness).
* It is a bit unclear in what context the NetworkDataCompanion tool could be used outside the workflow. It is also unclear how it helps with quality controls. Please clarify these aspects.

	We thank the reviewer for this point. We have now included a quickstart and documentation for NetworkDataCompanion such the barrier to use is lower. The quickstart is provided as an RMarkdown file (https://github.com/QuackenbushLab/NetworkDataCompanion/blob/main/quickstart.Rmd) so that users can download and run it directly. The rendered HTML can also be viewed at https://htmlpreview.github.io/?https://github.com/QuackenbushLab/NetworkDataCompanion/blob/main/quickstart.html.<br>In addition, we wish to highlight that while some of NDC's functions are specifically designed for wrangling TCGA data, many can be broadly applied to any set of gene expression data that is stored in RangedSummarizedExperiment objects and to any matrix of methylation beta values with an accompanying manifest of the appropriate format. We believe that the impact of NDC is therefore broadly relevant to the readers of GigaScience<br>There are two key aspects of NetworkDataCompanion that ensure quality control in the Prepare step of the pipeline. First, NetworkDataCompanion incorporates unit tests for every function. These tests are toy examples where the function of the code is checked by asserting equality between results calculated by NetworkDataCompanion and "gold standard" results calculated manually. The tests are built with every addition to the package via continuous integration through GitHub actions, so if a contributor makes changes to the code, it is automatically<br>checked that there are no problems caused to the existing functions. Second, NetworkDataCompanion is version-controlled via GitHub. This facilitates reproducibility of tcga-data-nf workflows: as NetworkDataCompanion evolves, users can document which version of the repository they have used in their analysis and that exact code will continue to be accessible via GitHub's versioning scheme.<br>* The manuscript is well-written, but words are sometimes missing or wrongly written, it needs careful re-read.<br>We thank the reviewer for pointing this out. We have gone through the paper and edited it to address missing words and incorrect word choice.<br>* The expression '"same-same"' is unclear to me. In this sentence: "Some of "same-same" genes (STAT5A, CREB3L1"…, I am not sure in which table or figure I can find this result?<br>We apologize for the colloquial language and have revised the expression and rephrased the paragraph. Also, we have added Supplementary Figure S6 that summarizes these results.<br>* Text is too small in the Directed Acyclic Graph, especially in Figure S4. Also, I would recommend adding the Directed Acyclic Graphs from Figure S1-S4 to the online documentation.<br>We thank the reviewer for the comment, and have now fixed all figures and added them to online documentation. Since we were not able to produce a Figure S4 with visible names, we opted for keeping it only in the online documentation, while we made figures S1-S3 larger.<br>* Regarding the code, I was puzzled to see a copyConfigFiles process. Also, there are files in `bin/r/local_assets`, these should be located in `assets`. And the container for the singularity and docker profile is likely the same, this should be clarified in the code.<br>We thank the reviewer for this suggestion and we have now moved the local assets. Regarding copyConfigFiles. In practice, we have noticed that manually copying all configuration files into the output folder is helpful for reproducibility, especially for less experienced users.<br>* It is recommended to remove the "defaults" channel from the list of channels declared in the `containers/conda_envs/analysis.yml` file. Please see information about that here[https://www.anaconda.com/blog/is-conda-free] (https://www.anaconda.com/blog/is-conda-free) and here[https://www.theregister.com/2024/08/08/anaconda_puts_the_squeeze_on/] (https://www.theregister.com/2024/08/08/anaconda_puts_the_squeeze_on/).<br>We thank the reviewer for this suggestion! This was part of the reason we moved the containers away from using conda, but we have now followed their suggestions and removed the default channel from the environment's yaml file.<br>Additional comments (which do not need to be addressed):<br>* Future work may consider enabling the use of the pipeline to build GRNs from other data sources than TCGA (i.e., nf-netzoo). Recount3 data is already being parsed for GTEx and TCGA samples, so it might be relatively easy to adapt the pipeline so that it can be used on any arbitrary recount3 dataset. Similarly, it could be useful if one could

Powered by Editorial Manager® and ProduXion Manager® from Aries Systems Corporation

	specify a dataset on the recountmethylation database 6 to build GRNs. While these unimodal datasets could not be used with the DRAGON method they would still benefit from all other features of the pipeline.<br>We are indeed considering a method-centric pipeline that would be independent of TCGA, but that needs to be addressed separately. These are all great suggestions that we'll definitely consider as we continue to build and refine the pipeline!<br>* Using a nf-core template would enable better structure of the code and increase the visibility of the tool. Also using multiple containers is usually easier to maintain and update than a single large container, especially when a single tool needs to be updated or when modifying part of the pipeline. Another comment is that the code contains many comments which are not to explain the code but more like quick draft which makes the code harder to read by others.<br>We had thought about adding it to nf-core, but their guidelines were not compatible with the development of this workflow. We'll surely consider it for future projects. We have also added more containers, specific for each step, and reviewed the comments, thanks for this suggestion!<br>References<br>1. Padi, M., and Quackenbush, J. (2018). Detecting phenotype-driven transitions in regulatory network structure. npj Syst Biol Appl 4, 1-12. https://doi.org/10.1038/s41540-018-0052-5.<br>2. Lim, J.T., Chen, C., Grant, A.D., and Padi, M. (2021). Generating Ensembles of Gene Regulatory Networks to Assess Robustness of Disease Modules. Front. Genet. 11.https://doi.org/10.3389/fgene.2020.603264.<br>3. Tao, W., Radstake, T.R.D.J., and Pandit, A. (2022). RegEnrich gene regulator enrichment analysis reveals a key role of the ETS transcription factor family in interferon signaling. Commun Biol 5, 1-12. https://doi.org/10.1038/s42003-021-02991-5.<br>4. Srivastava, H., Ferrell, D., and Popescu, G.V. (2022). NetSeekR: a network analysis pipeline for RNA-Seq time series data. BMC Bioinformatics 23, 54. https://doi.org/10.1186/s12859-021-04554-1.<br>5. Hu, Y., Guo, X., Yun, Y., Lu, L., Huang, X., and Jia, S. (2025). DisGeNet: a disease-centric interaction database among diseases and various associated genes. Database 2025, baae122.https://doi.org/10.1093/database/baae122.<br>6. Maden, S.K., Walsh, B., Ellrott, K., Hansen, K.D., Thompson, R.F., and Nellore, A. (2023). recountmethylation enables flexible analysis of public blood DNA methylation array data. Bioinformatics Advances 3, vbad020. https://doi.org/10.1093/bioadv/vbad020.
**Additional Information:**	
**Question**	**Response**
Are you submitting this manuscript to a special series or article collection?	No
**Experimental design and statistics**<br><br>Full details of the experimental design and statistical methods used should be given in the Methods section, as detailed in our Minimum Standards Reporting Checklist. Information essential to interpreting the data presented should be made available in the figure legends.	Yes

Have you included all the information requested in your manuscript?	
**Resources**<br><br>A description of all resources used, including antibodies, cell lines, animals and software tools, with enough information to allow them to be uniquely identified, should be included in the Methods section. Authors are strongly encouraged to cite Research Resource Identifiers (RRIDs) for antibodies, model organisms and tools, where possible.<br><br>Have you included the information requested as detailed in our Minimum Standards Reporting Checklist?	Yes
**Availability of data and materials**<br><br>All datasets and code on which the conclusions of the paper rely must be either included in your submission or deposited in publicly available repositories (where available and ethically appropriate), referencing such data using a unique identifier in the references and in the "Availability of Data and Materials" section of your manuscript.<br><br>Have you have met the above requirement as detailed in our Minimum Standards Reporting Checklist?	Yes
GigaScience has policies and guidelines in place for the use of generative AI-writing tools such as ChatGPT. If you have used such writing tools to assist with writing the manuscript this must be declared and cited in the text. Authors should not list AI-writing tools and other AI-assisted technologies as an author or co-author and should acknowledge that they are fully responsible for text generated or refined by AI-writing tools.<p><br><br>A summary of use (particularly in the introduction or among methods) needs to	No

be included at the end of the paper, and the outputs should also be included as a supplementary file hosted in GigaDB or other open repositories. Please read our guidelines for more information. <p><br><br>By submitting to GigaScience, you are aware of the journal's AI-writing tools policy, and if you have declared use of such tools below, you have acknowledged this where appropriate in your manuscript and have made a summary of use and outputs available. <p><br><b>AI-assisted writing tools have been used in the preparation of this manuscript?	

Powered by Editorial Manager® and ProduXion Manager® from Aries Systems Corporation

Revised Manuscript

Click here to access/download;Manuscript;tcga_processing_manuscript_revi

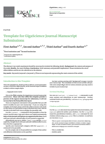

GigaScience, 2023, 1–S34

doi: xx.xxxx/xxxx
Manuscript in Preparation
Paper

PAPER

Reproducible processing of TCGA regulatory networks

Viola Fanfani[1], Katherine H. Shutta[1,2], Panagiotis Mandros[1], Jonas Fischer[1], Enakshi Saha[1], Soel Micheletti[1], Chen Chen[1], Marouen Ben Guebila[1], Camila M. Lopes-Ramos[1,2,3] and John Quackenbush[1,2]

[1]Department of Biostatistics, Harvard T.H. Chan School of Public Health, Boston, MA, USA and [2]Channing Division of Network Medicine, Brigham and Women's Hospital, Boston, MA, USA and [3]Department of Medicine, Harvard Medical School, Boston, MA, USA

[*]Corresponding Author, johnq@hsph.harvard.edu

Abstract

Background. Technological advances in sequencing and computation have allowed deep exploration of the molecular basis of diseases. Biological networks have proven to be a useful framework for interrogating omics data and modeling regulatory gene and protein interactions. Large collaborative projects, such as The Cancer Genome Atlas (TCGA), have provided a rich resource for building and validating new computational methods resulting in a plethora of open-source software for downloading, pre-processing, and analyzing those data. However, for an end-to-end analysis of regulatory networks, a coherent and reusable workflow is essential to integrate all relevant packages into a robust pipeline.
Findings. We developed tcga-data-nf, a Nextflow workflow that allows users to reproducibly infer regulatory networks from the thousands of samples in TCGA using a single command. The workflow can be divided into three main steps: multi-omics data, such as RNA-seq and methylation, are (i) downloaded, (ii) pre-processed, and (iii) analyzed to infer regulatory network models with the Network Zoo. The workflow is powered by the NetworkDataCompanion R package, a standalone collection of functions for managing, mapping, and filtering TCGA data. Here, we demonstrate how the pipeline can be used to study the differences between colon cancer subtypes that could be explained by epigenetic mechanisms. Lastly, we provide a database of pre-generated networks for the 10 most common cancer types that can be readily accessed by the public.
Conclusions. tcga-data-nf is a complete yet flexible and extensible framework that enables the reproducible inference and analysis of cancer regulatory networks, bridging a gap in the current universe of software tools for analyzing TCGA data.

Key words: "Gene Regulatory Network"; "The Cancer Genome Atlas"; "Cancer"; "Nextflow"; "NetworkDataCompanion"; "reproducibility" (3 to 10 keywords)

Background

There is a growing recognition of the importance of ensuring that scientific research is reproducible and that analytical steps are transparent [1, 2]. The field of bioinformatics has been particularly receptive to this trend, with many prominent scientists and journals advocating for the use of open-source software, open data, and reproducible methods [3, 4]. Projects such as Bioconductor [5] and Bioconda [6] facilitate sharing and reusing bioinformatics software. The Galaxy project [7] has pioneered the development of platforms for training and sharing best practices for complete data analysis workflows. Workflow management tools such as Nextflow [8], Snakemake [9], and WDL [10] facilitate reproducibility of complex data analysis pipelines.

These methodological advances are intertwined with the increasing availability of large-scale biological data. The falling cost of sequencing has enabled the generation of population-level omics data; thousands of subjects can be profiled in a single project to investigate complex traits and diseases. As notable examples, the UKBiobank [11] has collected multi-omics and clinical data for more

Compiled on: May 7, 2025.
Draft manuscript prepared by the author.

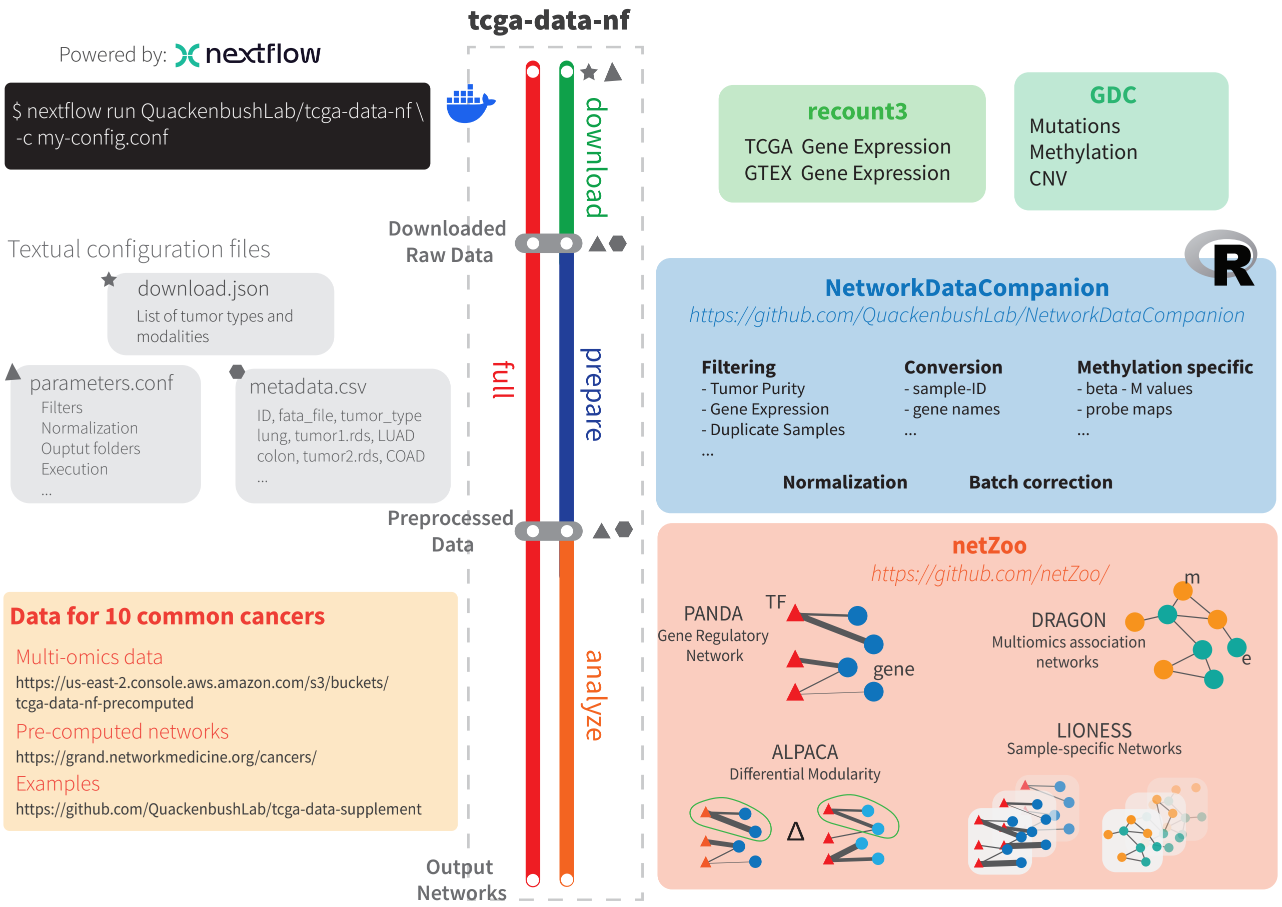

Figure 1. Graphical Abstract

than 500, 000 individuals representative of the UK population, and the 1000 Genomes Project [12] includes samples from more than 4, 000 individuals collected to characterize human genetic variation. Ongoing data collection efforts (such as the 100, 000 genome project [13]) demonstrate that this deluge of data is not slowing down. With the consequent massive scope for downstream analyses comes the need for reproducible and strictly managed software pipelines to work with these data.

The Cancer Genome Atlas (TCGA) [14] was one of the first large collaborative projects designed to study the molecular basis of disease and includes samples collected from more than 10, 000 cancer patients representing over 30 tumor types. TCGA data have been invaluable for the study of regulation in both healthy and tumor tissues[15, 16, 17, 18, 19] and for developing and benchmarking analytical methods for omics data [20, 21]. The TCGA dataset has grown in value with data from related projects such as The Cancer Protein Atlas (TCPA) [22, 23] and the Clinical Proteomic Tumor Atlas Consortium (CPTAC) [24, 25], which create new opportunities for innovative methods development and applications.

Given that most pathologies are the result of the complex interplay between multiple genomic, transcriptomic, epigenomic and other factors [26, 27], inference and analysis of network models integrating multiple omics is possibly the most important analytical trend enabled by large-scale omic data resources. Biological networks represent interactions between biological entities to model high-level organization of biological systems and they aim to describe the molecular mechanisms that define biological states and the progression between them. Most notably, network analyses have helped to elucidate the etiology and progression of tumors and provide insight into important features of their clinical manifestation[28, 29, 30, 31, 32]. Many network types have contributed to our understanding of biological processes, including protein-protein interaction networks, networks of DNA-protein interactions, and co-expression networks. Among them, gene regulatory networks (GRNs) consisting of transcription factors (TFs) and the genes they target for regulation in a particular phenotype have emerged as a particularly powerful tool to describe the complex machinery driving disease development, progression, and response to therapy [33, 34, 35, 36, 37, 38]. Expanding on the idea of characterizing regulation by modeling the interactions between TFs and genes, multi-omic association networks combine multi-modal data to shed light on other factors that can influence regulation. For example, combining epigenetic and transcriptomic data [39] can provide insights into links between observed gene expression patterns and DNA methylation, potentially controlled by a proximal or distal element. For these and other applications, the TCGA dataset is uniquely positioned to be analyzed using network methods including single and multi-omic networks, gene regulatory networks, and networks modeling the relationship between somatic aberrations and their effects on regulation.

However, accessing and analyzing, from raw data to networks, the wealth of data that TCGA or similar projects is a non-trivial task. For example, raw sequencing data cannot be publicly released and must be aligned and quantified prior to downstream analyses. Integration of multi-omic data requires matching of samples across assays, and each omic has its own set of data pre-processing and filtering steps that are fundamental to the quality of the analysis (e.g., removing samples that are of low quality, handling batch effects). The Genomic Data Commons (GDC) provides an application programming interface (API) [40, 41] that interfaces with R via the TCGAbiolinks R package [42, 43]. TCGAbiolinks provides both programmable access to the TCGA data and tools for data wrangling

tasks such as identifier mapping and data filtering based on clinical features. For example, with a few lines of code, one could download all RNA-seq and mutation data for a specific tumor (or subtype) from those subjects over 70 years of age. The TCGAbiolinks package also provides a number of functions for *ad-hoc* pre-processing of the data, on top of the most commonly used analytical steps such as differential gene expression analysis [44, 43].

Once the data are downloaded and pre-processed, additional analyses can be performed to extract biologically meaningful insights. The Network Zoo project (netZoo; `https://netzoo.github.io/`) is a growing open-source suite of tools for the inference and analysis of biological networks [45]. The 16 methods currently in netZoo include PANDA [33], which uses gene expression data together with prior TF-binding information and TF-TF interaction data to infers Gene Regulatory Networks (GRNs) describing TF-gene interactions, ALPACA [46], that compares two PANDA's network structure, DRAGON [39], which creates robust multi-omic Gaussian graphical models (partial correlation networks), and LIONESS [47], which estimates individual networks for each sample in a population by using a leave-one-out strategy with linear interpolation.

It is clear that a full analytical pipeline for TCGA or similar data involves a range of data access, pre-processing, and analysis steps. Although there are reusable and documented pieces of software for each specific step, as detailed above, chaining these processes back to back from data download to final analysis yields a complex workflow with many interdependent processing decisions. Moreover, tools to facilitate the transfer of data and results from one step to the next are required. As with any programmatic workflow, each step introduces additional possibilities for issues with accuracy and reproducibility. Given the number of steps involved in network analysis of TCGA data in particular, we propose that a single robust and transparent workflow is an essential tool for conducting accurate, reproducible analyses yielding meaningful results.

To this end, we developed *tcga-data-nf* (`https://github.com/QuackenbushLab/tcga-data-nf`), a Nextflow workflow to generate network models of TCGA data with a single command that manages all the steps from data download through pre-processing to network generation. *tcga-data-nf* is structured around three key workflow modules: Download , Prepare , and Analyze . First, in the Download module, *tcga-data-nf* facilitates the download of TCGA clinical and phenotypic data as well as several omics modalities including RNA-seq, mutation, methylation and copy number variation (CNV) data. The Prepare module involves pre-processing steps specific to each datatype. Finally, the Analyze module generates individual sample GRNs and expression-methylation/CNV association networks by combining LIONESS with PANDA and DRAGON, respectively. In a detailed example, we show that *tcga-data-nf* not only allows us to swiftly generate networks for the four consensus subtypes of colon cancer [48], but also we demonstrate the use of *tcga-data-nf* to expand our understanding of the regulatory processes driving subtype-specific prognosis. In particular, we find molecular evidence for epigenetic involvement in the more aggressive CMS4 subtype.

As part of developing *tcga-data-nf*, we created the *NetworkDataCompanion* (*NDC*, `https://github.com/QuackenbushLab/NetworkDataCompanion`). *NDC* streamlines routine steps in TCGA data processing, including filtering and mapping gene and sample identifiers between modalities (which is often a challenge with such heterogeneous data) and modality-specific data transformation, such as normalization and cleaning. While *NDC* was designed to provide necessary back-end functions for *tcga-data-nf*, it also serves as a standalone tool for separate use.

To provide users with a seamless out-of-the-box experience, *tcga-data-nf* comes equipped with all essential supplementary components, including a Docker container, conda environments, comprehensive documentation, and introductory tutorials to help users get started. As an additional illustrative example and resource for the public, we have applied *tcga-data-nf* to generate GRNs for the ten most frequent cancer types (BRCA, LUAD, LUSC, KIRC, LIHC, PAAD, PRAD, SKCM, STAD, COAD) and published these networks on the Gene Regulatory Network Database (GRAND) [49] (version 1.7) for easy access and exploration.

Findings

The open-source *tcga-data-nf* is a Nextflow [8] workflow that allows users to fully execute network analysis on TCGA data in a single, end-to-end pipeline spanning the data download, preparation, and network inference. By chaining and combining atomic tasks, called processes, this pipeline enables users to infer GRNs and other networks with a single command. Tissues of interest (e.g., BRCA, COAD) and workflow parameters are specified by the user in configuration files called by the workflow.

The whole workflow consists of three main steps: Download , Prepare , and Analyze , which involve downloading the raw data, preparing the data for the analysis, and inferring the networks (Figure 1). **Download** Data downloaded include RNA-seq, mutation, methylation, and copy number variation data, which are the modalities used to generate regulatory networks and to characterize genetic and epigenetic aberrations that could explain dysregulation in cancer. Clinical and phenotypic data are also downloaded to support downstream investigations. **Prepare** Gene expression and methylation data are cleaned and pre-processed. Sample duplicates, outliers, and lowly-expressed genes are removed. CpG-level methylation is mapped to overall gene promoter methylation values, and sample identifiers are matched to facilitate multi-omic integration. **Analyze** The *tcga-data-nf* pipeline provides an interface for estimating gene regulatory networks with PANDA [33] and multi-omic partial correlation networks with DRAGON [39]. For both methods, *tcga-data-nf* also facilitates the generation of sample-specific networks with LIONESS [47], and ALPACA is used to identify differential modules between multiple PANDA networks [46].

Although designed as a full pipeline that runs all the steps above, the modular design of *tcga-data-nf* allows users to run the Download , Prepare , and Analyze steps independently(Figure 1). The decoupling of these three different steps is useful from a practical standpoint; the Download step does not require efficient computational resources, but it is time-consuming and is performed once to generate a long-term data repository. The Prepare step involves several choices of data preparation parameters, and a user may wish to test several configurations. The Analyze step is likely to be run multiple times in the course of an investigation, to observe how various parameters affect the results or fit new types of networks.

The Prepare step of *tcga-data-nf* is powered by the associated *NetworkDataCompanion* (*NDC*) R package, which provides a wide range of functions for working with different data modalities in TCGA. While there are many existing Bioconductor packages to work with TCGA data, it is often necessary to resort to multiple tools to carry out simple functions. The *NDC* package solves this issue by integrating a set of tools into one package, streamlining the routine steps of TCGA data processing. TCGA-specific functions allow users to filter and map gene and sample identifiers between modalities, addressing one often vexing challenge with heterogeneous data. Omic-specific functions allow users to normalize, transform, and filter the data, based on the specific needs of the downstream tasks. Although *NDC* is designed specifically to support *tcga-data-nf*, the package is standalone and can be and reused in other contexts.

State-of-the-art environment and containerization tools are also integrated with *tcga-data-nf*, which can interface with Docker [50], Singularity [51], and conda [52]. We provide a general container, (vfanfani/tcga-data-nf) and configuration details for both Docker and Singularity; we also provide a customizable configuration structure for the user to define conda environments.

In developing *tcga-data-nf* and *NetworkDataCompanion*, we generated PANDA and PANDA-LIONESS networks for the ten most common tumors in TCGA. We have published these networks in GRAND, our group's cloud-based network database, so they can be used by the wider cancer research community without the need to run the more expensive steps of the workflow. Finally, we present a full analysis using the colon cancer networks (TCGA-COAD), uncovering key differences between consensus molecular subtypes. Below, details of the workflow are presented, including descriptions of the *tcga-data-nf* pipeline steps and *NetworkDataCompanion* functionalities.

Download

For network generation and analysis, we focus on five TCGA data modalities: gene expression, methylation, copy number variation (CNV), and mutation data, alongside patient clinical data. All TCGA data are downloaded from the Genomic Data Commons (GDC project [53]). To download methylation, CNV, mutation, and clinical data, *tcga-data-nf* uses the "TCGAbiolinks" [43] and "GenomicDataCommons" [40] R packages. For the gene expression data, *tcga-data-nf* uses the recount3 [54] R package, taking advantage of recount3's normalization tools and standardized processing across studies. We also leverage recount3 to download GTEx gene expression data to enable future analyses comparing cancer with normal tissue [55].

Given the variety of data types and the large number of parameters that need to be specified, the Download step is driven by a json configuration file (Listing 1). The structure of this file is modality-centric, which means that for each data modality (gene expression, mutations, CNVs, ...) one can specify the cancer types to be downloaded. The configuration also provides users with the ability to pass a list of samples that one wants to select from the entire population, which is useful for discarding problematic samples or to focus on a specific subpopulation.

The Download step generates simple, comma-separated tables of metabdata that store key configuration parameters used to download each dataset and the path of the resulting files. These metadata tables directly interface with the following Prepare step. A schematic of the Download step is shown in Supplementary Figure S1. For the Download step we have a written a dedicated testing profile (testDownload) described in the "Testing" section that allows the workflow to be piloted and validated.

Prepare

The raw downloaded data needs to be pre-processed before being used in downstream analyses. pre-processing requires a range of parameter choices for steps such as normalization and filtering, leading to a large number of possible parameter configurations. Since these choices can affect the results and conclusions from any analysis[56, 57], *tcga-data-nf* provides a config file for the user to define such parameters for the pre-processing steps implemented in the Prepare step.

As implemented in *tcga-data-nf*, the Prepare step primarily deals with pre-processing gene expression and methylation data, which are the data types we use here for the generation of GRN and association networks. However, users may also have specific pre-processing steps that are unique to their analysis. To this end, *tcga-data-nf* is naturally extensible and users can easily implement their own functions to integrate with the existing Prepare step. Below, we describe the key steps used to pre-process expression and methylation data in the current pipeline.

Expression From recount3, we obtain gene-level raw count data for RNA-seq data from both TCGA and GTEx. To clean these data, we implemented the following steps:

- Normalization: Raw count data are normalized, with options to generate either TPM [58, 59] or CPM (CPM with TMM normalized library size [60, 61]).
- Duplicates: Where duplicate samples are present (two or more samples from the same research subject), the sample with the highest sequencing depth is retained and others are discarded.
- Batch correction: If specified, batch effect is removed using ComBat [62, 63]. We also visualize the effect of batch removal using PCA.
- Low expression: We remove genes that have low expression, defined as those genes with less than n counts in at least p% of samples for user-defined values of n and p.
- Sample purity: For TCGA tumor samples, we remove those that have low purity, using previously computed purity values [64] and allowing the user to specify their method of choice.
- Tissue type: TCGA contains both tumor and adjacent normal tissue samples, and one can save the data for these tissue types separately to facilitate subsequent analyses comparing tumor to adjacent normal tissue.

All these choices are set by appropriate parameters in the configuration file, and users can specify multiple values for each parameter so that *tcga-data-nf* outputs results for all parameter combinations.

Methylation The TCGA methylation array data undergo two key pre-processing steps: mapping of individual CpG probes to genes to generate gene-level promoter methylation values and general data cleaning/transformation. By default, CpGs are mapped to genes using the publicly available annotation for the Illumina 450k array, mapped to hg38 using Gencode v36(https://zwdzwd.github.io/InfiniumAnnotation) although users can supply their own annotation file if desired. Once CpGs are mapped to genes, the average promoter methylation for each gene is calculated. The promoter region is defined as the area 200 basepairs upstream of the transcription start site (users can redefine this boundary), and average of the methylation beta values for any probes falling within this region is calculated.

It is not necessary to perform this mapping to promoter methylation over all the genes represented on the EPIC array but rather to a particular subset that may be relevant for a particular analysis. For example, in the application of DRAGON [39] that we describe, we map the probes only to genes encoding transcription factors (TFs). The default behavior is to map to all genes on the array.

After obtaining gene-level methylation for each sample, the following pre-processing options are available:

- Duplicates: If a sample has multiple methylation array profiles, the user can handle these in a number of ways, including choosing the duplicate with the least missingness, choosing a duplicate at random, or excluding samples with duplicates altogether.
- Missing data: Any gene that has missing promoter methylation values for more than m% of the samples is removed from the analysis, with m = 20% by default. If a gene has missing values for $\leq m$% of the samples, these missing values are estimated by mean imputation.
- Conversion from beta to M-values: The mean promoter methylation beta value $\beta \in [0, 1]$ is converted into an M-value $M \in (-\infty, \infty)$ using the formula:

$$M = \log_2 \left\{ \frac{\beta}{1-\beta} \right\} \quad (1)$$

  See [65] for a detailed discussion of the relative merits of using β-values vs. M-values in methylation analyses.
- Transformation to approximate normality: We provide the option to apply a nonparanormal transformation [66] to the

M-values to achieve approximate normality in the distribution for input into DRAGON as it requires approximately normal data. The nonparanormal transformation is powered by the `huge.npn` function of the R package `huge` [67].

A simple schematic of the Prepare step is shown in Supplementary Figure S2. At the end of these steps, we obtain a comma-separated file, with rows being the probes and columns the samples. As in the Download step, a metadata table storing the parameters used for the Prepare step is also produced. The Prepare step relies heavily on the companion R package *NetworkDataCompanion* (*NDC*), which is described in depth in the "*NetworkDataCompanion* " section.

Analyze

Following the downloading and preparation of TCGA data, the Analyze step implements the necessary code to generate network models. The Analyze step generates two types of networks: GRNs, generated with PANDA, and multi-omic association networks, generated with DRAGON. PANDA [33] is a GRN inference method that uses bulk expression data, together with a prior TF-gene binding network (based on TF motif mapping) and a TF-TF protein interaction network, and generates population-level bipartite TF-gene regulatory networks. DRAGON [39] is a network inference method based on Gaussian graphical models that infers partial correlations on multi-omic data. We also used LIONESS [47], which estimates sample-specific networks by interpolation between a network for the entire population and that for the population less the sample for which we are estimating a network. LIONESS is agnostic to network estimation method; the choice of the method is delegated to users who can specify the method in the configuration file. In our pipeline, we used LIONESS to estimate both PANDA and DRAGON networks for each sample. Finally, to compare the connectivity of PANDA GRNs, we use a netZoo method called ALPACA [46], which identifies the gene modules that best distinguish two networks by maximizing their differential modularity. To show how the workflow can be easily extended to run methods that do not belong to the netZoo suite, we have implemented WGCNA [?] and GENIE3 [?], that are third-party network inference methods . A simple scheme of the Prepare step is shown in Supplementary Figure S2.

The *analyze* workflow relies on implementations of PANDA, DRAGON, and LIONESS in the netZooPy package and ALPACA from the netZooR package [45] . The processes for network inference use the command line interfaces and Python objects for PANDA, DRAGON, and LIONESS; these require users to specify the expression/methylation/CNV input files and the parameters for each method. ALPACA uses the output from PANDA networks to identify condition-specific modules, and only requires the unique identifiers of the inferred GRNs. Conveniently, the Prepare step in *tcga-data-nf* generates a metadata table that the users can directly use to specify which files are then used by the Analyze step. Given the storage requirements of the data the pipeline can generate, we updated netZooPy to save networks in Hierarchical Data Format (HDF) which reduces the size and reading/writing time for storing the networks.

Lastly, we note that although we have assembled pipeline of methods that focuses on gene expression and methylation to generate network models, the workflow can be easily edited to include other data types and methods. We have provided examples of this by incorporating CNV data in estimating DRAGON networks and we have added two third-party methods, WGCNA and GENIE3, to the pipeline, including clear documentation on how it can be extended, to facilitate community efforts.

Full

The full pipeline combines the Download , Prepare , and Analyze steps described above. It is designed to be run with a single command, and it represents most complete network analysis pipeline. The full pipeline generates PANDA GRNs, which are compared with ALPACA. It also generates multi-omic DRAGON methylation-expression and CNV-expression networks using DRAGON, and incorporates LIONESS to generate both PANDA-LIONESS and DRAGON-LIONESS sample-specific networks.

While we recommend separating the three steps by, e.g., downloading the data once and then running the Prepare and Analyze steps as needs change, there are instances where researchers may wish use the full pipeline to run all three steps for a single project at once. In the sections "Multiomics associations identify changes between colon subtypes" and "Regulatory differences between colon cancer subtypes GRNs", we show how the full pipeline can be used to generate DRAGON and PANDA networks for the TCGA colon cancer consensus molecular subtypes, and to generate insight into the differences between their regulatory programs.

For the full pipeline, the configuration files and parameters mirror those of the three modular pipelines above. First, a "json" configuration file similar to that used in the Download step (Listing 2) needs to be populated with all the data modalities of interest. Then, the processing and analysis parameters need to be specified in the Nextflow configuration file as they were in the Prepare and Analyze steps.

NetworkDataCompanion

The *NetworkDataCompanion* (*NDC*) R package supports preprocessing of TCGA bulk RNA-seq and DNA methylation data. The purpose of having a version-controlled R package for these functions is to attain the high standard of reproducibility in the overall *tcga-data-nf* pipeline. While *NDC* is the engine behind the *tcga-data-nf* workflow processing steps, the software is standalone and can be installed and used outside the workflow. *NDC* currently provides three broad classes of functions: functions for mapping identifiers, functions for filtering data, and functions for preparing expression and methylation data (such as normalization and scaling) (Figure 1). While many of the functions described below are intuitively simple, it is worth noting that the complexity of the TCGA project and its wealth of data require commensurately complex data wrangling to handle tasks such as filtering by sample quality and matching sample identifiers between omics types. The goal of *NDC* is to provide a version-controlled and unit-tested environment for developing and maintaining tools for these tasks, with the end goal of enhancing accuracy and reproducibility.

Mapping functions Given the variety of data types used in the pipeline and the number of other resources with which they interface, there are many instances where we need to map between "synonymous" identifiers. We have implemented several wrapper functions that use existing tools such as GDC and TCGAutils to retrieve and convert various sample identifiers (TCGA barcodes, UUIDs). We have also implemented functions for translating gene names between Ensembl IDs [68], HUGO Gene Nomenclature [69], and Entrez IDs [70, 71] by leveraging Gencode v26 [72] which was used for TCGA and the AnnotationDbi R package [73].

Sample filtering functions By default, *tcga-data-nf* downloads and processes all samples available for a particular TCGA dataset. *NDC* provides three different functions that allow a user to filter these samples. First, a user may eliminate duplicate samples using one of four methods: select the sample with the strongest signal based on RNA sequencing depth (for expression duplicates), select the sample with the least missing data (for methylation duplicates), select the sample with the highest tumor purity (for any sample type for which tumor purity is available), or select a single sample from a set of duplicates at random. Second, a user may filter samples based on the TCGA sample type (for example, primary tumor tissue, metastatic tissue, or adjacent normal tissue). Finally, a user may filter samples based on tumor purity, excluding samples where the number of non-tumor cells is too large according to sample published annotation [64].

Data preparation functions Lastly, with *NDC* users can apply

common data transformations to both gene expression and methylation data. For RNA-seq data from recount3, one can normalize read counts to transcripts per million (TPM) [58] or counts per million (CPM) [61] and their corresponding log transformations, where a pseudocount is added to avoid undefined logs. For methylation data, functions are provided to convert methylation beta values to m-values (logit base-2 transformed beta values) and vice-versa [74] and to aggregate methylation values to get gene-level information, such as average methylation within a promoter region or gene body.

Collectively, the functions in *NetworkDataCompanion* represent a comprehensive set of tools for basic processing, filtering, and mapping that are needed to clean and prepare TCGA data. We note that although there are R packages that cover each of the individual tasks described above, *NetworkDataCompanion* aggregates them into a unified solution in one version-controlled and unit-tested package to facilitate their use together and to allow them to be seamlessly integrated into the *tcga-data-nf* pipeline.

Multi-omic partial correlation networks identify differences between colon subtypes

Colorectal cancer affects nearly two million individuals worldwide each year and is forecast to increase in incidence by 84% in the next 20 years [75]. Molecular profiling studies have identified four major expression-based consensus subtypes (CMS1, MSI immune, CMS2, canonical, CMS3, metabolic, and CMS4, mesenchymal) [48] with distinct phenotypic and clinical features. CMS1 and CMS3, each of which has a distinctive genomic and epigenomic profile, together represent around 25% of cases. CMS2 and CMS4 are the most common subtypes, and although they have similar patterns of somatic mutation, structural variation, and methylation, they differ significantly in outcomes; CMS2 tumors have good survival rates, while CMS4 cases are characterized by more aggressive tumors and poorer prognosis [76]. Additional studies have described intra-tumor heterogeneity and clinically actionable features that distinguish CMS4 and have found evidence that this more aggressive mesenchymal subtype often arises from CMS2-like tumors [77]. We then reasoned that multiomics association networks and GRNs could provide further insight into the mechanisms that drive the difference between these subtypes.

Using the full pipeline, we specified the TCGA samples that belong to each subtype [48], downloaded and pre-processed the data, and generated various network models for each subtype. We began with the four subtype-specific DRAGON partial correlation networks integrating TF gene expression with promoter methylation. Given F transcription factors for which we have both methylation and expression data, each network has $2F$ nodes, is symmetric, and can be divided into three blocks: (i) expression-expression partial correlations (E_i, E_j)), (ii) methylation-expression partial correlations (M_i, E_j), and (iii) methylation-methylation partial correlations (M_i, M_j).

Because promoter methylation is generally inhibitory, we expect it to exhibit an inversely partial correlation with gene expression [78, 79]. Indeed, by examining the distribution of methylation to expression edge weights (M_i, E_j), we see that weights for edges connecting a gene to the its promoter methylation (M_i, E_i) have a distribution more strongly skewed to negative values than the distribution of the promoter methylation to the other genes (M_i, E_j) (Figure 2A). Additionally, we notice that there is good correlation of these (M_i, E_i) edges between different subtypes (Supplementary Figure S4). To understand whether these TFs for which we infer direct epigenetic regulation are relevant to the cancer phenotype, we select those for which the (M_i, E_i) edge is in the first decile of the distribution of at least one of the subtypes. We then obtain 120 TFs for which we have evidence of the "silencing" effect of the promoter methylation on the same gene (Supplementary Figure S5). Some of these TFs (STAT5A, CREB3L1, ZNF24, HMGA1, IRF8, PAX8, CDX2, CREB3L2, TFEB, MGA, NFIB, KLF6, LEF1, HOXD13, HOXA13, HOXB13, GATA2) are known to be cancer drivers [80, 81, 82, 83, 84]. For example, STAT5A , the only TF that has low (M_i, E_i) edges in all subtypes, is a known oncogene involved in the JAK signaling cascade [85, 86], while CREB3L1, LEF1, PAX8 are all known to be involved invasion and metastasis [87, 88, 89, 90]. To further validate these networks, we compared DRAGON edges with the co-expression edges in the StringDB database, and found evidence of correlation with our expression-expression edges (Supplementary Figure S6).

We then explored the inter-omic partial correlation edges (M_i, E_j) that connect methylation associated with one TF to the expression of another TF and focused on the edges that differ between the subtypes, under the hypothesis that such edges can identify complex regulatory relationships that distinguish disease states. We selected the edges with the highest absolute value (either the strongest positive or negative partial correlation) and used hierarchical clustering (average linkage, Euclidean distance) on these edges. We identified five clusters (Figure 2B) with clusters A and D showing distinct patterns that represent reversed associations between CMS2 and CMS4. Even when compared to the rest of the edges connecting the same TFs (Supplementary Figure S4), these edges switch direction between the two, suggesting a specific change in the regulatory process. Using functional enrichment analysis, we found that TFs in clusters A and D are involved in the metabolism of proteins and DNA repair and that cluster D, in contrast to cluster A, includes genes preferentially involved in the Immune System (Supplementary Figure S8). specifically BATF, GATA3, NFATC3, NFATC2 and TP53 are involved in the Immune System, while E2F, TERF2, and TP53 participate in cellular responses to stimuli (Figure 2C). This over-representation of immune-related TFs is consistent with reports that CMS4-like tumors exhibit greater degree of immune infiltration than do other subtypes [77]. Further, epigenetic changes in the FOX/HOX and SNAI TF families are known to be involved in colorectal cancer etiology [91], and TP53, FOXA1, GATA3, and GATA6 are all TFs that, when active in an aberrant form, are recognized as being hallmarks of cancer [83]. All these differences between CMS2 and CMS4 can also be visualized at once as the subgraph that emerges from cluster D, Figure 2D. We note a general limitation of this functional enrichment analysis is that most TFs are only annotated to transcription pathways and not those they have a regulatory effect on.

Since somatic DNA variation is well-studied in the context of cancer and has previously been established as characteristic of subtype CMS1, we have focused our investigation on the associations between promoter methylation and expression, data types which have not yet been as thoroughly explored as somatic variation. However, as an illustrative example of the flexibility of *tcga-data-nf* ot include additional omics, we have also generated CNV-expression DRAGON networks that capture associations between copy number changes at the gene level and expression. Analogously to what we described above for the methylation-expression DRAGONs, we now have three types of edges: CNV-CNV (C_i, C_j), CNV-expression (C_i, E_j), and expression-expression (E_i, E_j). By exploring these edge distributions, we demonstrate that CNV-expression dragons are also able to detect key effects of DNA aberrations as follows.

As we have done for the methylation-expression DRAGONs, we first confirm the validity of these networks. CNV usually involve deletions of duplications of DNA segments. For this reason, genes that are near each other on the genome are expected to have similar CNV values. This effect is also visible also in the adjacency matrices representing the CNV-expression DRAGON networks, where a block structure in the CNV-CNV edges is evident(Supplementary Figure S9A). Moreover, we have evidence that, even in this case, DRAGON networks are able to capture direct effects of CNV onto expression: the CNV-expression edges on the same TFs (C_i, E_i) tend to be skewed towards positive values (Supplementary Figure S9B), which is expected behavior for somatic variation. Within the top 20 TFs that show high correlation between CNV and expression, we

observe known colon cancer drivers [83] ZNF703 and SMAD4, and IRF2 and GATA6 whose DNA aberrations are likely oncogenic [78].

Finally, we identify which TFs have the most different CNV-expression edges between the CMS2 and CMS4 subtypes. For each CNV node C_i, we compare the edge set $\{(C_i, E_j)\}$ between the two subtypes. We conducted a paired Wilcoxon signed-rank test, for each TF i, testing the null hypothesis that there is no difference in CNV-expression edge weights between CMS2 and CMS4 (Supplementary Figure S9C). Among the top TFs with varying edges between CMS2 and CMS4 based on the Wilcoxon test results were KLF16, which plays a role in the stress-related programming of colorectal cancer [92], DOT1L, a methyltransferase that regulates core stem cell genes and affects tumorigenesis and drug resistance [93], and colon cancer drivers HIF1A and CUX1, both of which are implicated in tumorigenesis and progression [84].

Regulatory differences between colon cancer subtype GRNs

The DRAGON networks that we inferred capture information about patterns of DNA methylation and how these patterns are associated with transcription factor expression. The working hypothesis behind this analysis is that some transcription factors exhibit altered patterns of methylation that affect gene expression and ultimately exert downstream effects that help to define different cancer subtypes. Indeed, in the DRAGON networks constructed on transcription factors, we identified a number of edges that differ between the CMS2 and CMS4 subtypes and provide a plausible explanation for the differences they present in the clinical context. However, the partial correlations represented by edges in DRAGON networks are limited in that they are measures of association and do not capture the actual regulatory effects that TF have on their target genes.

PANDA is a GRN inference method that uses prior knowledge on motif binding and TF-TF physical interactions together with assayed gene expression to generate a bipartite graph associating TFs with the genes they likely regulate. Each TF-gene edge is weighted by a measure of the evidence of a regulatory relationship. We generated PANDA GRN networks on the TCGA-COAD data, enabling us to perform an analysis directed at identifying the regulatory context for the TFs identified in clusters A and D for which we have evidence of changes in methylation and expression patterns between CMS2 and CMS4. After having identified the TFs of interest with the DRAGON analysis, we proceeded to investigate the functional role of their targets. We compared the two PANDA networks and selected the edges with the largest changes between the two subtypes, to identify targets of the selected TFs that are differentially regulated. A gene set enrichment analysis on these differentially targeted genes reveals that they are involved in the transcriptional misregulation of cancer and various immune-related Reactome pathways that include "Creation of C4 and C2 activators," "Initial triggering of complement," and "Signaling by the B Cell Receptor (BCR)" (Supplementary Tables S1,S2). Moreover, TFs in cluster D seem to consistently differentially target genes in the "TGF-*beta* signaling", "Cytokine-cytokine receptor interaction", and "Antigen processing and presentation" pathways (Supplementary Figure S10 and Supplementary Tables S3, S4. These pathways are consistent with prior evidence that TGF-β is not only associated with poorer prognosis in colon cancer, but it is also involved in the transition between CMS2 to CMS4 [76].

Lastly, we extend our search beyond the TFs identified by DRAGON and perform a genome-wide comparison the two PANDA networks with ALPACA to identify a set of differential modules that optimize the diferential modularity between the two subtypes. ALPACA finds 14 differential modules. First, we checked the overall differences in network connectivity by looking at the differential in-module and out-module degrees of genes and TFs (details are in the method section). Modules 1, 2, and 4, are those the exhibit the biggest differences in connectivity (Supplementary Tables S5 and S6).

We investigated which pathways are enriched in these modules (Supplementary Figure S12). Modules 1 and 2 are enriched for Immune System and Cell Cycle pathways, confirming once again that CMS2 and CMS4 exhibit differences in the immunological response and cell growth and DNA replication. Both module 2 and 4 include TFs and genes that are known for their role in cancer. Interestingly, module 4 is instead uniquely involved into pathways that control the Extracellular Matrix Organization.

ALPACA provides a ranking of nodes based on their contribution to modularity, prioritizing those that justify the strongest changes in modularity (Supplementary Figure S11). For instance, if we look more in depth into the TFs that have the highest contribution to modularity for module 1, the majority have been validated as key regulators of colorectal cancer progression. Indeed, 6 out of 10 leading TFs, ZNF334 [94], FOXD2 [95], FOXD3 [96], POU3F3 [97], NR1H4 [98], VSX2 [99], have direct evidence, and some experimental validation, of their involvement in colon cancer risk and progression. Lastly, while many zinc finger proteins are still not well characterized, ZNF341 is a regulator of STAT3 and plays a key role in hyper-IgE syndrome (HIES), a condition that often manifests as severe and chronic bacterial infections [100]. In parallel, we found reports of associations between IgE-mediated immune reactions and colorectal cancer risk [101]. While we do not have definitive evidence, or validation, of the role of ZNF341 in the colon cancer subtypes, we believe that this is a suggestive result that hints at how multi-omic network analysis can find complex regulatory patterns.

Testing

Apart from the main workflows for TCGA data analysis, which we showcased in the previous section, our framework also offers a testing option that assesses the general availability and formatting of data and software and runs a trial analysis on a sample dataset.

Nextflow profiles are sets of configuration parameters accessible with the "–profile" scope. For testing purposes, we provide testing configuration profiles for all the workflows with the following names: "test" for the full pipeline, and "testDownload", "testPrepare", "testAnalyze" for the specific workflows.

test. This profile runs the full *tcga-data-nf* pipeline on a subset of TCGA Pancreatic Adenocarcinoma data (PAAD). All parameters are specified in the ('conf/test.config')

testDownload. To test the download step, we need to confirm that the TCGA data are available and retrievable and hence we cannot use minimal dummy datasets. To avoid downloading files that are too large, during testing we only retrieve data from TCGA Pancreas Adenocarcinoma, one of the smallest datasets. All data are downloaded into the 'results/download_test/' folder.

testPrepare. To test the prepare step, dummy expression and methylation datasets are provided that contain randomized sample labels and values. Both datasets have only 10 samples each, hence this step should compute quickly. To further simplify this test, each of the parameters that we pass to the recount pipeline is tested for only one value, such that only one input configuration is tested and only one output is produced.

testAnalyze. To test the analyze step, we provide dummy pre-processed data for expression and methylation data in the `testdata/analyze_expression.csv` and `testdata/analyze_methylation.csv` files. Alongside the input data, we also provide TF-target binding and PPI network dummy data which are necessary to infer PANDA networks. The test requires that all network methods (PANDA, DRAGON, LIONESS, and ALPACA) are computed in order to confirm that the whole implementation is functioning properly and the netZooPy/netZooR

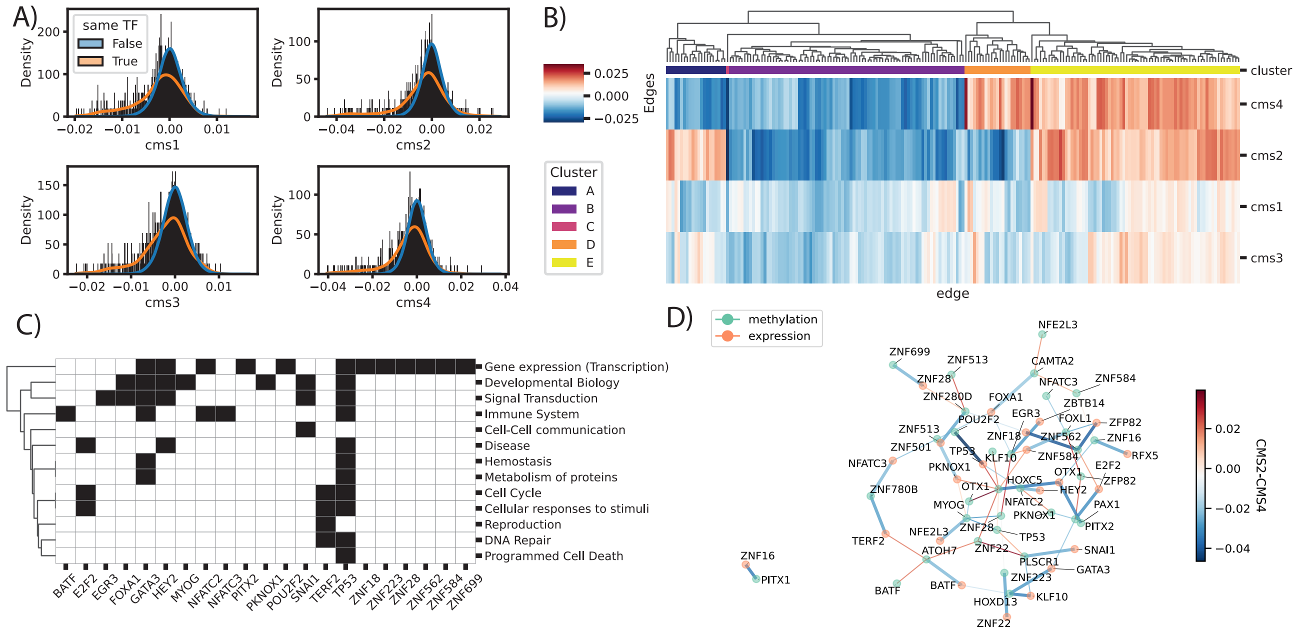

Figure 2. Differences in methylation-expression association of TFs in colon cancer subtypes A) Distribution of partial correlation values between methylation and expression of TFs in all subtypes. In orange we show the values for the edges of the same TF, that is the correlation between the methylation of the promoter and the expression of the that same TF. As expected, methylation and expression tend to be negatively correlated. The histogram represents the distribution density and it is normalized per subtype and per group. B) For inter-modality edge weights (methylation to expression) we remove the edges between the same transcription factor, and we select the first 200 strongest edges (highest average absolute value of correlation) and we cluster them by correlation values for each subtype (average linkage, Euclidean distance). Interestingly, for cluster A and D the edge values for CMS2 and CMS4 are swapped in direction. C) Annotation of all TFs in cluster D (columns) to the Reactome parent term. "Immune system" and "Cellular respondes to stimuli" are more consistenly involved in cluster D, in comparison to cluster A. Here we can discern which TFs are annotated to each term, for instance a a good number of TFs are involved in the immune system (BATF, GATA3, NFATC2, NFATC3, TP53). As expected many of the TFs are annotated to generic transcription pathways, and TP53 is annotated to almost all terms.D) Association graph for the TFs in cluster D for CMS2 (left) and CMS4 (right). We have selected the edges that belong to cluster D (thicker edges) and we have added other 20 top edges, by absolute value, that connect the same TFs (thinner edges), such that we have a connected graph. We show the TFs as nodes with different colors for methylation (green) and expression (orange), and we show the edge values color-coded by the differential partial correlation values.

packages are correctly installed and running.

Computational performance

Nextflow allows users to track computational requirements and performance of all processes. While wall and CPU time heavily depend on the data type and size the estimation of CPU and memory usage on sample datasets can help guiding the user in their choices. First, we report the performance of the pipeline on test data. These are the minimal requirements to run the workflow, as the test data is typically smaller that the real one, but do provide an estimate of the relative size of processes. We report the performance on an AWS EC2 instance c5.4xlarge with 32Gb of memory and 16 vCPUs. We ran all tests with the v0.0.15 version of *tcga-data-nf* with standard configuration files and no parallelization of processes. The test full workflow requires 1h 11min of wall time (4.7 CPU hours) and a peak memory of 13.6 Gb for runTCGAPanda. This is a realistic run of the workflow on the actual TCGA-PAAD data. The *tcga-data-nf* documentation includes the report for this test workflow, including with a detailed description of the CPU and memory performance for each of the processes (https://github.com/QuackenbushLab/tcga-data-nf/blob/main/assets/execution_report_test_full.html). The testDownload workflow took 9 minutes (0.6 CPU hours) and has a peak memory usage of 4.9Gb for the download CNV process. The testPrepare run uses 1m 23sec (0.1 CPU hours) and has a peak of 4 Gb for GetGeneLevelPromoterMethylation. Lastly, testAnalyze only took 43sec (<0.1CPU hours) and has a peak of 2.9Gb of memory for the alignMethylationExpression process.

Secondly, running LIONESS on PANDA is undoubtedly the most expensive process, as it reconstructs one leave-one-out PANDA network for each sample in the population and requires double the memory ([102]). However, netZooPy supports GPU-based computation of PANDA-LIONESS network [?]. On an Nvidia H100 GPU, with 32Gb of memory allocated, running PANDA-LIONESS for TCGA-PAAD (183 samples) took 62 minutes, which is consistent with the observation that each PANDA network computation takes around 19 seconds.

Discussion

There is a growing recognition that inferring and analyzing gene regulatory network models can provide unique and verifiable insights into the drivers of disease, particularly in rich public databases such as TCGA [34]. The GRAND database provides access to some freely available, genome-wide gene regulatory network models from TCGA that have been published during the years [49]. Yet, GRAND is intended as a dynamic resource and, as methods and knowledge evolves, we expect the database to be enriched with new networks. However, for those wishing to generate network models from the TCGA data, setting up the requisite environments and data structures can present some challenges, even for those with experience in bioinformatics and computational biology. In this manuscript, we aim to bridge the gap between the wealth of data in TCGA and the skills and computational resources needed to analyze it.

We describe an end-to-end reproducible workflow to download, pre-process, and generate regulatory networks from TCGA cancer data with a single command. This workflow is publicly available, uses fully open-source software, and adheres to what has been deemed the gold standard for reproducibility [3]. The workflow allows the pre-processing of multi-omic data and inference of regulatory networks without requiring users to write code *de novo*;

instead, the user need only specify a relatively small number of parameters. In addition to the workflow itself, we also provide several supporting resources to facilitate more agile and reproducible generation of insights from the TCGA data. These resources include Docker containers and conda environments, extensive configuration files, documentation describing how to reuse the workflow, and pre-generated networks for the ten most common cancer types in TCGA.

As a demonstration of the value and flexibility of the *tcga-data-nf* pipeline, we applied the workflow to TCGA colon adenocarcinoma (COAD) data. The ability of the *tcga-data-nf* pipeline to reproducibly and repeatedly run the same workflow with different inputs is of particular relevance in the COAD dataset, where consensus molecular subtypes are well-established and it is of interest to study networks within each of the subtypes. Using *tcga-data-nf*, we generated multi-omic association networks (DRAGON) and GRNs (PANDA) to study the four consensus molecular subtypes of colon cancer. We found evidence of previously undescribed methylation-gene expression interactions that target TGF-β signaling and that may help to explain factors influencing the transition between subtypes CMS2 and CMS4. To fully showcase the use of *tcga-data-nf* we also computed DRAGON networks for CNV and expression and analyzed the differences between CMS2 and CMS4 PANDAs with ALPACA. The code for this example is available at https://github.com/QuackenbushLab/tcga-data-supplement, and the data have been archived on the Harvard Dataverse at https://doi.org/10.7910/DVN/MCSSYJ, ensuring complete reproducibility of the analysis.

The biggest challenge we faced in designing *tcga-data-nf* was balancing the trade-off between flexibility and completeness. We reasoned that individual pieces of software, such as GDC, TCGABiolinks, edgeR, and netZooPy already provide a broad set of functions covering all the steps required for network generation and analysis. As such, the workflow was designed to chain these tools seamlessly together, allowing users to carry out complex analyses simply by specifying a small number of parameters. While flexible by design, the release version of *tcga-data-nf* does not provide users with unlimited options. For example, we provide only two common RNA-seq normalization methods, the full workflow can only be applied to single tumor types and cannot generate pancancer analyses without post-hoc coding, and the workflow does not cover all possible data types available from TCGA, such as miRNA expression. However, we believe that the flexibility of *tcga-data-nf* enables the user to easily overcome these limitations. We organized *tcga-data-nf* so that it can be easily extended to include other data types and analysis methods and we provide individual Download , Prepare , and Analyze steps that can be used separately from the rest of the pipeline. We have also provided examples for how the pipeline can be modified and expanded, e.g.,to download more data modalities or run other GRN inference methods.

At last, we recognize that a limitation of *tcga-data-nf* is that it was designed to generate GRNs from bulk sequencing data from TCGA, and most of the intermediate steps available are specific to these modalities. While one could, in principle, use *tcga-data-nf* to download scRNAseq data from GDC from the CPTAC3 project or extend the Analyze steps to include SCORPION [103], a method for inferring GRNs from single cell transcriptomics data, we expect that other workflows will address these tasks more efficiently.

Methods

Pathway Analysis

In the section "Multiomics associations identify changes between colon subtypes", we carried out all pathways analysis using the GSEApy package [104]. We tested for over-representation (ORA) of TFs and genes in both the Reactome and KEGG sets of pathways with a hypergeometric test. For both cases we selected the appropriate background, that is, all TFs in the DRAGON networks or the gene targets in the PANDA networks. The KEGG dataset was downloaded from the GSEApy package as "KEGG2021" dataset. For all tests, we applied the Benjamini-Hochberg procedure to control the false discovery rate (FDR) during multiple testing correction [?]. From the Reactome database, we downloaded the pathway files on June 18th, 2024. We have downloaded the tables that map each gene identifier to a pathway, and that also map each pathway to the parent terms. For instance "Intracellular signaling by second messengers", "Signaling by GPCR", "Signaling by Hedgehog"... are all part of the "Signaling Transduction" term. To reduce the number of tested pathways, which also reduces overlaps between pathways, we have generated a "slim" set of pathways. For each leaf in the Reactome dataset, we have kept only the "parent" node. This way we avoid keeping all the nodes that are too small, and we keep only the depth-1 term.

In section "Regulatory differences between colon cancer subtypes GRNs " for the analysis of ALPACA results we used R's clusterProfiler package to run the KEGG pathway analysis. Indeed, we have included an example on how to analyze the data in R. Test pvalues are corrected with Benjamini-Hochberg procedure and we consider as significant those with FDR<0.05.

All code and data used for the pathway analysis are in the https://github.com/QuackenbushLab/tcga-data-supplement/ repository.

Reference data

PANDA uses prior knowledge on putative TF-motif binding and TF-TF contact, i.e., protein-protein interactions (PPIs). To create the regulatory motif network, we downloaded TF motifs for *Homo sapiens* with direct or inferred evidence from the Catalog of Inferred Sequence Binding Preferences (CIS-BP) Build 2.0, accessible at http://cisbp.ccbr.utoronto.ca. These TF position weight matrices (PWM) were mapped to the human genome (hg38) using FIMO [105]. We retained only highly significant matches ($p \leq 10^-5$) occurring within the promoter regions of Ensembl genes (specifically, GENCODE v39 annotations retrieved from http://genome.ucsc.edu/cgi-bin/hgTables). These promoter regions were defined as the interval of [-750; +250] base pairs centered around the transcription start site (TSS). This process yielded an initial set of potential regulatory interactions involving 997 TFs that collectively targeted 61,485 genes.

For the TF-TF cooperativity prior, we obtained PPI data from the StringDB database (version 11.5) using the STRINGdb Bioconductor package [106]. Subsequently, we filtered the PPI data to retain only interactions between transcription factors in the TF-motif network (using a score threshold index of 0). To maintain consistency in PPI scores, we normalized them by dividing each score by 1000, thereby restricting the values to a uniform range of 0 to 1 for both the PPI dataset and the TF-motif network. Additionally, we set self-interactions between TFs to a value of one. Since PPI networks are inherently undirected, we transformed the data into a symmetric PPI matrix.

Cancer Datasets

We annotated the TFs to cancer-specific collections of genes from the following databases. We downloaded the OncoKB ([81]) gene list on December 13, 2022 from https://www.oncokb.org/. We downloaded the Cancer Gene Census v101 [84] from https://cancer.sanger.ac.uk/cosmic on April 25, 2025; for the colon-only genes, we selected those that have the terms 'colon' or 'colorectal' as Tumor Types (Somatic). Finally, we downloaded the DisGeNet v25.1.1 [82] curated gene-disease associations for the term C0009404 (Colorectal Neoplasms) from https://www.disgenet.com/ on April 25, 2025.

Differential degree testing

Given two PANDA GRN networks for subtypes CMS2 and CMS4, with weighted edges $e_{u,v}^{(CMS2)}$ and $e_{u,v}^{(CMS4)}$ connecting TF u to gene v, ALPACA uniquely assigns P TFs and R genes to each of the C modules. For each gene r in module c, we compute the in-module differential degree as the sum of all differences between CMS2 and CMS4 edges connecting the gene to all the TFs P that belong to the same module:

$$\mathrm{diff}_r(c) = \sum_{p \in \mathcal{P}} (e_{pr}^{CMS2} - e_{pr}^{CMS4})$$

For comparison, we also compute the out-module differential degree, accounting for all edges directed to nodes that do not belong to module c,

$$\mathrm{diff}_r(\neg c) = \sum_{i \notin \mathcal{P}} (e_{ir}^{CMS2} - e_{ir}^{CMS4})$$

.

For each module, we ran a Wilcoxon signed-rank test between the in-module and out-module gene differential degrees (Supplementary Table S5). We then ran the same analysis with each TF (Supplementary Table S6), where we obtain the degrees by summing up the edge values connecting each TF p to the R genes.

Availability of source code and requirements

tcga-data-nf

- Project name: *tcga-data-nf*
- Project home page: e.g. https://github.com/QuackenbushLab/tcga-data-nf
- Operating system(s): e.g. Platform independent
- Docker: https://hub.docker.com/r/violafanfani/tcga-data-nf
- Programming language: Nextflow, R, Python, bash
- Other requirements: Java, Nextflow
- License: GNU General Public License v3.0
- WorkflowHub SEEK ID: https://workflowhub.eu/workflows/1306?version=1

NetworkDataCompanion

- Project name: *NetworkDataCompanion*, *NDC*
- Project home page: https://github.com/QuackenbushLab/NetworkDataCompanion
- Operating system(s): MacOS, Linux
- Programming language: R
- License: GNU General Public License v3.0
- SciCrunch registry: RRID:SCR_026532
- bio.tools registry: https://bio.tools/NetworkDataCompanion

Notebooks and configuration files

We provide a GitHub repository that contains i) all configuration files mentioned in this manuscript ii) Notebooks and supplementary data for the analysis of colon cancer subtypes.

- Project name: tcga-data-supplement
- Project home page: e.g. https://github.com/QuackenbushLab/tcga-data-supplement
- Operating system(s): Linux, MacOS, Windows
- Programming language: Python
- License: MIT

Data availability

We precomputed networks for 10 common solid tumors: Breast invasive carcinoma (BRCA), Lung adenocarcinoma and Lung squamous cell carcinoma (LUAD, LUSC), Kidney renal clear cell carcinoma (KIRC), Liver hepatocellular carcinoma (LIHC), Pancreatic adenocarcinoma (PAAD), Skin Cutaneous Melanoma (SKCM), Stomach adenocarcinoma (STAD), Colon adenocarcinoma (COAD), and Prostate adenocarcinoma (PRAD). Raw, multimodal data, and processed data are available at https://us-east-2.console.aws.amazon.com/s3/buckets/tcga-data-nf-precomputed?region=us-east-2&bucketType=general&tab=objects and a guide to the configuration files and data structure can be found in the supplemental repository https://github.com/QuackenbushLab/tcga-data-supplement/blob/main/README.md. PANDA and PANDA-LIONESS networks are available on GRAND (v1.7) https://grand.networkmedicine.org/cancers/ [49].

Replication data for the "Multiomics associations identify changes between colon subtypes" subsection are stored on the Harvard Dataverse (https://doi.org/10.7910/DVN/MCSSYJ).

Declarations

Funding

This work was supported by grants from the National Institutes of Health: ES, CMLR, MBG, VF, JF, KHS, PM, CC, SM and JQ were supported by R35CA220523; MBG and JQ were also supported by U24CA231846; JQ received additional support from P50CA127003; JQ was supported by R01HG011393; KHS was supported by P01HL114501 and T32HL007427; CMLR was supported by K01HL166376; CMLR and ES were also supported by the American Lung Association grant LCD-821824.

Author's Contributions

Conceptualization: VF, ES, PM, JF, KHS, CMLR and JQ; **Methodology:** VF, KHS, PM, JF, SM, CC; **Software VF, KHS, PM, JF, SM, CC**; **Formal Analysis:** VF; **Resources:** JQ, CMRS, and MBG; **Data Curation:** VF, ES, KHS, PM, and CMLR; **Writing – Original Draft:** VF, KHS; **Writing – Review and Editing:** PM, MBG, JF, KHS, CC, SM, CMLR and JQ; **Visualization:** VF; **Supervision:** JQ, CMLR; **Funding Acquisition:** JQ and CMLR.

References

1. Mesirov JP. Accessible Reproducible Research. Science 2010 Jan;327(5964):415–416.
2. Baker M. 1,500 Scientists Lift the Lid on Reproducibility. Nature 2016 May;533(7604):452–454.
3. Heil BJ, Hoffman MM, Markowetz F, Lee SI, Greene CS, Hicks SC. Reproducibility Standards for Machine Learning in the Life Sciences. Nature methods 2021 Oct;18(10):1132–1135.
4. Munafò MR, Nosek BA, Bishop DVM, Button KS, Chambers CD, Percie du Sert N, et al. A Manifesto for Reproducible Science. Nature Human Behaviour 2017 Jan;1(1):1–9.

5. Gentleman RC, Carey VJ, Bates DM, Bolstad B, Dettling M, Dudoit S, et al. Bioconductor: Open Software Development for Computational Biology and Bioinformatics. Genome Biology 2004;.
6. Grüning B, Dale R, Sjödin A, Chapman BA, Rowe J, Tomkins-Tinch CH, et al. Bioconda: Sustainable and Comprehensive Software Distribution for the Life Sciences. Nature Methods 2018 Jul;15(7):475–476.
7. Jalili V, Afgan E, Gu Q, Clements D, Blankenberg D, Goecks J, et al. The Galaxy Platform for Accessible, Reproducible and Collaborative Biomedical Analyses: 2020 Update. Nucleic Acids Research 2020 Jul;48(W1):W395–W402.
8. Di Tommaso P, Chatzou M, Floden EW, Barja PP, Palumbo E, Notredame C. Nextflow Enables Reproducible Computational Workflows. Nature Biotechnology 2017 Apr;35(4):316–319.
9. Köster J, Rahmann S. Snakemake—a Scalable Bioinformatics Workflow Engine. Bioinformatics 2012 Oct;28(19):2520–2522.
10. Voss K, der Auwera GV, Gentry J. <p>Full-stack Genomics Pipelining with GATK4 + WDL + Cromwell</P>. F1000Research 2017 Aug;6.
11. Sudlow C, Gallacher J, Allen N, Beral V, Burton P, Danesh J, et al. UK Biobank: An Open Access Resource for Identifying the Causes of a Wide Range of Complex Diseases of Middle and Old Age. PLOS Medicine 2015 Mar;12(3):e1001779.
12. Fairley S, Lowy-Gallego E, Perry E, Flicek P. The International Genome Sample Resource (IGSR) Collection of Open Human Genomic Variation Resources. Nucleic Acids Research 2020 Jan;48(D1):D941–D947.
13. Turnbull C, Scott RH, Thomas E, Jones L, Murugaesu N, Pretty FB, et al. The 100 000 Genomes Project: bringing whole genome sequencing to the NHS. BMJ 2018 Apr;361:k1687.
14. Weinstein JN, Collisson EA, Mills GB, Shaw KRM, Ozenberger BA, Ellrott K, et al. The Cancer Genome Atlas Pan-Cancer Analysis Project. Nature Genetics 2013 Oct;45(10):1113–1120.
15. Bailey MH, Tokheim C, Porta-Pardo E, Sengupta S, Bertrand D, Weerasinghe A, et al. Comprehensive Characterization of Cancer Driver Genes and Mutations. Cell 2018 Apr;173(2):371–385.e18.
16. Sanchez-Vega F, Mina M, Armenia J, Chatila WK, Luna A, La KC, et al. Oncogenic Signaling Pathways in The Cancer Genome Atlas. Cell 2018 Apr;173(2):321–337.e10.
17. Ding L, Bailey MH, Porta-Pardo E, Thorsson V, Colaprico A, Bertrand D, et al. Perspective on Oncogenic Processes at the End of the Beginning of Cancer Genomics. Cell 2018 Apr;173(2):305–320.e10.
18. Hoadley KA, Yau C, Hinoue T, Wolf DM, Lazar AJ, Drill E, et al. Cell-of-Origin Patterns Dominate the Molecular Classification of 10,000 Tumors from 33 Types of Cancer. Cell 2018 Apr;173(2):291–304.e6.
19. Weighill D, Ben Guebila M, Glass K, Platig J, Yeh JJ, Quackenbush J. Gene targeting in disease networks. Front Genet 2021 Apr;12:649942.
20. Ellrott K, Bailey MH, Saksena G, Covington KR, Kandoth C, Stewart C, et al. Scalable Open Science Approach for Mutation Calling of Tumor Exomes Using Multiple Genomic Pipelines. Cell Systems 2018 Mar;6(3):271–281.e7.
21. Way GP, Sanchez-Vega F, La K, Armenia J, Chatila WK, Luna A, et al. Machine Learning Detects Pan-cancer Ras Pathway Activation in The Cancer Genome Atlas. Cell Reports 2018 Apr;23(1):172–180.e3.
22. Li J, Lu Y, Akbani R, Ju Z, Roebuck PL, Liu W, et al. TCPA: A Resource for Cancer Functional Proteomics Data. Nature Methods 2013 Nov;10(11):1046–1047.
23. Li J, Akbani R, Zhao W, Lu Y, Weinstein JN, Mills GB, et al. Explore, Visualize, and Analyze Functional Cancer Proteomic Data Using the Cancer Proteome Atlas. Cancer Research 2017 Nov;77(21):e51–e54.
24. Edwards NJ, Oberti M, Thangudu RR, Cai S, McGarvey PB, Jacob S, et al. The CPTAC Data Portal: A Resource for Cancer Proteomics Research. Journal of Proteome Research 2015 Jun;14(6):2707–2713.
25. Ellis MJ, Gillette M, Carr SA, Paulovich AG, Smith RD, Rodland KK, et al. Connecting Genomic Alterations to Cancer Biology with Proteomics: The NCI Clinical Proteomic Tumor Analysis Consortium. Cancer Discovery 2013 Oct;3(10):1108–1112.
26. Cowen L, Ideker T, Raphael BJ, Sharan R. Network Propagation: A Universal Amplifier of Genetic Associations. Nature Reviews Genetics 2017 Sep;18(9):551–562.
27. Sonawane AR, Platig J, Fagny M, Chen CY, Paulson JN, Lopes-Ramos CM, et al. Understanding Tissue-Specific Gene Regulation. Cell reports 2017;21(4):1077–1088.
28. Reyna MA, Haan D, Paczkowska M, Verbeke LPC, Vazquez M, Kahraman A, et al. Pathway and Network Analysis of More than 2500 Whole Cancer Genomes. Nature Communications 2020 Feb;11(1):729.
29. Leiserson MDM, Vandin F, Wu HT, Dobson JR, Eldridge JV, Thomas JL, et al. Pan-Cancer Network Analysis Identifies Combinations of Rare Somatic Mutations across Pathways and Protein Complexes. Nature Genetics 2015 Feb;47(2):106–114.
30. Silverbush D, Cristea S, Yanovich-Arad G, Geiger T, Beerenwinkel N, Sharan R. Simultaneous Integration of Multi-omics Data Improves the Identification of Cancer Driver Modules. Cell Systems 2019 May;8(5):456–466.e5.
31. Belova T, Biondi N, Hsieh PH, Lutsik P, Chudasama P, Kuijjer ML. Heterogeneity in the Gene Regulatory Landscape of Leiomyosarcoma. NAR Cancer 2023 Sep;5(3):zcad037.
32. Lopes-Ramos CM, Kuijjer ML, Ogino S, Fuchs CS, DeMeo DL, Glass K, et al. Gene regulatory network analysis identifies sex-linked differences in colon cancer drug metabolism. Cancer Res 2018 Oct;78(19):5538–5547.
33. Glass K, Huttenhower C, Quackenbush J, Yuan GC. Passing Messages between Biological Networks to Refine Predicted Interactions. PLOS ONE 2013 May;8(5):e64832.
34. Weighill D, Guebila MB, Lopes-Ramos C, Glass K, Quackenbush J, Platig J, et al. Gene Regulatory Network Inference as Relaxed Graph Matching. Proceedings of the AAAI Conference on Artificial Intelligence 2021 May;35(11):10263–10272.
35. Chen C, Padi M, Joint Inference of Transcription Factor Activity and Context-Specific Regulatory Networks. bioRxiv; 2022.
36. Margolin AA, Nemenman I, Basso K, Wiggins C, Stolovitzky G, Favera RD, et al. ARACNE: An Algorithm for the Reconstruction of Gene Regulatory Networks in a Mammalian Cellular Context. BMC Bioinformatics 2006 Mar;7(1):S7.
37. Alvarez MJ, Shen Y, Giorgi FM, Lachmann A, Ding BB, Ye BH, et al. Network-Based Inference of Protein Activity Helps Functionalize the Genetic Landscape of Cancer. Nature genetics 2016 Aug;48(8):838–847.
38. Saha E, Ben Guebila M, Fanfani V, Fischer J, Shutta KH, Mandros P, et al. Gene regulatory networks reveal sex difference in lung adenocarcinoma. Biol Sex Differ 2024 Aug;15(1):62.
39. Shutta KH, Weighill D, Burkholz R, Guebila MB, DeMeo DL, Zacharias HU, et al. DRAGON: Determining Regulatory Associations Using Graphical Models on Multi-Omic Networks. Nucleic Acids Research 2023 Feb;51(3):e15.
40. GenomicDataCommons;. http://bioconductor.org/packages/GenomicDataCo[illegible]
41. Morgan MT, Davis SR, GenomicDataCommons: A Bioconductor Interface to the NCI Genomic Data Commons. bioRxiv; 2017.
42. Colaprico A, Silva TC, Olsen C, Garofano L, Cava C, Garolini D, et al. TCGAbiolinks: An R/Bioconductor Package for Integrative Analysis of TCGA Data. Nucleic Acids Research 2016 May;44(8):e71.
43. Mounir M, Lucchetta M, Silva TC, Olsen C, Bontempi G, Chen X, et al. New Functionalities in the TCGAbiolinks Package for

the Study and Integration of Cancer Data from GDC and GTEx. PLoS computational biology 2019 Mar;15(3):e1006701.
44. Silva TC, Colaprico A, Olsen C, D'Angelo F, Bontempi G, Ceccarelli M, et al., *TCGA Workflow*: Analyze Cancer Genomics and Epigenomics Data Using Bioconductor Packages; 2016.
45. Ben Guebila M, Wang T, Lopes-Ramos CM, Fanfani V, Weighill D, Burkholz R, et al. The Network Zoo: A Multilingual Package for the Inference and Analysis of Gene Regulatory Networks. Genome Biology 2023 Mar;24(1):45.
46. Padi M, Quackenbush J. Detecting phenotype-driven transitions in regulatory network structure. NPJ systems biology and applications 2018;4(1):16.
47. Kuijjer ML, Tung MG, Yuan G, Quackenbush J, Glass K. Estimating Sample-Specific Regulatory Networks. iScience 2019 Apr;14:226–240.
48. Guinney J, Dienstmann R, Wang X, de Reyniès A, Schlicker A, Soneson C, et al. The Consensus Molecular Subtypes of Colorectal Cancer. Nature Medicine 2015 Nov;21(11):1350–1356.
49. Ben Guebila M, Lopes-Ramos CM, Weighill D, Sonawane AR, Burkholz R, Shamsaei B, et al. GRAND: A Database of Gene Regulatory Network Models across Human Conditions. Nucleic Acids Research 2022 Jan;50(D1):D610–D621.
50. Merkel D. Docker: Lightweight Linux Containers for Consistent Development and Deployment. Linux journal 2014;2014(239):2.
51. Kurtzer GM, Sochat V, Bauer MW. Singularity: Scientific Containers for Mobility of Compute. PLOS ONE 2017 May;12(5):e0177459.
52. Anaconda Software Distribution. Anaconda Inc.; 2020.
53. Grossman Robert L , Heath Allison P , Ferretti Vincent, Varmus Harold E , Lowy Douglas R , Kibbe Warren A , et al. Toward a Shared Vision for Cancer Genomic Data. New England Journal of Medicine 2016;375(12):1109–1112.
54. Wilks C, Zheng SC, Chen FY, Charles R, Solomon B, Ling JP, et al. Recount3: Summaries and Queries for Large-Scale RNA-seq Expression and Splicing. Genome biology 2021;.
55. THE GTEX CONSORTIUM. The GTEx Consortium Atlas of Genetic Regulatory Effects across Human Tissues. Science 2020 Sep;369(6509):1318–1330.
56. Arora S, Pattwell SS, Holland EC, Bolouri H, Uncertainty in RNA-seq Gene Expression Data; 2018.
57. Johnson KA, Krishnan A. Robust Normalization and Transformation Techniques for Constructing Gene Coexpression Networks from RNA-seq Data. Genome Biology 2022 Jan;23(1):1.
58. Collado-Torres L, Nellore A, Kammers K, Ellis SE, Taub MA, Hansen KD, et al. Reproducible RNA-seq Analysis Using Recount2. Nature Biotechnology 2017 Apr;35(4):319–321.
59. Li B, Ruotti V, Stewart RM, Thomson JA, Dewey CN. RNA-Seq Gene Expression Estimation with Read Mapping Uncertainty. Bioinformatics 2010 Feb;26(4):493–500.
60. Robinson MD, McCarthy DJ, Smyth GK. edgeR: A Bioconductor Package for Differential Expression Analysis of Digital Gene Expression Data. Bioinformatics 2010 Jan;26(1):139–140.
61. Chen Y, Chen L, Lun ATL, Baldoni PL, Smyth GK, edgeR 4.0: Powerful Differential Analysis of Sequencing Data with Expanded Functionality and Improved Support for Small Counts and Larger Datasets. bioRxiv; 2024.
62. Johnson WE, Li C, Rabinovic A. Adjusting Batch Effects in Microarray Expression Data Using Empirical Bayes Methods. Biostatistics (Oxford, England) 2007 Jan;8(1):118–127.
63. Leek JT, Johnson WE, Parker HS, Jaffe AE, Storey JD. The Sva Package for Removing Batch Effects and Other Unwanted Variation in High-Throughput Experiments. Bioinformatics 2012 Mar;28(6):882–883.
64. Aran D, Sirota M, Butte AJ. Systematic Pan-Cancer Analysis of Tumour Purity. Nature Communications 2015 Dec;6(1):8971.
65. Du P, Zhang X, Huang CC, Jafari N, Kibbe WA, Hou L, et al. Comparison of Beta-value and M-value Methods for Quantifying Methylation Levels by Microarray Analysis. BMC bioinformatics 2010;11:1–9.
66. Liu H, Lafferty J, Wasserman L. The Nonparanormal: Semiparametric Estimation of High Dimensional Undirected Graphs. Journal of Machine Learning Research 2009;10(10).
67. Zhao T, Liu H, Roeder K, Lafferty J, Wasserman L. The Huge Package for High-Dimensional Undirected Graph Estimation in R. The Journal of Machine Learning Research 2012;13(1):1059–1062.
68. Martin FJ, Amode MR, Aneja A, Austine-Orimoloye O, Azov AG, Barnes I, et al. Ensembl 2023. Nucleic Acids Research 2023 Jan;51(D1):D933–D941.
69. Seal RL, Braschi B, Gray K, Jones TEM, Tweedie S, Haim-Vilmovsky L, et al. Genenames.Org: The HGNC Resources in 2023. Nucleic Acids Research 2023 Jan;51(D1):D1003–D1009.
70. Brown GR, Hem V, Katz KS, Ovetsky M, Wallin C, Ermolaeva O, et al. Gene: A Gene-Centered Information Resource at NCBI. Nucleic Acids Research 2015 Jan;43(Database issue):D36–42.
71. Maglott D, Ostell J, Pruitt KD, Tatusova T. Entrez Gene: Gene-Centered Information at NCBI. Nucleic Acids Research 2011 Jan;39(suppl_1):D52–D57.
72. Frankish A, Diekhans M, Jungreis I, Lagarde J, Loveland JE, Mudge JM, et al. GENCODE 2021. Nucleic Acids Research 2021 Jan;49(D1):D916–D923.
73. AnnotationDbi;. http://bioconductor.org/packages/AnnotationDbi/.
74. Du P, Zhang X, Huang CC, Jafari N, Kibbe WA, Hou L, et al. Comparison of Beta-value and M-value Methods for Quantifying Methylation Levels by Microarray Analysis. BMC Bioinformatics 2010 Nov;11(1):587.
75. Ferlay J, Ervik M, Lam F, Colombet M, Mery L, Piñeros M, et al. Global Cancer Observatory: Cancer Today. Lyon, France: international agency for research on cancer 2024;Available from: https://gco.iarc.who.int/today, accessed [19 June 2024].(0):0.
76. Marisa L, Blum Y, Taieb J, Ayadi M, Pilati C, Le Malicot K, et al. Intratumor CMS Heterogeneity Impacts Patient Prognosis in Localized Colon Cancer. Clinical Cancer Research 2021 Sep;27(17):4768–4780.
77. Mouillet-Richard S, Cazelles A, Sroussi M, Gallois C, Taieb J, Laurent-Puig P. Clinical Challenges of Consensus Molecular Subtype CMS4 Colon Cancer in the Era of Precision Medicine. Clinical Cancer Research 2024 Apr;p. OF1–OF8.
78. OncoKB: A Precision Oncology Knowledge Base | JCO Precision Oncology;. https://ascopubs.org/doi/full/10.1200/PO.17.00011.
79. Mattei AL, Bailly N, Meissner A. DNA Methylation: A Historical Perspective. Trends in Genetics 2022 Jul;38(7):676–707.
80. Chakravarty D, Gao J, Phillips S, Kundra R, Zhang H, Wang J, et al. OncoKB: A Precision Oncology Knowledge Base. JCO Precision Oncology 2017 Dec;(1):1–16.
81. Suehnholz SP, Nissan MH, Zhang H, Kundra R, Nandakumar S, Lu C, et al. Quantifying the Expanding Landscape of Clinical Actionability for Patients with Cancer. Cancer Discovery 2024 Jan;14(1):49–65.
82. Piñero J, Ramírez-Anguita JM, Saüch-Pitarch J, Ronzano F, Centeno E, Sanz F, et al. The DisGeNET knowledge platform for disease genomics: 2019 update. Nucleic Acids Research 2019 11;48(D1):D845–D855. https://doi.org/10.1093/nar/gkz1021.
83. Sondka Z, Bamford S, Cole CG, Ward SA, Dunham I, Forbes SA. The COSMIC Cancer Gene Census: Describing Genetic Dysfunction across All Human Cancers. Nature Reviews Cancer 2018 Nov;18(11):696–705.
84. Tate JG, Bamford S, Jubb HC, Sondka Z, Beare DM, Bindal N, et al. COSMIC: the Catalogue Of Somatic Mutations In Cancer. Nucleic Acids Research 2018 10;47(D1):D941–D947. https://doi.org/10.1093/nar/gky1015.
85. Tan SH, Nevalainen MT. Signal Transducer and Activator of Transcription 5A/B in Prostate and Breast Cancers. Endocrine-

Related Cancer 2008 Jun;15(2):367–390.
86. Haddad BR, Gu L, Mirtti T, Dagvadorj A, Vogiatzi P, Hoang DT, et al. STAT5A/B Gene Locus Undergoes Amplification during Human Prostate Cancer Progression. The American Journal of Pathology 2013 Jun;182(6):2264–2275.
87. Di Palma T, Lucci V, de Cristofaro T, Filippone MG, Zannini M. A Role for PAX8 in the Tumorigenic Phenotype of Ovarian Cancer Cells. BMC cancer 2014 Apr;14:292.
88. Jesse S, Koenig A, Ellenrieder V, Menke A. Lef-1 Isoforms Regulate Different Target Genes and Reduce Cellular Adhesion. International Journal of Cancer 2010 Mar;126(5):1109–1120.
89. Chiang YT, Wang K, Fazli L, Qi RZ, Gleave ME, Collins CC, et al. GATA2 as a Potential Metastasis-Driving Gene in Prostate Cancer. Oncotarget 2014 Jan;5(2):451–461.
90. Mellor P, Deibert L, Calvert B, Bonham K, Carlsen SA, Anderson DH. CREB3L1 Is a Metastasis Suppressor That Represses Expression of Genes Regulating Metastasis, Invasion, and Angiogenesis. Molecular and Cellular Biology 2013 Dec;33(24):4985–4995.
91. Heide T, Househam J, Cresswell GD, Spiteri I, Lynn C, Mossner M, et al. The Co-Evolution of the Genome and Epigenome in Colorectal Cancer. Nature 2022 Nov;611(7937):733–743.
92. Ma XD, Xu SD, Hao SH, Han K, Chen JW, Ling H, et al. KLF16 Enhances Stress Tolerance of Colorectal Carcinomas by Modulating Nucleolar Homeostasis and Translational Reprogramming. Molecular Therapy 2022 Aug;30(8):2828–2843.
93. Kurani H, Slingerland JM. DOT1L Mediates Stem Cell Maintenance and Represents a Therapeutic Vulnerability in Cancer. Cancer Research 2025 Mar;85(5):838–847.
94. Yang B, Tang H, Wang N, Gu J, Wang Q. Targeted DNA Demethylation of the ZNF334 Promoter Inhibits Colorectal Cancer Growth. Cell Death & Disease 2023 Mar;14(3):1–10.
95. Kim HM, Kang B, Park S, Park H, Kim CJ, Lee H, et al. Forkhead Box Protein D2 Suppresses Colorectal Cancer by Reprogramming Enhancer Interactions. Nucleic Acids Research 2023 Jul;51(12):6143–6155.
96. Li K, Guo Q, Yang J, Chen H, Hu K, Zhao J, et al. FOXD3 Is a Tumor Suppressor of Colon Cancer by Inhibiting EGFR-Ras-Raf-MEK-ERK Signal Pathway. Oncotarget 2016 Dec;8(3):5048–5056.
97. Shan TD, Xu JH, Yu T, Li JY, Zhao LN, Ouyang H, et al. Knockdown of Linc-POU3F3 Suppresses the Proliferation, Apoptosis, and Migration Resistance of Colorectal Cancer. Oncotarget 2015 Oct;7(1):961–975.
98. Lee YJ, Lee EY, Choi BH, Jang H, Myung JK, You HJ. The Role of Nuclear Receptor Subfamily 1 Group H Member 4 (NR1H4) in Colon Cancer Cell Survival through the Regulation of c-Myc Stability. Molecules and Cells 2020 May;43(5):459–468.
99. Shah R, Jones E, Vidart V, Kuppen PJK, Conti JA, Francis NK. Biomarkers for Early Detection of Colorectal Cancer and Polyps: Systematic Review. Cancer Epidemiology, Biomarkers & Prevention 2014 Sep;23(9):1712–1728.
100. Béziat V, Fieschi C, Momenilandi M, Migaud M, Belaid B, Djidjik R, et al. Inherited Human ZNF341 Deficiency. Current opinion in immunology 2023 Jun;82:102326.
101. Hay Fever and Asthma as Markers of Atopic Immune Response and Risk of Colorectal Cancer in Three Large Cohort Studies | Cancer Epidemiology, Biomarkers & Prevention | American Association for Cancer Research;. https://aacrjournals.org/cebp/article/22/4/661/69817/Hay-Fever-and-Asthma-as-Markers-of-Atopic-Immune.
102. Bayesian Inference of Sample-Specific Coexpression Networks;. https://genome.cshlp.org/content/34/9/1397.
103. Osorio D, Capasso A, Eckhardt SG, Giri U, Somma A, Pitts TM, et al. Population-Level Comparisons of Gene Regulatory Networks Modeled on High-Throughput Single-Cell Transcriptomics Data. Nature Computational Science 2024 Mar;4(3):237–250.
104. Fang Z, Liu X, Peltz G. GSEApy: a comprehensive package for performing gene set enrichment analysis in Python. Bioinformatics 2022 11;39(1):btac757. https://doi.org/10.1093/bioinformatics/btac757.
105. Grant CE, Bailey TL, Noble WS. FIMO: scanning for occurrences of a given motif. Bioinformatics 2011;27(7):1017–1018.
106. Szklarczyk D, Gable AL, Nastou KC, Lyon D, Kirsch R, Pyysalo S, et al. The STRING database in 2021: customizable protein–protein networks, and functional characterization of user-uploaded gene/measurement sets. Nucleic acids research 2021;49(D1):D605–D612.

Supplementary

Quickstart

Here is a short guide on how to start running the workflow and where to find all the relevant resources. However, we always recommend to read and follow the updated documentation that is at `https://github.com/QuackenbushLab/tcga-data-nf.git`

Relevant resources and links

- **Supplementary code**: We keep all the companion data and configuration files for the paper in the the `tcga-data-supplement` repository `https://github.com/QuackenbushLab/tcga-data-supplement`. You can find all the code for the colon cancer analysis and find all links and instructions for the precomputed GRNs of common cancer types.
- **Supplementary data**: all data used and generated for this paper is hosted on the Harvard Dataverse `https://doi.org/10.7910/DVN/MCSSYJ`
- **Docker**:The docker container is hosted on docker.io at `https://hub.docker.com/r/violafanfani/tcga-data-nf`
- **Nextflow**: Nextflow.io hosts extensive documentation on the language and the best practices to build a workflow `https://nextflow.io/docs/latest/index.html`

Getting started

Here are the main steps required to run the workflow. Please refer to the official and updated documentation for a more comprehensive explanation.

1. Install Nextflow on your machine, you can follow the instructions on their website[1].
2. Pull the workflow: `nextflow pull QuackenbushLab/tcga-data-nf`
3. Install and pull the docker/singularity container or conda to run the whole pipeline. Details can be found in the documentation[2]
4. Run some test workflows:
   - test the download: `nextflow rurn QuackenbushLab/tcga-data-nf -profile <docker/conda>,testDownload`
   - test the prepare: `nextflow run QuackenbushLab/tcga-data-nf -profile <docker/conda>,testPrepare`
   - test the analyze: `nextflow run QuackenbushLab/tcga-data-nf -profile <docker/conda>,testAnalyze`
   - test the full workflow: `nextflow run QuackenbushLab/tcga-data-nf -profile <docker/conda>,test`

Configuration

The whole workflow can be personalized by changing the configuration parameters inside a `my-config.conf` file, that is then added to the workflow run as follows:

‘`nextflow run QuackenbushLab/tcga-data-nf -c my-config.conf`‘

First there are three main parameters that need to be passed by the user:

- `resultsDir = "results"`: general folder under which you want to find the results. This can directly reference an AWS S3 bucket
- `batchName = "my-batch"`: name of the run, this is gonna create a subfolder where the results are stored.
- `pipeline = ’download’`: name of the pipeline, one of download,prepare,analyze,full.

This way all data generated by the pipeline will be found inside the `resultsDir/batchName/` folder. If nothing is passed, all results will be in the `results/my-batch` folder. One of the advantages of Nextflow is the consistent organization of the results folder. For an updated and complete description of the result folders, please refer to the documentation[3]

For a full list of the configuration parameters check the docs[4].

1 `https://www.nextflow.io/docs/latest/install.html`
2 `https://github.com/QuackenbushLab/tcga-data-nf/blob/main/docs.md#conda`
3 `https://github.com/QuackenbushLab/tcga-data-nf/blob/main/docs.md#result-folders`
4 `https://github.com/QuackenbushLab/tcga-data-nf/blob/main/docs.md#configurations`

Download

Listing 1. download-test.json, Example of a json configuration file for the download step. With this file the pipeline downloads all modalities for TCGA LUAD and gtex lung.


```
{
    "expression_recount3": {
        "tcga_luad": {
            "project": "LUAD",
            "project_home" : "data_sources/tcga",
            "organism" : "human",
            "annotation" : "gencode_v26",
            "type" : "gene",
            "samples" : "NA"
        },
        "gtex_lung": {
                "project": "LUNG",
                "project_home" : "data_sources/gtex",
                "organism" : "human",
                "annotation" : "gencode_v26",
                "type" : "gene",
                "samples" : "NA"
              }
        },
        "mutation_tcgabiolinks":{
          "tcga_luad":{
            "project" : "TCGA-LUAD",
            "data_category" : "Simple Nucleotide Variation",
            "data_type" : "Masked Somatic Mutation",
            "download_dir" : "gdc_tcga_mutation",
            "samples" : "NA"
          }},
        "clinical_tcgabiolinks": {
          "tcga_luad":{
            "project" : "TCGA-LUAD",
            "data_category" : "Clinical",
            "data_type" : "Clinical Supplement",
            "data_format" : "BCR Biotab"
            }},
        "methylation_gdc":{
          "tcga_luad":{
            "project" : "TCGA-LUAD",
            "gdc_type" : "methylation_beta_value",
            "gdc_platform" : "illumina human methylation 450",
            "download_dir" : "gdc_tcga_methylation",
            "samples" : "NA"
          }
        }
    }
```

Listing 2. full-test.json, Example of a json configuration file for the full pipeline. With this file we specify which modalities and samples need to be downloaded, pre-processed and analyzed. In this case we are interested in LUAD samples.

```
{ "tcga_luad": {
        "expression_recount3": {
              "project" : "LUAD",
              "project_home" : "data_sources/tcga",
              "organism" : "human",
              "annotation" : "gencode_v26",
              "type" : "gene",
              "samples" : "testdata/tcga_luad_samples.txt"
            },
      "mutation_tcgabiolinks": {
              "project" : "TCGA-LUAD",
              "data_category" : "Simple Nucleotide Variation",
              "data_type" : "Masked Somatic Mutation",
              "download_dir" : "gdc_tcga_mutation",
              "samples" : "testdata/tcga_luad_mutation_samples.txt"
            },
      "clinical_tcgabiolinks": {
              "project" : "TCGA-LUAD",
              "data_category" : "Clinical",
              "data_type" : "Clinical Supplement",
              "data_format" : "BCR Biotab"
              },
      "methylation_gdc": {
              "project" : "TCGA-LUAD",
              "gdc_type" : "methylation_beta_value",
              "gdc_platform" : "illumina human methylation 450",
              "download_dir" : "gdc_tcga_methylation",
              "samples" : "testdata/tcga_luad_samples.txt"
            }
      }
    }
```

Supplementary Figures

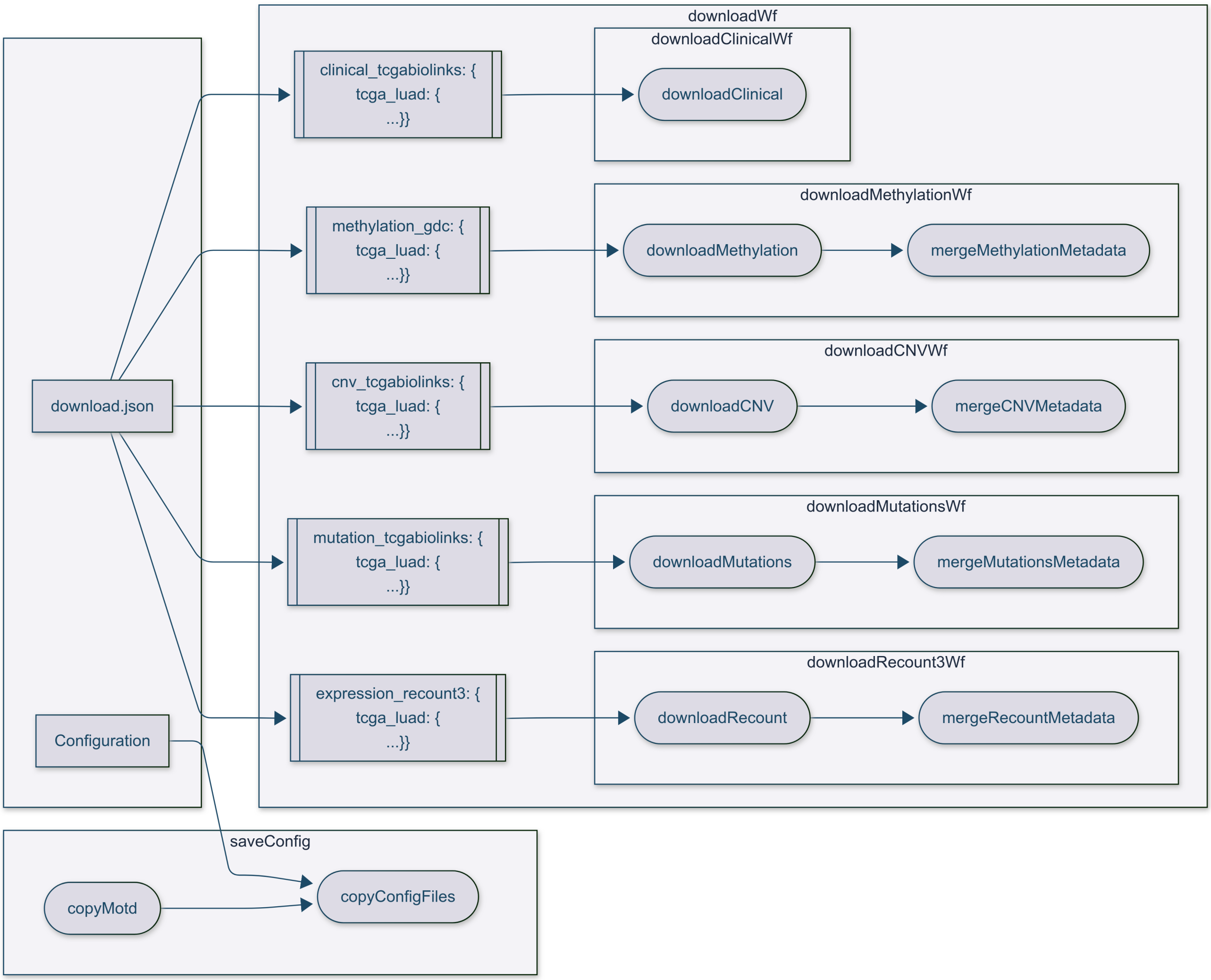

Figure S1. Download. Directed Acyclic Graph of the processes specified in the **Download** pipeline. For each modality that is specified in the configuration file, *tcga-data-nf* downloads the data and generates metadata tables with the names, paths, and parameters of the files. Whenever *tcga-data-nf* is run, we also generate and save the configuration parameters which can be then examined and reused (saveConfig process).

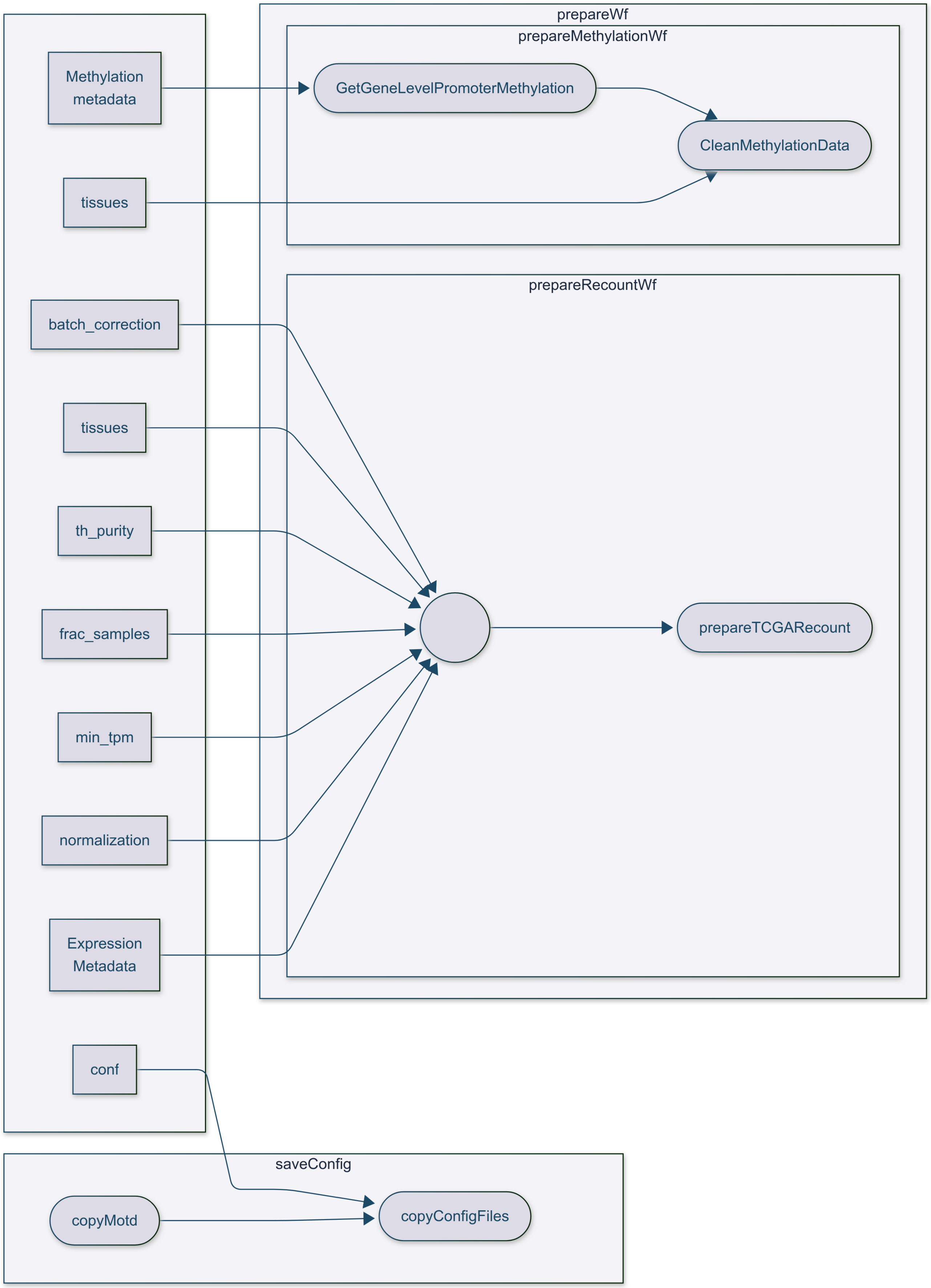

Figure S2. Prepare. Directed Acyclic Graph of the processes specified in the **Prepare** pipeline. The expression and methylation data specified in the configuration metadata is processed using the combination of all input parameters (tissues, purity, minTPM...). Whenever *tcga-data-nf* is run, we also generate and save the configuration parameters which can be then examined and reused (saveConfig process).

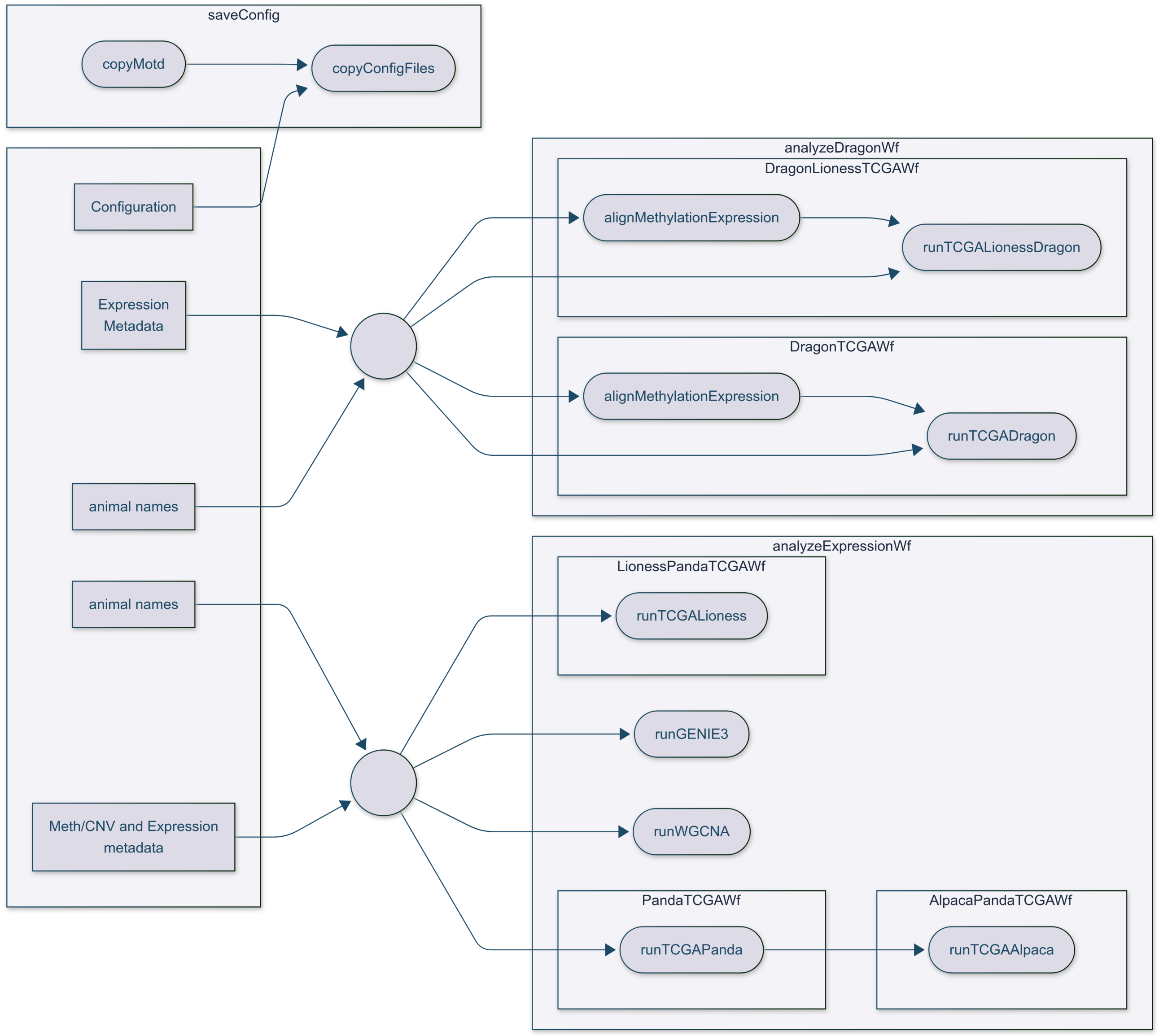

Figure S3. Analyze. Directed Acyclic Graph of the processes specified in the **Analyze** pipeline. Using the input metadata, the *tcga-data-nf* workflow generates PANDA, DRAGON, LIONESS, GENIE3, WGCNA networks, and matches them with log files and intermediate tables, useful for further investigation of the results. PANDA networks are compared with ALPACA. Whenever *tcga-data-nf* is run, we also generate and save the configuration parameters which can be then examined and reused (saveConfig process).

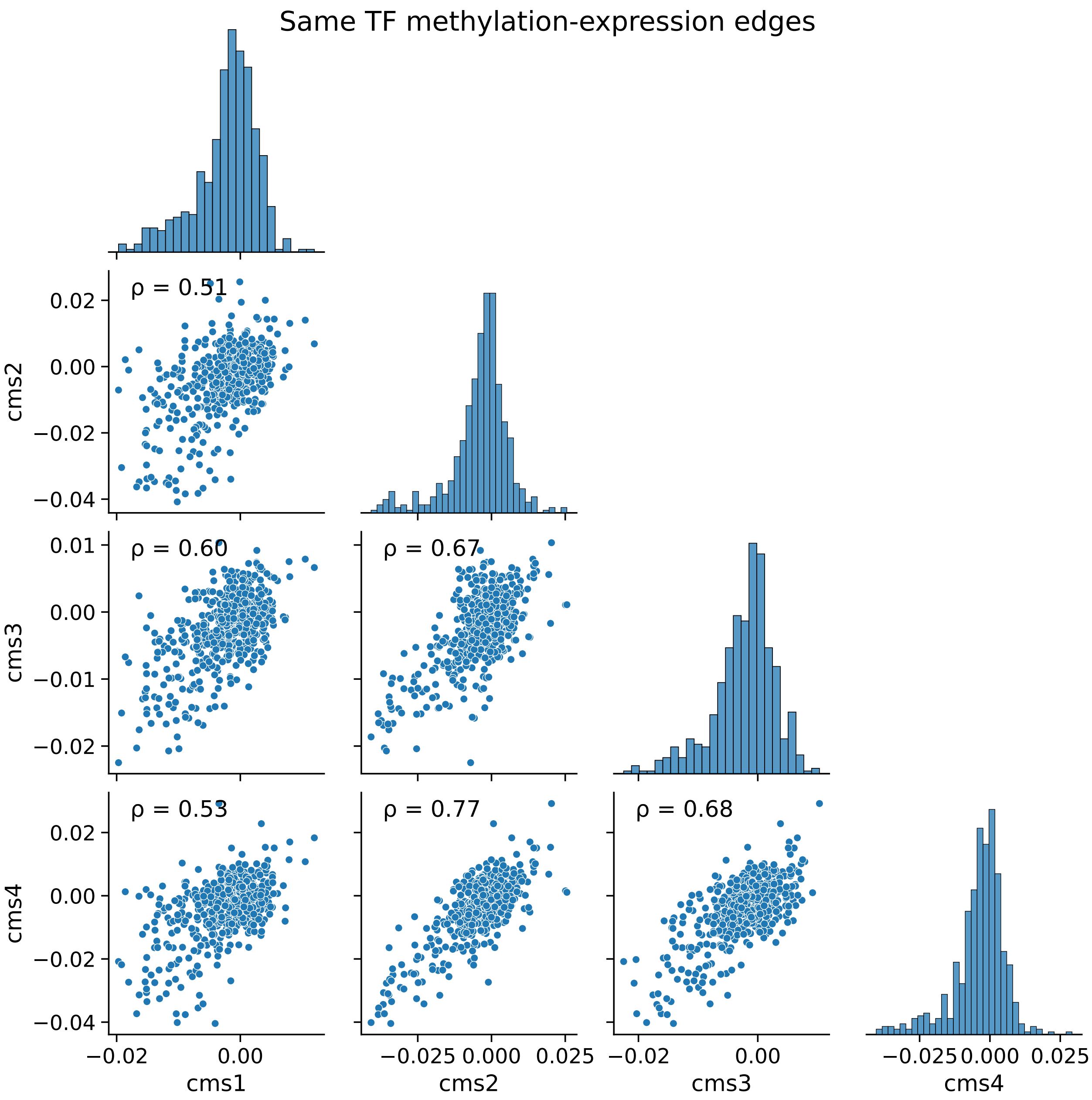

Figure S4. Correlation between DRAGON methylation-expression edges on the the same TFs. For all (M_i, E_i) edges we plot their distribution in each subtype (histograms on the diagonal) and the correlation of the edge weights between each pair of subtypes. While all Pearson correlation values are above 0.50 it is worth noting that CMS2 and CMS4 are the most similar to each other with $\rho = 0.77$. This shows that many of the TFs for which we have evidence of negative partial correlations between methylation and expression are conserved across the CMS2 and CMS4 subtypes, while being more different for CMS1.

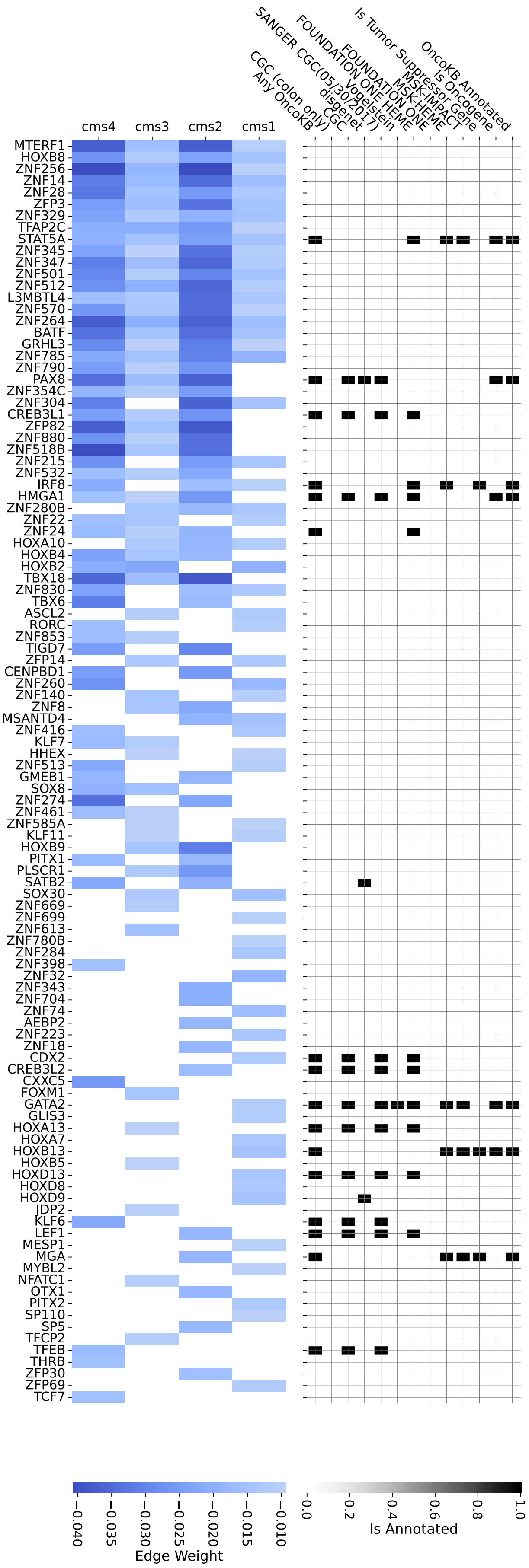

Figure S5. TFs with evidence of epigenetic effect on expression. From all (M_i, E_i) edges, for each subtype, we select those whose values is in the first decile of the distribution, that is the smallest 10% of edges. Here we plot the edge value on the left and the annotation to one of each TF to a cancer related database. In particular OncoKB (downloaded 12/13/2022), Cancer Gene Census (v101, downloaded 04/25/2025), and DisGeNet (GDA CURATED C0009404, downloaded on 04/25/2025)

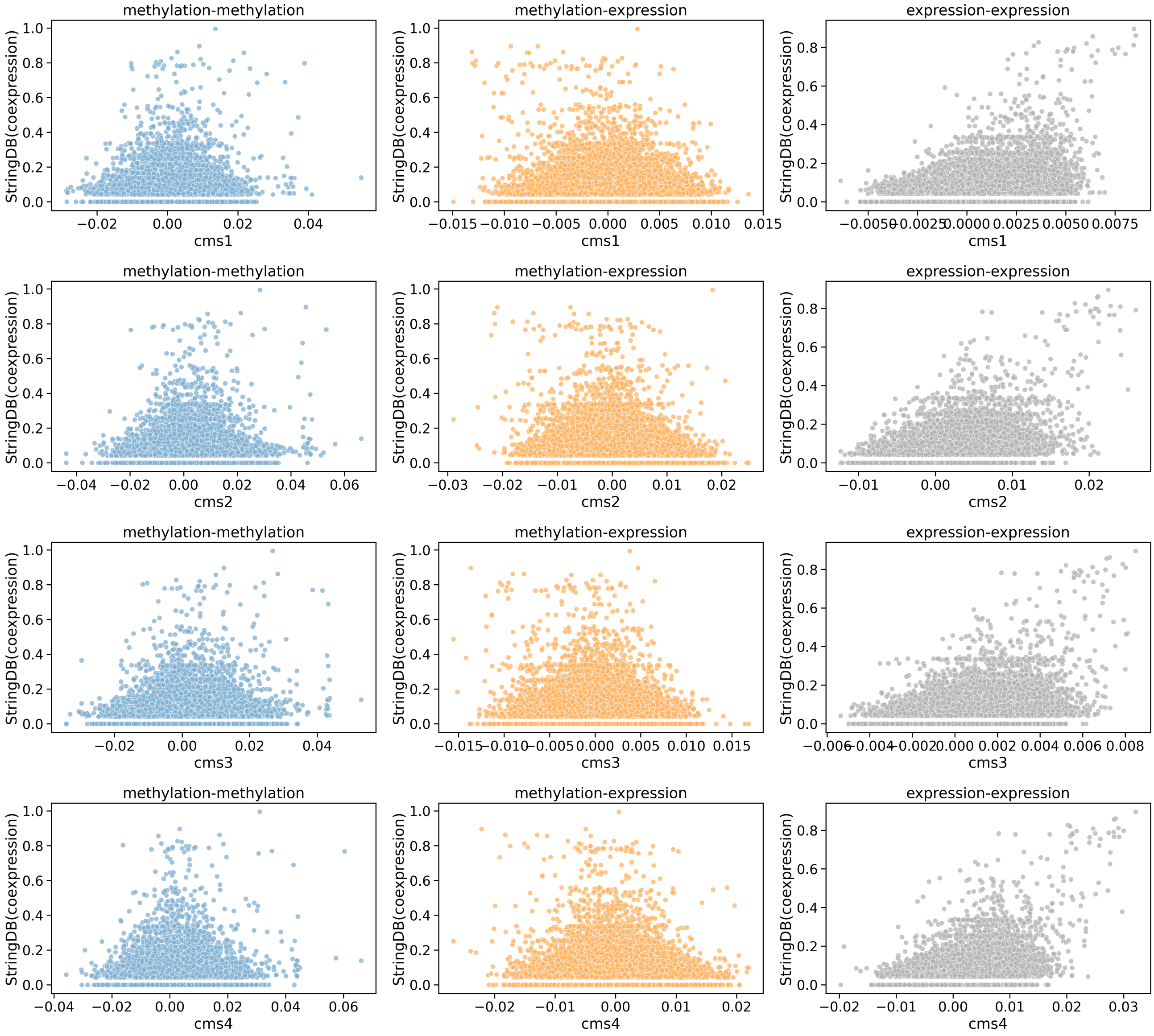

Figure S6. Relationship between DRAGON edges and StringDB co-expression evidence. For each colon cancer subtype (rows), we plot the values of the DRAGON edges on the x-axis, grouped by edge type (columns) and the confidence score in the StringDB database on the y-axis. For all subtypes there is evidence of some correlation between the DRAGON and StringDB co-expression, confirming the validity of our inferred associations, while methylation-methylation and methylation-expression edges serve as negative controls.

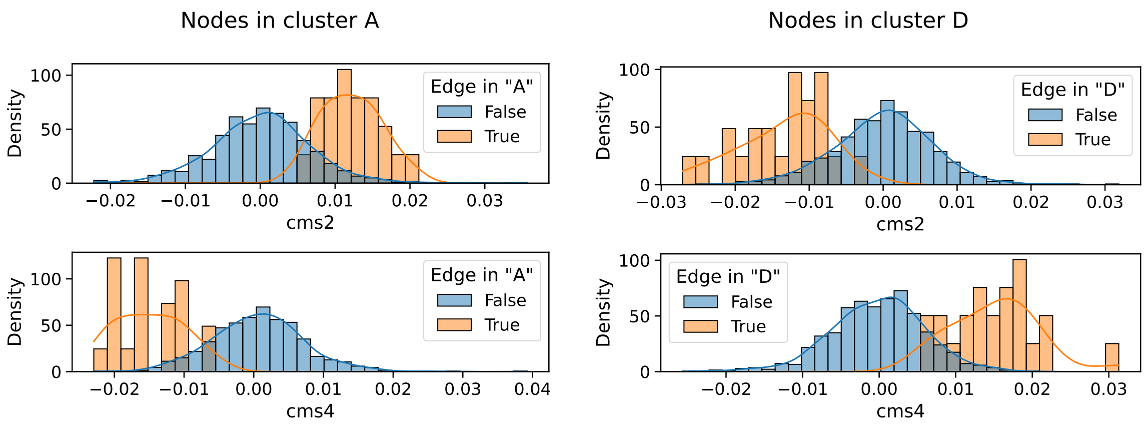

Figure S7. Edge weights for nodes in Cluster A and D of the DRAGON networks. We select first the subgraphs with all the nodes in each cluster, it is worth noting that these graphs contains both the edges represented in Figure 2B, and those that connect the nodes in the cluster but were not the strongest edges. We compare the values of the edges of interest (orange), that are those shown in Figure 2B, and the rest of the edges (blue) that connect the same TFs, as controls. We observe that for both groups the average edge value is around 0, meaning that there is not a general difference between the edge values of the two subtypes, while the edges that were selected in the clusters swap between higher and lower values for CMS2 and CMS4.

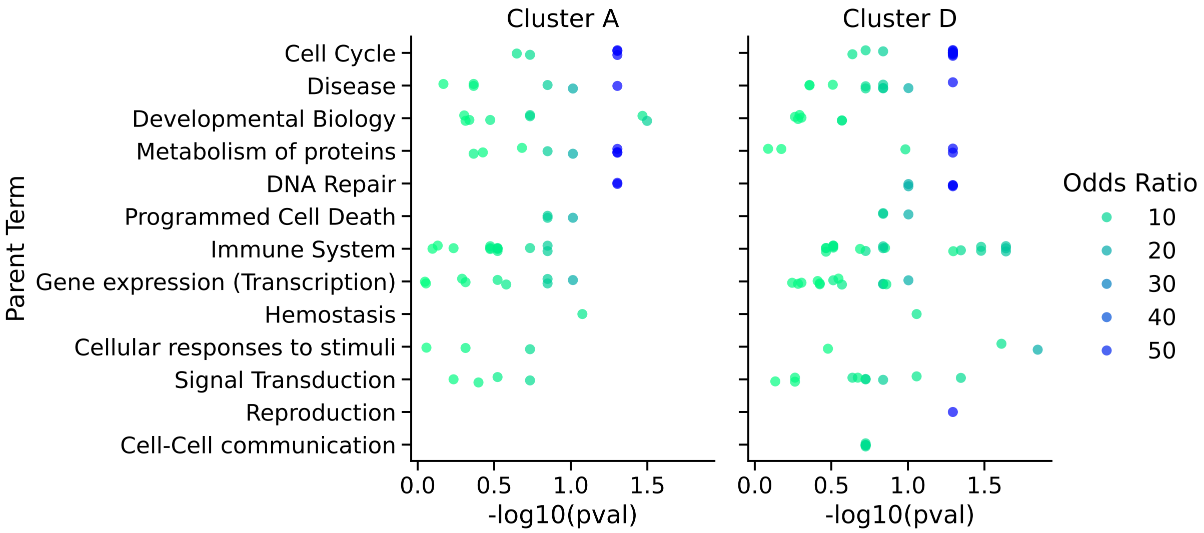

Figure S8. Reactome pathways for clusters A and D. Using the clusters found from the DRAGON edges, we run a pathway over-representation analysis of the TFs in both cluster A and D. With REACTOME we are able to identify the general pathway to which each term belongs to. For each "parent"" pathway (y-axis), we plot the $-log_{10}(pvalue)$ (x-axis) of all the pathways tested that belong to that parent term, and we color them by the corresponding Odds-Ratio. Since pathway analysis on TFs is challenging (there are only ~ 1000 TFs and many of them are annotated only to the general transcriptional pathway terms) we report here all results, even those that are not significant, such that one can observe the general trend. TFs in cluster D seems to be more consistently annotated to Immune System pathways, while the TFs in cluster A have some stronger terms related to Developmental Biology.

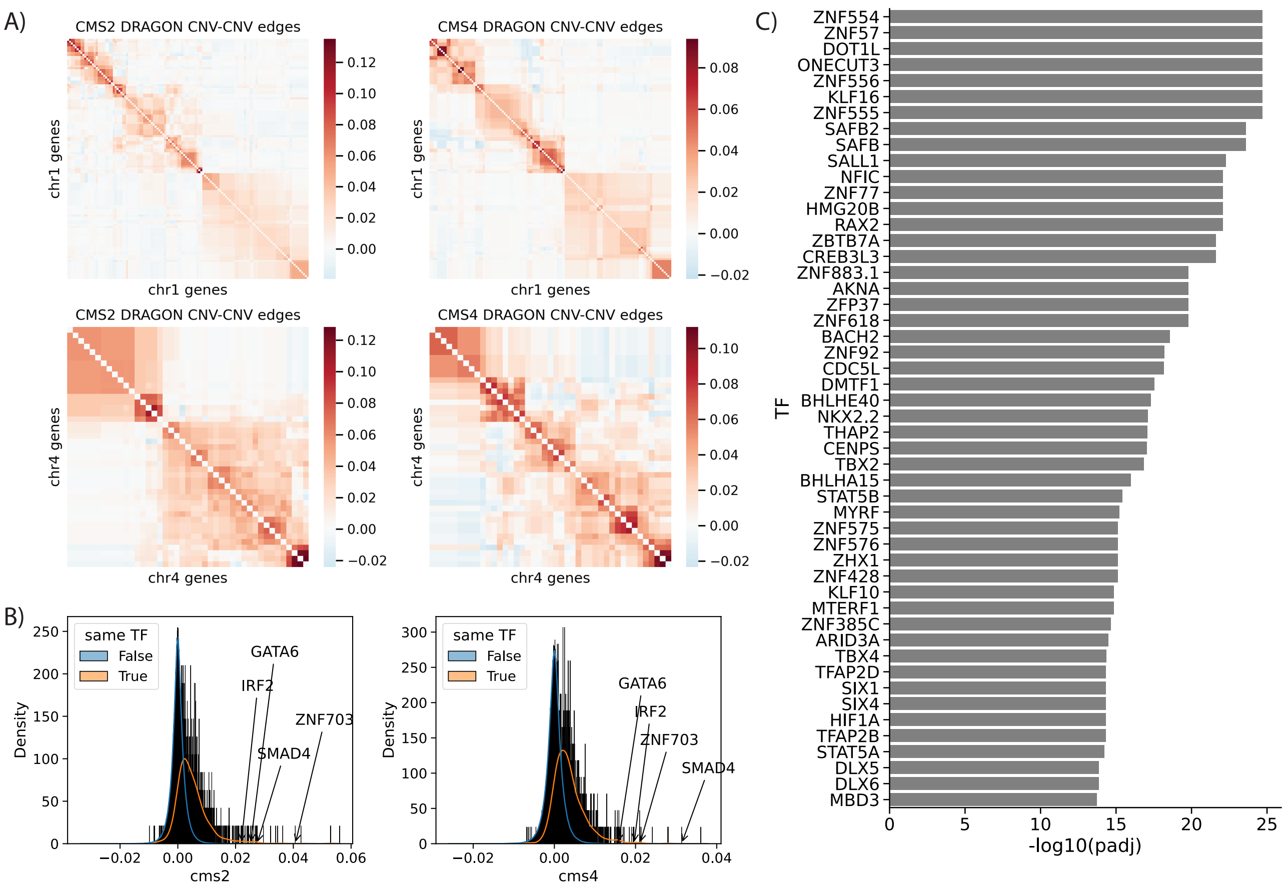

Figure S9. DRAGON CNV-expression networks. A) DRAGON CNV-CNV edges for genes in chromosome 1 and 4. For both CMS2 and CMS4 there is evidence of a block structure that depends on the genome location. Adjacent genes are more likely to be correlated. B) Distribution of partial correlation values between CNV and expression of TFs in both subtypes. In orange we show the values for the edges of the same TF $((C_i, E_i))$, while in blue we show all the others $((C_i, E_j))$. As expected, CNV and expression tend to be positively correlated. The histogram represents the distribution density and it is normalized per subtype and per group. We have also annotated the TFs that are in the strongest 20 by mean value, in both subtypes, that are also known for their role in cancer. C) TFs with CNV-expression edges that are significantly different between CMS2 and CMS4. We show the 50 with the lowest adjusted p value (Benjiamini-Hochberg FDR) from the paired Wilkoxon signed-rank test.

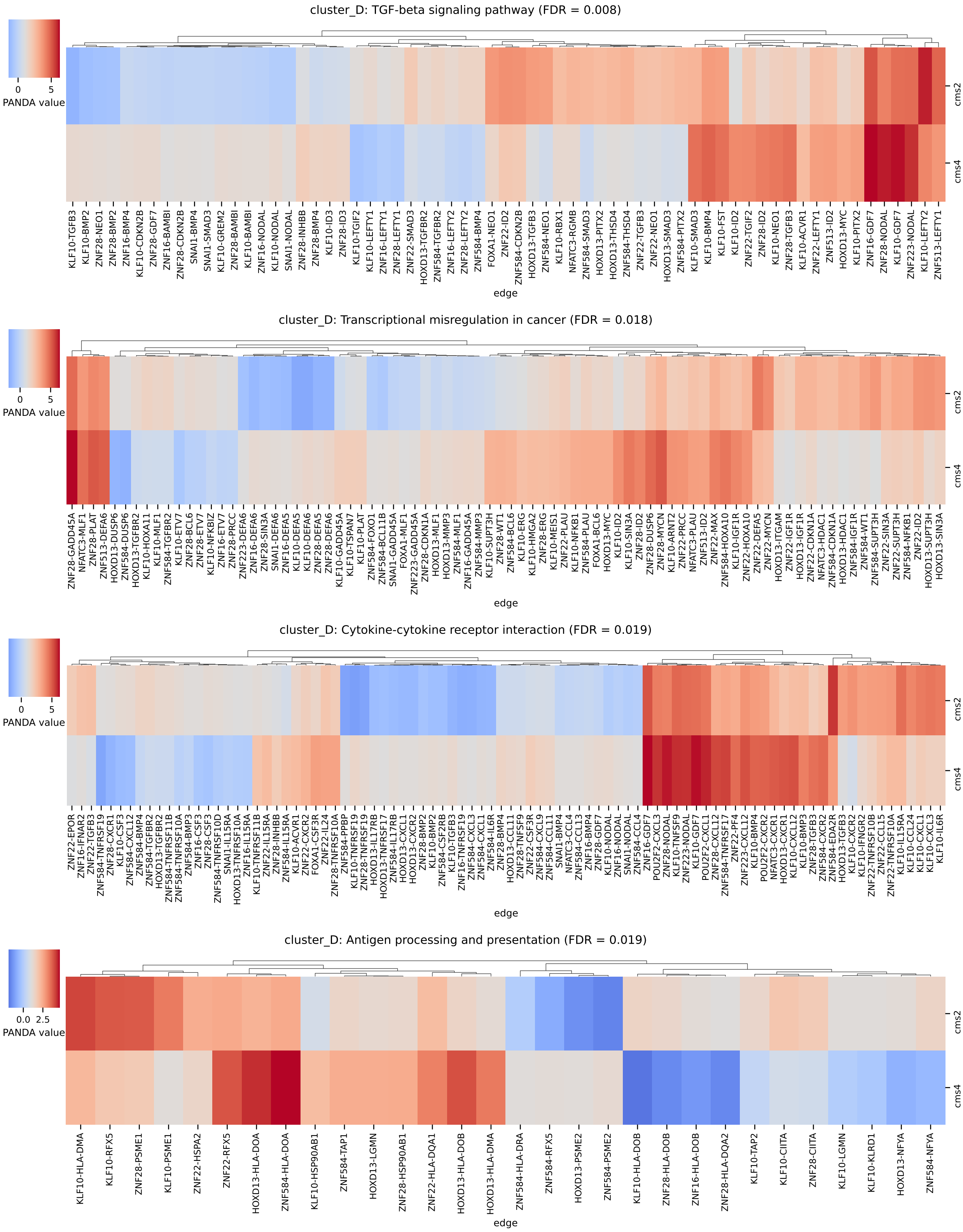

Figure S10. PANDA edges involved in the main pahtways targeted by cluster D. We selected the regulatory edges of the TFs in the cluster D (defined by the analysis on DRAGON networks) and we investigated which edges in the PANDA networks underwent the biggest changes between CMS2 and CMS4. The target genes of the edges were found to be preferentially involved in the TGF-beta signaling pathway, Transcriptional misregulation in cancer, Cytokine-cytokine receptor interaction and Antigen processing anr presentation. Here, we represent the PANDA edges (edge weight represented by different colors) connecting the main targets for each pathway, for both CMS2 and CMS4 (rows).

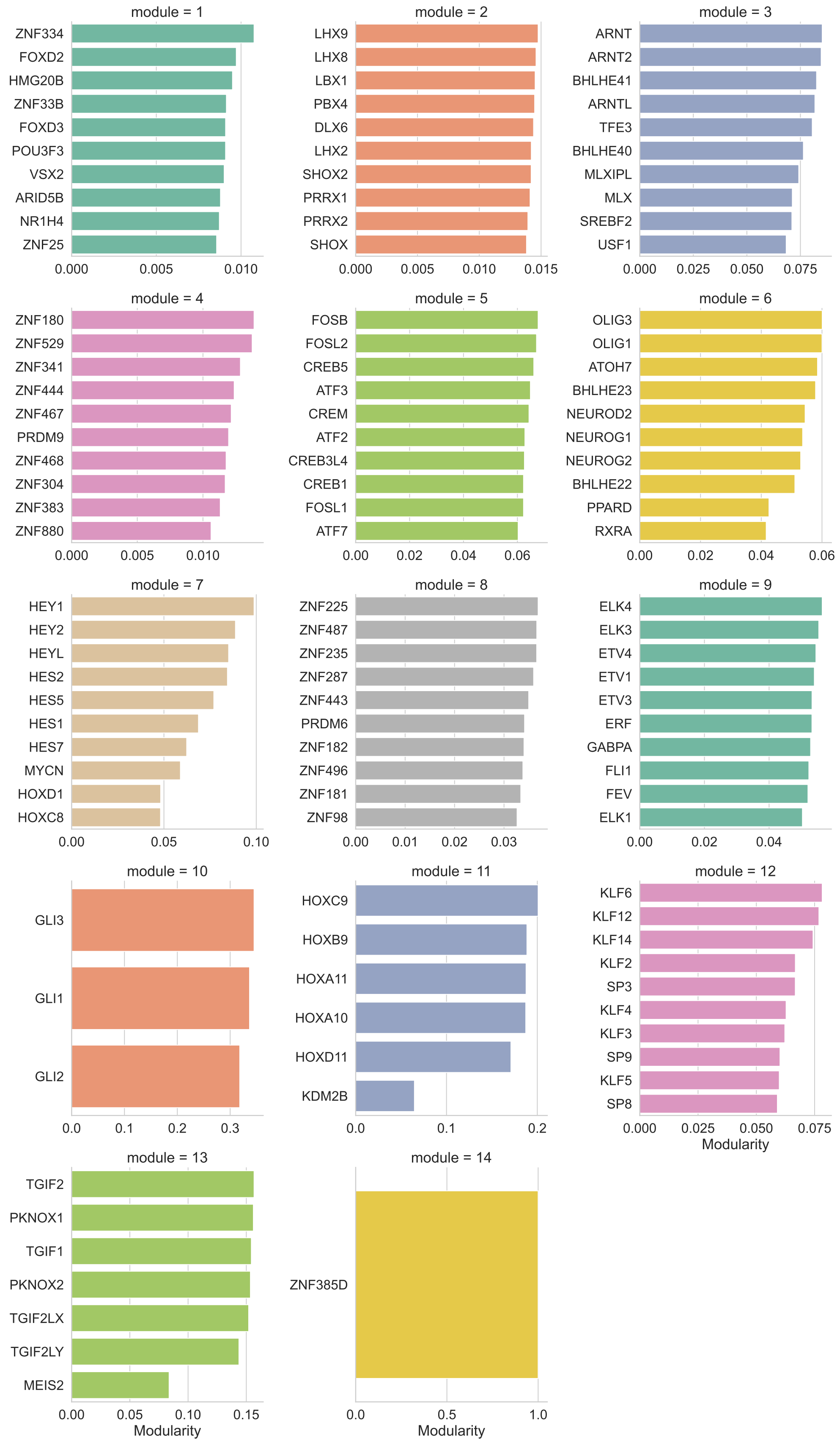

Figure S11. ALPACA's top TFs. For each module, we selected the 10 TFs with the highest modularity, that is those that contribute the most to the differential modularity between CMS2 and CMS4.

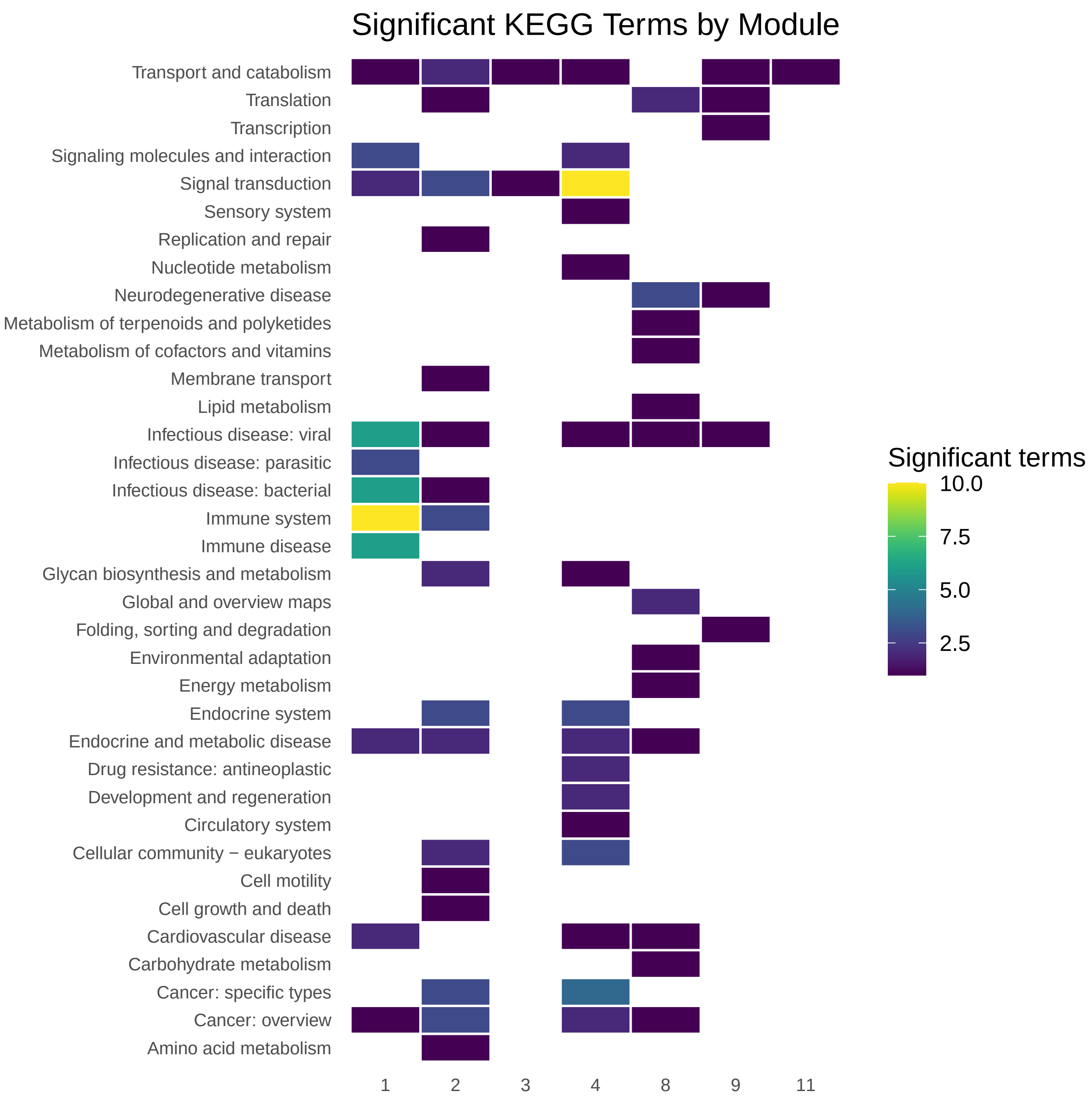

Figure S12. Pathway analysis of ALPACA's modules. For each module, we run the pathway analysis between the nodes in each module and the KEGG pathways, using R's clusterProfiler package. We then group the results based on the KEGG subcategory they belong to and count the significant terms.

Supplementary Tables

REACTOME enrichment for targets of TFs in cluster D

Term	Overlap	P-value	Adjusted P-value	Odds Ratio
Creation of C4 and C2 activators	31/81	1.1188890835418E-09	6.90354564545292E-07	4.6464702446038900
Cell surface interactions at the vascular wall	51/182	3.0709751817686E-09	9.47395843575613E-07	2.928937872861060
Binding and Uptake of Ligands by Scavenger Receptors	34/102	1.16220368969732E-08	2.39026558847748E-06	3.7526856922617600
Initial triggering of complement	31/89	1.56987176647831E-08	2.42152719979279E-06	4.00919352961713
Leishmania phagocytosis	39/129	2.23130556805187E-08	2.75343107097601E-06	3.254975191441080
Complement cascade	35/110	2.75594984807199E-08	2.83403509376736E-06	3.5035349241328300
Fcgamma receptor (FCGR) dependent phagocytosis	41/156	6.8681651106458E-07	6.05379696181208E-05	2.6779980022871300
Signaling by the B Cell Receptor (BCR)	42/167	1.77910217466724E-06	0.00013721325522121100	2.5236253682339000
Anti-inflammatory response favouring Leishmania parasite infection	35/137	8.63735349188979E-06	0.0005921385671662220	2.576603530486490
rRNA processing in the mitochondrion	14/35	2.31537263450701E-05	0.0014285849154908300	4.995559391886160
Fc epsilon receptor (FCERI) signaling	40/194	0.0003690257884500380	0.020698991952152200	1.9484735437419800
Class A/1 (Rhodopsin-like receptors)	35/167	0.0006085153001697720	0.03128782835039580	1.9897413064168900
GPCR ligand binding	42/224	0.001961469140422810	0.09309434304929800	1.7296595421891000
Peptide ligand-binding receptors	23/105	0.002715058138266520	0.11965649080788900	2.110627272074610
Amine ligand-binding receptors	5/10	0.0036276202738507000	0.14921611393105900	7.38555246847617
FOXO-mediated transcription	13/53	0.008199622126970790	0.31619792827131100	2.4652941596969000
Insulin-like Growth Factor-2 mRNA Binding Proteins (IGF2BPs/IMPs/VICKZs) bind RNA	4/8	0.009540610302219240	0.34626803273348600	7.382824182866910
Antimicrobial peptides	8/28	0.014087406060553000	0.4583737223150080	3.0635722599468700
FGFR4 ligand binding and activation	3/5	0.014115236181499400	0.4583737223150080	10.332735426009000
SARS-CoV Infections	68/442	0.01686712763842170	0.5203508876453100	1.358632067296990
Amyloid fiber formation	10/42	0.023488719520119900	0.6310051520689820	2.3874754930227200
Dissolution of Fibrin Clot	4/10	0.023522072119265100	0.6310051520689820	5.11059438318571
Kidney development	9/36	0.022815479951734500	0.6310051520689820	2.552488273875560
Regulation of necroptotic cell death	8/31	0.025896689199173200	0.6657607181620780	2.672013513575040
Biological oxidations	26/153	0.0400358591366688	0.8451238953644460	1.5380033574784200
Innate Immune System	131/945	0.03681566433944630	0.8451238953644460	1.2012559517099100
Myogenesis	6/22	0.039771494279398200	0.8451238953644460	2.9088511801789500
Loss of Function of TGFBR1 in Cancer	3/7	0.041091923599567900	0.8451238953644460	5.739744228533470
Adaptive Immune System	106/750	0.035921911295563	0.8451238953644460	1.2283152941168300
Defective RIPK1-mediated regulated necrosis	3/7	0.041091923599567900	0.8451238953644460	5.739744228533470

1

Table S1. REACTOME pathway enrichment for the targets of TFs in cluster D. For the targets of the TFs in cluster D, we run a pathway overrepresentation analysis with the REACTOME pathway database. Here we show the pathways with $p - value < 0.05$.

REACTOME enrichment for targets of TFs in cluster A

Term	Overlap	P-value	Adjusted P-value	Odds Ratio
Leishmania phagocytosis	26/129	4.21569919738418E-07	0.0002360791550535140	3.5828676075905400
Cell surface interactions at the vascular wall	30/182	4.83181100421583E-06	0.000825468123954678	2.799768607790200
Fcgamma receptor (FCGR) dependent phagocytosis	27/156	5.5958460183126E-06	0.000825468123954678	2.969623006175530
Creation of C4 and C2 activators	18/81	5.89620088539056E-06	0.000825468123954678	4.060816767411140
Complement cascade	21/110	1.28921880837977E-05	0.0012716123180039300	3.3512599252410800
Binding and Uptake of Ligands by Scavenger Receptors	20/102	1.36244176928992E-05	0.0012716123180039300	3.465189205487710
Initial triggering of complement	18/89	2.3373435996165E-05	0.0018698748796932000	3.604877735943840
Anti-inflammatory response favouring Leishmania parasite infection	22/137	0.0001270912393394170	0.008896386753759200	2.7158487353964500
Signaling by the B Cell Receptor (BCR)	25/167	0.00014565613639090400	0.009063048486545160	2.4968073275774900
Fc epsilon receptor (FCERI) signaling	25/194	0.0014133478427089400	0.07914747919170080	2.0959648329531300
Kidney development	8/36	0.00234245308328935	0.1192521569674580	4.133478367895470
FOXO-mediated transcription	10/53	0.002585571926244530	0.1206600232247450	3.3476825962256200
Aspirin ADME	5/19	0.007310164794079320	0.31489940651418600	5.249063890302300
Loss of function of MECP2 in Rett syndrome	4/13	0.009053510120533320	0.3621404048213330	6.551885384538850
Myogenesis	5/22	0.013989648003434500	0.45044475301132200	4.348510891303170
Innate Immune System	81/945	0.01567025708207290	0.45044475301132200	1.3198759025841800
Insulin-like Growth Factor-2 mRNA Binding Proteins (IGF2BPs/IMPs/VICKZs) bind RNA	3/8	0.013319620109513900	0.45044475301132200	8.79730054607274
Transcriptional regulation of granulopoiesis	6/31	0.016087312607547200	0.45044475301132200	3.5279937041079200
Transcriptional regulation by the AP-2 (TFAP2) family of transcription factors	6/30	0.013739054736949700	0.45044475301132200	3.6721943605158800
Glucuronidation	4/15	0.015512274229252800	0.45044475301132200	5.411835292183780
Dissolution of Fibrin Clot	3/10	0.02578302194706470	0.6562951041071020	6.450648532930360
Regulation of CDH11 function	3/10	0.02578302194706470	0.6562951041071020	6.450648532930360
Transcriptional regulation by RUNX3	12/96	0.02808705812843130	0.6838588066052840	2.050130429596150
Smooth Muscle Contraction	6/36	0.032000930629106800	0.7466883813458260	2.9488271332890500
Signaling by NOTCH1 HD+PEST Domain Mutants in Cancer	8/57	0.03678967677180870	0.7923930381620330	2.377146512527630
Signaling by NOTCH1 PEST Domain Mutants in Cancer	8/57	0.03678967677180870	0.7923930381620330	2.377146512527630
Postsynaptic nicotinic acetylcholine receptors	2/5	0.03976267467822500	0.8247073266594800	9.868144313217930
Surfactant metabolism	4/20	0.042127490094425900	0.8425498018885190	3.770854098642380
Signaling by NOTCH1	9/70	0.045349117204883900	0.8550701771095160	2.138624828373520
Signaling by NOTCH4	10/81	0.04580733091658120	0.8550701771095160	2.0335786507453800
GPCR ligand binding	22/224	0.04985446694235830	0.8554507505937300	1.5416294024956300
Glycosphingolipid metabolism	7/50	0.04965414778353900	0.8554507505937300	2.38576524545609

1

Table S2. REACTOME pathway enrichment for the targets of TFs in cluster A. For the targets of the TFs in cluster A, we run a pathway overrepresentation analysis with the REACTOME pathway database. Here we show the pathways with $p - value < 0.05$.

KEGG 2021 enrichment for targets of TFs in cluster D

Term	P-value	Adjusted P-value	Odds Ratio
TGF-beta signaling pathway	2.48574311940069E-05	0.0077058036701421300	3.019988310929280
Transcriptional misregulation in cancer	0.00011838600511097900	0.018349830792201800	2.2167008430166300
Cytokine-cytokine receptor interaction	0.00018606258744939800	0.018861201326119900	2.01001783033033
Antigen processing and presentation	0.00024337033969187000	0.018861201326119900	3.1492372152986700
Amoebiasis	0.003167301213570950	0.16063641285107300	2.2336917562724000
Asthma	0.003300331201939580	0.16063641285107300	4.703154782744
Viral protein interaction with cytokine and cytokine receptor	0.003627273838572620	0.16063641285107300	2.25914860748794
Gastric cancer	0.004165072298866050	0.16139655158106000	1.930047973264340
Pathways in cancer	0.005309881763878660	0.18289592742248700	1.4278343896029800
Hematopoietic cell lineage	0.00642522348971196	0.19918192818107100	2.169620497014060
Epstein-Barr virus infection	0.00857343648688083	0.22511457232477200	1.6532418398493700
Arachidonic acid metabolism	0.008714112477087950	0.22511457232477200	2.6239508869930700
Neuroactive ligand-receptor interaction	0.011684597675844700	0.2734075242836080	1.7469509466507200
Calcium signaling pathway	0.013035464353594000	0.2734075242836080	1.6528891842498800
Signaling pathways regulating pluripotency of stem cells	0.013803833745416300	0.2734075242836080	1.7899688597363000
Retinol metabolism	0.014111356092057200	0.2734075242836080	2.54912389563573
Staphylococcus aureus infection	0.01520219063915510	0.27721641753753300	2.185045435045440
IL-17 signaling pathway	0.01630080366536720	0.2807360631257680	1.972852233676980
Inflammatory bowel disease	0.020345197764012200	0.33194796351809400	2.2588584083440500
Intestinal immune network for IgA production	0.02281522942217870	0.3536360560437700	2.4633832976445400
Primary immunodeficiency	0.02589642462614270	0.36567840481762300	2.5699821322215600
Linoleic acid metabolism	0.025951370664476400	0.36567840481762300	3.6923899102180400
Complement and coagulation cascades	0.029464306184411400	0.3971276050942400	1.9615125329411000
Type I diabetes mellitus	0.031024169351406000	0.40072885412232700	2.4627568493150700
Autoimmune thyroid disease	0.03533995207094060	0.4382154056796640	2.585387248609330
Graft-versus-host disease	0.042424200647072800	0.5032938481671670	2.4621309370988400
Prostate cancer	0.043835270646817800	0.5032938481671670	1.6761839278040400
Hepatocellular carcinoma	0.04938104058133400	0.5467186635790540	1.4917963805311000

1

Table S3. KEGG pathway enrichment for the targets of TFs in cluster D. For the targets of the TFs in cluster D, we run a pathway overrepresentation analysis with the KEGG pathway database. Here we show the pathways with $p - value < 0.05$.

KEGG 2021 enrichment for targets of TFs in cluster A

Term	P-value	Adjusted P-value	Odds Ratio
Pathways in cancer	0.000747938	0.14230677574040300	1.7114527709087400
Transcriptional misregulation in cancer	0.000978053	0.14230677574040300	2.302796633179470
Gastric cancer	0.003165216	0.250779096	2.272352647352650
Intestinal immune network for IgA production	0.009353948	0.250779096	3.3438080050194800
Hippo signaling pathway	0.005438134	0.250779096	2.0849770642201800
Hepatocellular carcinoma	0.005589136	0.250779096	2.031952209993530
Basal cell carcinoma	0.007511519	0.250779096	2.9016263177411700
Cytokine-cytokine receptor interaction	0.004160565	0.250779096	1.9401753529151800
PPAR signaling pathway	0.015161895289182000	0.250779096	2.545488354795220
Endometrial cancer	0.013600153655384300	0.250779096	2.5986617312072900
Bacterial invasion of epithelial cells	0.012675416382725400	0.250779096	2.475711029092490
Bile secretion	0.012160736019803900	0.250779096	2.6540978044879600
Hematopoietic cell lineage	0.011645194336257000	0.250779096	2.383638307984790
Colorectal cancer	0.011599440441574000	0.250779096	2.280352786639140
Epstein-Barr virus infection	0.010780171259324300	0.250779096	1.8245258620689700
TGF-beta signaling pathway	0.00965665	0.250779096	2.3448453276738100
Fatty acid biosynthesis	0.015512109	0.250779096	5.030944849401730
Linoleic acid metabolism	0.015512109	0.250779096	5.030944849401730
Metabolism of xenobiotics by cytochrome P450	0.018044423	0.2723088279033320	2.6387744779247100
Adherens junction	0.018715383361053700	0.2723088279033320	2.310157042
Porphyrin and chlorophyll metabolism	0.021584665678258800	0.28550626	3.0764309764309800
Steroid hormone biosynthesis	0.021584665678258800	0.28550626	3.0764309764309800
Mineral absorption	0.026930818315737400	0.3407333969512860	2.61967502
Thyroid cancer	0.028226189	0.34224254437779000	2.863949843260190
Asthma	0.029600377927220100	0.3445483990728420	3.952236870542470
Viral protein interaction with cytokine and cytokine receptor	0.033977179	0.3802830416208250	2.068003448
Drug metabolism	0.036708966396366000	0.395641082	2.037479886
Ether lipid metabolism	0.040496699562009600	0.4208764133051710	2.5950284090909100
Retinol metabolism	0.045230707223669600	0.45386675179613300	2.5162534435261700
Ascorbate and aldarate metabolism	0.049349391	0.47868908895841100	3.254249354810000

1

Table S4. KEGG pathway enrichment for the targets of TFs in cluster A. For the targets of the TFs in cluster A, we run a pathway overrepresentation analysis with the KEGG pathway database. Here we show the pathways with $p - value < 0.05$.

module	stat	p	Bonferroni FWER	mean IN-module degree	mean OUT-module degree
1	385813.00	0.00E+00	0.00E+00	-78.194178	43.655882
2	2143764.00	1.87E-32	5.81E-31	2.421301	10.373974
3	36981.00	5.90E-03	1.83E-01	-0.132168	13.994770
4	1433866.00	0.00E+00	0.00E+00	-63.115197	89.458204
5	98890.00	2.12E-04	6.58E-03	0.165063	12.623169
6	104579.00	2.28E-19	7.05E-18	0.347839	28.194577
7	32643.00	3.15E-05	9.78E-04	-0.414056	21.782637
8	2663962.00	8.13E-07	2.52E-05	-4.844230	2.151773
9	218707.00	7.61E-07	2.36E-05	-1.094298	14.393717
10	955.00	8.54E-03	2.65E-01	0.074718	39.860753
11	869.00	1.59E-09	4.93E-08	0.659262	53.029421
12	98998.00	3.37E-01	1.00E+00	-0.456192	9.702970
13	9620.00	6.20E-02	1.00E+00	-0.154742	14.593134
14	62.00	1.96E-01	1.00E+00	0.024142	-20.562472

Table S5. Comparison of edge differences (CMS2-CMS4) for each gene's IN-module vs OUT-module degree. For each module, we apply a pairwise Wilcoxon signed-rank test of which we report the statistic and p-value, and we compute the Bonferroni FWER. We also report the average IN-module and OUT-module degrees.

module	stat	p	Bonferroni FWER	mean IN-module degree	mean OUT-module degree
1	40.00	1.02E-42	1.43E-41	-1121.821654	1331.685448
2	7088.00	1.77E-12	2.47E-11	33.390045	133.276001
3	11.00	8.39E-04	1.17E-02	-3.249790	-110.794156
4	788.00	9.05E-42	1.27E-40	-1139.382961	1268.302363
5	207.00	8.31E-01	1.00E+00	3.910279	-2.354879
6	2.00	1.40E-09	1.96E-08	8.815545	115.128064
7	83.00	4.30E-01	1.00E+00	-8.550258	13.984968
8	254.00	1.91E-06	2.67E-05	-282.032701	170.765688
9	136.00	1.15E-04	1.62E-03	-28.205519	-121.859152
10	3.00	1.00E+00	1.00E+00	1.892866	-2.441624
11	0.00	3.12E-02	4.38E-01	11.427210	139.448435
12	28.00	6.94E-04	9.71E-03	-13.333253	-158.706423
13	0.00	1.56E-02	2.19E-01	-4.686480	206.724260
14	0.00	1.00E+00	1.00E+00	0.458702	-102.928285

Table S6. Comparison of edge differences (CMS2-CMS4) for each TF's IN-module vs OUT-module degree. For each module, we apply a pairwise Wilcoxon signed-rank test of which we report the statistic and p-value, and we compute the Bonferroni FWER. We also report the average IN-module and OUT-module degrees.

Click here to access/download
Supplementary Material
gigascience_review_reply_1.pdf

Cover Letter to Editor
Click here to access/download;Personal Cover;tcga_pipeline_cover_letter_1.pdf

HARVARD T.H. CHAN SCHOOL OF PUBLIC HEALTH

John Quackenbush
Henry Pickering Walcott Professor
of Computational Biology
and Bioinformatics

Chair, Department of Biostatistics

May 6th, 2025

Dear Dr. Lan,

Attached please find a revised version of our paper, "Reproducible processing of TCGA regulatory networks," that we are resubmitting to *GigaScience* as a Technical Note.

In this manuscript we describe, tcga-data-nf, a robust, highly configurable Nextflow workflow that allows users to reproducibly infer gene regulatory networks for the thousands of samples in The Cancer Genome Atlas (TCGA) and that can easily be adapted to working with other large datasets.

Multiomic and gene regulatory network (GRN) modeling has been shown to provide insight into the drivers of health and disease phenotypes. Our research group has long been a leader in developing methods for inferring and analyzing GRNs; our collected tools, "The Network Zoo" (NetZoo), including DRAGON, PANDA, LIONESS, and ALPACA, have been cited more than 700 times. Given our interest in understanding common features among various cancer types, we undertook a project to infer GRNs in each sample and each cancer type represented in TCGA. Because consistency in the inference of so many networks was essential if we were to compare them, we recognized we needed to establish a uniform, validated workflow in which software tools were chained in a logical and justifiable order and that could be easily used by individuals with limited experience in GRN inference and analysis. In this manuscript we detail the structure and implementation details of the pipeline and then demonstrate how it can be used to identify differences between colon cancer subtypes.

Following the reviewers' requests we have now implemented the following substantial changes to the pipeline and the manuscript:

- We added DRAGON for joint analysis of CNV-expression data and ALPACA for comparison of PANDA networks. We also included these tools in our analysis of the regulatory changes that distinguish colon cancer subtypes.
- We documented how to add network inference methods (GENIE3 and WGCNA) that were developed by other groups, increasing the overall impact of our work.
- We improved and extended the "cross-validation" evidence of quality and usefulness of the networks.
- We now provide details on the computational performance of the pipeline.
- We addressed all minor issues raised by the reviewers, both in the code and the manuscript.

We believe that we have addressed all reviewer's concerns and correctly edited the manuscript to meet the journal's standards. A more detailed accounting of the changes we have made can be found in the response letter attached to this submission. As you requested, we have now registered the tools on WorkflowHub, bio.tools, and scicrunch.

As always, if you have any questions or if we can provide any additional information to assist you in your decision, please let us know.

On behalf of my co-authors and colleagues, I offer you my best,

John Quackenbush, Ph.D.
Henry Pickering Walcott Professor of Computational Biology and Bioinformatics
Chair, Department of Biostatistics

Department of Biostatistics
655 Huntington Ave
Building II, Room 419
Boston, MA 02115

TEL 617 432 9028
FAX 617 432 5619
johnq@hsph.harvard.edu

https://www.hsph.harvard.edu/john-quackenbush/
