# Supplementary material for: Reproducible processing of TCGA regulatory networks
Source: Gigascience. 2025 Oct 20;14:giaf126. doi: 10.1093/gigascience/giaf126 (PMC12720619; doi:10.1093/gigascience/giaf126)
Supplement: giaf126_GIGA-D-24-00535_Revision_2 [file giaf126_giga-d-24-00535_revision_2.pdf]

GigaScience

Reproducible processing of TCGA regulatory networks

--Manuscript Draft--

**Manuscript Number:**	GIGA-D-24-00535R2
**Full Title:**	Reproducible processing of TCGA regulatory networks
**Article Type:**	Technical Note
**Funding Information:**	NATIONAL CANCER INSTITUTE, NIH (R35CA220523) — Prof John Quackenbush<br>NATIONAL CANCER INSTITUTE, NIH (U24CA231846) — Prof John Quackenbush<br>NATIONAL CANCER INSTITUTE, NIH (P50CA127003) — Prof John Quackenbush<br>National Human Genome Research Institute (R01HG011393) — Prof John Quackenbush<br>NHLBI, NIH (T32HL007427) — Dr Katherine H. Shutta<br>NHLBI, NIH (P01HL114501) — Dr Katherine H. Shutta<br>NHLBI, NIH (K01HL166376) — Dr Camila M. Lopes-Ramos<br>American Lung Association (LCD-821824) — Dr Camila M. Lopes-Ramos

Funder	Recipient
NATIONAL CANCER INSTITUTE, NIH (R35CA220523)	Prof John Quackenbush
NATIONAL CANCER INSTITUTE, NIH (U24CA231846)	Prof John Quackenbush
NATIONAL CANCER INSTITUTE, NIH (P50CA127003)	Prof John Quackenbush
National Human Genome Research Institute (R01HG011393)	Prof John Quackenbush
NHLBI, NIH (T32HL007427)	Dr Katherine H. Shutta
NHLBI, NIH (P01HL114501)	Dr Katherine H. Shutta
NHLBI, NIH (K01HL166376)	Dr Camila M. Lopes-Ramos
American Lung Association (LCD-821824)	Dr Camila M. Lopes-Ramos

Abstract:

Background. Technological advances in sequencing and computation have allowed deep exploration of the molecular basis of diseases. Biological networks have proven to be a useful framework for interrogating omics data and modeling regulatory gene and protein interactions. Large collaborative projects, such as The Cancer Genome Atlas (TCGA), have provided a rich resource for building and validating new computational methods, resulting in a plethora of open-source software for downloading, pre-processing, and analyzing those data. However, for an end-to-end analysis of regulatory networks, a coherent and reusable workflow is essential to integrate all relevant packages into a robust pipeline.

Findings. We developed tcga-data-nf, a Nextflow workflow that allows users to reproducibly infer regulatory networks fromthe thousands of samples in TCGA using a single command. The workflow can be divided into three main steps: multi-omic data, such as RNA-seq and methylation, are (i) downloaded, (ii) pre-processed, and (iii) analyzed to infer regulatory network models with the Network Zoo. The workflow is powered by the NetworkDataCompanion R package, a standalone collection of functions for managing, mapping, and filtering TCGA data. Here, we demonstrate how the pipeline can be used to study the differences between colon cancer subtypes that are attributable to epigenetic mechanisms. Lastly, we provide a database of pre-generated networks for the 10 most common cancer types that can be readily accessed by the public.

Conclusions. tcga-data-nf is a complete, yet flexible and extensible, framework that enables the reproducible inference and analysis of cancer regulatory networks, bridging a gap in the current universe of software tools for analyzing TCGA data.

Corresponding Author: John Quackenbush
Harvard University HSPH: Harvard University T H Chan School of Public Health
Boston, MA UNITED STATES

Corresponding Author Secondary Information:

Powered by Editorial Manager® and ProduXion Manager® from Aries Systems Corporation

**Corresponding Author's Institution:**	Harvard University HSPH: Harvard University T H Chan School of Public Health
**Corresponding Author's Secondary Institution:**	
**First Author:**	Viola Fanfani
**First Author Secondary Information:**	
**Order of Authors:**	Viola Fanfani
	Katherine H. Shutta
	Panagiotis Mandros
	Jonas Fischer
	Enakshi Saha
	Soel Micheletti
	Chen Chen
	Marouen Ben Guebila
	Camila M. Lopes-Ramos
	John Quackenbush
**Order of Authors Secondary Information:**	
**Response to Reviewers:**	GigaScience review N2<br><br>Reviewer #2:<br>The authors have developed a well-structured and reproducible workflow for regulatory network inference from TCGA data, integrating established tools and offering valuable infrastructure for the community. The additions of ALPACA, support for more omics types, and improved parameter flexibility are welcome enhancements. However, further work is needed to strengthen the impact of the pipeline, particularly through clearer demonstration of the biological significance of findings (e.g., through gene sets enrichment analyses). This point if of particular importance, given the lack of benchmark with other tools, and will help better position this resource for broader adoption and scientific utility.<br><br>We truly thank the reviewer for providing such a careful and detailed comment to our manuscript. We have addressed and corrected all issues they pointed out and below you will find our detailed response.<br><br>Majors comments<br><br>* Figure 2D: The legend was not changed, it still reads: "Association graph for the TFs in cluster D for CMS2 (left) and CMS4 (right)." This should be fixed. The accompanying text for this panel is "All these differences between CMS2 and CMS4 can also be visualized at once as the subgraph that emerges from cluster D, Figure 2D.", which is clearly not very insightful. I would suggest either describing key insights from this panel, or moving it to the supplements. In the first case, we can now clearly see that some edges are much stronger; like TP53 to POU2F2. It may be interesting to describe in particular such examples, if relevant.<br><br>We thank the reviewer for pointing this out, and we apologize for the error; we have fixed the caption accordingly. We also agree that we should have provided more details on what we learned from the results presented in the panel, particularly as these will provide readers not only insight into colorectal cancer but also familiarity with the value of DRAGON networks.<br><br>“While we have described the individual role of these TFs, it is important to remember that these differences between CMS2 and CMS4 can also be contextualized as the subgraph that emerges from cluster D (Figure 2D). For instance, there is a clearly strong difference between the association of methylation of POU2F2 and expression of TP53 (edge values CMS2: –0.024, CMS4: 0.021). Positive correlation between TP53

Powered by Editorial Manager® and ProduXion Manager® from Aries Systems Corporation

expression and POU2F2 methylation is consistent with evidence that silencing of TP53 and availability of POU2F2 are leading to oncogene-induced senescence escape and tumor progression in colorectal cancer [97]. If we instead focus on interactions between more than two nodes, we can see how PAX1, OTX1, HOXC5, and PKNOX1 are differentially associated between the two subtypes. These are all TFs involved in the “Wnt/β-catenin signaling” pathway, which is well known for its role in cancer development and progression [98] and was also enriched for the epigenetically controlled TFs in our analysis of DRAGON edges.”

* Figure S5: This figure is not very informative as one doesn't know if the presence of certain genes from your network in various databases is higher than expected or just as expected by chance. As such, the figure is of little value, and it is hard to conclude much. I would recommend conducting gene set enrichment analysis to see which gene set (CMS4, CMS2, cluster A, ect) is enriched in which database, and showing the results in an additional supplementary figure.

We thank the reviewer raising this point and we agree that we should have presented our results more clearly. Figure S5 is a detailed version of the tail of Figure 2A and we show the actual TFs for which we have evidence of “silencing”. In the previous version we had only reported the names of some of these genes, and we realized that without a graphic element it was hard to follow.

Also, following the reviewer’s suggestion we have now tested the actual distributions of the (Mi-Ei) edges for each subtype and the disgenet/cosmic annotations and we show that some of them are known for their role in cancer and we annotate them in this figure to help the reader follow the text and results. We have also carried out pathway ORA and included the results in the Supplementary Figure S5.

We have revised the text as follows:

“To evaluate the relevance of these edges, we investigated the relationship between cancer phenotype and TFs for which we inferred direct epigenetic regulation based on the DRAGON networks. We first considered the actual edge weights (Mi, Ei) of the cancer drivers (according to the COSMIC cancer gene census). We used a Kolmogorov-Smirnov test to check if the cancer-associated TFs have lower edge weights than is the case for TFs that are not annotated as being cancer-associated. We found that colon drivers have lower edge weights in CMS2 (p-value: 0.039) and all drivers have lower edge weights in CMS4 (p-value: 0.088). Then, we selected the TFs for which the (Mi, Ei) edge weights are in the first decile of the distribution of at least one subtype and we found 108 TFs with evidence ofmethylation-driven “silencing” (Supplementary Figure S5). Some of these TFs (STAT5A, CREB3L1, ZNF24, HMGA1, IRF8, PAX8, CDX2, CREB3L2, TFEB, MGA, NFIB, KLF6, LEF1, HOXD13, HOXA13, HOXB13, GATA2) are known to be cancer drivers [83, 84, 85, 86, 87]. For example, STAT5A, the only TF that has low (Mi, Ei) edges in all subtypes, is a known oncogene involved in the JAK signaling cascade [88, 89], while CREB3L1, LEF1, and PAX8 are all known to be involved in invasion and metastasis [90, 91, 92, 93].

We also assessed the over-representation of the TFs under putative epigenetic regulation in the Reactome pathway database using a hypergeometric test. While the pathway analysis on TFs is not sufficiently powered because most pathways contain fewer than 10 TFs, we found nominal enrichment not only for broadly defined transcriptional pathways, such as “Generic Transcription Pathway” (OR: 1.71, p-value:0.0093) and “RNA Polymerase II Transcription” (OR:1.64, p-value 0.014), but also formore specific pathways such as “Formation of paraxialmesoderm” (OR: 31.96, p-value: 0.006), “Gastrulation” (OR: 2.72, p-value: 0.032), “Incretin synthesis, secretion, and inactivation” (OR: 22.63, p-value: 0.033) and “Beta catenin independent WNT signaling” (OR: 6.37, p-value: 0.045) (Supplementary Figure S5). These are all pathways related to the epithelial-mesenchymal transition (EMT), which is an established hallmark of cancer that drives invasion and metastasis [94, 95]. Lastly, to further validate these networks, we compared DRAGON edges with the co-expression edges in the StringDB database and found evidence of correlation with DRAGON expression-expression edges (Supplementary Figure S6).”

Also in the sentence: "edges in cluster A of the DRAGON methylation-expression

networks are enriched for TFs that are involved in Colorectal Neoplasms according to the DisGeNet" -> I could not find this information easily/clearly in the Figure. Also, if this is a relevant result it should be mentioned in the main text, not only in the letter to the editor. Enrichment of cancer gene sets in some of your subtypes/clusters could be quite convincing/informative.

We apologize for not including this in the main text. Upon further investigation, we realized that most cancer drivers are annotated to Cluster E and so have included this result in the main text:

"It is finally worth mentioning that the cancer driver TFs are only over-represented in cluster E, with 19 TFs (CAMTA1, HLF, IRF4, STAT5A, HOXB13, ETV4, GATA2, STAT5B, HOXC13, FOXA1, ATF1, MGA, PRRX1, IKZF3, ERF, HOXA13, DOT1L, MNX1, TP53) annotated in the OncoKB dataset (Fisher's exact test OR: 1.84, p-value: 0.005), and 15 annotated in the Cancer Gene Census dataset (Fisher's exact test OR: 1.84, p-value: 0.013). In thismodule, we do not see differentially associated edges between CMS2 and CMS4, which suggests a shared contribution of methylation to cancer etiology."

In terms of style: I would put x-axis labels in a 90 degree angle.

Thank you for the suggestion. We have made this change.

* "and colon cancer drivers HIF1A and CUX1, both of which are implicated in tumorigenesis and progression [84]"; I could not find CUX1 in the list. Also going up to 50 genes seems a bit extreme; I would not describe any gene more than the top 10 or 20. If one wants to include more genes I would do pathway enrichment analysis to show more systematically what this list of genes contains. Because out of 50 genes, one can most likely pick a few that support any given hypothesis.

We thank the reviewer for the suggestions and we have implemented them in the manuscript. We first show the top 20 TFs and briefly describe the involvement of some of them in key processes. We also tested whether TFs annotated to Cancer driver lists (Disgenet and colon cancer drivers from the Cancer Gene Census) had stronger evidence of being different between CMS2 and CMS4. For that we applied both a Fisher's Exact test and a Kolmogorov Smirnov test on the statistics, and we have evidence that they show stronger differences between subtypes than expected by chance. We also include these TFs in the figure.

We have revised the text as follows:

"Among the top TFs with varying edges between CMS2 and CMS4 based on the Wilcoxon test results were ZNF554, for which we have partial evidence of involvement in cancer progression through the WNT/β-Catenin Signaling Pathway [99], KLF16, which plays a role in the stress-related programming of colorectal cancer [100], and DOT1L, a methyltransferase that regulates core stem cell genes and affects tumorigenesis and drug resistance [101]. These TFs are significantly associated with colon cancer drivers. When comparing The Wilcoxon test statistics of the cancer TFs to the rest of the TFs, we get a significant Kolmogorov-Smirnov test (stat: 0.21, p-value: 0.013). Also, there are 27 significant (FDR < 0.01) TFs that are annotated by either the Cancer Gene Census or the DisGeNET dataset as colon cancer drivers (Fisher's exact test OR : 1.6, p-value: 0.057, Supplementary Figure S9D). Among the cancer drivers that show the biggest association changes between CMS2 and CMS4,we found HIF1A and CUX1, both of which are implicated in tumorigenesis and progression [87], and GATA4, which controls senescence [102, 103]."

* "Modules 1 and 2 are enriched for Immune System and Cell Cycle pathways" -> I could not find enrichment for "Cell Cycle pathways" on the figure. Also, and more importantly, I think displaying the number of enriched KEGG subcategories is peculiar and uncommon. This type of analysis can hide for instance a very significant term. I think the authors should provide, in addition, a table with the top significant results to ensure the immune terms are indeed the most significant results. Alternatively, I would directly show the most significant terms on the y-axis of Figure S12.

We thank the reviewer for this comment. We had initially thought that a summary plot would be easier to read, but we agree that an uncommon plot is sometimes harder to read. For this reason, we have now changed the figure to a complete plot of all significant pathways (Supplementary figure S11). We again thank the reviewer because this helped us drawing more meaningful conclusions than presented in the initial manuscript:

“ALPACA uniquely assigns TFs and genes in the network to modules and also provides a ranking of nodes based on their contribution to modularity. We can thus use ALPACA results to investigate the functional role of eachmodule. For example, we find that Module 1 is enriched for many immune-related pathways, including "Immune System", “Immune Disease”, and “Infectious Disease”, cytokine-related signaling pathways, “TNF signaling pathway”, and “NF-κB” (Figure 2E). Of the TFs that have the highest contribution to modularity for Module 1, the majority have been validated as key regulators of colorectal cancer (Figure 2F). Indeed, 6 out of 10 leading TFs have direct evidence, and some experimental validation, of their involvement in colon cancer risk and progression: ZNF334 [104], FOXD2 [105], FOXD3 [106], POU3F3 [107], NR1H4 [108], and VSX2 [109].

We performed a similar analysis on all the other modules. Module 2 appears to be more cancer-specific as it is consistently enriched for cell cycle pathways (“Cell cycle”, “Cellular senescence”), known cancer-related signaling pathways (“mTOR” and “JAK-STAT” signaling pathways),metabolic pathways, and cancer specific disease terms (Supplementary Figure S11). Module 4 is strongly enriched for signaling pathways, including “Wnt signaling”, and development and regeneration pathways (Supplementary Figure S11). While we know that zinc finger proteins (ZNF) are still not well characterized, hindering the functional annotation of module 4where 9 out of 10 top TF are ZNFs, we found that ZNF341, which is the fourth highest-ranking hit formodularity contribution (Supplementary Figure S12), is a regulator of STAT3 and plays a key role in hyper-IgE syndrome (HIES), a condition that often manifests as severe and chronic bacterial infections [110]. In parallel, we found reports of associations between IgE-mediated immune reactions and colorectal cancer risk [111]. Although we do not have definitive evidence or validation of the role of ZNF341 in the colon cancer subtypes, we believe that this suggestive result demonstrates how multi-omic network analysis can find complex regulatory patterns. Taken together, the analysis of Modules 1,2, and 4 shows that CMS2 and CMS4 at the regulatory level exhibit differences in the immunological response, cellular processes, such as growth and senescence, and signaling and developmental pathways.”

* Are there any ALPACA modules that are similar to clusters A and D? This angle might help streamline the results section. It would also be interesting to have a glimpse of how results from these two analysis type overlap/relates. Also, since Alpaca is a great addition to the pipeline, I would suggest adding one or 2 panels from the Alpaca analysis to Figure 2. For instance, it could be module 1 from Figure S11 and top significant results from module 1 in Figure S12.

We thank the reviewer for the great suggestion! We have indeed changed the panel as suggested, adding the top pathways and TFs for module 1 to figure 2. We have also fixed the caption accordingly. We also looked into the overlap between DRAGON and ALPACA results finding an interesting trend of differential silencing between CMS2 and CMS4 for HOX TFs, that we now explain in the end of the results section and in Figure 2G.

“Finally, we examined the relationship between the results of the multi-omic DRAGON analysis (methylation-expression) and ALPACA. It is worth noting that DRAGON, as used here, estimates partial correlations between the methylation and expression of TFs, while ALPACA infers differential modularity between GRNs. Methylation is only one of many possible modifications that can change gene expression, translation, and regulation (mutations, CNVs, chromatin accessibility, etc.). Therefore, we do not necessarily expect DRAGON and ALPACA (used with PANDA-based GRNs) to be concordant. However, using the DRAGON results, we explored whether promoter methylation can explain the differential modularity of these colorectal cancer GRNs.

To interpret these results, we first note that DRAGON uses a different, more stringent filtering method than PANDA. Consequently, only about 50% of TFs in the ALPACAmodules can be found in the DRAGON networks (Figure 2G). Nevertheless, we investigated the representation of these overlapping TFs in the ALPACA modules that might indicate that epigenetic changes drive the regulatory differences in a specificModule. Specifically, we looked for TFs in ALPACA modules for which we have evidence of methylation-driven “silencing” in either CMS2 or CMS4, that are those with a strong negative association between expression and methylation of the same TF in DRAGON (Figure 2G, Supplementary Figure S13). While most “silenced” TFs are distributed across the ALPACA modules, in Module 11 we found both the TFs, HOXA10 and HOXB9, “silenced” in CMS2 and not CMS4. This result suggests that these TFs may play an important role in the overall differential regulation of gene expression in the two subtypes. It is also consistent with reports that HOX TFs are differentially accessible in colorectal cancer and that they are involved in cancer development and progression [96]."

##Minor comments##
* The authors write "We have gone through the paper and edited it to address missing words and incorrect word choice."; however this is questionable as there are still many instances of missing pieces. See: "we have implemented WGCNA [?] and GENIE3 [?], that are third-party network inference methods" , "However, netZooPy supports GPU-based com-putation of PANDA-LIONESS network [?].", "method section", "metabdata", ". specifically BATF". Also, the legend in Figure 2d was not updated; "D) Association graph for the TFs in cluster D for CMS2 (left) and CMS4 (right).". I would encourage a more careful read for the next submission.

We apologize and thank the reviewer for the careful re-read. Multiple coauthors have reviewed the manuscript and we are hopeful that the number of typos has been significantly reduced in number.

* "This effect is also visible also in the adjacency matrices representing the CNV-expression DRAGON networks, where a block structure in the CNV-CNV edges is evident (Supplementary Figure S9A)." -> unclear if "CNV-expression" was the right expression here; isn't Figure S9A showing only CNV-CNV edges? If the wording was correct, I would clarify the legend to explain better.

We thank the reviewer for pointing this out, and we have now rephrased to make it clearer.

We have revised the text as follows: “This effect was also visible in the adjacency matrices, where there is an evident block structure in the CNV-CNV edges (Supplementary Figure S9A). Moreover, we have evidence that, even in this case, DRAGON networks are able to capture direct effects of CNV onto expression; the CNV-expression edges on the same TFs (Ci, Ei) tend to be skewed towards positive values (Supplementary Figure S9B), which is expected behavior for somatic variation.”

* It is unclear to me how the modules displayed in Figure S12 were selected. For instance, module 6 is quite significant and was not displayed. Please clarify this selection in the legend.

We thank the reviewer as it gives us an opportunity to prepare a more easy-to-follow figure and write a clearer legend (this now appears as Figure S11)

* Figure S8: Add a light red bar or similar at p-value = 0.05 so that one can easily see the significance cutoff.

Thank you for the suggestion, we have now implemented it.

* In the computational performance part, if I understood correctly the new features are not benchmarked, including ALPACA. I would recommend updating the benchmark to include all features in the latest pipeline.

Powered by Editorial Manager® and ProduXion Manager® from Aries Systems Corporation

Thank you for pointing this out. We have now updated the test performance report and have included the correct link. We have also released v0.0.18 of the workflow.

* Figure S12 appears before Figure S11 in the text.

Thank you for noticing, we have now swapped them.

* Figure 2C: use 90 degree angles to show the x-axis labels (BATF, E2F2, …). Currently, it is difficult to map them to a given tick. I would also consider swapping this panel with Figure S8 as to my view it is more informative to highlight significant results, especially given the stated importance of immune infiltration in CSM4-like tumors.

We thank the reviewer for the suggestion; we have changed the orientation of the labels. The main reason for not including pathway analysis results on TFs is how hard it is to do any pathway analysis on such a small set of terms, as most pathways are annotated to less than 10 TFs. Given how small the dataset of interest is, we do believe that annotating these TFs to their respective pathways is useful to the reader to gain better insight.

* "Using functional enrichment analysis, we found that TFs in clusters A and D are involved in the metabolism of proteins and DNA repair and that cluster D, in contrast to cluster A, includes genes preferentially involved in the Immune System (Supplementary Figure S8). Specifically BATF, GATA3, NFATC3, NFATC2 and TP53 are involved in the Immune System, while E2F, TERF2, and TP53 participate in cellular responses to stimuli (Figure 2C)." -> this part should be re-written slightly to indicate the enrichment in the term "cellular responses to stimuli" and clarify that the genes listed are the one driving the enrichment.

We thank the reviewer for the suggestion and we have updated the text to:

“Using functional enrichment analysis, we found that TFs in clusters A and D are involved in the metabolism of proteins and DNA repair. TFs in cluster D, in contrast to cluster A, include genes preferentially involved in the Immune System (Supplementary Figure S8). These include BATF, GATA3, NFATC3, NFATC2, and TP53, which we found to be involved in the “Immune System” pathways (including “Signaling by the B Cell Receptor (BCR)” (nominal p-value : 0.023, OR: 13.8) and “Innate Immune System” (nominal p-value: 0.03, OR: 4.71), while E2F, TERF2, and TP53 participate in "Cellular responses to stimuli" pathways, including "DNA Damage/Telomere Stress Induced Senescence", (nominal p-value : 0.014, OR: 19.3), and "Cellular Senescence" (nominal p-value : 0.024, OR: 6.54) (Figure 2C).

We note that a general limitation of this functional enrichment analysis is that most TFs are only annotated to a small subset of pathways related to transcription and not comprehensively mapped to those on which they have a regulatory effect.”

* "We ran all tests with the v0.0.15 version of tcga-data-nf with standard configuration files and no parallelization of processes. The test full workflow requires 1h 11min of wall time (4.7 CPU hours) and a peak memory of 13.6 Gb for runTCGAPanda". -> What do you mean "no parallelization of processes"? I could not find such parameter in the documentation. Also, Nextflow automatically parallelizes jobs. And the wall time vs CPU hours indicates a degree of parallelization. Note, that this is also mentioned on the documentation but the clickable links yield "404 - page not found".

We thank the reviewer for the comment, because this sentence was indeed unclear. Nextflow parallelizes jobs based on the “executor” and “process” configurations. For the tests, we use a “process” definition that was using all resources available. This way nextflow runs one process after the other (yet the CPUs are parallelizing tasks). The

reason to do this here is to show the maximum wall time used to run the tests, which is what the end user should expect when running this on a local machine, or before deciding how many resources to use on a HPC. We have changed the sentence accordingly and we have updated the documentation link.

“We report the performance on an AWS EC2 instance c5.4xlarge with 32Gb of memory and 16 vCPUs. We ran all tests with the v0.0.18 version of \nf with standard configuration files that allow nextflow to run all jobs serially, with no job parallelization, where each process is allowed to use all computing resources available. For reference, this would be consistent with testing the workflow on a local machine.

* "Within the top 20 TFs that show high correlation between CNV and expression, we observe known colon cancer drivers [83] ZNF703 and SMAD4, and IRF2 and GATA6 whose DNA aberrations are likely oncogenic [78]."
Where is this data shown? Also, what is the difference with the next text part: "Finally, we identify which TFs have the most different CNV-expression edges between the CMS2 and CMS4 subtypes."

We thank the reviewer for pointing this out. The figure is the same one referenced in the sentence before, but we have now added the reference again to make it clearer to the reader. Also, the beginning of the next paragraph was confusing, so we have now rewritten it.

“...Moreover, we have evidence that, even in this case, DRAGON networks are able to capture direct effects of CNV onto expression; the CNV-expression edges on the same TFs (Ci, Ei) tend to be skewed towards positive values (Supplementary Figure S9B), which is expected behavior for somatic variation. Within the top 20 TFs that showed high correlation between their own CNV and expression, we observed known colon cancer drivers [86] ZNF703 andSMAD4, and IRF2 and GATA6, whose DNA aberrations are likely oncogenic [81] (Supplementary Figure S9B). Finally, we explored the strongest differences between CMS2 and CMS4 subtypes in terms of partial correlations between CNV and expression. For each CNV node Ci, we compared the edge set {(Ci, Ej)} between the two subtypes. We conducted a paired Wilcoxon signed-rank test for each TF i, testing the null hypothesis that there is no difference in CNV-expression edge weights between CMS2 and CMS4 (Supplementary Figure S9C). Among the top TFs with varying edges between CMS2 and CMS4 based on the Wilcoxon test results were ZNF554, for which we have partial evidence of involvement in cancer progression through the WNT/β-Catenin Signaling Pathway [99], KLF16, which plays a role in the stress-related programming of colorectal cancer [100], and DOT1L, amethyltransferase that regulates core stem cell genes and affects tumorigenesis and drug resistance [101]. These TFs are significantly associated with colon cancer drivers. When comparing the Wilcoxon test statistics of the cancer TFs to the rest of the TFs, we get a significant Kolmogorov-Smirnov test (stat: 0.21, p-value: 0.013). Also, there are 27 significant (FDR < 0.01) TFs that are annotated by either the Cancer Gene Census or the DisGeNET dataset as colon cancer drivers (Fisher’s exact test OR : 1.6, p-value: 0.057, Supplementary Figure S9D). Among the cancer drivers that show the biggest association changes between CMS2 and CMS4, we found HIF1A and CUX1, both of which are implicated in tumorigenesis and progression [87], and GATA4, which controls senescence [102, 103].”

* "Interestingly, module 4 is instead uniquely involved into pathways that control the Extracellular Matrix Organization." I could also not find this result in the figure. Also, why is this relevant? (include reference if needed).

We thank the reviewer for spotting this and we apologize for the mistake. This is a result we found in a previous analysis but isn’t shown in any of the figures. We have now removed it. Also, that paragraph has now been updated and extended.

	* "Lastly, while many zinc finger proteins are still not well characterized, ZNF341 is a regulator of STAT3" -> Please clarify that most TFs with the highest modularity in module 4 were encoding for zing finger proteins. Also, clarify that ZNF341 was the fourth hit in this module.<br><br>We thank the reviewer for pointing this out and we have now rewritten the text to include their comments.<br><br>##Recommendations##<br>* Currently, the ending is a bit negative. I would add one or 2 sentences at the very end of the discussion to summarize the main advantages of your pipeline and how it could be used or expanded in future work.<br><br>* "Modules 1, 2, and 4, are those the exhibit the biggest differences in connectivity" -> " the most significant difference in connectivity"<br><br>* Consistent usage of the past tense in the results section would likely be better (i.e., "We investigated which pathways are enriched", "we extend our search beyond")<br><br>* The expression "third-party network" gives a business tone; I would recommend replacing it with "externally developed network inference methods", or "community-developed network inference methods"<br><br>We truly thank the reviewer for this detailed review! We have also implemented these recommendations since we agree that they improve the overall clarity and quality of the paper.
Additional Information:	
Question	Response
Are you submitting this manuscript to a special series or article collection?	No
**Experimental design and statistics**<br><br>Full details of the experimental design and statistical methods used should be given in the Methods section, as detailed in our Minimum Standards Reporting Checklist. Information essential to interpreting the data presented should be made available in the figure legends.<br><br>Have you included all the information requested in your manuscript?	Yes
**Resources**<br><br>A description of all resources used, including antibodies, cell lines, animals and software tools, with enough	Yes

information to allow them to be uniquely identified, should be included in the Methods section. Authors are strongly encouraged to cite Research Resource Identifiers (RRIDs) for antibodies, model organisms and tools, where possible.<br><br>Have you included the information requested as detailed in our Minimum Standards Reporting Checklist?	
**Availability of data and materials**<br><br>All datasets and code on which the conclusions of the paper rely must be either included in your submission or deposited in publicly available repositories (where available and ethically appropriate), referencing such data using a unique identifier in the references and in the “Availability of Data and Materials” section of your manuscript.<br><br>Have you have met the above requirement as detailed in our Minimum Standards Reporting Checklist?	Yes
GigaScience has policies and guidelines in place for the use of generative AI-writing tools such as ChatGPT. If you have used such writing tools to assist with writing the manuscript this must be declared and cited in the text. Authors should not list AI-writing tools and other AI-assisted technologies as an author or co-author and should acknowledge that they are fully responsible for text generated or refined by AI-writing tools.<p><br><br>A summary of use (particularly in the introduction or among methods) needs to be included at the end of the paper, and the outputs should also be included as a supplementary file hosted in GigaDB or other open repositories. Please read our guidelines for more information. <p><br><br>By submitting to GigaScience, you are aware of the journal's AI-writing tools	No

Powered by Editorial Manager® and ProduXion Manager® from Aries Systems Corporation

policy, and if you have declared use of such tools below, you have acknowledged this where appropriate in your manuscript and have made a summary of use and outputs available. <p> <b>AI-assisted writing tools have been used in the preparation of this manuscript?	

Manuscript
Click here to access/download;Manuscript;GIGA-D-24-00535_manuscript.pdf

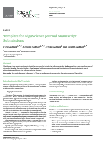

GigaScience, 2023, 1–15

doi: xx.xxxx/xxxx
Manuscript in Preparation
Paper

PAPER

Reproducible processing of TCGA regulatory networks

Viola Fanfani[1], Katherine H. Shutta[1,2], Panagiotis Mandros[1], Jonas Fischer[1], Enakshi Saha[1], Soel Micheletti[1], Chen Chen[1], Marouen Ben Guebila[1], Camila M. Lopes-Ramos[1,2,3] and John Quackenbush[1,2]

[1]Department of Biostatistics, Harvard T.H. Chan School of Public Health, Boston, MA, USA and [2]Channing Division of Network Medicine, Brigham and Women's Hospital, Boston, MA, USA and [3]Department of Medicine, Harvard Medical School, Boston, MA, USA

[*]Corresponding Author, johnq@hsph.harvard.edu

Abstract

Background. Technological advances in sequencing and computation have allowed deep exploration of the molecular basis of diseases. Biological networks have proven to be a valuable framework for analyzing omics data and modeling regulatory interactions between genes and proteins. Large collaborative projects, such as The Cancer Genome Atlas (TCGA), have provided a rich resource for building and validating new computational methods, resulting in a plethora of open-source software for downloading, pre-processing, and analyzing those data. However, for an end-to-end analysis of regulatory networks, a coherent and reusable workflow is essential to integrate all relevant packages into a robust pipeline.
Findings. We developed tcga-data-nf, a Nextflow workflow that allows users to reproducibly infer regulatory networks from the thousands of samples in TCGA using a single command. The workflow can be divided into three main steps: multi-omic data, such as RNA-seq and methylation, are (i) downloaded, (ii) pre-processed, and (iii) analyzed to infer regulatory network models with the Network Zoo. The workflow is powered by the NetworkDataCompanion R package, a standalone collection of functions for managing, mapping, and filtering TCGA data. Here, we demonstrate how the pipeline can be used to investigate the differences between colon cancer subtypes attributed to epigenetic mechanisms. Lastly, we provide a database of pre-generated networks for the 10 most common cancer types that can be readily accessed by the public.
Conclusions. tcga-data-nf is a complete, yet flexible and extensible, framework that enables the reproducible inference and analysis of cancer regulatory networks, bridging a gap in the current universe of software tools for analyzing TCGA data.

Key words: "Gene Regulatory Network"; "The Cancer Genome Atlas"; "Cancer"; "Nextflow"; "NetworkDataCompanion"; "reproducibility" (3 to 10 keywords)

Background

There is a growing recognition of the importance of ensuring that scientific research is reproducible and that analytical steps are transparent [1, 2]. The field of bioinformatics has been particularly receptive to this trend, with many prominent scientists and journals advocating for the use of open-source software, open data, and reproducible methods [3, 4]. Projects such as Bioconductor [5] and Bioconda [6] facilitate the sharing and reuse of bioinformatics software. The Galaxy project [7] has pioneered the development of platforms for training and sharing best practices for complete data analysis workflows. Workflow management tools such as Nextflow [8], Snakemake [9], and WDL [10] facilitate reproducibility of complex data analysis pipelines.

These methodological advances are closely tied to the growing availability of large-scale biological data. The falling cost of sequencing has enabled the generation of population-level omics data, allowing thousands of subjects to be profiled in a single project to investigate complex traits and diseases. As notable examples, the UKBiobank [11] has collected multi-omic and clinical data for more

Compiled on: September 27, 2025.
Draft manuscript prepared by the author.

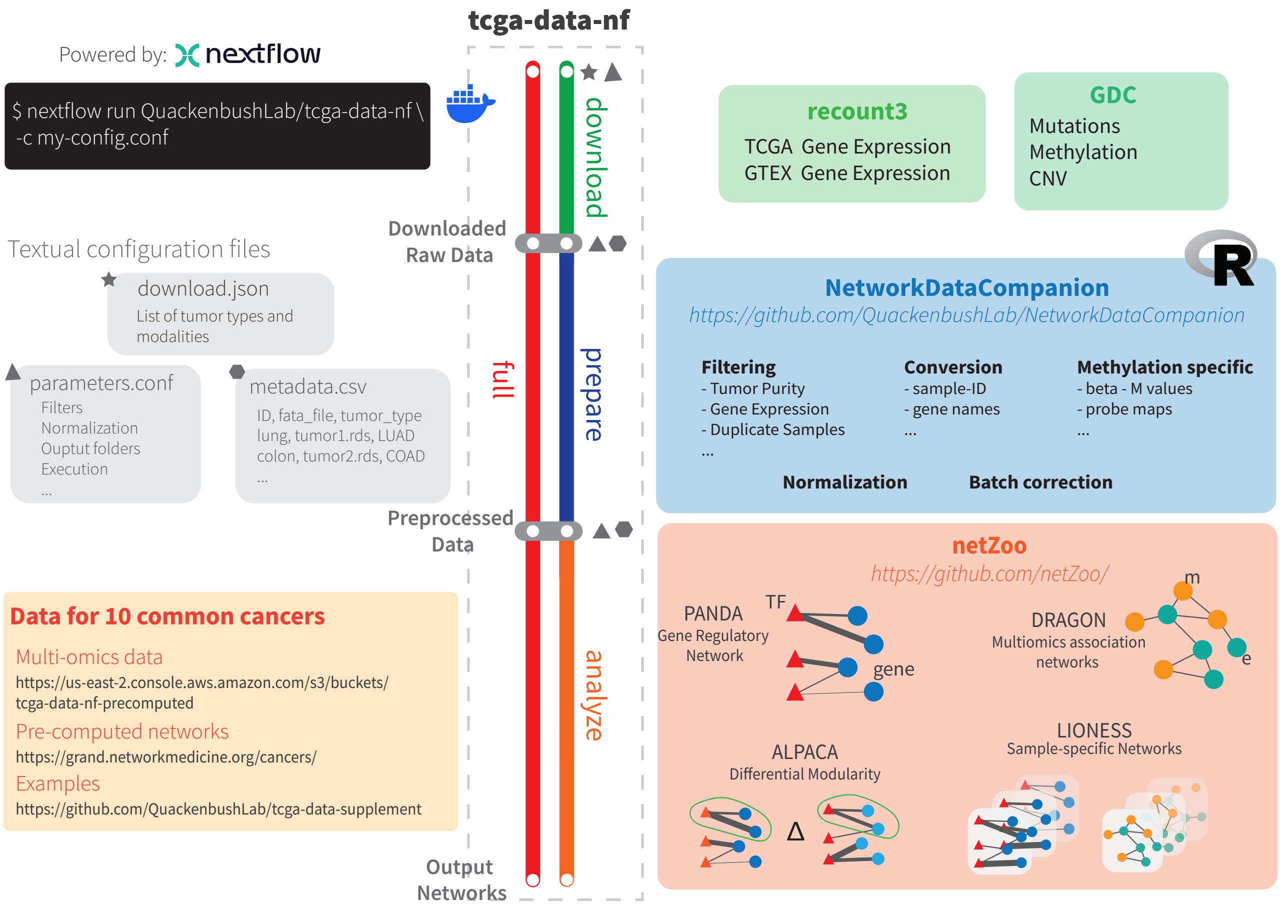

Figure 1. Graphical Abstract

than 500,000 individuals representative of the UK population, and the 1000 Genomes Project [12] includes samples from more than 4,000 individuals collected to characterize human genetic variation. Ongoing data collection efforts (such as the 100,000 genome project [13]) demonstrate that this deluge of data is not slowing down. With the consequent massive scope for downstream analyses comes the need for reproducible and rigorously managed software pipelines to work with these data.

The Cancer Genome Atlas (TCGA) [14] was one of the first large-scale collaborative projects designed to study the molecular basis of disease, including samples collected from more than 10,000 cancer patients representing over 30 tumor types. TCGA data have been invaluable for the study of regulation in both healthy and tumor tissues[15, 16, 17, 18, 19] and for developing and benchmarking analytical methods for omics data [20, 21]. The TCGA dataset has grown in value with data from related projects, such as The Cancer Protein Atlas (TCPA) [22, 23] and the Clinical Proteomic Tumor Atlas Consortium (CPTAC) [24, 25], which create new opportunities for the development of innovative methods and applications.

Given that most pathologies result from the complex interplay among multiple genomic, transcriptomic, epigenomic, and other factors [26, 27], the inference and analysis of network models integrating multiple omics are important analytical approaches that have been enabled by large-scale omic data resources. Biological networks model the high-level organization of biological systems by representing interactions between biological entities, capturing the molecular mechanisms that define biological states and the progression between them. Most notably, network analyses have helped to elucidate the etiology and progression of tumors and provide insight into important clinical features[28, 29, 30, 31, 32]. Many network types have been used to explore biological processes, including protein-protein interaction networks, networks of DNA-protein interactions, and co-expression networks. Of particular utility are bipartite gene regulatory networks (GRNs), which consist of transcription factors (TFs) and the genes they regulate in a specific phenotype [33]. Comparison of GRNs across individual phenotypes can lead to the discovery of regulatory "rewiring" to create or eliminate disease-related functions and pathways [33, 34, 35, 36, 37, 38]. Multi-omic association networks can capture additional interactions that influence regulation by combining multi-modal data. For example, combining epigenetic and transcriptomic data identified putative regulatory associations between gene expression patterns and DNA methylation in breast cancer samples [39].

For studies of cancer and the regulatory changes that drive the disease, the TCGA dataset is unique in the breadth and depth of multi-omic data available from the same samples, allowing a wide range of complementary network analyses to be performed. However, accessing and analyzing the wealth of data from TCGA or other large-cohort studies, from raw data to networks, can be a non-trivial task. For example, raw sequencing data cannot be publicly released and must be aligned and quantified prior to downstream analyses, integration of multi-omic data requires matching of samples across assays, and each data type has its own set of data pre-processing and filtering steps.

Several existing tools have been designed to address these challenges. The Genomic Data Commons (GDC) provides an application programming interface (API) [40, 41] that interfaces with R via the TCGAbiolinks R package [42, 43]. TCGAbiolinks provides both programmable access to the TCGA data and tools for data wrangling tasks such as identifier mapping and data filtering based on clinical features. For example, with a few lines of code, one could download

all RNA-seq and mutation data for a specific tumor (or subtype) from subjects over 70 years of age. The TCGAbiolinks package also provides a number of functions for *ad hoc* pre-processing of the data, in addition to the most commonly used analytical steps, such as differential gene expression analysis [44, 43].

Once the data are downloaded and pre-processed, additional analyses can be performed to extract biologically meaningful insights. The Network Zoo project [45] is a growing open-source suite of tools for the inference and analysis of biological networks [46]. The 16 methods currently in netZoo include PANDA [33], which uses gene expression data together with prior TF-binding information and TF-TF interaction data to infer Gene Regulatory Networks (GRNs) describing TF-gene interactions, ALPACA [47], which compares the structure of two PANDA networks, DRAGON [39], which creates robust multi-omic Gaussian graphical models (partial correlation networks), and LIONESS [48], which estimates individual networks for each sample in a population by using a leave-one-out strategy with linear interpolation.

It is then clear that a full analytical pipeline for TCGA or similar data involves a large number of data access, pre-processing, and analysis steps. Although reusable and documented pieces of software exist to handle each specific step, as detailed above, chaining these processes back-to-back from data download to final analysis yields a complex workflow with many interdependent processing decisions. Moreover, tools are required to facilitate the transfer of data and results from one step to the next. As with any programmatic workflow, each step introduces additional possibilities for issues with accuracy and reproducibility. Given the number of steps involved in network analysis of TCGA data, in particular, we propose that a single, robust, and transparent workflow is an essential tool for conducting accurate, reproducible analyses that yield meaningful results.

To this end, we developed *tcga-data-nf*, a Nextflow workflow that generates network models of TCGA data with a single command, managing all steps from data download through pre-processing to network generation. *tcga-data-nf* is structured around three key workflow modules: Download , Prepare , and Analyze . First, in the Download module, *tcga-data-nf* facilitates the download of TCGA clinical and phenotypic data as well as several omics modalities, including RNA-seq, mutation, methylation, and copy number variation (CNV) data. The Prepare module involves pre-processing steps specific to each data type. Finally, the Analyze module generates individual sample GRNs and expression-methylation/CNV association networks by combining LIONESS with PANDA and DRAGON, respectively. In a detailed example, we show that *tcga-data-nf* not only allows us to swiftly generate networks for the four consensus subtypes of colon cancer [49], but we also demonstrate the use of *tcga-data-nf* to expand our understanding of the regulatory processes driving subtype-specific prognosis. In particular, we find molecular evidence for epigenetic involvement in the more aggressive CMS4 subtype.

As part of developing *tcga-data-nf*, we created the *NetworkDataCompanion* (*NDC*). *NDC* streamlines routine steps in TCGA data processing, including filtering and mapping gene and sample identifiers between modalities (which is often a challenge with such heterogeneous data) and modality-specific data transformation, such as normalization and cleaning. While *NDC* was designed to provide necessary back-end functions for *tcga-data-nf*, it also serves as a standalone tool for separate use.

To provide users with a seamless out-of-the-box experience, *tcga-data-nf* comes equipped with all essential supplementary components, including a Docker container, conda environments, comprehensive documentation, and introductory tutorials to help users get started. As an additional illustrative example and resource for the public, we have applied *tcga-data-nf* to generate GRNs for the ten most frequent cancer types (BRCA, LUAD, LUSC, KIRC, LIHC, PAAD, PRAD, SKCM, STAD, COAD) and published these networks on the Gene Regulatory Network Database (GRAND) [50] (version 1.7) for easy access and exploration.

Findings

The open-source *tcga-data-nf* is a Nextflow [8] pipeline that allows users to fully execute network analysis on TCGA data in a single, end-to-end pipeline spanning data download, preparation, and network inference. By chaining and combining atomic tasks, called processes, this pipeline enables users to infer GRNs and other networks with a single command. Specific tumor sources (such as BRCA or COAD) and workflow parameters are specified by the user in configuration files called by the workflow.

The whole workflow consists of three main steps: Download , Prepare , and Analyze , which involve downloading the raw data, preparing the data for the analysis, and inferring the networks (Figure 1). **Download** Data downloaded include RNA-seq, mutation, methylation, and copy number variation data, which are the modalities used to generate regulatory networks and to characterize genetic and epigenetic aberrations that could explain dysregulation in cancer. Clinical and phenotypic data are also downloaded to support downstream investigations. **Prepare** Gene expression and methylation data are cleaned and pre-processed. Sample duplicates, outliers, and lowly expressed genes are removed. CpG-level methylation is mapped to overall gene promoter methylation values, and sample identifiers are matched to facilitate multi-omic integration. **Analyze** The *tcga-data-nf* pipeline provides an interface for estimating GRNs with PANDA [33] and multi-omic partial correlation networks with DRAGON [39]. For both methods, *tcga-data-nf* also facilitates the generation of sample-specific networks with LIONESS [48], and ALPACA [47] is used to identify differential modules between multiple PANDA networks.

Although designed as a full pipeline that runs all the steps above, the modular design of *tcga-data-nf* allows users to run the Download , Prepare , and Analyze steps independently(Figure 1). The decoupling of these three different steps is useful from a practical standpoint; the Download step does not require efficient computational resources, but it is time-consuming and is performed once to generate a long-term data repository. The Prepare step involves several choices of data preparation parameters, and a user may wish to test several configurations. The Analyze step is likely to be run multiple times in the course of an investigation, to observe how various parameters affect the results or fit new types of networks.

The Prepare step of *tcga-data-nf* is powered by the associated *NetworkDataCompanion* (*NDC*) R package, which provides a wide range of functions for working with different data modalities in TCGA. While there are many existing packages to work with TCGA data, it is often necessary to resort to multiple tools to carry out simple functions. The *NDC* package addresses this issue by integrating a set of tools into a single package, streamlining the routine steps of TCGA data processing. TCGA-specific functions enable users to filter and map gene and sample identifiers across modalities, addressing one of the often vexing challenges with heterogeneous data. Omic-specific functions allow users to normalize, transform, and filter data according to the specific needs of downstream tasks. Although *NDC* is explicitly designed to support *tcga-data-nf*, the package is standalone and can be reused in other applications.

State-of-the-art environment and containerization tools are also integrated with *tcga-data-nf*, which can interface with Docker [51], Singularity [52], and conda [53]. We provide containers and configuration details for both Docker and Singularity, as well as a customizable configuration structure that allows users to define conda environments.

In developing *tcga-data-nf* and *NetworkDataCompanion*, we generated PANDA and PANDA-LIONESS networks for the ten most common tumors in TCGA. We have published these networks in GRAND, our group's cloud-based network database, so they can be used by the broader cancer research community without the

need to run the more expensive steps of the workflow. Finally, we present a complete analysis of the four consensus molecular subtypes of colon cancer (TCGA-COAD) that integrates the PANDA and DRAGON COAD networks to uncover key epigenetic and regulatory differences between them. Below, details of the workflow and COAD application are presented, including descriptions of the *tcga-data-nf* pipeline steps and *NetworkDataCompanion* functionalities.

Download

For network generation and analysis, we focus on five TCGA data modalities: gene expression, methylation, copy number variation (CNV), and mutation data, alongside patient clinical data. All TCGA data are downloaded from the Genomic Data Commons (GDC) project [54]. To download methylation, CNV, mutation, and clinical data, *tcga-data-nf* uses the "TCGAbiolinks" [43] and "GenomicDataCommons" [40] R packages. For the gene expression data, *tcga-data-nf* uses the recount3 R package [55], taking advantage of recount3's normalization tools and standardized processing across studies. We also leverage recount3 to download GTEx gene expression data, enabling future analyses that compare cancer with normal tissue [56].

The Download step is driven by a JSON configuration file (Listing 1) that is modality-centric, which means that for each data type (gene expression, mutations, CNVs, ...) one can specify multiple cancer types to be downloaded. It also provides users with the ability to pass a list of samples of interest, which is helpful in selecting data from a specific subpopulation or discarding problematic samples.

The Download step generates simple, comma-separated tables of metadata that store key configuration parameters used to download each dataset and the path of the resulting files. These metadata tables directly interface with the following Prepare step. A schematic of the Download step is shown in Supplementary Figure S1. For the Download step, we have written a dedicated testing profile ("testDownload") described in the "Testing" section that allows the workflow to be piloted and validated.

Prepare

The raw downloaded data needs to be pre-processed before being used in downstream analyses. Pre-processing requires a range of parameter choices for steps such as normalization and filtering, leading to a large number of possible parameter configurations. Since these choices can affect the results and conclusions from any analysis[57, 58], *tcga-data-nf* provides a config file for the user to define such parameters for the pre-processing steps implemented in the Prepare step.

As implemented in *tcga-data-nf*, the Prepare step primarily deals with pre-processing gene expression and methylation data, which are the data types we used to generate GRN and multi-omic association networks. However, users may also have specific pre-processing steps that are unique to their analysis. Consequently, *tcga-data-nf* is naturally extensible such that users can easily implement their own functions to integrate with the existing Prepare step. Below, we describe the key steps used to pre-process gene expression and methylation data in the current pipeline.

Expression From recount3, we obtain gene-level raw count data for RNA-seq data from both TCGA and GTEx. To clean these data, we implement the following steps:

- Normalization: Raw count data are normalized, with options to generate either TPM [59, 60] or CPM (CPM with TMM normalized library size [61, 62]).
- Duplicates: Where duplicate samples are present (two or more samples from the same research subject), the sample with the greatest sequencing depth is retained, and others are discarded.
- Batch correction: If specified, batch effects are removed using ComBat [63, 64]. We also visualize the effect of batch removal using PCA.
- Low expression: We remove genes that have low expression, defined as those genes with less than n counts in at least p% of samples for user-defined values of n and p.
- Sample purity: For TCGA tumor samples, we remove those that have low purity, using previously computed purity values [65] and allowing the user to specify their method of choice.
- Tissue type: TCGA contains both tumor and adjacent normal tissue samples, and one can save the data for these tissue types separately to facilitate subsequent analyses comparing tumor to adjacent normal tissue.

All these choices are set by appropriate parameters in the configuration file, and users can specify multiple values for each parameter, allowing *tcga-data-nf* to output results for all parameter combinations.

Methylation The TCGA methylation array data undergo two key pre-processing steps: mapping of individual CpG probes to genes to generate gene-level promoter methylation values and general data cleaning/transformation. By default, CpGs are mapped to genes using the publicly available annotation for the Illumina 450k array, which is mapped to hg38 using GENCODE v36 [66][67]; however, users can supply their own annotation file if desired. Once CpGs are mapped to genes, the average promoter methylation for each gene is calculated. The promoter region is defined as the area 200 base pairs upstream of the transcription start site (users can redefine this boundary), and the average of methylation *beta*-values for any probes falling within this region is calculated.

It is not always necessary to perform this mapping to promoter methylation for all the genes represented on the EPIC array; in some cases, only a particular subset may be relevant for a specific analysis. For example, in the application of DRAGON below, we map the probes only to genes encoding transcription factors (TFs). The default behavior is to map to all genes on the array.

After obtaining gene-level methylation for each sample, the following pre-processing options are available:

- Duplicates: If a sample has multiple methylation array profiles, the user can handle these in several ways, including selecting the duplicate with the least missingness, choosing a duplicate at random, or excluding samples with duplicates altogether.
- Missing data: Any gene that has missing promoter methylation values for more than m% of the samples is removed from the analysis, with $m = 20\%$ by default. If a gene has missing values for $\leq m\%$ of the samples, these missing values are estimated by mean imputation. If a user does not wish to impute any data, they may set $m = 0$ to exclude all missing data.
- Conversion from *beta* to M-values: The mean promoter methylation *beta* value $\beta \in [0, 1]$ is converted into an M-value $M \in (-\infty, \infty)$ using the formula:

$$M = \log_2 \left\{ \frac{\beta}{1 - \beta} \right\} \quad (1)$$

  See [68] for a detailed discussion of the relative merits of using β-values vs. M-values in methylation analyses.
- Transformation to approximate normality: We provide the option to apply a nonparanormal transformation [69] to the M-values to achieve approximate normality in the distribution for input into DRAGON, which requires approximately normal data. The nonparanormal transformation is powered by the `huge.npn` function of the R package `huge` [70].

A simple schematic of the Prepare step is shown in Supplementary Figure S2. At the end of these steps, we obtain a comma-separated file with probes in rows and samples in columns. As in the Download step, a metadata table storing the parameters used for the Prepare step is also produced. The Prepare step relies heavily on the companion R package *NetworkDataCompanion* (*NDC*), which is described in depth in the *NetworkDataCompanion* section.

Analyze

Following the downloading and preparation of TCGA data, the Analyze step implements the necessary code to generate network models. The Analyze step generates two types of networks: GRNs, generated with PANDA, and multi-omic association networks, generated with DRAGON. PANDA [33] is a GRN inference method that uses bulk expression data, together with a prior TF-gene binding network (based on TF motif mapping) and a TF-TF protein interaction network, and generates population-level bipartite TF-gene regulatory networks. DRAGON [39] is a network inference method based on Gaussian graphical models that infers partial correlations on multi-omic data. We also used LIONESS [48], which estimates sample-specific networks by interpolation between a network for the entire population and that for the population less the sample for which we are estimating a network. In our pipeline, we used LIONESS to estimate both sample-specific PANDA and sample-specific DRAGON networks for each sample. Finally, to compare the connectivity of PANDA GRNs, we use a netZoo method called ALPACA [47], which identifies the gene modules that best distinguish two networks by maximizing their differential modularity. To show how the workflow can be easily extended to run methods that do not belong to the netZoo suite. For example, we implemented WGCNA [71] and GENIE3 [72], both methods developed by others and not included in netZoo. A simple scheme of the Prepare step is shown in Supplementary Figure S2.

The *analyze* workflow relies on implementations of PANDA, DRAGON, and LIONESS in the netZooPy package and ALPACA from the netZooR package [46]. The processes for network inference use the command line interfaces and Python objects for PANDA, DRAGON, and LIONESS; these require users to specify the various input omic files and the parameters for each method. ALPACA uses the output from PANDA networks to identify condition-specific modules and only requires the unique identifiers of the inferred GRNs. Conveniently, the Prepare step in *tcga-data-nf* generates a metadata table that the users can directly use to specify which files are then used by the Analyze step. Given the storage requirements of the data the pipeline can generate, we have updated netZooPy to save networks in Hierarchical Data Format (HDF), which reduces the size and reading/writing time for storing the networks.

Lastly, we note that although the pipeline described here primarily uses gene expression and methylation data to generate network models, the workflow can be easily edited to include other data types and methods. As an example, we included an analysis that integrates CNV data with gene expression to estimate DRAGON networks. To demonstrate extensibility, we also include two externally developed network inference methods, WGCNA and GENIE3. To facilitate engagement among the broader biomedical research community, *tcga-data-nf* documentation includes clear explanations of how to extend the pipeline.

Full

The full pipeline combines the Download , Prepare , and Analyze steps described above. It is designed to be run with a single command and represents the most comprehensive network analysis pipeline described in this manuscript. Specifically, the full pipeline integrates PANDA, ALPACA, DRAGON, and LIONESS. It generates PANDA GRNs, which are compared with ALPACA. It also generates multi-omic DRAGON methylation-expression and CNV-expression networks using DRAGON, and incorporates LIONESS to generate both PANDA-LIONESS and DRAGON-LIONESS sample-specific networks.

While we recommend separating the three steps by, for example, downloading the data once and then running the Prepare and Analyze steps as needs change, there are instances where researchers may wish to use the full pipeline to run all three steps for a single project at once. We illustrate this approach in the sections "Multiomics associations identify changes between colon subtypes" and "Regulatory differences between colon cancer subtypes GRNs", where we demonstrate how the full pipeline can be used to generate DRAGON and PANDA networks for the TCGA colon cancer consensus molecular subtypes, and to gain insight into the differences between their regulatory programs.

For the full pipeline, the configuration files and parameters mirror those of the three modular pipelines above. First, a "JSON" configuration file similar to that used in the Download step (Listing 2) needs to be populated with all the data modalities of interest. Then, the processing and analysis parameters need to be specified in the Nextflow configuration file as they were in the Prepare and Analyze steps.

NetworkDataCompanion

The *NetworkDataCompanion* (*NDC*) R package supports preprocessing of TCGA bulk RNA-seq and DNA methylation data. The purpose of having a version-controlled R package for these functions is to attain the high standard of reproducibility in the overall *tcga-data-nf* pipeline. While *NDC* is the engine behind the *tcga-data-nf* workflow processing steps, the software is standalone and can be installed and used outside the workflow. *NDC* currently provides three broad classes of functions: functions for mapping identifiers, functions for filtering data, and functions for preparing expression and methylation data (such as normalization and scaling) (Figure 1). While many of the functions described below are intuitively simple, it is worth noting that the complexity of the TCGA project and its wealth of data require commensurately complex data wrangling to handle tasks such as filtering by sample quality and matching sample identifiers between omics types. The goal of *NDC* is to provide a version-controlled and unit-tested environment for developing and maintaining tools for these tasks, with the end goal of enhancing accuracy and reproducibility.

Mapping functions Given the variety of data types used in the pipeline and the number of other resources with which they interface, there are many instances where we need to map between "synonymous" identifiers. We have implemented several wrapper functions that use existing tools such as GDC and TCGAutils to retrieve and convert various sample identifiers (TCGA barcodes, UUIDs). We have also implemented functions for translating gene names between Ensembl IDs [73], HUGO Gene Nomenclature [74], and Entrez IDs [75, 76] by leveraging GENCODE v26 [77], which was used for TCGA and the AnnotationDbi R package [78].

Sample filtering functions By default, *tcga-data-nf* downloads and processes all samples available for a particular TCGA dataset. *NDC* provides three different functions that allow a user to filter these samples. First, a user may eliminate duplicate samples using one of several methods: select the sample with the strongest signal based on RNA sequencing depth (for expression duplicates), select the sample with the least missing data (for methylation duplicates), select the sample with the highest tumor purity (for any sample type for which tumor purity is available), select a single sample from a set of duplicates at random, or exclude all samples with duplicates. Second, users can filter samples based on the TCGA sample type (for example, primary tumor tissue, metastatic tissue, or adjacent normal tissue). Finally, a user may filter samples based on tumor purity, excluding samples where the number of non-tumor cells is too large according to the sample's published annotation [65].

Data preparation functions With *NDC*, users can apply common data transformations to both gene expression and methylation data. For RNA-seq data from recount3, one can normalize read counts to transcripts per million (TPM) [59] or counts per million (CPM) [62]

and their corresponding log transformations, where a pseudocount is added to avoid undefined logs. For methylation data, functions are provided to convert methylation *beta*-values to *M*-values (logit base-2 transformed *beta*-values) and vice-versa [79] and to aggregate methylation values to get gene-level information, such as average methylation within a promoter region or gene body.

Collectively, the functions in *NetworkDataCompanion* represent a comprehensive set of tools for basic processing, filtering, and mapping that are needed to clean and prepare TCGA data. We note that although there are R packages that cover each of the individual tasks described above, *NetworkDataCompanion* aggregates them into a unified solution in one version-controlled and unit-tested package to facilitate their use together and to allow them to be seamlessly integrated into the *tcga-data-nf* pipeline. To engage the broader research community, *NetworkDataCompanion* is hosted on GitHub and contributions are encouraged.

Multi-omic partial correlation networks identify differences between colon subtypes

Colorectal cancer affects nearly two million individuals worldwide each year and is forecast to increase in incidence by 84% in the next 20 years [80]. Molecular profiling studies have identified four major expression-based consensus subtypes (CMS1: MSI immune; CMS2: canonical; CMS3: metabolic; CMS4: mesenchymal) [49] with distinct phenotypic and clinical features. CMS1 and CMS3, each of which has a distinctive genomic and epigenomic profile, together represent approximately 25% of cases. CMS2 and CMS4 are the most common subtypes, and although they have similar patterns of somatic mutation, structural variation, and methylation, they differ significantly in outcomes; CMS2 tumors have relatively good survival rates, while CMS4 cases are characterized by more aggressive tumors and poorer prognosis [81]. Additional studies have described intra-tumor heterogeneity and clinically actionable features that distinguish CMS4 and have found evidence that this more aggressive mesenchymal subtype often arises from CMS2-like tumors [82]. We then reasoned that multi-omic association networks and GRNs could provide further insight into the mechanisms that drive the difference between these subtypes.

Using the `full` pipeline, we specified the TCGA samples that belong to each subtype [49], downloaded and pre-processed the data, and generated multi-omic and GRN networks for each subtype. We began by constructing four subtype-specific DRAGON partial correlation networks that integrate TF gene expression with promoter methylation. Given F_M transcription factors for which we have methylation data and F_E for which we have expression data, each network has a total of $F_M + F_E$ nodes. The corresponding adjacency matrix is symmetric (as DRAGON networks are undirected) and can be divided into three blocks: (i) expression-expression partial correlations (E_i, E_j)), (ii) methylation-expression partial correlations (M_i, E_j), and (iii) methylation-methylation partial correlations (M_i, M_j).

Because promoter methylation is generally inhibitory, we expected it to exhibit an inverse partial correlation with gene expression [83, 84]. Indeed, by examining the distribution of methylation to expression edge weights (M_i, E_j), we saw that weights for edges connecting a gene to its promoter methylation (M_i, E_i) have a distribution more strongly skewed to negative values than the distribution of the promoter methylation to the other genes (M_i, E_j) (Figure 2A). We also noticed that there is often consistency in these (M_i, E_i) edges across different subtypes (Supplementary Figure S4).

To evaluate the relevance of these edges, we investigated the relationship between cancer phenotype and TFs for which we inferred direct epigenetic regulation based on the DRAGON networks. We first considered the actual edge weights (M_i, E_i) of the cancer drivers (according to the COSMIC cancer gene census). We used a Kolmogorov-Smirnov test to check if the cancer-associated TFs have lower edge weights than is the case for TFs that are not annotated as being cancer-associated. We found that colon drivers have lower edge weights in CMS2 (p-value = 0.039) and all drivers have lower edge weights in CMS4 (p-value = 0.088). Then, we selected the TFs for which the (M_i, E_i) edge weights are in the first decile of the distribution of at least one subtype, and we found 108 TFs with evidence of methylation-driven "silencing" (Supplementary Figure S5). Some of these TFs (STAT5A, CREB3L1, ZNF24, HMGA1, IRF8, PAX8, CDX2, CREB3L2, TFEB, MGA, NFIB, KLF6, LEF1, HOXD13, HOXA13, HOXB13, GATA2) are known to be cancer drivers [85, 86, 87, 88, 89]. For example, STAT5A, the only TF that has low (M_i, E_i) edges in all subtypes, is a known oncogene involved in the JAK signaling cascade [90, 91], while CREB3L1, LEF1, and PAX8 are all known to be involved in invasion and metastasis [92, 93, 94, 95].

We also assessed the over-representation of the TFs under putative epigenetic regulation in the Reactome pathway database using a hypergeometric test. While the pathway analysis on TFs is not sufficiently powered because most pathways contain fewer than 10 TFs, we found nominal enrichment not only for broadly defined transcriptional pathways, such as "Generic Transcription Pathway" (OR: 1.71, p-value = 0.0093) and "RNA Polymerase II Transcription" (OR:1.64,p-value = 0.014), but also for more specific pathways such as "Formation of paraxial mesoderm" (OR: 31.96, p-value: 0.006), "Gastrulation" (OR = 2.72, p-value = 0.032), "Incretin synthesis, secretion, and inactivation" (OR = 22.63, p-value = 0.033) and "Beta-catenin independent WNT signaling" (OR = 6.37, p-value = 0.045) (Supplementary Figure S5). These pathways are all related to the epithelial-mesenchymal transition (EMT), a well-established hallmark of cancer that drives invasion and metastasis [96, 97]. Lastly, to further validate these networks, we compared DRAGON edges with the co-expression edges in the StringDB database and found evidence of correlation with DRAGON expression-expression edges (Supplementary Figure S6).

We then explored the inter-omic partial correlation edges (M_i, E_j) that connect methylation associated with one TF to the expression of another TF and focused on the edges that differ between the subtypes, under the hypothesis that such edges can identify complex regulatory relationships that distinguish disease states. We selected the edges with the highest absolute value (either the strongest positive or negative partial correlation) and performed hierarchical clustering (using average linkage and Euclidean distance) on these edges. We identified five clusters (Figure 2B), with clusters A and D showing distinct patterns that represent opposite associations between CMS2 and CMS4. Even when compared to the rest of the edges connecting the same TFs (Supplementary Figure S7), edges in clusters A and D switch direction between these two subtypes, suggesting a specific change in the regulatory process.

Using functional enrichment analysis, we found that TFs in clusters A and D are involved in the metabolism of proteins and DNA repair. TFs in cluster D, in contrast to cluster A, include genes preferentially involved in the Immune System (Supplementary Figure S8). These include BATF, GATA3, NFATC3, NFATC2, and TP53, which we found to be involved in the "Immune System" pathways (including "Signaling by the B Cell Receptor (BCR)" (nominal p-value = 0.023, OR = 13.8) and "Innate Immune System" (nominal p-value = 0.03, OR = 4.71), while E2F, TERF2, and TP53 participate in "Cellular responses to stimuli" pathways, including "DNA Damage/Telomere Stress Induced Senescence", (nominal p-value = 0.014, OR = 19.3), and "Cellular Senescence" (nominal p-value = 0.024, OR = 6.54) (Figure 2C). We note that a general limitation of this functional enrichment analysis is that most TFs are only annotated to a small subset of pathways related to transcription and not comprehensively mapped to those on which they have a regulatory effect. This structural limitation of the pathway database results in smaller pathway overlap and reduced power to detect enrichment.

This over-representation of immune-related TFs is consistent with reports that CMS4-like tumors exhibit a greater degree of immune infiltration than do other subtypes [82]. Further, epigenetic changes in the FOX/HOX and SNAIL TF families are known to be in-

volved in colorectal cancer etiology [98], and TP53, FOXA1, GATA3, and GATA6 are all TFs that, when active in an aberrant form, are recognized as being hallmarks of cancer [88].

While we have described the individual role of these TFs, it is important to remember that these differences between CMS2 and CMS4 can also be contextualized as the subgraph that emerges from cluster D (Figure 2D). For instance, there is a clearly strong difference between the association of methylation of POU2F2 and expression of TP53 (edge values CMS2: −0.024, CMS4: 0.021). Positive correlation between TP53 expression and POU2F2 methylation is consistent with evidence that silencing of TP53 and availability of POU2F2 are leading to oncogene-induced senescence escape and tumor progression in colorectal cancer [99]. If we instead focus on interactions between more than two nodes, we can see how PAX1, OTX1, HOXC5, and PKNOX1 are differentially associated between the two subtypes. These are all TFs involved in the "Wnt/β-catenin signaling" pathway, which is well known for its role in cancer development and progression [100] and was also enriched for the epigenetically controlled TFs in our analysis of DRAGON edges. It is finally worth mentioning that the cancer driver TFs are only over-represented in cluster E, with 19 TFs (CAMTA1, HLF, IRF4, STAT5A, HOXB13, ETV4, GATA2, STAT5B, HOXC13, FOXA1, ATF1, MGA, PRRX1, IKZF3, ERF, HOXA13, DOT1L, MNX1, TP53) annotated in the OncoKB dataset (Fisher's exact test OR = 1.84, p-value = 0.005), and 15 annotated in the Cancer Gene Census dataset (Fisher's exact test OR = 1.84, p-value = 0.013). In this module, we do not see differentially associated edges between CMS2 and CMS4, which suggests a shared contribution of methylation to cancer etiology.

Since somatic DNA variation is well-studied in the context of cancer and has previously been established as characteristic of the CMS1 subtype, we have focused the above investigation on the associations between promoter methylation and expression, data types that have not yet been ethoroughly explored. However, as an illustrative example of the flexibility of *tcga-data-nf* to include additional omics, we have also generated CNV-expression DRAGON networks that capture associations between copy number changes at the gene level and expression. Analogously to what we described above for the methylation-expression DRAGON networks, we now have three types of edges: CNV-CNV (C_i, C_j), CNV-expression (C_i, E_j), and expression-expression (E_i, E_j). By exploring these edge distributions, we demonstrate that CNV-expression DRAGON networks can also detect key effects of DNA aberrations, as follows.

As we did for the methylation-expression DRAGON networks, we first confirmed the validity of these CNV-expression networks. CNV usually involves deletions or duplications of DNA segments. For this reason, genes that are near each other on the genome are expected to have similar CNV values. This effect was also visible in the adjacency matrices, where there is an evident block structure in the CNV-CNV edges (Supplementary Figure S9A). Moreover, we have evidence that, even in this case, DRAGON networks can capture direct effects of CNV onto expression; the CNV-expression edges on the same TFs (C_i, E_i) tend to be skewed towards positive values (Supplementary Figure S9B), which is expected behavior for somatic variation. Within the top 20 TFs that showed high correlation between their own CNV and expression, we observed known colon cancer drivers [88] ZNF703 and SMAD4, and IRF2 and GATA6, whose DNA aberrations are likely oncogenic [83] (Supplementary Figure S9B).

Finally, we explored the greatest differences between CMS2 and CMS4 subtypes in terms of partial correlations between CNV and expression. For each CNV node C_i, we compared the edge set $\{(C_i, E_j)\}$ between the two subtypes. We conducted a paired Wilcoxon signed-rank test for each TF i, testing the null hypothesis that there is no difference in CNV-expression edge weights between CMS2 and CMS4 (Supplementary Figure S9C). Among the top TFs with varying edges between CMS2 and CMS4 based on the Wilcoxon test results were ZNF554, for which we have partial evidence of involvement in cancer progression through the WNT/β-Catenin Signaling Pathway [101], KLF16, which plays a role in the stress-related programming of colorectal cancer [102], and DOT1L, a methyltransferase that regulates core stem cell genes and affects tumorigenesis and drug resistance [103]. These TFs are significantly associated with colon cancer drivers. When comparing the Wilcoxon test statistics of the cancer TFs to the rest of the TFs, we get a significant Kolmogorov-Smirnov test (stat = 0.21, p-value = 0.013). Also, there are 27 significant (FDR < 0.01) TFs that are annotated by either the Cancer Gene Census or the DisGeNET dataset as colon cancer drivers (Fisher's exact test OR = 1.6, p-value = 0.057, Supplementary Figure S9D). Among the cancer drivers that show the biggest association changes between CMS2 and CMS4, we found HIF1A and CUX1, both of which are implicated in tumorigenesis and progression [89], and GATA4, which controls senescence [104, 105].

Regulatory differences between colon cancer subtype GRNs

The DRAGON networks that we inferred capture information about patterns of DNA methylation and how these patterns are associated with transcription factor expression. The working hypothesis behind this analysis was that some transcription factors exhibit altered patterns of methylation that affect gene expression and ultimately exert downstream effects that help to define different cancer subtypes. Indeed, in the DRAGON networks constructed using transcription factors, we identified several edges that differ between the CMS2 and CMS4 subtypes, providing a plausible explanation for the differences they present in the clinical context. However, the partial correlations represented by edges in DRAGON networks are limited in that they are measures of association and do not capture the actual regulatory effects that TFs have on their target genes.

PANDA is a GRN inference method that uses prior knowledge on motif binding and TF-TF physical interactions together with assayed gene expression to generate a bipartite graph associating TFs with the genes they likely regulate. Each TF-gene edge is weighted by a measure of the evidence of a regulatory relationship. We generated PANDA GRN networks using the TCGA-COAD data, enabling us to perform an analysis aimed at identifying the regulatory context for the TFs identified in clusters A and D, for which we have evidence of changes in methylation and expression patterns between CMS2 and CMS4. After identifying the TFs using the DRAGON analysis, we proceeded to investigate the functional role of their targets.

We compared the two PANDA networks and selected the edges with the greatest differences in edge weights between CMS2 and CMS4 to identify patterns of differential regulation attributable to the selected TFs from the DRAGON analysis. A gene set enrichment analysis on these differentially targeted genes reveals that they are involved in the transcriptional misregulation of cancer and various immune-related Reactome pathways that include "Creation of C4 and C2 activators," "Initial triggering of complement," and "Signaling by the B Cell Receptor (BCR)" (Supplementary Tables S1,S2). Moreover, TFs in cluster D exhibit consistent differential targeting of genes in the "TGF-β signaling," "Cytokine-cytokine receptor interaction," and "Antigen processing and presentation" pathways (Supplementary Figure S10 and Supplementary Tables S3, S4). These pathways are consistent with prior evidence that TGF-β is not only associated with poorer prognosis in colon cancer, but it is also involved in the transition from CMS2 to CMS4 [81].

Finally, we extended our search beyond the TFs identified by DRAGON and performed a genome-wide comparison between the two PANDA networks with ALPACA. ALPACA identifies submodules that optimize the differential modularity of two networks, finding 14 differential modules between CMS2 and CMS4. First, we checked the overall differences in network connectivity by looking at the differential in-module and out-module degrees of genes and TFs (details in the Methods section). Modules 1, 2, and 4 are those exhibiting the most significant difference in connectivity between CMS2 and CMS4 (Supplementary Tables S5 and S6).

ALPACA uniquely assigns TFs and genes in the network to modules and also provides a ranking of nodes based on their contribution to modularity. We can thus use ALPACA results to investigate the functional role of each module. For example, we find that Module 1 is enriched for many immune-related pathways, including "Immune System", "Immune Disease," and "Infectious Disease," cytokine-related signaling pathways, "TNF signaling pathway," and "NF-κB" (Figure 2E). Of the TFs that have the highest contribution to modularity for Module 1, the majority have been validated as key regulators of colorectal cancer (Figure 2F). Indeed, 6 out of 10 leading TFs have direct evidence, and some experimental validation, of their involvement in colon cancer risk and progression: ZNF334 [106], FOXD2 [107], FOXD3 [108], POU3F3 [109], NR1H4 [110], and VSX2 [111].

We performed a similar analysis on all the other modules. Module 2 appears to be more cancer-specific as it is consistently enriched for cell cycle pathways ("Cell cycle," "Cellular senescence"), known cancer-related signaling pathways ("mTOR" and "JAK-STAT" signaling pathways), metabolic pathways, and cancer-specific disease terms (Supplementary Figure S11). Module 4 is strongly enriched for signaling pathways, including "Wnt signaling," and development and regeneration pathways (Supplementary Figure S11). While we know that zinc finger proteins (ZNF) are still not well characterized, hindering the functional annotation of module 4 where 9 out of 10 top TF are ZNFs, we found that ZNF341, which is the fourth highest-ranking hit for modularity contribution (Supplementary Figure S12), is a regulator of STAT3 and plays a key role in hyper-IgE syndrome (HIES), a condition that often manifests as severe and chronic bacterial infections [112]. In parallel, we found reports of associations between IgE-mediated immune reactions and colorectal cancer risk [113]. Although we do not have definitive evidence or validation of the role of ZNF341 in the colon cancer subtypes, we believe that this suggestive result demonstrates how multi-omic network analysis can find complex regulatory patterns. Taken together, the analysis of Modules 1,2, and 4 shows that CMS2 and CMS4 at the regulatory level exhibit differences in the immunological response, cellular processes, such as growth and senescence, and signaling and developmental pathways.

Finally, we examined the relationship between the results of the multi-omic DRAGON analysis (methylation-expression) and ALPACA. It is worth noting that DRAGON, as used here, estimates partial correlations between the methylation and expression of TFs, while ALPACA infers differential modularity between GRNs. Methylation is only one of many possible modifications that can change gene expression, translation, and regulation (mutations, CNVs, chromatin accessibility, etc.). Therefore, we do not necessarily expect DRAGON and ALPACA (used with PANDA-based GRNs) to be concordant. However, using the DRAGON results, we explored whether promoter methylation can explain the differential modularity of these colorectal cancer GRNs.

To interpret these results, we first note that DRAGON uses a different, more stringent filtering method than PANDA. Consequently, only about 50% of TFs in the ALPACA modules can be found in the DRAGON networks (Figure 2G). Nevertheless, we investigated the representation of these overlapping TFs in the ALPACA modules that might indicate that epigenetic changes drive the regulatory differences in a specific Module. Specifically, we looked for TFs in ALPACA modules for which we have evidence of methylation-driven "silencing" in either CMS2 or CMS4, that are those with a strong negative association between expression and methylation of the same TF in DRAGON (Figure 2G, Supplementary Figure S13). While most "silenced" TFs are distributed across the ALPACA modules, in Module 11 we found both the TFs, HOXA10 and HOXB9, "silenced" in CMS2 and not CMS4. This result suggests that these TFs may play an important role in the overall differential regulation of gene expression in the two subtypes. It is also consistent with reports that HOX TFs are differentially accessible in colorectal cancer and that they are involved in cancer development and progression [98].

Testing

Apart from the main workflows for TCGA data analysis, showcased in the previous section, our framework also offers a testing option that assesses the general availability and formatting of data and software and runs a trial analysis on a sample dataset.

Nextflow profiles are sets of configuration parameters accessible with the "–profile" scope. For testing purposes, we provide testing configuration profiles for all workflows, with the following names: "test" for the full pipeline, and "testDownload", "testPrepare", and "testAnalyze" for the specific workflows.

test. This profile runs the full *tcga-data-nf* pipeline on a subset of TCGA Pancreatic Adenocarcinoma data (PAAD). All parameters are specified in the 'conf/test.config'

testDownload. To test the download step, we need to confirm that the TCGA data are available and retrievable, and therefore, we cannot use minimal dummy datasets. To avoid downloading files that are too large, during testing, we only retrieve data from TCGA Pancreas Adenocarcinoma, one of the smallest datasets. All data are downloaded into the 'results/download_test/' folder.

testPrepare. To test the prepare step, dummy expression and methylation datasets are provided that contain randomized sample labels and values. Both datasets have only 10 samples each; consequently, this step computes quickly. Each of the recount pipeline parameters is tested for only one value, such that only one input configuration is tested and only one output is produced.

testAnalyze. To test the analyze step, we provide dummy pre-processed data for expression and methylation data in the `testdata/analyze_expression.csv` and `testdata/analyze_methylation.csv` files. Alongside the input data, we also provide dummy TF-target binding and dummy PPI data, which are necessary to infer PANDA networks. The test computes all network methods (PANDA, DRAGON, LIONESS, and ALPACA) in order to confirm that the whole implementation is functioning properly and the netZooPy and netZooR packages are correctly installed and running.

Computational performance

Nextflow allows users to track computational requirements and performance of all processes. While wall and CPU time heavily depend on the data type and size, estimating CPU and memory usage on sample datasets can help guide users in their choices. First, we report the performance of the pipeline on test data where the analyses were run on an AWS EC2 c5.4xlarge instance with 32Gb of memory and 16 vCPUs. We ran all tests with the v0.0.18 version of *tcga-data-nf* with standard configuration files that allow Nextflow to run all jobs serially, with no job parallelization, where each process is allowed to use all computing resources available. For reference, this would be consistent with testing the workflow on a local machine. The testDownload workflow took 4m 2sec (1.1 CPU hours) and has a peak memory usage of 4.1Gb for the download CNV process. The testPrepare run took 1m 17sec (0.3 CPU hours) and has a peak of 3.5 Gb for GetGeneLevelPromoterMethylation. The testAnalyze run only took 59sec (0.2CPU hours) and has a peak of 3.1 Gb of memory for the runTCGAAlpaca process. We recommend using these tests first to ensure smooth operation of the workflow on dummy data. The test full workflow, instead, requires 1h 48min of wall time (28 CPU hours) and a peak memory of 22 Gb for runTCGAPanda. This is a realistic run of the workflow on the actual TCGA-LUAD data, and includes both ALPACA and LIONESS runs, which are very time-consuming. The *tcga-data-nf* documentation includes the report for this test workflow [114], with a detailed description of the CPU

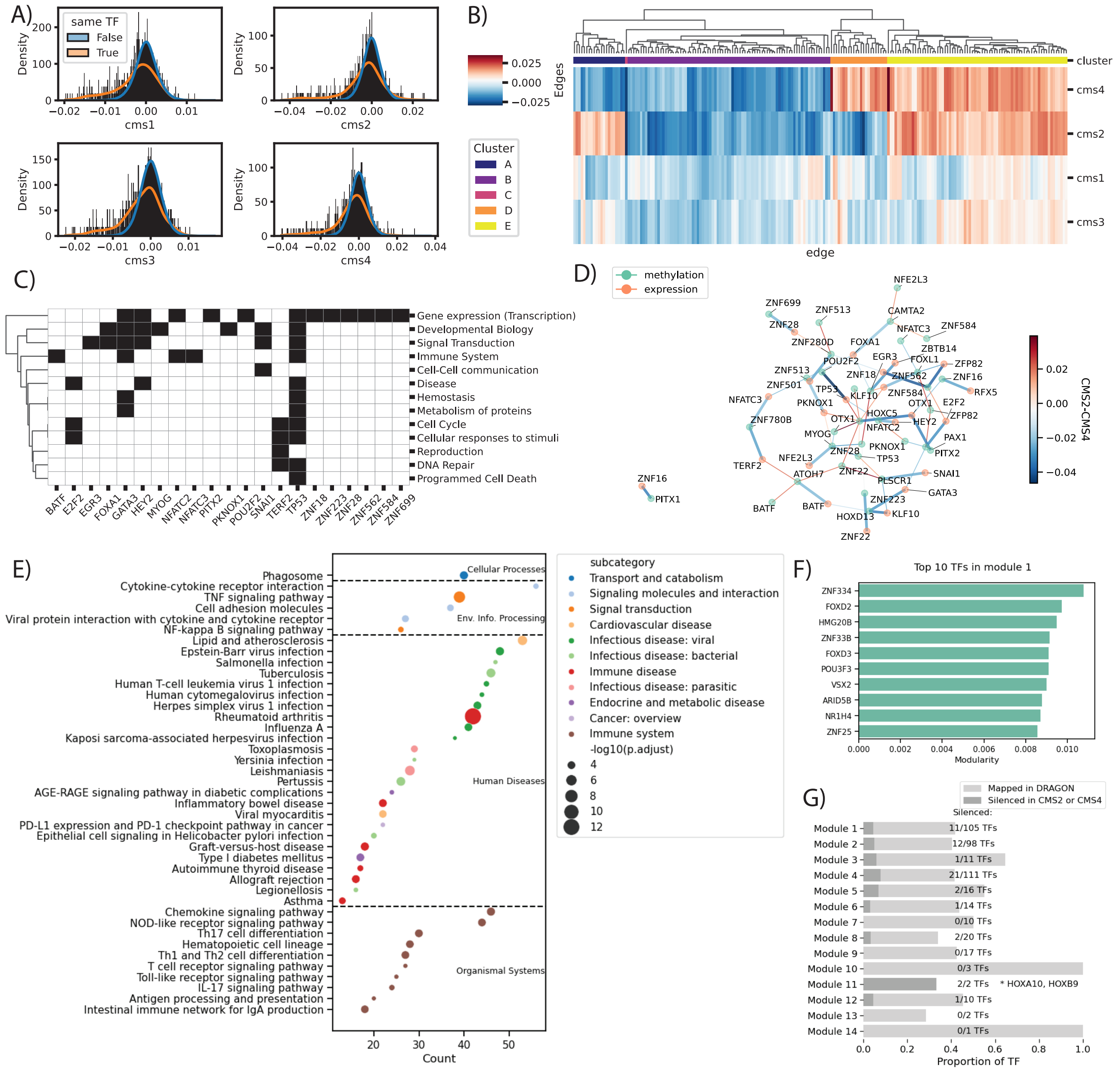

Figure 2. Multi-omic and regulatory differences between colon cancer subtypes A) Distribution of partial correlation DRAGON values between methylation and expression of TFs in all subtypes. In orange, we show the values for the edges of the same TF, that is the correlation between the methylation of the promoter and the expression of that same TF. As expected, methylation and expression tend to be negatively correlated. The histogram represents the distribution density, normalized per subtype and per group. B) For inter-modality DRAGON edge weights (methylation-to-expression), we remove the edges between the same transcription factor, and select the first 200 strongest edges (highest average absolute value of correlation), and cluster them by correlation values for each subtype (average linkage, Euclidean distance). It is interesting that, for cluster A and D, the edge values for CMS2 and CMS4 are swapped in direction. C) Detailed annotation of all TFs in cluster D (columns) to the Reactome parent term. "Immune system" and "Cellular responses to stimuli" are more consistently involved in cluster D, in comparison to cluster A. Here, we can identify which TFs are annotated to each term. For example, a good number of TFs are involved in the immune system (BATF, GATA3, NFATC2, NFATC3, TP53). As expected, many of the TFs are annotated to generic transcription pathways, and TP53 is annotated to almost all terms. D) DRAGON association graph for the TFs in cluster D, showing the difference between the partial correlations in CMS2 and CMS4. We have selected the edges that belong to cluster D (thicker edges), and we have added 20 other top edges, by absolute value, that connect the same TFs (thinner edges), such that we have a connected graph. We show the TFs as nodes with different colors for methylation (green) and expression (orange), and we show edge values color-coded by the differential partial correlation values. E) Pathway analysis (over-representation) of Module 1 of ALPACA comparing CMS2 and CMS4. We show all significant pathways (adjusted p-value < 0.01). The x-axis represents the overlap between Module 1 and each pathway, and the dot size depends on the adjusted *p*-value. Dots are organized by category (annotated on the plot) and colored by subcategory. Many of the terms are relative to "Immune system" (brown dots), and signaling pathways (blue and orange dots). Among the "Human Diseases" terms, many of which involve the immune system, we find PD-L1 expression and the PD-1 checkpoint pathway in cancer. F) Top 10 TFs in module 1 of ALPACA sorted by modularity. G) Overlap between the ALPACA and DRAGON results. In light gray, we show the share of TFs in each ALPACA module that is mapped in DRAGON (around 50% among most modules). In dark gray is the proportion of the TFs that are considered "silencing" in either subtype CMS2 or CMS4. Module 11 has evidence of silencing in CMS2 for both TFs, HOXA10 and HOXB9, known for their developmental role.

and memory performance for each of the processes.

Second, running LIONESS with PANDA is undoubtedly the most expensive process, as it reconstructs one leave-one-out PANDA network for each sample in the population and requires double the memory [115]. However, netZooPy supports GPU-based computation of PANDA-LIONESS networks [116]. On an Nvidia H100 GPU with 32 GB of memory allocated, running PANDA-LIONESS for TCGA-PAAD (183 samples) took 62 minutes, meaning that computing each PANDA-LIONESS network takes approximately 19 seconds.

Discussion

There is a growing recognition that inferring and analyzing gene regulatory network models can provide unique and verifiable insights into the drivers of disease, particularly in rich public databases such as TCGA [34]. Indeed, the GRAND database already provides access to more than 20,000 genome-wide gene regulatory network models, built on publicly available datasets (including TCGA) and used to generate insights in numerous published studies over the years [50]. However, as methods and data evolve, we anticipate that others will be interested in generating and investigating novel networks using TCGA data. Because setting up the requisite environments and data structures to estimate such network models can be challenging, even for those with experience in bioinformatics and computational biology, in this manuscript, we bridge the gap between the wealth of data in TCGA and the skills and computational resources needed to analyze it by providing a user-friendly, standalone workflow.

We developed an end-to-end reproducible workflow capable of downloading, pre-processing, and generating regulatory networks from TCGA cancer data with a single command. This workflow is publicly available, and uses fully open-source software, and adheres to what has been deemed the "gold standard" for reproducibility [3]. The workflow allows the pre-processing of multi-omic data and inference of regulatory networks without requiring users to write code *de novo*; instead, the user needs only to specify a relatively small number of parameters. In addition to the workflow itself, we provide several supporting resources including Docker containers and conda environments, extensive configuration files, documentation describing how to reuse the workflow, and pre-generated networks for the ten most common cancer types in TCGA.

As a demonstration of the value and flexibility of the *tcga-data-nf* pipeline, we applied the workflow to TCGA colon adenocarcinoma (COAD) data. We used *tcga-data-nf* to generate multi-omic association networks (DRAGON) and GRNs (PANDA) for the four consensus molecular subtypes. This is an excellent use case for the work presented here; the ability of the *tcga-data-nf* pipeline to reproducibly and repeatedly run the same workflow with different inputs made it straightforward to analyze different COAD subtypes with multiple network estimation methods. This analysis uncovered evidence of previously undescribed methylation-gene expression interactions that suggest epigenetic regulation of TGF-β signaling and may help to explain factors influencing the transition between subtypes CMS2 and CMS4. To fully showcase the use of *tcga-data-nf* we also computed DRAGON networks for CNV and expression. We used ALPACA to analyze the differences between CMS2 and CMS4 PANDA networks, providing further evidence for relevant epigenetic silencing of critical TFs in the CMS2-CMS4 transition. The code for this example is available on GitHub [117], and the data have been archived on the Harvard Dataverse [118], ensuring complete reproducibility of the analysis.

The biggest challenge we faced in designing *tcga-data-nf* was balancing the trade-off between flexibility and completeness. We reasoned that individual pieces of software, such as GDC, TCGABiolinks, edgeR, and netZooPy, already provide a broad set of functions covering all the steps required for network generation and analysis. As such, the workflow was designed to chain these tools seamlessly together, allowing users to carry out complex analyses simply by specifying a small number of parameters. While flexible by design, the release version of *tcga-data-nf* does not provide users with unlimited options. For example, we provide only two commonly used RNA-seq normalization methods; the full workflow can only be applied to single tumor types and cannot generate pan-cancer analyses without post-hoc coding, and the workflow does not cover all possible data types available from TCGA, such as miRNA expression. However, the flexibility of *tcga-data-nf*'s design should allow users to overcome these limitations; we provide individual Download , Prepare , and Analyze steps that can be used separately from the rest of the pipeline and run with custom input data. We have also provided examples of how the pipeline can be modified and expanded for different analyses—for example, downloading additional data modalities or running other GRN inference or analysis methods. Finally, we recognize that a limitation of *tcga-data-nf* is that it was designed to generate GRNs from bulk sequencing data from TCGA, and most of the intermediate steps available are specific to the analysis of such data. While one could, in principle, use *tcga-data-nf* to download scRNAseq data from GDC from the CPTAC3 project, or extend the Analyze steps to include SCORPION [119] to infer GRNs from single-cell transcriptomics data, creating a dedicated workflow would handle the single-cell data more efficiently.

Overall, *tcga-data-nf* addresses a clear need for reproducible, easy-to-use, and efficient network analysis workflows for large-scale cancer data. In this paper, we demonstrate how this pipeline can be utilized to generate new insights into multi-omic data from TCGA, and we provide comprehensive documentation and supplemental resources to aid researchers in navigating pre-computed networks or generating new ones. We have also illustrated the modularity of the pipeline and its extensibility to other tools within and beyond the Network Zoo. Not only do we expect *tcga-data-nf* to grow as we develop novel network analysis methods, but we also envision *tcga-data-nf* contributing broadly to future biomedical research, serving as the foundation for other GRN analysis workflows.

Methods

Pathway Analysis

In the section "Multi-omic partial correlation networks identify differences between colon subtypes," we carried out all pathway analysis using the GSEApy package [120]. We tested for over-representation (ORA) of TFs and genes in both the Reactome and KEGG sets of pathways with a hypergeometric test. For both cases, we selected the appropriate background, that is, all TFs in the DRAGON networks or the gene targets in the PANDA networks. The KEGG dataset was downloaded from the GSEApy package as the "KEGG2021" dataset. For all tests, we applied the Benjamini-Hochberg procedure to control the false discovery rate (FDR) during multiple testing [121]. We downloaded the pathway files from the Reactome database on June 18, 2024. We downloaded the tables that map each gene identifier to a pathway—and that also map each pathway to its parent terms. For instance "Intracellular signaling by second messengers," "Signaling by GPCR," "Signaling by Hedgehog," etc., are all part of the "Signaling Transduction" term. To reduce the number of tested pathways, which also reduces overlaps between pathways, we have generated a "slim" set of pathways. For each leaf in the Reactome dataset, we have retained only the "parent" node. In this way, we avoid keeping all the nodes that are too small and keep only the depth-1 term.

In the section "Regulatory differences between colon cancer subtypes GRNs" we used R's clusterProfiler package [122] on ALPACA results to run the KEGG pathway analysis. Test p-values were corrected with Benjamini-Hochberg procedure, and we considered as significant those with FDR$<$ 0.05.

All code and data used for the pathway analysis are in the 'tcga-data-supplement' repository [117].

Reference data

PANDA uses "prior knowledge" on putative TF-motif binding and a TF-TF "cooperativity prior" based on TF protein-protein interaction data (PPIs) together with correlations between genes computed from the expression profiles of all samples. To create

a regulatory motif network, we downloaded TF motifs for *Homo sapiens* with direct or inferred evidence from the Catalog of Inferred Sequence Binding Preferences (CIS-BP) Build 2.0, accessible at http://cisbp.ccbr.utoronto.ca. These TF position weight matrices (PWM) were mapped to the human genome (hg38) using FIMO [123]. We retained only highly significant matches ($p \leq 10^{-5}$) occurring within the promoter regions of Ensembl genes (specifically, GENCODE v39 annotations retrieved from http://genome.ucsc.edu/cgi-bin/hgTables). These promoter regions were defined as the interval of [-750; +250] base pairs centered around the transcription start site (TSS). This process yielded an initial set of potential regulatory interactions involving 997 TFs that collectively targeted 61,485 genes.

For the TF-TF cooperativity prior, we obtained PPI data from the StringDB database (version 11.5) using the STRINGdb Bioconductor package [124]. Subsequently, we filtered the PPI data to retain only interactions between transcription factors in the TF-motif network (using a score threshold index of 0). To maintain consistency in PPI scores, we normalized them by dividing each score by 1000, thereby restricting the values to a range of 0 to 1 for both the PPI dataset and the TF-motif network. Additionally, we set self-interactions between TFs to a value of one. Since PPI networks are inherently undirected, we transformed the data into a symmetric PPI matrix.

Cancer Datasets

We annotated the TFs to cancer-specific gene collections from a variety of resources. We downloaded the OncoKB [86, 125] gene list on December 13, 2022. We downloaded the Cancer Gene Census v101 [89, 126] on April 25, 2025; for the colon-only genes, we selected those that have the terms 'colon' or 'colorectal' as Tumor Types (Somatic). Finally, we downloaded the DisGeNet v25.1.1 [87, 127] curated gene-disease associations for the term C0009404 (Colorectal Neoplasms) on April 25, 2025.

Differential degree testing

Given two PANDA networks for subtypes CMS2 and CMS4, with weighted edges $e_{u,v}^{(CMS2)}$ and $e_{u,v}^{(CMS4)}$ connecting TF u to gene v, ALPACA partitions the set of P TFs and R genes into C modules that maximize the differential modularity between CMS2 and CMS4. For each gene r in module c, we defined the *in-module differential degree* as:

$$\mathrm{diff}_r(c) = \sum_{p \in \mathcal{P}} (e_{pr}^{CMS2} - e_{pr}^{CMS4})$$

This is the sum of all differences between CMS2 and CMS4 edges connecting the gene to all the TFs P that belong to the same module.

For comparison, for each gene r in module c, we also defined the *out-module differential degree*, accounting for all edges from gene r directed to nodes that do not belong to module c:

$$\mathrm{diff}_r(\neg c) = \sum_{i \notin \mathcal{P}} (e_{ir}^{CMS2} - e_{ir}^{CMS4})$$

For each module, we ran a Wilcoxon signed-rank test between the in-module and out-module gene differential degrees (Supplementary Table S5). We then ran the same analysis with each TF (Supplementary Table S6), where we obtained the degrees by summing up the edge values connecting each TF p to the R genes.

Availability of source code and requirements

tcga-data-nf

- Project name: *tcga-data-nf*
- Project home page: e.g. https://github.com/QuackenbushLab/tcga-data-nf
- Operating system(s): e.g. Platform independent
- Docker: https://hub.docker.com/r/violafanfani/tcga-data-nf
- Programming language: Nextflow, R, Python, bash
- Other requirements: Java, Nextflow
- License: GNU General Public License v3.0
- WorkflowHub SEEK ID: https://workflowhub.eu/workflows/1306?version=1

NetworkDataCompanion

- Project name: *NetworkDataCompanion*, *NDC*
- Project home page: https://github.com/QuackenbushLab/NetworkDataCompanion
- Operating system(s): MacOS, Linux
- Programming language: R
- License: GNU General Public License v3.0
- SciCrunch registry: RRID:SCR_026532
- bio.tools registry: https://bio.tools/NetworkDataCompanion

Notebooks and configuration files

We provide a GitHub repository that contains i) all configuration files mentioned in this manuscript ii) notebooks and supplementary data for the analysis of colon cancer subtypes.

- Project name: tcga-data-supplement
- Project home page: e.g. https://github.com/QuackenbushLab/tcga-data-supplement
- Operating system(s): Linux, MacOS, Windows
- Programming language: Python
- License: MIT

Data availability

We pre-computed networks for 10 common solid tumors: breast invasive carcinoma (BRCA), lung adenocarcinoma and lung squamous cell carcinoma (LUAD, LUSC), kidney renal clear cell carcinoma (KIRC), liver hepatocellular carcinoma (LIHC), pancreatic adenocarcinoma (PAAD), skin cutaneous melanoma (SKCM), stomach adenocarcinoma (STAD), colon adenocarcinoma (COAD), and prostate adenocarcinoma (PRAD). Raw, multimodal data, and processed data are available through AWS {awsdata, and a guide to the configuration files and data structure can be found in the supplemental repository [117]. PANDA and PANDA-LIONESS networks are available on GRAND (v1.7) [50, 128].

Replication data for the "Multi-omic partial correlation networks identify differences between colon subtypes" subsection are stored on the Harvard Dataverse [118].

Declarations

Funding

This work was supported by grants from the National Institutes of Health: ES, CMLR, MBG, VF, JF, KHS, PM, CC, SM, and JQ were supported by R35CA220523; MBG and JQ were also supported by U24CA231846; JQ received additional support from P50CA127003; JQ was supported by R01HG011393; KHS was supported by P01HL114501 and T32HL007427; CMLR was supported by K01HL166376; CMLR and ES were also supported by the American Lung Association grant LCD-821824.

Author's Contributions

Conceptualization: VF, ES, PM, JF, KHS, CMLR and JQ; **Methodology:** VF, KHS, PM, JF, SM, CC; **Software VF, KHS, PM, JF, SM, CC**; **Formal Analysis:** VF; **Resources:** JQ, CMRS, and MBG; **Data Curation:** VF, ES, KHS, PM, and CMLR; **Writing – Original Draft:** VF, KHS; **Writing – Review and Editing:** PM, MBG, JF, KHS, CC, SM, CMLR and JQ; **Visualization:** VF; **Supervision:** JQ, CMLR; **Funding Acquisition:** JQ and CMLR.

References

1. Mesirov JP. Accessible Reproducible Research. Science 2010 Jan;327(5964):415–416.
2. Baker M. 1,500 Scientists Lift the Lid on Reproducibility. Nature 2016 May;533(7604):452–454.
3. Heil BJ, Hoffman MM, Markowetz F, Lee SI, Greene CS, Hicks SC. Reproducibility Standards for Machine Learning in the Life Sciences. Nature methods 2021 Oct;18(10):1132–1135.
4. Munafò MR, Nosek BA, Bishop DVM, Button KS, Chambers CD, Percie du Sert N, et al. A Manifesto for Reproducible Science. Nature Human Behaviour 2017 Jan;1(1):1–9.
5. Gentleman RC, Carey VJ, Bates DM, Bolstad B, Dettling M, Dudoit S, et al. Bioconductor: Open Software Development for Computational Biology and Bioinformatics. Genome Biology 2004;.
6. Grüning B, Dale R, Sjödin A, Chapman BA, Rowe J, Tomkins-Tinch CH, et al. Bioconda: Sustainable and Comprehensive Software Distribution for the Life Sciences. Nature Methods 2018 Jul;15(7):475–476.
7. Jalili V, Afgan E, Gu Q, Clements D, Blankenberg D, Goecks J, et al. The Galaxy Platform for Accessible, Reproducible and Collaborative Biomedical Analyses: 2020 Update. Nucleic Acids Research 2020 Jul;48(W1):W395–W402.
8. Di Tommaso P, Chatzou M, Floden EW, Barja PP, Palumbo E, Notredame C. Nextflow Enables Reproducible Computational Workflows. Nature Biotechnology 2017 Apr;35(4):316–319.
9. Köster J, Rahmann S. Snakemake—a Scalable Bioinformatics Workflow Engine. Bioinformatics 2012 Oct;28(19):2520–2522.
10. Voss K, der Auwera GV, Gentry J. <p>Full-stack Genomics Pipelining with GATK4 + WDL + Cromwell</P>. F1000Research 2017 Aug;6.
11. Sudlow C, Gallacher J, Allen N, Beral V, Burton P, Danesh J, et al. UK Biobank: An Open Access Resource for Identifying the Causes of a Wide Range of Complex Diseases of Middle and Old Age. PLOS Medicine 2015 Mar;12(3):e1001779.
12. Fairley S, Lowy-Gallego E, Perry E, Flicek P. The International Genome Sample Resource (IGSR) Collection of Open Human Genomic Variation Resources. Nucleic Acids Research 2020 Jan;48(D1):D941–D947.
13. Turnbull C, Scott RH, Thomas E, Jones L, Murugaesu N, Pretty FB, et al. The 100 000 Genomes Project: bringing whole genome sequencing to the NHS. BMJ 2018 Apr;361:k1687.
14. Weinstein JN, Collisson EA, Mills GB, Shaw KRM, Ozenberger BA, Ellrott K, et al. The Cancer Genome Atlas Pan-Cancer Analysis Project. Nature Genetics 2013 Oct;45(10):1113–1120.
15. Bailey MH, Tokheim C, Porta-Pardo E, Sengupta S, Bertrand D, Weerasinghe A, et al. Comprehensive Characterization of Cancer Driver Genes and Mutations. Cell 2018 Apr;173(2):371–385.e18.
16. Sanchez-Vega F, Mina M, Armenia J, Chatila WK, Luna A, La KC, et al. Oncogenic Signaling Pathways in The Cancer Genome Atlas. Cell 2018 Apr;173(2):321–337.e10.
17. Ding L, Bailey MH, Porta-Pardo E, Thorsson V, Colaprico A, Bertrand D, et al. Perspective on Oncogenic Processes at the End of the Beginning of Cancer Genomics. Cell 2018 Apr;173(2):305–320.e10.
18. Hoadley KA, Yau C, Hinoue T, Wolf DM, Lazar AJ, Drill E, et al. Cell-of-Origin Patterns Dominate the Molecular Classification of 10,000 Tumors from 33 Types of Cancer. Cell 2018 Apr;173(2):291–304.e6.
19. Weighill D, Ben Guebila M, Glass K, Platig J, Yeh JJ, Quackenbush J. Gene targeting in disease networks. Front Genet 2021 Apr;12:649942.
20. Ellrott K, Bailey MH, Saksena G, Covington KR, Kandoth C, Stewart C, et al. Scalable Open Science Approach for Mutation Calling of Tumor Exomes Using Multiple Genomic Pipelines. Cell Systems 2018 Mar;6(3):271–281.e7.
21. Way GP, Sanchez-Vega F, La K, Armenia J, Chatila WK, Luna A, et al. Machine Learning Detects Pan-cancer Ras Pathway Activation in The Cancer Genome Atlas. Cell Reports 2018 Apr;23(1):172–180.e3.
22. Li J, Lu Y, Akbani R, Ju Z, Roebuck PL, Liu W, et al. TCPA: A Resource for Cancer Functional Proteomics Data. Nature Methods 2013 Nov;10(11):1046–1047.
23. Li J, Akbani R, Zhao W, Lu Y, Weinstein JN, Mills GB, et al. Explore, Visualize, and Analyze Functional Cancer Proteomic Data Using the Cancer Proteome Atlas. Cancer Research 2017 Nov;77(21):e51–e54.
24. Edwards NJ, Oberti M, Thangudu RR, Cai S, McGarvey PB, Jacob S, et al. The CPTAC Data Portal: A Resource for Cancer Proteomics Research. Journal of Proteome Research 2015 Jun;14(6):2707–2713.
25. Ellis MJ, Gillette M, Carr SA, Paulovich AG, Smith RD, Rodland KK, et al. Connecting Genomic Alterations to Cancer Biology with Proteomics: The NCI Clinical Proteomic Tumor Analysis Consortium. Cancer Discovery 2013 Oct;3(10):1108–1112.
26. Cowen L, Ideker T, Raphael BJ, Sharan R. Network Propagation: A Universal Amplifier of Genetic Associations. Nature Reviews Genetics 2017 Sep;18(9):551–562.
27. Sonawane AR, Platig J, Fagny M, Chen CY, Paulson JN, Lopes-Ramos CM, et al. Understanding Tissue-Specific Gene Regulation. Cell reports 2017;21(4):1077–1088.
28. Reyna MA, Haan D, Paczkowska M, Verbeke LPC, Vazquez M, Kahraman A, et al. Pathway and Network Analysis of More than 2500 Whole Cancer Genomes. Nature Communications 2020 Feb;11(1):729.
29. Leiserson MDM, Vandin F, Wu HT, Dobson JR, Eldridge JV, Thomas JL, et al. Pan-Cancer Network Analysis Identifies Combinations of Rare Somatic Mutations across Pathways and Protein Complexes. Nature Genetics 2015 Feb;47(2):106–114.
30. Silverbush D, Cristea S, Yanovich-Arad G, Geiger T, Beerenwinkel N, Sharan R. Simultaneous Integration of Multi-omics Data Improves the Identification of Cancer Driver Modules. Cell Systems 2019 May;8(5):456–466.e5.
31. Belova T, Biondi N, Hsieh PH, Lutsik P, Chudasama P, Kuijjer ML. Heterogeneity in the Gene Regulatory Landscape of Leiomyosarcoma. NAR Cancer 2023 Sep;5(3):zcad037.
32. Lopes-Ramos CM, Kuijjer ML, Ogino S, Fuchs CS, DeMeo DL, Glass K, et al. Gene regulatory network analysis identifies sex-linked differences in colon cancer drug metabolism. Cancer

Res 2018 Oct;78(19):5538–5547.
33. Glass K, Huttenhower C, Quackenbush J, Yuan GC. Passing Messages between Biological Networks to Refine Predicted Interactions. PLOS ONE 2013 May;8(5):e64832.
34. Weighill D, Guebila MB, Lopes-Ramos C, Glass K, Quackenbush J, Platig J, et al. Gene Regulatory Network Inference as Relaxed Graph Matching. Proceedings of the AAAI Conference on Artificial Intelligence 2021 May;35(11):10263–10272.
35. Chen C, Padi M, Joint Inference of Transcription Factor Activity and Context-Specific Regulatory Networks. bioRxiv; 2022.
36. Margolin AA, Nemenman I, Basso K, Wiggins C, Stolovitzky G, Favera RD, et al. ARACNE: An Algorithm for the Reconstruction of Gene Regulatory Networks in a Mammalian Cellular Context. BMC Bioinformatics 2006 Mar;7(1):S7.
37. Alvarez MJ, Shen Y, Giorgi FM, Lachmann A, Ding BB, Ye BH, et al. Network-Based Inference of Protein Activity Helps Functionalize the Genetic Landscape of Cancer. Nature genetics 2016 Aug;48(8):838–847.
38. Saha E, Ben Guebila M, Fanfani V, Fischer J, Shutta KH, Mandros P, et al. Gene regulatory networks reveal sex difference in lung adenocarcinoma. Biol Sex Differ 2024 Aug;15(1):62.
39. Shutta KH, Weighill D, Burkholz R, Guebila MB, DeMeo DL, Zacharias HU, et al. DRAGON: Determining Regulatory Associations Using Graphical Models on Multi-Omic Networks. Nucleic Acids Research 2023 Feb;51(3):e15.
40. GenomicDataCommons;. http://bioconductor.org/packages/GenomicDataCommons/.
41. Morgan MT, Davis SR, GenomicDataCommons: A Bioconductor Interface to the NCI Genomic Data Commons. bioRxiv; 2017.
42. Colaprico A, Silva TC, Olsen C, Garofano L, Cava C, Garolini D, et al. TCGAbiolinks: An R/Bioconductor Package for Integrative Analysis of TCGA Data. Nucleic Acids Research 2016 May;44(8):e71.
43. Mounir M, Lucchetta M, Silva TC, Olsen C, Bontempi G, Chen X, et al. New Functionalities in the TCGAbiolinks Package for the Study and Integration of Cancer Data from GDC and GTEx. PLoS computational biology 2019 Mar;15(3):e1006701.
44. Silva TC, Colaprico A, Olsen C, D'Angelo F, Bontempi G, Ceccarelli M, et al., *TCGA Workflow*: Analyze Cancer Genomics and Epigenomics Data Using Bioconductor Packages; 2016.
45. netZoo;. https://netzoo.github.io/.
46. Ben Guebila M, Wang T, Lopes-Ramos CM, Fanfani V, Weighill D, Burkholz R, et al. The Network Zoo: A Multilingual Package for the Inference and Analysis of Gene Regulatory Networks. Genome Biology 2023 Mar;24(1):45.
47. Padi M, Quackenbush J. Detecting phenotype-driven transitions in regulatory network structure. NPJ systems biology and applications 2018;4(1):16.
48. Kuijjer ML, Tung MG, Yuan G, Quackenbush J, Glass K. Estimating Sample-Specific Regulatory Networks. iScience 2019 Apr;14:226–240.
49. Guinney J, Dienstmann R, Wang X, de Reyniès A, Schlicker A, Soneson C, et al. The Consensus Molecular Subtypes of Colorectal Cancer. Nature Medicine 2015 Nov;21(11):1350–1356.
50. Ben Guebila M, Lopes-Ramos CM, Weighill D, Sonawane AR, Burkholz R, Shamsaei B, et al. GRAND: A Database of Gene Regulatory Network Models across Human Conditions. Nucleic Acids Research 2022 Jan;50(D1):D610–D621.
51. Merkel D. Docker: Lightweight Linux Containers for Consistent Development and Deployment. Linux journal 2014;2014(239):2.
52. Kurtzer GM, Sochat V, Bauer MW. Singularity: Scientific Containers for Mobility of Compute. PLOS ONE 2017 May;12(5):e0177459.
53. Anaconda Software Distribution. Anaconda Inc.; 2020.
54. Grossman Robert L , Heath Allison P , Ferretti Vincent, Varmus Harold E , Lowy Douglas R , Kibbe Warren A , et al. Toward a Shared Vision for Cancer Genomic Data. New England Journal of Medicine 2016;375(12):1109–1112.
55. Wilks C, Zheng SC, Chen FY, Charles R, Solomon B, Ling JP, et al. Recount3: Summaries and Queries for Large-Scale RNA-seq Expression and Splicing. Genome biology 2021;.
56. THE GTEX CONSORTIUM. The GTEx Consortium Atlas of Genetic Regulatory Effects across Human Tissues. Science 2020 Sep;369(6509):1318–1330.
57. Arora S, Pattwell SS, Holland EC, Bolouri H, Uncertainty in RNA-seq Gene Expression Data; 2018.
58. Johnson KA, Krishnan A. Robust Normalization and Transformation Techniques for Constructing Gene Coexpression Networks from RNA-seq Data. Genome Biology 2022 Jan;23(1):1.
59. Collado-Torres L, Nellore A, Kammers K, Ellis SE, Taub MA, Hansen KD, et al. Reproducible RNA-seq Analysis Using Recount2. Nature Biotechnology 2017 Apr;35(4):319–321.
60. Li B, Ruotti V, Stewart RM, Thomson JA, Dewey CN. RNA-Seq Gene Expression Estimation with Read Mapping Uncertainty. Bioinformatics 2010 Feb;26(4):493–500.
61. Robinson MD, McCarthy DJ, Smyth GK. edgeR: A Bioconductor Package for Differential Expression Analysis of Digital Gene Expression Data. Bioinformatics 2010 Jan;26(1):139–140.
62. Chen Y, Chen L, Lun ATL, Baldoni PL, Smyth GK, edgeR 4.0: Powerful Differential Analysis of Sequencing Data with Expanded Functionality and Improved Support for Small Counts and Larger Datasets. bioRxiv; 2024.
63. Johnson WE, Li C, Rabinovic A. Adjusting Batch Effects in Microarray Expression Data Using Empirical Bayes Methods. Biostatistics (Oxford, England) 2007 Jan;8(1):118–127.
64. Leek JT, Johnson WE, Parker HS, Jaffe AE, Storey JD. The Sva Package for Removing Batch Effects and Other Unwanted Variation in High-Throughput Experiments. Bioinformatics 2012 Mar;28(6):882–883.
65. Aran D, Sirota M, Butte AJ. Systematic Pan-Cancer Analysis of Tumour Purity. Nature Communications 2015 Dec;6(1):8971.
66. Infinium Annotation;. https://zwdzwd.github.io/InfiniumAnnotation.
67. Zhou W, Laird PW, Shen H. Comprehensive characterization, annotation and innovative use of Infinium DNA methylation BeadChip probes. Nucleic acids research 2017;45(4):e22–e22.
68. Du P, Zhang X, Huang CC, Jafari N, Kibbe WA, Hou L, et al. Comparison of Beta-value and M-value Methods for Quantifying Methylation Levels by Microarray Analysis. BMC bioinformatics 2010;11:1–9.
69. Liu H, Lafferty J, Wasserman L. The Nonparanormal: Semiparametric Estimation of High Dimensional Undirected Graphs. Journal of Machine Learning Research 2009;10(10).
70. Zhao T, Liu H, Roeder K, Lafferty J, Wasserman L. The Huge Package for High-Dimensional Undirected Graph Estimation in R. The Journal of Machine Learning Research 2012;13(1):1059–1062.
71. Langfelder P, Horvath S. WGCNA: an R package for weighted correlation network analysis. BMC bioinformatics 2008;9(1):559.
72. Huynh-Thu VA, Irrthum A, Wehenkel L, Geurts P. Inferring regulatory networks from expression data using tree-based methods. PloS one 2010;5(9):e12776.
73. Martin FJ, Amode MR, Aneja A, Austine-Orimoloye O, Azov AG, Barnes I, et al. Ensembl 2023. Nucleic Acids Research 2023 Jan;51(D1):D933–D941.
74. Seal RL, Braschi B, Gray K, Jones TEM, Tweedie S, Haim-Vilmovsky L, et al. Genenames.Org: The HGNC Resources in 2023. Nucleic Acids Research 2023 Jan;51(D1):D1003–D1009.
75. Brown GR, Hem V, Katz KS, Ovetsky M, Wallin C, Ermolaeva O, et al. Gene: A Gene-Centered Information Resource at NCBI. Nucleic Acids Research 2015 Jan;43(Database issue):D36–42.
76. Maglott D, Ostell J, Pruitt KD, Tatusova T. Entrez Gene: Gene-Centered Information at NCBI. Nucleic Acids Research 2011

Jan;39(suppl_1):D52–D57.
77. Frankish A, Diekhans M, Jungreis I, Lagarde J, Loveland JE, Mudge JM, et al. GENCODE 2021. Nucleic Acids Research 2021 Jan;49(D1):D916–D923.
78. AnnotationDbi;. http://bioconductor.org/packages/AnnotationDbi/.
79. Du P, Zhang X, Huang CC, Jafari N, Kibbe WA, Hou L, et al. Comparison of Beta-value and M-value Methods for Quantifying Methylation Levels by Microarray Analysis. BMC Bioinformatics 2010 Nov;11(1):587.
80. Ferlay J, Ervik M, Lam F, Colombet M, Mery L, Piñeros M, et al. Global Cancer Observatory: Cancer Today. Lyon, France: international agency for research on cancer 2024;Available from: https://gco.iarc.who.int/today, accessed [19 June 2024].(0):0.
81. Marisa L, Blum Y, Taieb J, Ayadi M, Pilati C, Le Malicot K, et al. Intratumor CMS Heterogeneity Impacts Patient Prognosis in Localized Colon Cancer. Clinical Cancer Research 2021 Sep;27(17):4768–4780.
82. Mouillet-Richard S, Cazelles A, Sroussi M, Gallois C, Taieb J, Laurent-Puig P. Clinical Challenges of Consensus Molecular Subtype CMS4 Colon Cancer in the Era of Precision Medicine. Clinical Cancer Research 2024 Apr;p. OF1–OF8.
83. OncoKB: A Precision Oncology Knowledge Base | JCO Precision Oncology;. https://ascopubs.org/doi/full/10.1200/PO.17.00011.
84. Mattei AL, Bailly N, Meissner A. DNA Methylation: A Historical Perspective. Trends in Genetics 2022 Jul;38(7):676–707.
85. Chakravarty D, Gao J, Phillips S, Kundra R, Zhang H, Wang J, et al. OncoKB: A Precision Oncology Knowledge Base. JCO Precision Oncology 2017 Dec;(1):1–16.
86. Suehnholz SP, Nissan MH, Zhang H, Kundra R, Nandakumar S, Lu C, et al. Quantifying the Expanding Landscape of Clinical Actionability for Patients with Cancer. Cancer Discovery 2024 Jan;14(1):49–65.
87. Piñero J, Ramírez-Anguita JM, Saüch-Pitarch J, Ronzano F, Centeno E, Sanz F, et al. The DisGeNET knowledge platform for disease genomics: 2019 update. Nucleic Acids Research 2019 11;48(D1):D845–D855. https://doi.org/10.1093/nar/gkz1021.
88. Sondka Z, Bamford S, Cole CG, Ward SA, Dunham I, Forbes SA. The COSMIC Cancer Gene Census: Describing Genetic Dysfunction across All Human Cancers. Nature Reviews Cancer 2018 Nov;18(11):696–705.
89. Tate JG, Bamford S, Jubb HC, Sondka Z, Beare DM, Bindal N, et al. COSMIC: the Catalogue Of Somatic Mutations In Cancer. Nucleic Acids Research 2018 10;47(D1):D941–D947. https://doi.org/10.1093/nar/gky1015.
90. Tan SH, Nevalainen MT. Signal Transducer and Activator of Transcription 5A/B in Prostate and Breast Cancers. Endocrine-Related Cancer 2008 Jun;15(2):367–390.
91. Haddad BR, Gu L, Mirtti T, Dagvadorj A, Vogiatzi P, Hoang DT, et al. STAT5A/B Gene Locus Undergoes Amplification during Human Prostate Cancer Progression. The American Journal of Pathology 2013 Jun;182(6):2264–2275.
92. Di Palma T, Lucci V, de Cristofaro T, Filippone MG, Zannini M. A Role for PAX8 in the Tumorigenic Phenotype of Ovarian Cancer Cells. BMC cancer 2014 Apr;14:292.
93. Jesse S, Koenig A, Ellenrieder V, Menke A. Lef-1 Isoforms Regulate Different Target Genes and Reduce Cellular Adhesion. International Journal of Cancer 2010 Mar;126(5):1109–1120.
94. Chiang YT, Wang K, Fazli L, Qi RZ, Gleave ME, Collins CC, et al. GATA2 as a Potential Metastasis-Driving Gene in Prostate Cancer. Oncotarget 2014 Jan;5(2):451–461.
95. Mellor P, Deibert L, Calvert B, Bonham K, Carlsen SA, Anderson DH. CREB3L1 Is a Metastasis Suppressor That Represses Expression of Genes Regulating Metastasis, Invasion, and Angiogenesis. Molecular and Cellular Biology 2013 Dec;33(24):4985–4995.
96. Scheibner K, Schirge S, Burtscher I, Büttner M, Sterr M, Yang D, et al. Epithelial cell plasticity drives endoderm formation during gastrulation. Nature cell biology 2021;23(7):692–703.
97. Hanahan D. Hallmarks of cancer: new dimensions. Cancer discovery 2022;12(1):31–46.
98. Heide T, Househam J, Cresswell GD, Spiteri I, Lynn C, Mossner M, et al. The Co-Evolution of the Genome and Epigenome in Colorectal Cancer. Nature 2022 Nov;611(7937):733–743.
99. Martínez-Zamudio RI, Stefa A, Freitas JANLF, Vasilopoulos T, Simpson M, Doré G, et al. Escape from oncogene-induced senescence is controlled by POU2F2 and memorized by chromatin scars. Cell Genomics 2023;3(4).
100. Zhan T, Rindtorff N, Boutros M. Wnt signaling in cancer. Oncogene 2017;36(11):1461–1473.
101. Guo Y, Huang C, Xu C, Qiu L, Yang F. Dysfunction of ZNF554 promotes ROS-induced apoptosis and autophagy in Fetal Growth Restriction via the p62-Keap1-Nrf2 pathway. Placenta 2023;143:34–44.
102. Ma XD, Xu SD, Hao SH, Han K, Chen JW, Ling H, et al. KLF16 Enhances Stress Tolerance of Colorectal Carcinomas by Modulating Nucleolar Homeostasis and Translational Reprogramming. Molecular Therapy 2022 Aug;30(8):2828–2843.
103. Kurani H, Slingerland JM. DOT1L Mediates Stem Cell Maintenance and Represents a Therapeutic Vulnerability in Cancer. Cancer Research 2025 Mar;85(5):838–847.
104. Bhat AA, Sharma A, Pope J, Krishnan M, Washington MK, Singh AB, et al. Caudal homeobox protein Cdx-2 cooperates with Wnt pathway to regulate claudin-1 expression in colon cancer cells. PloS one 2012;7(6):e37174.
105. Patel RS, Romero R, Watson EV, Liang AC, Burger M, Westcott PM, et al. A GATA4-regulated secretory program suppresses tumors through recruitment of cytotoxic CD8 T cells. Nature communications 2022;13(1):256.
106. Yang B, Tang H, Wang N, Gu J, Wang Q. Targeted DNA Demethylation of the ZNF334 Promoter Inhibits Colorectal Cancer Growth. Cell Death & Disease 2023 Mar;14(3):1–10.
107. Kim HM, Kang B, Park S, Park H, Kim CJ, Lee H, et al. Forkhead Box Protein D2 Suppresses Colorectal Cancer by Reprogramming Enhancer Interactions. Nucleic Acids Research 2023 Jul;51(12):6143–6155.
108. Li K, Guo Q, Yang J, Chen H, Hu K, Zhao J, et al. FOXD3 Is a Tumor Suppressor of Colon Cancer by Inhibiting EGFR-Ras-Raf-MEK-ERK Signal Pathway. Oncotarget 2016 Dec;8(3):5048–5056.
109. Shan TD, Xu JH, Yu T, Li JY, Zhao LN, Ouyang H, et al. Knockdown of Linc-POU3F3 Suppresses the Proliferation, Apoptosis, and Migration Resistance of Colorectal Cancer. Oncotarget 2015 Oct;7(1):961–975.
110. Lee YJ, Lee EY, Choi BH, Jang H, Myung JK, You HJ. The Role of Nuclear Receptor Subfamily 1 Group H Member 4 (NR1H4) in Colon Cancer Cell Survival through the Regulation of c-Myc Stability. Molecules and Cells 2020 May;43(5):459–468.
111. Shah R, Jones E, Vidart V, Kuppen PJK, Conti JA, Francis NK. Biomarkers for Early Detection of Colorectal Cancer and Polyps: Systematic Review. Cancer Epidemiology, Biomarkers & Prevention 2014 Sep;23(9):1712–1728.
112. Béziat V, Fieschi C, Momenilandi M, Migaud M, Belaid B, Djidjik R, et al. Inherited Human ZNF341 Deficiency. Current opinion in immunology 2023 Jun;82:102326.
113. Hay Fever and Asthma as Markers of Atopic Immune Response and Risk of Colorectal Cancer in Three Large Cohort Studies | Cancer Epidemiology, Biomarkers & Prevention | American Association for Cancer Research;. https://aacrjournals.org/cebp/article/22/4/661/69817/Hay-Fever-and-Asthma-as-Markers-of-Atopic-Immune.
114. Execution report test;. https://htmlpreview.github.io/?https://github.com/QuackenbushLab/tcga-data-nf/blob/main/assets/execution_reports_tests_aws/execution_report_test_full.html.
115. Saha E, Fanfani V, Mandros P, Guebila MB, Fischer J, Shutta

KH, et al. Bayesian inference of sample-specific coexpression networks. Genome Research 2024;34(9):1397–1410.

116. Guebila MB, Morgan DC, Glass K, Kuijjer ML, DeMeo DL, Quackenbush J. gpuZoo: Cost-effective estimation of gene regulatory networks using the Graphics Processing Unit. NAR Genomics and Bioinformatics 2022;4(1):lqac002.
117. tcga-data-supplement;. https://github.com/QuackenbushLab/tcga-data-supplement.
118. Fanfani V, Replication Data for: tcga-data-nf. Harvard Dataverse; 2024. https://doi.org/10.7910/DVN/MCSSYJ.
119. Osorio D, Capasso A, Eckhardt SG, Giri U, Somma A, Pitts TM, et al. Population-Level Comparisons of Gene Regulatory Networks Modeled on High-Throughput Single-Cell Transcriptomics Data. Nature Computational Science 2024 Mar;4(3):237–250.
120. Fang Z, Liu X, Peltz G. GSEApy: a comprehensive package for performing gene set enrichment analysis in Python. Bioinformatics 2022 11;39(1):btac757. https://doi.org/10.1093/bioinformatics/btac757.
121. Benjamini Y, Hochberg Y. Controlling the false discovery rate: a practical and powerful approach to multiple testing. Journal of the Royal statistical society: series B (Methodological) 1995;57(1):289–300.
122. Wu T, Hu E, Xu S, Chen M, Guo P, Dai Z, et al. clusterProfiler 4.0: A universal enrichment tool for interpreting omics data. The innovation 2021;2(3).
123. Grant CE, Bailey TL, Noble WS. FIMO: scanning for occurrences of a given motif. Bioinformatics 2011;27(7):1017–1018.
124. Szklarczyk D, Gable AL, Nastou KC, Lyon D, Kirsch R, Pyysalo S, et al. The STRING database in 2021: customizable protein–protein networks, and functional characterization of user-uploaded gene/measurement sets. Nucleic acids research 2021;49(D1):D605–D612.
125. OncoKB;. https://www.oncokb.org/.
126. Cancer Gene Census;. https://cancer.sanger.ac.uk/cosmic/.
127. Disgenet;. https://www.disgenet.com/.
128. GRAND cancer networks;. https://grand.networkmedicine.org/cancers/.

Click here to access/download
Supplementary Material
GIGA-D-24-00535_supplementary.pdf

Click here to access/download
Supplementary Material
gigascience_review_reply_2.pdf

Personal Cover
Click here to access/download;Personal Cover;tcga_pipeline_cover_letter_2.pdf

HARVARD T.H. CHAN SCHOOL OF PUBLIC HEALTH

John Quackenbush
Henry Pickering Walcott Professor
of Computational Biology
and Bioinformatics

Chair, Department of Biostatistics

August 13th, 2025

Dear Dr. Lan,

Attached please find the second revised version of our paper, “Reproducible processing of TCGA regulatory networks,” that we are resubmitting to *GigaScience* as a Technical Note.

In this manuscript we describe, tcga-data-nf, a robust, highly configurable Nextflow workflow that allows users to reproducibly infer gene regulatory networks for the thousands of samples in The Cancer Genome Atlas (TCGA) and that can easily be adapted to working with other large datasets.

Our research group has long been a leader in developing methods for inferring and analyzing GRNs and we have done substantial work to identify common features of regulation among various cancer types. We undertook a project to infer GRNs in each sample and each cancer type represented in TCGA but we soon recognized the need to establish a uniform, validated workflow in which software tools were chained in a logical and justifiable order and that could be easily used by others. In this manuscript we detail the structure and implementation details of the pipeline and then demonstrate how it can be used to identify differences between colon cancer subtypes.

Following the reviewers’ suggestions we have expanded our findings and discussion on colon cancer as follows:

- We expanded our analysis of the results derived from the DRAGON networks, describing how our findings are supported by other published studies and how the DRAGON networks provide new insights beyond what has been reported elsewhere.
- We more systematically tested all gene sets identified in our network analyses for functional enrichment of biological pathways and cancer drivers and found quantitative enrichment of genes in key cancer/proliferation pathways.
- We now include a comparison between ALPACA (applied to PANDA networks) and DRAGON results.
- We have updated the pipeline and provided an updated account of its computational performance.
- We addressed all minor issues noted by the reviewer.

We believe that we have addressed all reviewer’s concerns and correctly edited the manuscript to meet the journal’s standards. A more detailed accounting of the changes we have made can be found in the detailed response included with this submission.

As always, if you have any questions or if we can provide any additional information to assist you in your decision, please let us know.

On behalf of my co-authors and colleagues, I offer you my best,

John Quackenbush, Ph.D.
Henry Pickering Walcott Professor of Computational Biology and Bioinformatics
Chair, Department of Biostatistics

Department of Biostatistics
655 Huntington Ave
Building II, Room 419
Boston, MA 02115

TEL 617 432 9028
FAX 617 432 5619
johnq@hsph.harvard.edu

https://www.hsph.harvard.edu/john-quackenbush/
