# Supplementary material for: Reproducible processing of TCGA regulatory networks
Source: Gigascience. 2025 Oct 20;14:giaf126. doi: 10.1093/gigascience/giaf126 (PMC12720619; doi:10.1093/gigascience/giaf126)
Supplement: giaf126_Authors_Response_To_Reviewer_Comments_Original_Submission [file giaf126_authors_response_to_reviewer_comments_original_submission.pdf]

Author's Response To Reviewer Comments

Dear Dr. Lan,
Attached please find a revised version of our paper, "Reproducible processing of TCGA regulatory networks," that we are resubmitting to GigaScience as a Technical Note.
In this manuscript we describe, tcga-data-nf, a robust, highly configurable Nextflow workflow that allows users to reproducibly infer gene regulatory networks for the thousands of samples in The Cancer Genome Atlas (TCGA) and that can easily be adapted to working with other large datasets.
Multiomic and gene regulatory network (GRN) modeling has been shown to provide insight into the drivers of health and disease phenotypes. Our research group has long been a leader in developing methods for inferring and analyzing GRNs; our collected tools, "The Network Zoo" (NetZoo), including DRAGON, PANDA, LIONESS, and ALPACA, have been cited more than 700 times. Given our interest in understanding common features among various cancer types, we undertook a project to infer GRNs in each sample and each cancer type represented in TCGA. Because consistency in the inference of so many networks was essential if we were to compare them, we recognized we needed to establish a uniform, validated workflow in which software tools were chained in a logical and justifiable order and that could be easily used by individuals with limited experience in GRN inference and analysis. In this manuscript we detail the structure and implementation details of the pipeline and then demonstrate how it can be used to identify differences between colon cancer subtypes.
Following the reviewers' requests we have now implemented the following substantial changes to the pipeline and the manuscript:

- We added DRAGON for joint analysis of CNV-expression data and ALPACA for comparison of PANDA networks. We also included these tools in our analysis of the regulatory changes that distinguish colon cancer subtypes.
- We documented how to add network inference methods (GENIE3 and WGCNA) that were developed by other groups, increasing the overall impact of our work.
- We improved and extended the "cross-validation" evidence of quality and usefulness of the networks.
- We now provide details on the computational performance of the pipeline.
- We addressed all minor issues raised by the reviewers, both in the code and the manuscript.

We believe that we have addressed all reviewer's concerns and correctly edited the manuscript to meet the journal's standards. A more detailed accounting of the changes we have made can be found in the response letter attached to this submission. As you requested, we have now registered the tools on WorkflowHub, bio.tools, and scicrunch.
As always, if you have any questions or if we can provide any additional information to assist you in your decision, please let us know.
On behalf of my co-authors and colleagues, I offer you my best,
John Quackenbush, Ph.D.
Henry Pickering Walcott Professor of Computational Biology and Bioinformatics

Response to Reviewers
We would like to thank the editor and the reviewers for their comments and guidance. We have now extended the pipeline, improved the NetworkDataCompanion package, and revised the manuscript accordingly. Hopefully, you will find the presentation in the manuscript to be clearer and the pipeline more complete. We have also revised the colon cancer subtype analysis, adding more results obtained by using multiple omics and our group's state-of-the-art network analysis tools (DRAGON, ALPACA) to identify key regulatory differences between the CMS2 and CMS4 colon cancer subtypes.
Following the editor's guidance, we have now registered the workflow on WorkflowHub (SEEK ID: https://workflowhub.eu/workflows/1306?version=1), and NDC on bio.tools (https://bio.tools/NetworkDataCompanion) and scicrunch (RRID:SCR_026532)
Moreover, point-by-point responses (in blue) to all points raised by the reviewers can be found below, together with the original questions/comments.
Reviewer #1:

Fanfani et al. present tcga-data-nf, a Nextflow pipeline that streamlines the download, preprocessing, and network inference of TCGA bulk data (gene expression and DNA methylation). Alongside this pipeline, they introduce NetworkDataCompanion (NDC), an R package designed to unify tasks such as sample filtering, identifier mapping, and normalization. By leveraging modern workflow tools—Nextflow, Docker, and conda—they aim to provide a platform that is both reproducible and transparent. The authors illustrate the pipeline's utility with a colon cancer subtype example, showing how multi-omics networks (inferred via PANDA, DRAGON, and LIONESS) may help pinpoint epigenetic factors underlying more aggressive tumor phenotypes. Overall, this work addresses a clear need for standardized approaches in large-scale cancer bioinformatics.

Major

While tcga-data-nf promises a valuable resource, the following issues should be addressed more thoroughly before publication:

1. While PANDA, DRAGON, and LIONESS form a cohesive system, they were all developed by the same research group. To strengthen confidence, please include head-to-head comparisons with other GRN inference methods (e.g., ARACNe, GENIE3, Inferelator)**. A small benchmark dataset with known ground-truth (or partial experimental validation) would be especially valuable.

We thank the reviewer for the suggestion. While we had organized the workflow to be modular and extensible beyond the netZoo tools, we agree that we had not shown how that could have been done. The goal of this workflow is to enable researchers to generate regulatory networks from cancer datasets and so, as you suggested, we have now added GENIE3 and WGCNA to the pipeline, together with comprehensive documentation on how to add tools to the workflow (add link.XXX). However, we believe that benchmarking the quality of the resulting networks is beyond the scope of our work.

First, the methods are all published and have been benchmarked against various others and the results have been reported in the literature (https://journals.plos.org/plosone/article?id=10.1371/journal.pone.0064832 , https://doi.org/10.1371/journal.pone.0064832, https://doi.org/10.1016/j.isci.2019.03.021, https://academic.oup.com/nar/article/51/3/e15/6931867, https://www.nature.com/articles/s41540-018-0052-5). Second, any new method for GRN inference would require its own peer-reviewed manuscript to prove the correctness and relevance of the output networks. Third, many of these methods are based on different assumptions about networks and produce outputs that differ in structure and require nuanced interpretations. We believe that enabling broader application of existing methods is valuable beyond choosing the "best performing" tool, which itself can generally only be determined in a context-specific manner.

Although the manuscript identifies intriguing TFs and pathways, it lacks confirmation through orthogonal data or experiments. If available, consider including ChIP-seq or CRISPR-based evidence to reinforce at least a subset of inferred regulatory interactions. Even an in silico overlap with known TF-binding sites or curated gene sets would help validate the predictions.

We thank the reviewer for the comment and we have now added more controls on the quality of the inferred networks. For DRAGON edges we show the correlation between our predictions and StringDB co-expression edges, we show some exploratory statistics on the networks to confirm the validity of the predictions (promoter methylation and expression are negatively correlated, while CNV and expression on the same gene are positively correlated) and we have found both methylation-expression and CNV-expression evidence of TFs involvement in cancer progression. Moreover, we have added the ALPACA tool for comparing different PANDA GRNs, which allows identification of differential modules between CMS2 and CMS4. Membership values in those modules allowed us to prioritize TFs assigned to them by PANDA; many of the TFs that we identified using this approach have experimental evidence of a role in colorectal cancer risk and progression. Unfortunately, as these are subtype specific networks we were unable to retrieve orthogonal ChIP-seq or CRISPR-based matched to the "tissue" or subtype, but, we would like to reiterate that these methods have been extensively used, and published work from other labs have used our tools to identify a new TF in RA and have experimentally validated the results (https://www.life-science-alliance.org/content/8/1/e202402808)

1. PANDA and DRAGON emphasize correlation/partial correlation, so they may overlook nonlinear or combinatorial regulation. If feasible, please provide any preliminary steps taken to capture nonlinearities or discuss approaches that could be integrated into the pipeline.

We thank the reviewer for this point. First, we would point out that PANDA includes the effect of transcription factor complexes (PPIs) in its model, which could partially capture combinatorial regulation. Also, we have seen in practice that the appropriate choice of the priors can improve prioritization of TF-gene regulation, and they can be updated to include more complex

regulation mechanisms. To some extent, DRAGON has the potential to capture non-linear or combinatorial regulation in the sense that Gaussian graphical models (partial correlation networks) have been shown to capture known metabolic reactions involving non-linear network motifs such as feedback loops (Krumsiek et al. 2011

https://link.springer.com/article/10.1186/1752-0509-5-21). This is an advantage over correlation-based networks, as Krumsiek et al. demonstrate, and is likely due to the fact that partial correlations between two nodes involve conditioning on the states of the other nodes in the network, while correlations are simply marginal calculations between the two nodes and thus capture both direct and indirect dependence (Shutta et al. 2022 https://doi.org/10.1002/sim.9546)

Nonetheless, we recognize that nonlinearities are harder to manage with our existing tools. For that reason, we would recommend the user to extend the workflow to non-linear methods following the steps at https://github.com/QuackenbushLab/tcga-data-nf/blob/main/extend_workflow.md

1. LIONESS reconstructs a network for each sample in a leave-one-out manner, which can be demanding for large cohorts. **The paper does not mention runtime or memory requirements. Adding a Methods subsection with approximate CPU/memory benchmark**s (e.g., "On an HPC cluster with X cores, building LIONESS networks for 500 samples took Y hours") is recommended to guide prospective users.

We thank the reviewer for the helpful suggestion. We have now added the section “Computational performance” with details on the minimal requirements to run the workflow tests and LIONESS. We have also added the details to the workflow itself, such that users can be guided in the choice of the machine they ultimately choose to use for their analysis.

Currently, the pipeline only covers promoter methylation and standard gene expression, yet TCGA and related projects include other data types (e.g., miRNA, proteomics, histone modifications)**. If possible, offer a brief example or instructions on adding new omics layers,** even conceptually.

We thank the reviewer for the comment. First, we need to acknowledge that the pipeline already includes the download and pre-processing of mutation and CNV data, even though these were not used by the GRN inference methods. However, we adopted this suggestion and enabled the users to run DRAGON networks with CNV data. We have slightly modified the workflow – the metadata and output filename format – to infer DRAGON networks with any omics. For this paper, we have computed CNV-expression networks for the colon cancer subtypes and added the results to the “Multiomics associations identify changes between colon subtypes” section.

Recent methods often target single-cell RNA-seq, but tcga-data-nf is geared toward bulk datasets. **Please clarify limitations and potential extensions for single-cell or multi-region tumor data.** This would help readers understand whether (and how) the pipeline could be adapted to newer high-resolution profiles.

We thank the reviewer for the suggestion. First, we have included more examples on how the workflow can be used and extended to download other GDC-based data (even single cell data)
and how it can be instead used with different datasets. Nonetheless, we have now modified the “Discussion” section to recognize that this specific workflow is designed for enabling seamless processing of bulk tumor data from TCGA, and that scRNAseq or other large-scale projects would benefit from dedicated pipelines.

Minor point:

1. Provide clear guidance on cutoffs for low-expressed genes, outlier samples, and methylation missing-value imputation.
2. Consider expanding the supplement with a "quick-start" guide, offering step-by-step usage examples.
3. Ensure stable version tagging in your GitHub repository so that readers can reproduce the exact pipeline described in the manuscript.

We thank the reviewer for the suggestions. We have expanded the documentation to include clearer guidance on parameters and we have added the quickstart guide to the supplement so that readers have an overview of the online resources. We have now switched to a more stable tagging of the versions.

Reviewer 2:

This manuscript presents tcga-data-nf, a Nextflow-based pipeline for downloading, preprocessing, and analyzing TCGA multi-omic data, with a focus on gene regulatory network (GRN) inference. The workflow integrates established bioinformatics tools (PANDA, DRAGON, and LIONESS) and adheres to best practices for reproducibility through containerization (Docker, Conda, and Nextflow profiles). The authors demonstrate the utility of their pipeline by applying it to colorectal cancer subtypes, identifying potential regulatory interactions in TGF-β signaling.

The manuscript is well-written and well-structured and provides sufficient methodological details, as well as Jupyter notebooks, for reproducibility. However, there are some areas that require clarification and improvement for acceptance in GigaScience, particularly regarding the scope of the tool, the quality of the inferred regulatory networks, the case study figure, benchmarking, statistical validation, and parameters.

Major comments:

While the pipeline is well designed and executed, the overall impact of the tool feels somewhat limited, especially for a journal like GigaScience, due to its pretty specific application to building GRNs in TCGAs, the relatively small number of parameters, the support of only 2 omics type, and the lack of novel algorithms.

We agree with the reviewer that the scope of GRNS in TCGA, is of course, somewhat limited. Below, we detail how we

have extended the pipeline to include more omics types and methods, such that even within its boundaries, tcga-data-nf is broader and more flexible. We would also like to draw the reviewers' attention to the flexibility of NDC, which extends beyond the functions that are directly used in the pipeline. Moreover, based on the wealth of information encapsulated in TCGA data that has yet to be analyzed with network models, we believe this pipeline will serve to provide meaningful biological insights that are relevant for the broader cancer research community.
To increase the impact of this tool I would recommend **adding functionalities**, such as:
- **Supporting additional tools.** A great strength of the pipeline is the integration with the Network Zoo (NetZoo) ecosystem. However, only three tools are included from NetZoo. Including additional tools would likely increase the scope of users interested in using the pipeline. In particular, an important weakness of the current pipeline is that it is not possible to conduct differential analysis between different networks, which prevents users from identifying the most significant differences between two networks of interest (e.g., CMS2 vs CMS4). **The NetZoo contains different tools to conduct such analyses, such as Alpaca 1 or Crane 2,** thus this may be implemented to make the pipeline more useful to a broader user base.
We thank the reviewer for this suggestion and we agree that expanding the pipeline to include other methods would be useful. We have now added ALPACA for differential network analysis, and have incluced a demonstration of its application to the CMS2 vs CMS4 question. Together, we have modified the Results section (subsections: "Analyze", "Full", " Multi-omic partial correlation networks identify differences between colon subtypes") to reflect these changes in the pipeline. We also added CNV-expression DRAGON networks to the pipeline, which are useful to estimate complex effects of DNA aberrations.
o Adding parameters. A strength of the pipeline is the ability to customize it using various parameters. However, as such the pipeline does not offer many parameters. It would be beneficial to make the pipeline a bit more customizable. For example, novel parameters could be: adding options for excluding selected samples, using different batch correction methods, different methods to map CpGs to genes, additional normalization methods, and additional quality controls (e.g., PCA for methylation samples, md5sum checks). These are just examples and do not need to be all implemented but adding some extra parameters would help make the pipeline more appealing and customizable to various users.
We also thank the reviewer for this suggestion. As explained in the discussion of the paper, the workflow attempts to balance flexibility (we allow the user to specify many parameters) and ease-of-use, so that even moderately experienced users can run GRN inference with relatively little effort and a shallow learning curve but still achieve meaningful results. However, as
requested, we have added some features that we thought could benefit the workflow; drawing PCA plots for methylation, filtering duplicate methylation samples based on minimum missing data and parameters (e.g., choice of TCGA tumor purity method, choice of threshold for missingness to exclude vs. impute methylation data). Also, we plan to add more as needed based on user requests or changes in the methods.
- **The quality of the inferred regulatory networks is hard to judge.** There are no direct comparisons with any other tools. For instance, it is mentioned in the text that GRAND networks were derived using a fixed set of parameters, but it could be helpful to show a direct comparison between GRNs built from your tools with those from GRAND. This could reveal how the ability to customize GRNs using the pipeline's parameters helps in getting better biological insights.
o Alternatively, or in addition, one **could compare how networks built by your method fare in comparison to networks** built from other methods, like RegEnrich 3 or NetSeekR 4, in terms of biological insights, accuracy, scalability, speed, functionalities and/or memory usage.
We thank the reviewer for this comment. First, upon re-reading our conclusions we realized that we had not explained our conclusions regarding GRAND. We have now modified the paragraph to explain how GRAND is a dynamic database where published work is stored and accessible, while emphasizing that each user may want to carry out their own individual analyses like we did in this paper. Additionally, since the goal of this workflow is to enable researchers to easily generate regulatory networks from cancer datasets, we have added GENIE3 and WGCNA to the pipeline and have included comprehensive documentation on how users can add their choice of tools to the workflow.
With regard to judging the results, we do not believe this is necessary, as all of the methods included in the workflow have have been published and widely benchmarked in the literature ((
https://journals.plos.org/plosone/article?id=10.1371/journal.pone.0064832 ,
https://doi.org/10.1371/journal.pone.0064832, https://doi.org/10.1016/j.isci.2019.03.021,
https://academic.oup.com/nar/article/51/3/e15/6931867, https://www.nature.com/articles/s41540-018-0052-5).); our intention is to ease the use of these methods in a combined workflow. We do agree that any novel method for GRN inference or benchmarking would require its own peer-reviewed manuscript to prove the fidelity and quality of the output networks, but that is not our goal in implementing this workflow.
o Another angle to judge the regulatory networks would be to check in a case study if the predicted gene interactions between disease and control networks are enriched in disease and gene-gene interactions databases, such as DisGeNet 5.

We thank the reviewer for this idea. We have now added evidence of the relevance of the DRAGON edges we found. We now report (1) negative correlation between methylation and expression on the same TF, (2) positive correlation between CNV and expression, (3), that co-expression network (expression-expression edges) are supported by evidence from the STRINGDB database, (4), edges in cluster A of the DRAGON methylation-expression networks are enriched for TFs that are involved in Colorectal Neoplasms according to the DisGeNet
gene-disease association lists. Please note that we have used the original release of DisGeNet in the analysis reported here: https://academic.oup.com/database/article/doi/10.1093/database/baaf007/7994270

* Figure 2 needs re-work:

o Panel A and C: text is too small. "tf" should be written TF. "oi" should have another name. These panels might be moved to the supplements.

o Panel D is confusing. Without significance it is hard to understand what the point of this panel is. I can see that certain TFs are cited in the main text but without information about significance, these may seem like cherry-picking. The legends states: `Annotation of all TFs in cluster D (columns) to the Reactome parent term. "Immune system" and "Cellular respondes to stimuli" are more consistenly involved in cluster D, in comparison to cluster A.`. However, this is a key result which should be shown in a main figure, not in Figure S6. I would also recommend using a -log scale when displaying the p-values to highlight the most significant entries.

o Panel E is quite confusing; first, the color coding is unclear. For instance, what represents blue, purple and red colors? Second, what represents the edges' widths? I would recommend using different shapes for the methylation and expression nodes to reduce the number of colors, and adding a color legend. I would also consider merging the two graphs and representing in color the difference in the edge values so the reader can directly see the key differences.

We thank the reviewer for the detailed comments and have updated the figure accordingly. After careful consideration, we decided to keep the figure in panel C and update the text to better explain what it represents. Indeed, we only have a small number of TFs and we are interested in showing the general functional annotation terms they represent. These TFs were not preselected or chosen in a biased fashion, but instead encompass all of those in cluster D that were annotated to at least one pathway. Indeed, functional enrichment analysis on TFs is usually more “difficult” as the power is lower (give the total number of TFs) and they are all annotated to the regulatory terms, skewing the adjusted p-value. Nonetheless, it is interesting to see that these TFs present meaningful biological insights.

* Benchmarking analysis could be included to show the runtime and memory requirement for each pipeline step. It would also be beneficial to analyze a larger dataset than colon cancer to assess the scalability.

We thank the reviewer for the helpful suggestion. We have now added the section “Computational performance” with details on the minimal requirements to run the workflow tests and LIONESS. We have also added the details to the workflow itself, such that users can be guided in the choice of the machine.

* Statistical analysis: If computationally feasible, permutation testing could be implemented to quantify the robustness of inferred regulatory interactions. Also, in the method section, it should be clarified that FDR correction was applied for pathway enrichment analysis.

We thank the reviewer for these points. We have now revised and expanded the methods section. Regarding robustness, we agree that this is a significant question and that additional methods to quantify robustness would be useful. However, we also note that before showing statistical significance, one should investigate how to properly randomize data for each of these methods, including to provide good empirical estimates of the confidence intervals, or analytical work should be done to infer closed form solutions for the confidence interval; all of this would require its own detailed analysis and is beyond the scope of our current work.

Minor comments:

* I am not sure why duplicate samples are discarded in the pipeline. Why not add counts for RNA-Seq and averaging beta values? I would expect that to yield more robust results.

We thank the reviewer for the comment. From visually inspecting some of the data we had noticed that some of the duplicated samples, with lower depth, seemed very sparse. NDC does not support combining duplicated samples as there may be important technical differences that should not be masked (for example, contamination of a vial or differences in bulk tissue cell type composition). However, we have extended the options for selecting the duplicate based on missingness in methylation data (i.e., select the sample with the least overall missingness).

* It is a bit unclear in what context the NetworkDataCompanion tool could be used outside the workflow. It is also unclear how it helps with quality controls. Please clarify these aspects.

We thank the reviewer for this point. We have now included a quickstart and documentation for NetworkDataCompanion such the barrier to use is lower. The quickstart is provided as an RMarkdown file (https://github.com/QuackenbushLab/NetworkDataCompanion/blob/main/quickstart.Rmd) so that users can download and run it directly. The rendered HTML can also be viewed at
https://htmlpreview.github.io/?https://github.com/QuackenbushLab/NetworkDataCompanion/blob/main/quickstart.html.
In addition, we wish to highlight that while some of NDC’s functions are specifically designed for wrangling TCGA data,

many can be broadly applied to any set of gene expression data that is stored in RangedSummarizedExperiment objects and to any matrix of methylation beta values with an accompanying manifest of the appropriate format. We believe that the impact of NDC is therefore broadly relevant to the readers of GigaScience

There are two key aspects of NetworkDataCompanion that ensure quality control in the Prepare step of the pipeline. First, NetworkDataCompanion incorporates unit tests for every function. These tests are toy examples where the function of the code is checked by asserting equality between results calculated by NetworkDataCompanion and “gold standard” results calculated manually. The tests are built with every addition to the package via continuous integration through GitHub actions, so if a contributor makes changes to the code, it is automatically checked that there are no problems caused to the existing functions. Second, NetworkDataCompanion is version-controlled via GitHub. This facilitates reproducibility of tcga-data-nf workflows: as NetworkDataCompanion evolves, users can document which version of the repository they have used in their analysis and that exact code will continue to be accessible via GitHub’s versioning scheme.

* The manuscript is well-written, but words are sometimes missing or wrongly written, it needs careful re-read.

We thank the reviewer for pointing this out. We have gone through the paper and edited it to address missing words and incorrect word choice.

* The expression '"same-same"' is unclear to me. In this sentence: "Some of "same-same" genes (STAT5A, CREB3L1"…, I am not sure in which table or figure I can find this result?

We apologize for the colloquial language and have revised the expression and rephrased the paragraph. Also, we have added Supplementary Figure S6 that summarizes these results.

* Text is too small in the Directed Acyclic Graph, especially in Figure S4. Also, I would recommend adding the Directed Acyclic Graphs from Figure S1-S4 to the online documentation.

We thank the reviewer for the comment, and have now fixed all figures and added them to online documentation. Since we were not able to produce a Figure S4 with visible names, we opted for keeping it only in the online documentation, while we made figures S1-S3 larger.

* Regarding the code, I was puzzled to see a copyConfigFiles process. Also, there are files in `bin/r/local_assets`, these should be located in `assets`. And the container for the singularity and docker profile is likely the same, this should be clarified in the code.

We thank the reviewer for this suggestion and we have now moved the local assets. Regarding copyConfigFiles. In practice, we have noticed that manually copying all configuration files into the output folder is helpful for reproducibility, especially for less experienced users.

* It is recommended to remove the "defaults" channel from the list of channels declared in the `containers/conda_envs/analysis.yml` file. Please see information about that here[https://www.anaconda.com/blog/is-conda-free] (https://www.anaconda.com/blog/is-conda-free) and here[https://www.theregister.com/2024/08/08/anaconda_puts_the_squeeze_on/] (https://www.theregister.com/2024/08/08/anaconda_puts_the_squeeze_on/).

We thank the reviewer for this suggestion! This was part of the reason we moved the containers away from using conda, but we have now followed their suggestions and removed the default channel from the environment’s yaml file.

Additional comments (which do not need to be addressed):

* Future work may consider enabling the use of the pipeline to build GRNs from other data sources than TCGA (i.e., nf-netzoo). Recount3 data is already being parsed for GTEx and TCGA samples, so it might be relatively easy to adapt the pipeline so that it can be used on any arbitrary recount3 dataset. Similarly, it could be useful if one could specify a dataset on the recountmethylation database 6 to build GRNs. While these unimodal datasets could not be used with the DRAGON method they would still benefit from all other features of the pipeline.

We are indeed considering a method-centric pipeline that would be independent of TCGA, but that needs to be addressed separately. These are all great suggestions that we’ll definitely consider as we continue to build and refine the pipeline!

* Using a nf-core template would enable better structure of the code and increase the visibility of the tool. Also using multiple containers is usually easier to maintain and update than a single large container, especially when a single tool needs to be updated or when modifying part of the pipeline. Another comment is that the code contains many comments which are not to explain the code but more like quick draft which makes the code harder to read by others.

We had thought about adding it to nf-core, but their guidelines were not compatible with the development of this workflow. We’ll surely consider it for future projects. We have also added more containers, specific for each step, and reviewed the comments, thanks for this suggestion!

References

1. Padi, M., and Quackenbush, J. (2018). Detecting phenotype-driven transitions in regulatory network structure. npj Syst Biol Appl 4, 1-12. https://doi.org/10.1038/s41540-018-0052-5.
2. Lim, J.T., Chen, C., Grant, A.D., and Padi, M. (2021). Generating Ensembles of Gene Regulatory Networks to Assess

Robustness of Disease Modules. Front. Genet. 11.https://doi.org/10.3389/fgene.2020.603264.
3. Tao, W., Radstake, T.R.D.J., and Pandit, A. (2022). RegEnrich gene regulator enrichment analysis reveals a key role of the ETS transcription factor family in interferon signaling. Commun Biol 5, 1-12. https://doi.org/10.1038/s42003-021-02991-5.
4. Srivastava, H., Ferrell, D., and Popescu, G.V. (2022). NetSeekR: a network analysis pipeline for RNA-Seq time series data. BMC Bioinformatics 23, 54. https://doi.org/10.1186/s12859-021-04554-1.
5. Hu, Y., Guo, X., Yun, Y., Lu, L., Huang, X., and Jia, S. (2025). DisGeNet: a disease-centric interaction database among diseases and various associated genes. Database 2025, baae122.https://doi.org/10.1093/database/baae122.
6. Maden, S.K., Walsh, B., Ellrott, K., Hansen, K.D., Thompson, R.F., and Nellore, A. (2023). recountmethylation enables flexible analysis of public blood DNA methylation array data. Bioinformatics Advances 3, vbad020. https://doi.org/10.1093/bioadv/vbad020.

Editorial Manager®

Cookie Preference Center

We use cookies which are necessary to make our site work. We may also use additional cookies to analyze, improve and personalize our content and your digital experience. For more information, see our Cookie Policy.

You may choose not to allow some types of cookies. However, blocking some types may impact your experience of our site and the services we are able to offer. See the different category headings below to find out more or change your settings.

Allow all

Manage Consent Preferences

Strictly Necessary Cookies

Always active

These cookies are necessary for the website to function and cannot be switched off in our systems. They are usually only set in response to actions made by you which amount to a request for services, such as setting your privacy preferences, logging in or filling in forms. You can set your browser to block or alert you about these cookies, but some parts of the site will not then work.

Functional Cookies

These cookies enable the website to provide enhanced functionality and personalisation. They may be set by us or by third party providers whose services we have added to our pages. If you do not allow these cookies then some or all of these services may not function properly.

Performance Cookies

These cookies allow us to count visits and traffic sources so we can measure and improve the performance of our site. They help us to know which pages are the most and least popular and see how visitors move around the site.

Targeting Cookies

These cookies may be set through our site by our advertising partners. They may be used by those companies to build a profile of your interests and show you relevant adverts on other sites. If you do not allow these cookies, you will experience less targeted advertising.

Cookie List

Clear

Apply Cancel

Consent Leg.Interest

Confirm my choices

Powered by onetrust
