# Supplementary material for: Reproducible processing of TCGA regulatory networks
Source: Gigascience. 2025 Oct 20;14:giaf126. doi: 10.1093/gigascience/giaf126 (PMC12720619; doi:10.1093/gigascience/giaf126)
Supplement: giaf126_Authors_Response_To_Reviewer_Comments_Revision_1 [file giaf126_authors_response_to_reviewer_comments_revision_1.pdf]

Author's Response To Reviewer Comments

GigaScience review N2

Reviewer #2:
The authors have developed a well-structured and reproducible workflow for regulatory network inference from TCGA data, integrating established tools and offering valuable infrastructure for the community. The additions of ALPACA, support for more omics types, and improved parameter flexibility are welcome enhancements. However, further work is needed to strengthen the impact of the pipeline, particularly through clearer demonstration of the biological significance of findings (e.g., through gene sets enrichment analyses). This point if of particular importance, given the lack of benchmark with other tools, and will help better position this resource for broader adoption and scientific utility.

We truly thank the reviewer for providing such a careful and detailed comment to our manuscript. We have addressed and corrected all issues they pointed out and below you will find our detailed response.

Majors comments

* Figure 2D: The legend was not changed, it still reads: "Association graph for the TFs in cluster D for CMS2 (left) and CMS4 (right)." This should be fixed. The accompanying text for this panel is "All these differences between CMS2 and CMS4 can also be visualized at once as the subgraph that emerges from cluster D, Figure 2D.", which is clearly not very insightful. I would suggest either describing key insights from this panel, or moving it to the supplements. In the first case, we can now clearly see that some edges are much stronger; like TP53 to POU2F2. It may be interesting to describe in particular such examples, if relevant.

We thank the reviewer for pointing this out, and we apologize for the error; we have fixed the caption accordingly. We also agree that we should have provided more details on what we learned from the results presented in the panel, particularly as these will provide readers not only insight into colorectal cancer but also familiarity with the value of DRAGON networks.

“While we have described the individual role of these TFs, it is important to remember that these differences between CMS2 and CMS4 can also be contextualized as the subgraph that emerges from cluster D (Figure 2D). For instance, there is a clearly strong difference between the association of methylation of POU2F2 and expression of TP53 (edge values CMS2: −0.024, CMS4: 0.021). Positive correlation between TP53 expression and POU2F2 methylation is consistent with evidence that silencing of TP53 and availability of POU2F2 are leading to oncogene-induced senescence escape and tumor progression in colorectal cancer [97]. If we instead focus on interactions between more than two nodes, we can see how PAX1, OTX1, HOXC5, and PKNOX1 are differentially associated between the two subtypes. These are all TFs involved in the “Wnt/β-catenin signaling” pathway, which is well known for its role in cancer development and progression [98] and was also enriched for the epigenetically controlled TFs in our analysis of DRAGON edges.”

* Figure S5: This figure is not very informative as one doesn't know if the presence of certain genes from your network in various databases is higher than expected or just as expected by chance. As such, the figure is of little value, and it is hard to conclude much. I would recommend conducting gene set enrichment analysis to see which gene set (CMS4, CMS2, cluster A, ect) is enriched in which database, and showing the results in an additional supplementary figure.

We thank the reviewer raising this point and we agree that we should have presented our results more clearly. Figure S5 is a detailed version of the tail of Figure 2A and we show the actual TFs for which we have evidence of “silencing”. In the previous version we had only reported the names of some of these genes, and we realized that without a graphic element it was hard to follow.

Also, following the reviewer’s suggestion we have now tested the actual distributions of the (Mi-Ei) edges

for each subtype and the disgenet/cosmic annotations and we show that some of them are known for their role in cancer and we annotate them in this figure to help the reader follow the text and results. We have also carried out pathway ORA and included the results in the Supplementary Figure S5.

We have revised the text as follows:

“To evaluate the relevance of these edges, we investigated the relationship between cancer phenotype and TFs for which we inferred direct epigenetic regulation based on the DRAGON networks. We first considered the actual edge weights (Mi, Ei) of the cancer drivers (according to the COSMIC cancer gene census). We used a Kolmogorov-Smirnov test to check if the cancer-associated TFs have lower edge weights than is the case for TFs that are not annotated as being cancer-associated. We found that colon drivers have lower edge weights in CMS2 (p-value: 0.039) and all drivers have lower edge weights in CMS4 (p-value: 0.088). Then, we selected the TFs for which the (Mi, Ei) edge weights are in the first decile of the distribution of at least one subtype and we found 108 TFs with evidence ofmethylation-driven “silencing” (Supplementary Figure S5). Some of these TFs (STAT5A, CREB3L1, ZNF24, HMGA1, IRF8, PAX8, CDX2, CREB3L2, TFEB, MGA, NFIB, KLF6, LEF1, HOXD13, HOXA13, HOXB13, GATA2) are known to be cancer drivers [83, 84, 85, 86, 87]. For example, STAT5A, the only TF that has low (Mi, Ei) edges in all subtypes, is a known oncogene involved in the JAK signaling cascade [88, 89], while CREB3L1, LEF1, and PAX8 are all known to be involved in invasion and metastasis [90, 91, 92, 93].

We also assessed the over-representation of the TFs under putative epigenetic regulation in the Reactome pathway database using a hypergeometric test. While the pathway analysis on TFs is not sufficiently powered because most pathways contain fewer than 10 TFs, we found nominal enrichment not only for broadly defined transcriptional pathways, such as “Generic Transcription Pathway” (OR: 1.71, p-value:0.0093) and “RNA Polymerase II Transcription” (OR:1.64, p-value 0.014), but also formore specific pathways such as “Formation of paraxialmesoderm” (OR: 31.96, p-value: 0.006), “Gastrulation” (OR: 2.72, p-value: 0.032), “Incretin synthesis, secretion, and inactivation” (OR: 22.63, p-value: 0.033) and “Beta catenin independent WNT signaling” (OR: 6.37, p-value: 0.045) (Supplementary Figure S5). These are all pathways related to the epithelial-mesenchymal transition (EMT), which is an established hallmark of cancer that drives invasion and metastasis [94, 95]. Lastly, to further validate these networks, we compared DRAGON edges with the co-expression edges in the StringDB database and found evidence of correlation with DRAGON expression-expression edges (Supplementary Figure S6).”

Also in the sentence: "edges in cluster A of the DRAGON methylation-expression networks are enriched for TFs that are involved in Colorectal Neoplasms according to the DisGeNet" -> I could not find this information easily/clearly in the Figure. Also, if this is a relevant result it should be mentioned in the main text, not only in the letter to the editor. Enrichment of cancer gene sets in some of your subtypes/clusters could be quite convincing/informative.

We apologize for not including this in the main text. Upon further investigation, we realized that most cancer drivers are annotated to Cluster E and so have included this result in the main text:

“It is finally worth mentioning that the cancer driver TFs are only over-represented in cluster E, with 19 TFs (CAMTA1, HLF, IRF4, STAT5A, HOXB13, ETV4, GATA2, STAT5B, HOXC13, FOXA1, ATF1, MGA, PRRX1, IKZF3, ERF, HOXA13, DOT1L, MNX1, TP53) annotated in the OncoKB dataset (Fisher’s exact test OR: 1.84, p-value: 0.005), and 15 annotated in the Cancer Gene Census dataset (Fisher’s exact test OR: 1.84, p-value: 0.013). In thismodule, we do not see differentially associated edges between CMS2 and CMS4, which suggests a shared contribution of methylation to cancer etiology.”

In terms of style: I would put x-axis labels in a 90 degree angle.

Thank you for the suggestion. We have made this change.

* "and colon cancer drivers HIF1A and CUX1, both of which are implicated in tumorigenesis and progression [84]"; I could not find CUX1 in the list. Also going up to 50 genes seems a bit extreme; I would not describe any gene more than the top 10 or 20. If one wants to include more genes I would do pathway enrichment analysis to show more systematically what this list of genes contains. Because out of

50 genes, one can most likely pick a few that support any given hypothesis.

We thank the reviewer for the suggestions and we have implemented them in the manuscript. We first show the top 20 TFs and briefly describe the involvement of some of them in key processes. We also tested whether TFs annotated to Cancer driver lists (Disgenet and colon cancer drivers from the Cancer Gene Census) had stronger evidence of being different between CMS2 and CMS4. For that we applied both a Fisher's Exact test and a Kolmogorov Smirnov test on the statistics, and we have evidence that they show stronger differences between subtypes than expected by chance. We also include these TFs in the figure.

We have revised the text as follows:

"Among the top TFs with varying edges between CMS2 and CMS4 based on the Wilcoxon test results were ZNF554, for which we have partial evidence of involvement in cancer progression through the WNT/β-Catenin Signaling Pathway [99], KLF16, which plays a role in the stress-related programming of colorectal cancer [100], and DOT1L, a methyltransferase that regulates core stem cell genes and affects tumorigenesis and drug resistance [101]. These TFs are significantly associated with colon cancer drivers. When comparing The Wilcoxon test statistics of the cancer TFs to the rest of the TFs, we get a significant Kolmogorov-Smirnov test (stat: 0.21, p-value: 0.013). Also, there are 27 significant (FDR < 0.01) TFs that are annotated by either the Cancer Gene Census or the DisGeNET dataset as colon cancer drivers (Fisher's exact test OR : 1.6, p-value: 0.057, Supplementary Figure S9D). Among the cancer drivers that show the biggest association changes between CMS2 and CMS4,we found HIF1A and CUX1, both of which are implicated in tumorigenesis and progression [87], and GATA4, which controls senescence [102, 103]."

* "Modules 1 and 2 are enriched for Immune System and Cell Cycle pathways" -> I could not find enrichment for "Cell Cycle pathways" on the figure. Also, and more importantly, I think displaying the number of enriched KEGG subcategories is peculiar and uncommon. This type of analysis can hide for instance a very significant term. I think the authors should provide, in addition, a table with the top significant results to ensure the immune terms are indeed the most significant results. Alternatively, I would directly show the most significant terms on the y-axis of Figure S12.

We thank the reviewer for this comment. We had initially thought that a summary plot would be easier to read, but we agree that an uncommon plot is sometimes harder to read. For this reason, we have now changed the figure to a complete plot of all significant pathways (Supplementary figure S11). We again thank the reviewer because this helped us drawing more meaningful conclusions than presented in the initial manuscript:

"ALPACA uniquely assigns TFs and genes in the network to modules and also provides a ranking of nodes based on their contribution to modularity. We can thus use ALPACA results to investigate the functional role of eachmodule. For example, we find that Module 1 is enriched for many immune-related pathways, including "Immune System", "Immune Disease", and "Infectious Disease", cytokine-related signaling pathways, "TNF signaling pathway", and "NF-κB" (Figure 2E). Of the TFs that have the highest contribution to modularity for Module 1, the majority have been validated as key regulators of colorectal cancer (Figure 2F). Indeed, 6 out of 10 leading TFs have direct evidence, and some experimental validation, of their involvement in colon cancer risk and progression: ZNF334 [104], FOXD2 [105], FOXD3 [106], POU3F3 [107], NR1H4 [108], and VSX2 [109].

We performed a similar analysis on all the other modules. Module 2 appears to be more cancer-specific as it is consistently enriched for cell cycle pathways ("Cell cycle", "Cellular senescence"), known cancer-related signaling pathways ("mTOR" and "JAK-STAT" signaling pathways),metabolic pathways, and cancer specific disease terms (Supplementary Figure S11). Module 4 is strongly enriched for signaling pathways, including "Wnt signaling", and development and regeneration pathways (Supplementary Figure S11). While we know that zinc finger proteins (ZNF) are still not well characterized, hindering the functional annotation of module 4where 9 out of 10 top TF are ZNFs, we found that ZNF341, which is the fourth highest-ranking hit formodularity contribution (Supplementary Figure S12), is a regulator of STAT3 and plays a key role in hyper-IgE syndrome (HIES), a condition that often manifests as severe and chronic bacterial infections [110]. In parallel, we found reports of associations between IgE-mediated immune reactions and colorectal

cancer risk [111]. Although we do not have definitive evidence or validation of the role of ZNF341 in the colon cancer subtypes, we believe that this suggestive result demonstrates how multi-omic network analysis can find complex regulatory patterns. Taken together, the analysis of Modules 1,2, and 4 shows that CMS2 and CMS4 at the regulatory level exhibit differences in the immunological response, cellular processes, such as growth and senescence, and signaling and developmental pathways."

* Are there any ALPACA modules that are similar to clusters A and D? This angle might help streamline the results section. It would also be interesting to have a glimpse of how results from these two analysis type overlap/relates. Also, since Alpaca is a great addition to the pipeline, I would suggest adding one or 2 panels from the Alpaca analysis to Figure 2. For instance, it could be module 1 from Figure S11 and top significant results from module 1 in Figure S12.

We thank the reviewer for the great suggestion! We have indeed changed the panel as suggested, adding the top pathways and TFs for module 1 to figure 2. We have also fixed the caption accordingly. We also looked into the overlap between DRAGON and ALPACA results finding an interesting trend of differential silencing between CMS2 and CMS4 for HOX TFs, that we now explain in the end of the results section and in Figure 2G.

"Finally, we examined the relationship between the results of the multi-omic DRAGON analysis (methylation-expression) and ALPACA. It is worth noting that DRAGON, as used here, estimates partial correlations between the methylation and expression of TFs, while ALPACA infers differential modularity between GRNs. Methylation is only one of many possible modifications that can change gene expression, translation, and regulation (mutations, CNVs, chromatin accessibility, etc.). Therefore, we do not necessarily expect DRAGON and ALPACA (used with PANDA-based GRNs) to be concordant. However, using the DRAGON results, we explored whether promoter methylation can explain the differential modularity of these colorectal cancer GRNs.

To interpret these results, we first note that DRAGON uses a different, more stringent filtering method than PANDA. Consequently, only about 50% of TFs in the ALPACAmodules can be found in the DRAGON networks (Figure 2G). Nevertheless, we investigated the representation of these overlapping TFs in the ALPACA modules that might indicate that epigenetic changes drive the regulatory differences in a specificModule. Specifically, we looked for TFs in ALPACA modules for which we have evidence of methylation-driven "silencing" in either CMS2 or CMS4, that are those with a strong negative association between expression and methylation of the same TF in DRAGON (Figure 2G, Supplementary Figure S13). While most "silenced" TFs are distributed across the ALPACA modules, in Module 11 we found both the TFs, HOXA10 and HOXB9, "silenced" in CMS2 and not CMS4. This result suggests that these TFs may play an important role in the overall differential regulation of gene expression in the two subtypes. It is also consistent with reports that HOX TFs are differentially accessible in colorectal cancer and that they are involved in cancer development and progression [96]."

##Minor comments##
* The authors write "We have gone through the paper and edited it to address missing words and incorrect word choice."; however this is questionable as there are still many instances of missing pieces. See: "we have implemented WGCNA [?] and GENIE3 [?], that are third-party network inference methods" , "However, netZooPy supports GPU-based com-putation of PANDA-LIONESS network [?].", "method section", "metabdata", ". specifically BATF". Also, the legend in Figure 2d was not updated; "D) Association graph for the TFs in cluster D for CMS2 (left) and CMS4 (right).". I would encourage a more careful read for the next submission.

We apologize and thank the reviewer for the careful re-read. Multiple coauthors have reviewed the manuscript and we are hopeful that the number of typos has been significantly reduced in number.

* "This effect is also visible also in the adjacency matrices representing the CNV-expression DRAGON networks, where a block structure in the CNV-CNV edges is evident (Supplementary Figure S9A)." -> unclear if "CNV-expression" was the right expression here; isn't Figure S9A showing only CNV-CNV edges? If the wording was correct, I would clarify the legend to explain better.

We thank the reviewer for pointing this out, and we have now rephrased to make it clearer.

We have revised the text as follows: “This effect was also visible in the adjacency matrices, where there is an evident block structure in the CNV-CNV edges (Supplementary Figure S9A). Moreover, we have evidence that, even in this case, DRAGON networks are able to capture direct effects of CNV onto expression; the CNV-expression edges on the same TFs (Ci, Ei) tend to be skewed towards positive values (Supplementary Figure S9B), which is expected behavior for somatic variation.”

* It is unclear to me how the modules displayed in Figure S12 were selected. For instance, module 6 is quite significant and was not displayed. Please clarify this selection in the legend.

We thank the reviewer as it gives us an opportunity to prepare a more easy-to-follow figure and write a clearer legend (this now appears as Figure S11)

* Figure S8: Add a light red bar or similar at p-value = 0.05 so that one can easily see the significance cutoff.

Thank you for the suggestion, we have now implemented it.

* In the computational performance part, if I understood correctly the new features are not benchmarked, including ALPACA. I would recommend updating the benchmark to include all features in the latest pipeline.

Thank you for pointing this out. We have now updated the test performance report and have included the correct link. We have also released v0.0.18 of the workflow.

* Figure S12 appears before Figure S11 in the text.

Thank you for noticing, we have now swapped them.

* Figure 2C: use 90 degree angles to show the x-axis labels (BATF, E2F2, …). Currently, it is difficult to map them to a given tick. I would also consider swapping this panel with Figure S8 as to my view it is more informative to highlight significant results, especially given the stated importance of immune infiltration in CSM4-like tumors.

We thank the reviewer for the suggestion; we have changed the orientation of the labels. The main reason for not including pathway analysis results on TFs is how hard it is to do any pathway analysis on such a small set of terms, as most pathways are annotated to less than 10 TFs. Given how small the dataset of interest is, we do believe that annotating these TFs to their respective pathways is useful to the reader to gain better insight.

* "Using functional enrichment analysis, we found that TFs in clusters A and D are involved in the metabolism of proteins and DNA repair and that cluster D, in contrast to cluster A, includes genes preferentially involved in the Immune System (Supplementary Figure S8). Specifically BATF, GATA3, NFATC3, NFATC2 and TP53 are involved in the Immune System, while E2F, TERF2, and TP53 participate in cellular responses to stimuli (Figure 2C)." -> this part should be re-written slightly to indicate the enrichment in the term "cellular responses to stimuli" and clarify that the genes listed are the one driving the enrichment.

We thank the reviewer for the suggestion and we have updated the text to:

“Using functional enrichment analysis, we found that TFs in clusters A and D are involved in the metabolism of proteins and DNA repair. TFs in cluster D, in contrast to cluster A, include genes preferentially involved in the Immune System (Supplementary Figure S8). These include BATF, GATA3, NFATC3, NFATC2, and TP53, which we found to be involved in the “Immune System” pathways (including “Signaling by the B Cell Receptor (BCR)” (nominal p-value : 0.023, OR: 13.8) and “Innate Immune System” (nominal p-value: 0.03, OR: 4.71), while E2F, TERF2, and TP53 participate in "Cellular responses to stimuli" pathways, including "DNA Damage/Telomere Stress Induced Senescence", (nominal p-value : 0.014, OR: 19.3), and "Cellular Senescence" (nominal p-value : 0.024, OR: 6.54) (Figure 2C).

We note that a general limitation of this functional enrichment analysis is that most TFs are only annotated to a small subset of pathways related to transcription and not comprehensively mapped to those on which they have a regulatory effect.”

* "We ran all tests with the v0.0.15 version of tcga-data-nf with standard configuration files and no parallelization of processes. The test full workflow requires 1h 11min of wall time (4.7 CPU hours) and a peak memory of 13.6 Gb for runTCGAPanda". -> What do you mean "no parallelization of processes"? I could not find such parameter in the documentation. Also, Nextflow automatically parallelizes jobs. And the wall time vs CPU hours indicates a degree of parallelization. Note, that this is also mentioned on the documentation but the clickable links yield "404 - page not found".

We thank the reviewer for the comment, because this sentence was indeed unclear. Nextflow parallelizes jobs based on the “executor” and “process” configurations. For the tests, we use a “process” definition that was using all resources available. This way nextflow runs one process after the other (yet the CPUs are parallelizing tasks). The reason to do this here is to show the maximum wall time used to run the tests, which is what the end user should expect when running this on a local machine, or before deciding how many resources to use on a HPC. We have changed the sentence accordingly and we have updated the documentation link.

“We report the performance on an AWS EC2 instance c5.4xlarge with 32Gb of memory and 16 vCPUs. We ran all tests with the v0.0.18 version of \nf with standard configuration files that allow nextflow to run all jobs serially, with no job parallelization, where each process is allowed to use all computing resources available. For reference, this would be consistent with testing the workflow on a local machine.

* "Within the top 20 TFs that show high correlation between CNV and expression, we observe known colon cancer drivers [83] ZNF703 and SMAD4, and IRF2 and GATA6 whose DNA aberrations are likely oncogenic [78]."
Where is this data shown? Also, what is the difference with the next text part: "Finally, we identify which TFs have the most different CNV-expression edges between the CMS2 and CMS4 subtypes."

We thank the reviewer for pointing this out. The figure is the same one referenced in the sentence before, but we have now added the reference again to make it clearer to the reader. Also, the beginning of the next paragraph was confusing, so we have now rewritten it.

“...Moreover, we have evidence that, even in this case, DRAGON networks are able to capture direct effects of CNV onto expression; the CNV-expression edges on the same TFs (C_i, E_i) tend to be skewed towards positive values (Supplementary Figure S9B), which is expected behavior for somatic variation. Within the top 20 TFs that showed high correlation between their own CNV and expression, we observed known colon cancer drivers [86] ZNF703 andSMAD4, and IRF2 and GATA6, whose DNA aberrations are likely oncogenic [81] (Supplementary Figure S9B). Finally, we explored the strongest differences between CMS2 and CMS4

subtypes in terms of partial correlations between CNV and expression. For each CNV node Ci, we compared the edge set {(Ci, Ej)} between the two subtypes. We conducted a paired Wilcoxon signed-rank test for each TF i, testing the null hypothesis that there is no difference in CNV-expression edge weights between CMS2 and CMS4 (Supplementary Figure S9C). Among the top TFs with varying edges between CMS2 and CMS4 based on the Wilcoxon test results were ZNF554, for which we have partial evidence of involvement in cancer progression through the WNT/β-Catenin Signaling Pathway [99], KLF16, which plays a role in the stress-related programming of colorectal cancer [100], and DOT1L, amethyltransferase that regulates core stem cell genes and affects tumorigenesis and drug resistance [101]. These TFs are significantly associated with colon cancer drivers. When comparing the Wilcoxon test statistics of the cancer TFs to the rest of the TFs, we get a significant Kolmogorov-Smirnov test (stat: 0.21, p-value: 0.013). Also, there are 27 significant (FDR < 0.01) TFs that are annotated by either the Cancer Gene Census or the DisGeNET dataset as colon cancer drivers (Fisher’s exact test OR : 1.6, p-value: 0.057, Supplementary Figure S9D). Among the cancer drivers that show the biggest association changes between CMS2 and CMS4, we found HIF1A and CUX1, both of which are implicated in tumorigenesis and progression [87], and GATA4, which controls senescence [102, 103].”

*   "Interestingly, module 4 is instead uniquely involved into pathways that control the Extracellular Matrix Organization." I could also not find this result in the figure. Also, why is this relevant? (include reference if needed).

We thank the reviewer for spotting this and we apologize for the mistake. This is a result we found in a previous analysis but isn’t shown in any of the figures. We have now removed it. Also, that paragraph has now been updated and extended.

*   "Lastly, while many zinc finger proteins are still not well characterized, ZNF341 is a regulator of STAT3" -> Please clarify that most TFs with the highest modularity in module 4 were encoding for zing finger proteins. Also, clarify that ZNF341 was the fourth hit in this module.

We thank the reviewer for pointing this out and we have now rewritten the text to include their comments.

##Recommendations##
*   Currently, the ending is a bit negative. I would add one or 2 sentences at the very end of the discussion to summarize the main advantages of your pipeline and how it could be used or expanded in future work.

*   "Modules 1, 2, and 4, are those the exhibit the biggest differences in connectivity" -> " the most significant difference in connectivity"

*   Consistent usage of the past tense in the results section would likely be better (i.e., "We investigated which pathways are enriched", "we extend our search beyond")

*   The expression "third-party network" gives a business tone; I would recommend replacing it with "externally developed network inference methods", or "community-developed network inference methods"

We truly thank the reviewer for this detailed review! We have also implemented these recommendations since we agree that they improve the overall clarity and quality of the paper.

Cookie Preference Center

We use cookies which are necessary to make our site work. We may also use additional cookies to analyze, improve and personalize our content and your digital experience. For more information, see our Cookie Policy.

You may choose not to allow some types of cookies. However, blocking some types may impact your experience of our site and the services we are able to offer. See the different category headings below to find out more or change your settings.

Allow all

Manage Consent Preferences

Strictly Necessary Cookies

Always active

These cookies are necessary for the website to function and cannot be switched off in our systems. They are usually only set in response to actions made by you which amount to a request for services, such as setting your privacy preferences, logging in or filling in forms. You can set your browser to block or alert you about these cookies, but some parts of the site will not then work.

Functional Cookies

These cookies enable the website to provide enhanced functionality and personalisation. They may be set by us or by third party providers whose services we have added to our pages. If you do not allow these cookies then some or all of these services may not function properly.

Performance Cookies

These cookies allow us to count visits and traffic sources so we can measure and improve the performance of our site. They help us to know which pages are the most and least popular and see how visitors move around the site.

Targeting Cookies

These cookies may be set through our site by our advertising partners. They may be used by those companies to build a profile of your interests and show you relevant adverts on other sites. If you do not allow these cookies, you will experience less targeted advertising.

Cookie List

Clear

Apply Cancel
Consent Leg.Interest

Confirm my choices

Powered by onetrust
