# Supplementary material for: Reproducible processing of TCGA regulatory networks
Source: Gigascience. 2025 Oct 20;14:giaf126. doi: 10.1093/gigascience/giaf126 (PMC12720619; doi:10.1093/gigascience/giaf126)
Supplement: giaf126_Reviewer_1_Report_Original_Submission — Xi Chen -- 1/26/2025 [file giaf126_reviewer_1_report_original_submission.pdf]

Reviewer Report

Title: Reproducible processing of TCGA regulatory networks

Version: Original Submission **Date:** 1/26/2025

Reviewer name: Xi Chen

Reviewer Comments to Author:

Fanfani et al. present tcga-data-nf, a Nextflow pipeline that streamlines the download, preprocessing, and network inference of TCGA bulk data (gene expression and DNA methylation). Alongside this pipeline, they introduce NetworkDataCompanion (NDC), an R package designed to unify tasks such as sample filtering, identifier mapping, and normalization. By leveraging modern workflow toolsâ€"Nextflow, Docker, and condaâ€"they aim to provide a platform that is both reproducible and transparent. The authors illustrate the pipeline's utility with a colon cancer subtype example, showing how multi-omics networks (inferred via PANDA, DRAGON, and LIONESS) may help pinpoint epigenetic factors underlying more aggressive tumor phenotypes. Overall, this work addresses a clear need for standardized approaches in large-scale cancer bioinformatics.While tcga-data-nf promises a valuable resource, the following issues should be addressed more thoroughly before publication:1. While PANDA, DRAGON, and LIONESS form a cohesive system, they were all developed by the same research group. To strengthen confidence, please include head-to-head comparisons with other GRN inference methods (e.g., ARACNe, GENIE3, Inferelator). A small benchmark dataset with known ground-truth (or partial experimental validation) would be especially valuable.2. Although the manuscript identifies intriguing TFs and pathways, it lacks confirmation through orthogonal data or experiments. If available, consider including ChIP-seq or CRISPR-based evidence to reinforce at least a subset of inferred regulatory interactions. Even an in silico overlap with known TF-binding sites or curated gene sets would help validate the predictions.3. PANDA and DRAGON emphasize correlation/partial correlation, so they may overlook nonlinear or combinatorial regulation. If feasible, please provide any preliminary steps taken to capture nonlinearities or discuss approaches that could be integrated into the pipeline.4. LIONESS reconstructs a network for each sample in a leave-one-out manner, which can be demanding for large cohorts. The paper does not mention runtime or memory requirements. Adding a Methods subsection with approximate CPU/memory benchmarks (e.g., "On an HPC cluster with X cores, building LIONESS networks for 500 samples took Y hours") is recommended to guide prospective users.5. Currently, the pipeline only covers promoter methylation and standard gene expression, yet TCGA and related projects include other data types (e.g., miRNA, proteomics, histone modifications). If possible, offer a brief example or instructions on adding new omics layers, even conceptually.6. Recent methods often target single-cell RNA-seq, but tcga-data-nf is geared toward bulk datasets. Please clarify limitations and potential extensions for single-cell or multi-region tumor data. This would help readers understand whether (and how) the pipeline could be adapted to newer high-resolution profiles.Minor point:1. Provide clear guidance on cutoffs for low-expressed genes, outlier samples, and methylation missing-value imputation.2. Consider expanding the supplement with a "quick-start" guide, offering step-by-step

usage examples.3. Ensure stable version tagging in your GitHub repository so that readers can reproduce the exact pipeline described in the manuscript.

Level of Interest

Please indicate how interesting you found the manuscript: Choose an item.

Quality of Written English

Please indicate the quality of language in the manuscript: Choose an item.

Declaration of Competing Interests

Please complete a declaration of competing interests, considering the following questions:

- Have you in the past five years received reimbursements, fees, funding, or salary from an organisation that may in any way gain or lose financially from the publication of this manuscript, either now or in the future?
- Do you hold any stocks or shares in an organisation that may in any way gain or lose financially from the publication of this manuscript, either now or in the future?
- Do you hold or are you currently applying for any patents relating to the content of the manuscript?
- Have you received reimbursements, fees, funding, or salary from an organization that holds or has applied for patents relating to the content of the manuscript?
- Do you have any other financial competing interests?
- Do you have any non-financial competing interests in relation to this paper?

If you can answer no to all of the above, write 'I declare that I have no competing interests' below. If your reply is yes to any, please give details below.

None

I agree to the open peer review policy of the journal. I understand that my name will be included on my report to the authors and, if the manuscript is accepted for publication, my named report including any attachments I upload will be posted on the website along with the authors' responses. I agree for my report to be made available under an Open Access Creative Commons CC-BY license (http://creativecommons.org/licenses/by/4.0/). I understand that any comments which I do not wish to be included in my named report can be included as confidential comments to the editors, which will not be published.

Choose an item.

To further support our reviewers, we have joined with Publons, where you can gain additional credit to further highlight your hard work (see: https://publons.com/journal/530/gigascience). On publication of this paper, your review will be automatically added to Publons, you can then choose whether or not to claim your Publons credit. I understand this statement.

Yes Choose an item.
