# Supplementary material for: Reproducible processing of TCGA regulatory networks
Source: Gigascience. 2025 Oct 20;14:giaf126. doi: 10.1093/gigascience/giaf126 (PMC12720619; doi:10.1093/gigascience/giaf126)
Supplement: giaf126_Reviewer_2_Report_Original_Submission — Jérôme Salignon -- 2/12/2025 [file giaf126_reviewer_2_report_original_submission.pdf]

Reviewer Report

Title: Reproducible processing of TCGA regulatory networks

Version: Original Submission **Date:** 2/12/2025

Reviewer name: JÃ©rÃ´me Salignon

Reviewer Comments to Author:

This manuscript presents tcga-data-nf, a Nextflow-based pipeline for downloading, preprocessing, and analyzing TCGA multi-omic data, with a focus on gene regulatory network (GRN) inference. The workflow integrates established bioinformatics tools (PANDA, DRAGON, and LIONESS) and adheres to best practices for reproducibility through containerization (Docker, Conda, and Nextflow profiles). The authors demonstrate the utility of their pipeline by applying it to colorectal cancer subtypes, identifying potential regulatory interactions in TGF-Î² signaling.The manuscript is well-written and well-structured and provides sufficient methodological details, as well as Jupyter notebooks, for reproducibility. However, there are some areas that require clarification and improvement for acceptance in GigaScience, particularly regarding the scope of the tool, the quality of the inferred regulatory networks, the case study figure, benchmarking, statistical validation, and parameters.Major comments:* While the pipeline is well designed and executed, the overall impact of the tool feels somewhat limited, especially for a journal like GigaScience, due to its pretty specific application to building GRNs in TCGAs, the relatively small number of parameters, the support of only 2 omics type, and the lack of novel algorithms. To increase the impact of this tool I would recommend adding functionalities, such as:o Supporting additional tools. A great strength of the pipeline is the integration with the Network Zoo (NetZoo) ecosystem. However, only three tools are included from NetZoo. Including additional tools would likely increase the scope of users interested in using the pipeline. In particular, an important weakness of the current pipeline is that it is not possible to conduct differential analysis between different networks, which prevents users from identifying the most significant differences between two networks of interest (e.g., CMS2 vs CMS4). The NetZoo contains different tools to conduct such analyses, such as Alpaca 1 or Crane 2, thus this may be implemented to make the pipeline more useful to a broader user base.o Adding parameters. A strength of the pipeline is the ability to customize it using various parameters. However, as such the pipeline does not offer many parameters. It would be beneficial to make the pipeline a bit more customizable. For example, novel parameters could be: adding options for excluding selected samples, using different batch correction methods, different methods to map CpGs to genes, additional normalization methods, and additional quality controls (e.g., PCA for methylation samples, md5sum checks). These are just examples and do not need to be all implemented but adding some extra parameters would help make the pipeline more appealing and customizable to various users.* The quality of the inferred regulatory networks is hard to judge. There are no direct comparisons with any other tools.o For instance, it is mentioned in the text that GRAND networks were derived using a fixed set of parameters, but it could be helpful to show a direct comparison between GRNs built from your tools with those from GRAND. This could reveal how the ability to customize GRNs using the pipeline's parameters helps in getting better biological insights.o

Alternatively, or in addition, one could compare how networks built by your method fare in comparison to networks built from other methods, like RegEnrich 3 or NetSeekR 4, in terms of biological insights, accuracy, scalability, speed, functionalities and/or memory usage.o Another angle to judge the regulatory networks would be to check in a case study if the predicted gene interactions between disease and control networks are enriched in disease and gene-gene interactions databases, such as DisGeNet 5.* Figure 2 needs re-work:o Panel A and C: text is too small. "tf" should be written TF. "oi" should have another name. These panels might be moved to the supplements.o Panel D is confusing. Without significance it is hard to understand what the point of this panel is. I can see that certain TFs are cited in the main text but without information about significance, these may seem like cherry-picking. The legends states: `Annotation of all TFs in cluster D (columns) to the Reactome parent term. "Immune system" and "Cellular respondes to stimuli" are more consistenly involved in cluster D, in comparison to cluster A.`. However, this is a key result which should be shown in a main figure, not in Figure S6. I would also recommend using a -log scale when displaying the p-values to highlight the most significant entries.o Panel E is quite confusing; first, the color coding is unclear. For instance, what represents blue, purple and red colors? Second, what represents the edges' widths? I would recommend using different shapes for the methylation and expression nodes to reduce the number of colors, and adding a color legend. I would also consider merging the two graphs and representing in color the difference in the edge values so the reader can directly see the key differences.* Benchmarking analysis could be included to show the runtime and memory requirement for each pipeline step. It would also be beneficial to analyze a larger dataset than colon cancer to assess the scalability.* Statistical analysis: If computationally feasible, permutation testing could be implemented to quantify the robustness of inferred regulatory interactions. Also, in the method section, it should be clarified that FDR correction was applied for pathway enrichment analysis.Minor comments:* I am not sure why duplicate samples are discarded in the pipeline. Why not add counts for RNA-Seq and averaging beta values? I would expect that to yield more robust results.* It is a bit unclear in what context the NetworkDataCompanion tool could be used outside the workflow. It is also unclear how it helps with quality controls. Please clarify these aspects.* The manuscript is well-written, but words are sometimes missing or wrongly written, it needs careful re-read.* The expression '"same-same"' is unclear to me.* In this sentence: "Some of "same-same" genes (STAT5A, CREB3L1"…, I am not sure in which table or figure I can find this result?* Text is too small in the Directed Acyclic Graph, especially in Figure S4. Also, I would recommend adding the Directed Acyclic Graphs from Figure S1-S4 to the online documentation.* Regarding the code, I was puzzled to see a copyConfigFiles process. Also, there are files in `bin/r/local_assets`, these should be located in `assets`. And the container for the singularity and docker profile is likely the same, this should be clarified in the code.* It is recommended to remove the "defaults" channel from the list of channels declared in the `containers/conda_envs/analysis.yml` file. Please see information about that here https://www.anaconda.com/blog/is-conda-free and here https://www.theregister.com/2024/08/08/anaconda_puts_the_squeeze_on/.Additional comments (which do not need to be addressed):* Future work may consider enabling the use of the pipeline to build GRNs from other data sources than TCGA (i.e., nf-netzoo). Recount3 data is already being parsed for GTEx and TCGA samples, so it might be relatively easy to adapt the pipeline so that it can be used on any arbitrary recount3 dataset. Similarly, it could be useful if one could specify a dataset on the recountmethylation database 6 to build GRNs. While these unimodal datasets could not be used with

the DRAGON method they would still benefit from all other features of the pipeline.* Using a nf-core template would enable better structure of the code and increase the visibility of the tool. Also using multiple containers is usually easier to maintain and update than a single large container, especially when a single tool needs to be updated or when modifying part of the pipeline. Another comment is that the code contains many comments which are not to explain the code but more like quick draft which makes the code harder to read by others.References1. Padi, M., and Quackenbush, J. (2018). Detecting phenotype-driven transitions in regulatory network structure. npj Syst Biol Appl 4, 1-12. https://doi.org/10.1038/s41540-018-0052-5.2. Lim, J.T., Chen, C., Grant, A.D., and Padi, M. (2021). Generating Ensembles of Gene Regulatory Networks to Assess Robustness of Disease Modules. Front. Genet. 11. https://doi.org/10.3389/fgene.2020.603264.3. Tao, W., Radstake, T.R.D.J., and Pandit, A. (2022). RegEnrich gene regulator enrichment analysis reveals a key role of the ETS transcription factor family in interferon signaling. Commun Biol 5, 1-12. https://doi.org/10.1038/s42003-021-02991-5.4. Srivastava, H., Ferrell, D., and Popescu, G.V. (2022). NetSeekR: a network analysis pipeline for RNA-Seq time series data. BMC Bioinformatics 23, 54. https://doi.org/10.1186/s12859-021-04554-1.5. Hu, Y., Guo, X., Yun, Y., Lu, L., Huang, X., and Jia, S. (2025). DisGeNet: a disease-centric interaction database among diseases and various associated genes. Database 2025, baae122. https://doi.org/10.1093/database/baae122.6. Maden, S.K., Walsh, B., Ellrott, K., Hansen, K.D., Thompson, R.F., and Nellore, A. (2023). recountmethylation enables flexible analysis of public blood DNA methylation array data. Bioinformatics Advances 3, vbad020. https://doi.org/10.1093/bioadv/vbad020.

Level of Interest

Please indicate how interesting you found the manuscript: Choose an item.

Quality of Written English

Please indicate the quality of language in the manuscript: Choose an item.

Declaration of Competing Interests

Please complete a declaration of competing interests, considering the following questions:

- Have you in the past five years received reimbursements, fees, funding, or salary from an organisation that may in any way gain or lose financially from the publication of this manuscript, either now or in the future?
- Do you hold any stocks or shares in an organisation that may in any way gain or lose financially from the publication of this manuscript, either now or in the future?
- Do you hold or are you currently applying for any patents relating to the content of the manuscript?
- Have you received reimbursements, fees, funding, or salary from an organization that holds or has applied for patents relating to the content of the manuscript?

- Do you have any other financial competing interests?
- Do you have any non-financial competing interests in relation to this paper?

If you can answer no to all of the above, write 'I declare that I have no competing interests' below. If your reply is yes to any, please give details below.

I declare that I have no competing interests.

I agree to the open peer review policy of the journal. I understand that my name will be included on my report to the authors and, if the manuscript is accepted for publication, my named report including any attachments I upload will be posted on the website along with the authors' responses. I agree for my report to be made available under an Open Access Creative Commons CC-BY license (http://creativecommons.org/licenses/by/4.0/). I understand that any comments which I do not wish to be included in my named report can be included as confidential comments to the editors, which will not be published.

Choose an item.

To further support our reviewers, we have joined with Publons, where you can gain additional credit to further highlight your hard work (see: https://publons.com/journal/530/gigascience). On publication of this paper, your review will be automatically added to Publons, you can then choose whether or not to claim your Publons credit. I understand this statement.

Yes Choose an item.
