# Supplementary material for: Reproducible processing of TCGA regulatory networks
Source: Gigascience. 2025 Oct 20;14:giaf126. doi: 10.1093/gigascience/giaf126 (PMC12720619; doi:10.1093/gigascience/giaf126)
Supplement: giaf126_Reviewer_2_Report_Revision_1 — Jérôme Salignon -- 5/13/2025 [file giaf126_reviewer_2_report_revision_1.pdf]

Reviewer Report

Title: Reproducible processing of TCGA regulatory networks

Version: Revision 1 **Date:** 5/13/2025

Reviewer name: JÃ©rÃ´me Salignon

Reviewer Comments to Author:

The authors have developed a well-structured and reproducible workflow for regulatory network inference from TCGA data, integrating established tools and offering valuable infrastructure for the community. The additions of ALPACA, support for more omics types, and improved parameter flexibility are welcome enhancements. However, further work is needed to strengthen the impact of the pipeline, particularly through clearer demonstration of the biological significance of findings (e.g., through gene sets enrichment analyses). This point if of particular importance, given the lack of benchmark with other tools, and will help better position this resource for broader adoption and scientific utility.##Majors comments##* Figure 2D: The legend was not changed, it still reads: "Association graph for the TFs in cluster D for CMS2 (left) and CMS4 (right)." This should be fixed. The accompanying text for this panel is "All these differences between CMS2 and CMS4 can also be visualized at once as the subgraph that emerges from cluster D, Figure 2D.", which is clearly not very insightful. I would suggest either describing key insights from this panel, or moving it to the supplements. In the first case, we can now clearly see that some edges are much stronger; like TP53 to POU2F2. It may be interesting to describe in particular such examples, if relevant.* Figure S5: This figure is not very informative as one doesn't know if the presence of certain genes from your network in various databases is higher than expected or just as expected by chance. As such, the figure is of little value, and it is hard to conclude much. I would recommend conducting gene set enrichment analysis to see which gene set (CMS4, CMS2, cluster A, ect) is enriched in which database, and showing the results in an additional supplementary figure. Also in the sentence: "edges in cluster A of the DRAGON methylation-expression networks are enriched for TFs that are involved in Colorectal Neoplasms according to the DisGeNet" -> I could not find this information easily/clearly in the Figure. Also, if this is a relevant result it should be mentioned in the main text, not only in the letter to the editor. Enrichment of cancer gene sets in some of your subtypes/clusters could be quite convincing/informative. In terms of style: I would put x-axis labels in a 90 degree angle.* "and colon cancer drivers HIF1A and CUX1, both of which are implicated in tumorigenesis and progression [84]"; I could not find CUX1 in the list. Also going up to 50 genes seems a bit extreme; I would not describe any gene more than the top 10 or 20. If one wants to include more genes I would do pathway enrichment analysis to show more systematically what this list of genes contains. Because out of 50 genes, one can most likely pick a few that support any given hypothesis.* "Modules 1 and 2 are enriched for Immune System and Cell Cycle pathways" -> I could not find enrichment for "Cell Cycle pathways" on the figure. Also, and more importantly, I think displaying the number of enriched KEGG subcategories is peculiar and uncommon. This type of analysis can hide for instance a very significant term. I think the authors should provide, in addition, a table with the top significant results to ensure the immune terms are indeed the most significant results. Alternatively, I would directly show the most significant terms on

the y-axis of Figure S12.* Are there any ALPACA modules that are similar to clusters A and D? This angle might help streamline the results section. It would also be interesting to have a glimpse of how results from these two analysis type overlap/relates. Also, since Alpaca is a great addition to the pipeline, I would suggest adding one or 2 panels from the Alpaca analysis to Figure 2. For instance, it could be module 1 from Figure S11 and top significant results from module 1 in Figure S12.##Minor comments##* The authors write "We have gone through the paper and edited it to address missing words and incorrect word choice."; however this is questionable as there are still many instances of missing pieces. See: "we have implemented WGCNA [?] and GENIE3 [?], that are third-party network inference methods" , "However, netZooPy supports GPU-based com-putation of PANDA-LIONESS network [?].", "method section", "metabdata", ". specifically BATF". Also, the legend in Figure 2d was not updated; "D) Association graph for the TFs in cluster D for CMS2 (left) and CMS4 (right).". I would encourage a more careful read for the next submission.* "This effect is also visible also in the adjacency matrices representing the CNV-expression DRAGON networks, where a block structure in the CNV-CNV edges is evident(Supplementary Figure S9A)." -> unclear if "CNV-expression" was the right expression here; isn't Figure S9A showing only CNV-CNV edges? If the wording was correct, I would clarify the legend to explain better.* It is unclear to me how the modules displayed in Figure S12 were selected. For instance, module 6 is quite significant and was not displayed. Please clarify this selection in the legend.* Figure S8: Add a light red bar or similar at p-value = 0.05 so that one can easily see the significance cutoff.* In the computational performance part, if I understood correctly the new features are not benchmarked, including ALPACA. I would recommend updating the benchmark to include all features in the latest pipeline.* Figure S12 appears before Figure S11 in the text.* Figure 2C: use 90 degree angles to show the x-axis labels (BATF, E2F2, …). Currently, it is difficult to map them to a given tick. I would also consider swapping this panel with Figure S8 as to my view it is more informative to highlight significant results, especially given the stated importance of immune infiltration in CSM4-like tumors.* "Using functional enrichment analysis, we found that TFs in clusters A and D are involved in the metabolism of proteins and DNA repair and that cluster D, in contrast to cluster A, includes genes preferentially involved in the Immune System (Supplementary Figure S8). specifically BATF, GATA3, NFATC3, NFATC2 and TP53 are involved in the Immune System, while E2F, TERF2, and TP53 participate in cellular responses to stimuli (Figure 2C)." -> this part should be re-written slightly to indicate the enrichment in the term "cellular responses to stimuli" and clarify that the genes listed are the one driving the enrichment.* "We ran all tests with the v0.0.15 version of tcga-data-nf with standard configuration files and no parallelization of processes. The test full workflow requires 1h 11min of wall time (4.7 CPU hours) and a peak memory of 13.6 Gb for runTCGAPanda". -> What do you mean "no parallelization of processes"? I could not find such parameter in the documentation. Also, Nextflow automatically parallelizes jobs. And the wall time vs CPU hours indicates a degree of parallelization. Note, that this is also mentioned on the documentation but the clickable links yield "404 - page not found".* "Within the top 20 TFs that show high correlation between CNV and expression, we observe known colon cancer drivers [83] ZNF703 and SMAD4, and IRF2 and GATA6 whose DNA aberrations are likely oncogenic [78]."Where is this data shown? Also, what is the difference with the next text part: "Finally, we identify which TFs have the most different CNV-expression edges between the CMS2 and CMS4 subtypes."* "Interestingly, module 4 is instead uniquely involved into pathways that control the Extracellular Matrix Organization." I could also not find this result in the figure. Also, why is this

relevant? (include reference if needed).* "Lastly, while many zinc finger proteins are still not well characterized, ZNF341 is a regulator of STAT3" -> Please clarify that most TFs with the highest modularity in module 4 were encoding for zing finger proteins. Also, clarify that ZNF341 was the fourth hit in this module.##Recommendations##* Currently, the ending is a bit negative. I would add one or 2 sentences at the very end of the discussion to summarize the main advantages of your pipeline and how it could be used or expanded in future work.* "Modules 1, 2, and 4, are those the exhibit the biggest differences in connectivity" -> " the most significant difference in connectivity"* Consistent usage of the past tense in the results section would likely be better (i.e., "We investigated which pathways are enriched", "we extend our search beyond")* The expression "third-party network" gives a business tone; I would recommend replacing it with "externally developed network inference methods", or "community-developed network inference methods"

Level of Interest

Please indicate how interesting you found the manuscript: Choose an item.

Quality of Written English

Please indicate the quality of language in the manuscript: Choose an item.

Declaration of Competing Interests

Please complete a declaration of competing interests, considering the following questions:

- Have you in the past five years received reimbursements, fees, funding, or salary from an organisation that may in any way gain or lose financially from the publication of this manuscript, either now or in the future?
- Do you hold any stocks or shares in an organisation that may in any way gain or lose financially from the publication of this manuscript, either now or in the future?
- Do you hold or are you currently applying for any patents relating to the content of the manuscript?
- Have you received reimbursements, fees, funding, or salary from an organization that holds or has applied for patents relating to the content of the manuscript?
- Do you have any other financial competing interests?
- Do you have any non-financial competing interests in relation to this paper?

If you can answer no to all of the above, write 'I declare that I have no competing interests' below. If your reply is yes to any, please give details below.

I declare that I have no competing interests

I agree to the open peer review policy of the journal. I understand that my name will be included on my report to the authors and, if the manuscript is accepted for publication, my named report including any attachments I upload will be posted on the website along with the authors' responses. I agree for my report to be made available under an Open Access Creative Commons CC-BY license (http://creativecommons.org/licenses/by/4.0/). I understand that any comments which I do not wish to be included in my named report can be included as confidential comments to the editors, which will not be published.

Choose an item.

To further support our reviewers, we have joined with Publons, where you can gain additional credit to further highlight your hard work (see: https://publons.com/journal/530/gigascience). On publication of this paper, your review will be automatically added to Publons, you can then choose whether or not to claim your Publons credit. I understand this statement.

Yes Choose an item.
